# CCR2 deficiency protects against doxorubicin-induced cardiac dysfunction through enhanced IL12B-dependent autophagy

**DOI:** 10.7150/thno.131005

**Published:** 2026-07-22

**Authors:** Lizhi Hu, Li Lin, Long Chen, Yuhang Wang, Lulu Ning, Wanheng Tu, Cheng Wang, Shan Deng, Kai Huang

**Affiliations:** 1 Clinic Center of Human Gene Research, Union Hospital, Tongji Medical College, Huazhong University of Science and Technology, Wuhan, China.; 2 Hubei Key Laboratory of Metabolic Abnormalities and Vascular Aging, Huazhong University of Science and Technology, Wuhan, China.; 3 Hubei Clinical Research Center of Metabolic and Cardiovascular Disease, Huazhong University of Science and Technology, Wuhan, China.; 4 Department of Cardiology, Union hospital, Tongji Medical College, Huazhong University of Science and Technology, Wuhan, China.

**Keywords:** doxorubicin, cardiotoxicity, CCR2, autophagy, IL12B

## Abstract

**Rationale:**

Doxorubicin (DOX) is a potent chemotherapeutic agent whose antitumor benefits are limited by a well-recognized, dose-dependent cardiotoxicity. While previous studies have implicated inflammatory pathways in DOX-induced cardiomyopathy (DIC), the role of CCR2 in this process remains incompletely defined. This study aims to investigate whether CCR2 deficiency confers cardioprotection against DIC and to uncover the molecular mechanisms involved.

**Methods:**

CCR2 knockout (*CCR2^⁻/⁻^*) mouse was subjected to both acute and chronic DIC models. Bone marrow transplantation was used to establish the functional contribution of CCR2-deficient macrophages. Autophagic flux was evaluated using complementary approaches, including a tandem mRFP-GFP-LC3 reporter, western blotting, immunofluorescence, and transmission electron microscopy. The mediator linking CCR2-deficient macrophages to cardiomyocytes was identified by proteomics and validated using recombinant IL12B protein and a neutralizing antibody.

**Results:**

CCR2 deficiency substantially improved cardiac function, as evidenced by preserved left ventricular ejection fraction, fractional shortening and reduced serum cardiac injury markers. Mechanistic studies revealed that* CCR2^⁻/⁻^* hearts exhibited enhanced autophagic flux, with increased LC3B lipidation, autophagosome formation, and clearance of damaged cellular components. Proteomic profiling of cardiac macrophages identified interleukin-12B (IL12B) significantly upregulated in *CCR2^⁻/⁻^* mouse. Recombinant IL12B protein administration activated cardiomyocyte autophagy through PI3K/Akt/mTOR pathway inhibition and reproduced the cardioprotective effects in WT mouse. Conversely, IL12B neutralization completely abolished CCR2 deficiency-mediated protection.

**Conclusions:**

Our findings identify a novel CCR2-IL12B-autophagy axis that critically regulates DOX-induced cardiotoxicity. CCR2 deficiency promotes IL12B secretion from cardiac macrophages, which directly activates protective autophagy in cardiomyocytes. These results establish CCR2 inhibition and IL12B supplementation as two promising therapeutic strategies to prevent chemotherapy-induced cardiomyopathy, providing a transformative approach to cardio-oncology.

## Introduction

Doxorubicin (DOX) is a widely used anthracycline chemotherapeutic agent against a range of hematological and solid tumors. However, its clinical application is hampered by a well-documented, dose-dependent cardiotoxicity. A substantial proportion of patients receiving DOX chemotherapy eventually develop congestive heart failure 1, 2. Consequently, elucidating the underlying mechanisms and identifying optimal therapeutic strategies for DOX-induced cardiomyopathy (DIC) are critically important. Previous studies have proposed several pathogenic mechanisms of DIC, including DNA damage, oxidative stress, mitochondrial dysfunction, and inflammatory responses 3-6. Additionally, emerging evidence suggests that autophagy dysregulation is involved in the progression of DIC 7.

Autophagy is a conserved cellular process that targets cytoplasmic cargo for lysosomal degradation. The resulting degradation products serve as essential biosynthetic precursors, facilitating the continuous renewal of proteins and organelles to maintain cellular homeostasis 8. Increasing evidence highlights the critical role of autophagy in cardiac homeostasis 9, and numerous studies have identified autophagy as a promising therapeutic target for DIC. For example, Wang *et al*. demonstrated that autophagy is transiently activated during the early phase of DIC but is suppressed in later stages, indicating that timely activation of autophagy may represent a viable treatment strategy 10. Similarly, Li *et al*. reported that PI3Kγ knockout enhances mitophagy and alleviates DOX-induced cardiac dysfunction 11. Furthermore, Santin *et al*. reported that the administration of acidic nanoparticles protects against doxorubicin cardiotoxicity by enhancing lysosomal function and autophagic flux 12. Together, these findings highlight the essential role of autophagy in promoting cardiomyocyte survival and stress adaptation under pathological conditions.

C-C chemokine receptor type 2 (CCR2) is a chemokine receptor that primarily binds to CCL2, along with other ligands, including CCL7 and CCL12 13, 14. When inflammation or tissue injury occurs, chemokines such as CCL2 become highly upregulated. This increase in CCL2 expression facilitates the mobilization of CCR2⁺ monocytes from the bone marrow to the spleen and ultimately to injury sites, where they differentiate into macrophages 15, 16. Notably, therapeutic blockade of the CCL2/CCR2 axis has shown promise in treating myocardial infarction, atherosclerosis and cancer, although its role in DIC remains unexplored 17-19.

In this study, we investigated CCR2 function in DIC using *CCR2^-/-^* mouse. Our results demonstrate that CCR2 knockout significantly ameliorates DIC in both acute and low-dose models. Importantly, pharmacological inhibition of CCR2 using a specific antagonist similarly attenuated DIC but also preserved the antitumor efficacy of DOX in 4T1 breast cancer model, suggesting its potential clinical applicability. Mechanistic studies revealed that CCR2-deficient macrophages secrete IL12B, which acts directly on cardiomyocytes to inhibit the PI3K/Akt/mTOR pathway and subsequently increase autophagic flux. This increase in autophagy maintained cardiac homeostasis during DOX challenge. Although IL12B is well characterized as a partner of IL12A in the formation of heterodimeric cytokine IL12 with established roles in innate and adaptive immunity 20, 21, and also plays functions in autophagy 22, 23, its potential involvement in cardiovascular pathophysiology remains unexplored. Using complementary *in vitro* and *in vivo* approaches, we found that recombinant IL12B protein administration recapitulated the cardioprotective effects of CCR2 ablation and that the beneficial effects of CCR2 knockout were IL12B dependent. These findings collectively identify a CCR2/IL12B/autophagy axis involved in DIC pathogenesis, revealing potential therapeutic targets for preventing DIC.

## Methods

### Animals and ethical approval

Male C57BL/6J mouse and female BALB/c mouse (8 weeks old) were obtained from Beijing HFK Bioscience company. CCR2 knockout mouse (C57BL/6 genetic background) was bred and maintained at the Huazhong University of Science and Technology animal facility. All animal experiments were carried out in strict compliance with the National Institutes of Health Guide for the Care and Use of Laboratory Animals and were approved by the Institutional Animal Care and Use Committee of Huazhong University of Science and Technology (Animal protocol number: 4755). In accordance with institutional animal welfare guidelines, the maximal tumor burden permitted was 1.5 cm in any dimension. We confirm that this limit was not exceeded during the study, with tumor dimensions monitored regularly to ensure compliance with ethical standards. Mouse was anesthetized with a single injection of 0.5% pentobarbital sodium (50 mg/kg, intraperitoneally) prior to echocardiography and other experimental procedures. Euthanasia was performed by administration of a pentobarbital sodium overdose (150 mg/kg, intraperitoneally) following the completion of all experimental procedures. All experiments adhered to protocols designed to minimize animal suffering.

### DIC model

Acute DIC was induced in male mouse (8 weeks old) by a single injection of DOX (15 mg/kg, intraperitoneally, MCE HY-15142). Cardiac function was assessed 7 days post-injection, after which tissue was collected for subsequent experiments. For the chronic DIC model, mouse was administered with DOX (5 mg/kg, intraperitoneally) weekly for three consecutive weeks. Cardiac evaluation and tissue collection were performed 6 weeks after the initial injection. For the rescue or inhibitor experiments, 3-methyladenine (MCE HY-19312, 15 mg/kg), rapamycin (MCE HY-10219, 5 mg/kg), recombinant IL12B protein (MCE HY-P700100AF, 3 μg/kg), anti-IL12B neutralizing antibody (Proteintech Group 69006-1-Ig, 5 μg/kg), CCR2 antagonist4 (MCE HY-108323, 10 mg/kg), recombinant Cxcl12 protein (MCE HY-P700219AF, 3 μg/kg), anti-Ifnar2 neutralizing antibody (Thermo Fisher 213851, 5 μg/kg), Cxcr2 inhibitor SB225002 (MCE HY-16711, 2 mg/kg), and CCR1 inhibitor BX471 (MCE HY-12080, 4 mg/kg) were injected as indicated. To assess autophagic flux, bafilomycin A1 (MCE HY-100558, 5 mg/kg) was administered to the mouse 1 h before a single DOX injection (15 mg/kg). The mouse was euthanized and sacrificed 6 h after DOX injection, and the LC3B level was detected by western blot.

### Bone marrow transplantation

Recipient WT or *CCR2^-/-^* mouse (8 weeks old) on a C57BL/6 background was lethally irradiated with a single dose of 6.0 Gy using a Varian Unique irradiator. Within 24 h, they received an intravenous injection of 6 × 10⁶ bone marrow cells from either WT or *CCR2^-/-^* donor mouse via tail vein. Recipient mouse was maintained on antibiotic water for 4 weeks post-transplantation to prevent opportunistic infections. Eight weeks after bone marrow transplantation, we performed direct genomic PCR analysis on bone marrow cells harvested from chimeric mouse to confirm successful reconstitution of recipients with donor bone marrow. WT *CCR2* allele product (551 bp) was amplified with primers: 5'-TGCTCACCAGGAAATGCCAAG-3'; 5'-TGAGCAGGAAGAGCAGGTCAGAG-3'. *CCR2^-/-^* allele product (349 bp) was amplified with primers: 5'-TGCTCACCAGGAAATGCCAAG-3'; 5'-CTCGGGCGGAAAGAACCAGC-3'. The reconstitution efficiency was calculated by densitometric analysis of the WT and knockout fragments. The successful reconstitution chimeric mouse was then subjected to the established acute DIC model for functional and molecular analyses.

### Quantitative real-time PCR

Ventricular tissue samples from mouse or H9C2 cells were lysed in TRIzol reagent (Invitrogen), and total RNA was isolated. One microgram of total RNA was reverse transcribed using a TaKaRa kit. Quantitative real-time PCR was carried out in 20 μL reactions consisting of SYBR Green Master Mix (TaKaRa) and gene specific primers. The sequences of PCR primers used were list on Table S1.

### Western blot

Mouse ventricular tissues or H9C2 cell samples were homogenized in RIPA lysis buffer containing protease inhibitor and phosphatase inhibitor (Roche). The lysates were centrifuged at 12,000 × g for 10 min, and the supernatant protein concentration was measured with a BCA kit (Pierce). Equal amounts of protein were mixed with loading buffer, resolved by SDS-PAGE, and transferred onto PVDF membranes (Bio-Rad). After blocking with non-fat milk, membranes were incubated with primary antibodies **(Table S2)** for 12 h at 4 ℃, and then incubated with HRP-conjugated secondary antibodies for 1 h at 25 °C. Chemiluminescent signals were detected using an ECL substrate (Pierce) and analyzed using Image Lab software (version 5.2.1 Bio-Rad). All western blot experiments were performed with at least three biological replicates.

### Serum biochemical detection

Cardiac injury markers in serum were measured using commercial kits (Nanjing Jiancheng, Cat No: lactate dehydrogenase, A020-2-2; creatine kinase isoenzymes, H197-1-2; cardiac troponin I, H149-2-2) following the manufacturer's protocols.

### Flow cytometry analysis

Cardiac CCR2^+^ cell populations were quantified using a BD FACSymphony™ A1 flow cytometer following an established protocol with modifications 24. In brief, mouse hearts were excised, minced, and digested in an enzyme buffer (450 U/mL collagenase I, 125 U/mL collagenase XI, 60 U/mL DNase I, and 60 U/mL hyaluronidase) (Sigma‒Aldrich) for 1 h at 37 °C. The digested tissues were triturated, filtered through a 40 μm nylon mesh (BD Falcon), washed, and centrifuged at 300 × g for 5 min at 4 °C. Afterward, the cells were resuspended in PBS, and viable cells were counted. A total of 5 × 10^5^ cells in 100 µL of suspension were used for flow cytometry analysis. Fc receptors were blocked by preincubating cells with 0.25 µg of TruStain FcX™ PLUS (BioLegend Cat no: 156603) antibody in a 100 µL volume for 10 min on ice. FITC-conjugated anti-mouse CCR2 antibody (BioLegend Cat no: 150607) was added to the cell suspension and incubated on ice for 15 min in the dark. After being washed twice with PBS, the cells were subjected to flow cytometric analysis. Cells were gated by granularity and size, and singlet populations were selected to exclude clumps before counting CCR2⁺ cells.

### Echocardiography

A VEVO-1100 ultrasound machine equipped with a 30-MHz probe (Visual Sonics, Toronto, Canada) was used to evaluate mouse cardiac function. Briefly, mouse was anesthetized with a single injection of 0.5% pentobarbital sodium (50 mg/kg body weight), and parasternal long/short-axis views of the papillary muscle and 2D-guided M-mode images were recorded with an ultrasound system. Data on ejection fraction and fractional shortening were averaged and calculated from at least five consecutive cardiac cycles. Data were acquired by researchers who were blinded to the treatment groups.

### Histological assessments and immunofluorescence staining

Mouse tissue was isolated, fixed in 4% paraformaldehyde, and embedded in paraffin. Transverse sections (5 μm) were cut and stained with Masson's trichrome or wheat germ agglutinin (WGA) to evaluate cardiac fibrosis and myocyte size, respectively. For immunofluorescence staining, heart sections were dehydrated and permeabilized with 0.1% Triton X-100, then blocked with 5% BSA for 1 h and incubated with primary antibodies **(Table S2)** overnight at 4 °C. Then sections were incubated with fluorophore-conjugated secondary antibodies **(Table S2)** for 2 h, and nuclei were counterstained with DAPI.

### Transmission electron microscopy analysis

Ventricular tissue samples (< 1 mm³) were harvested from mouse under various treatment conditions. H9C2 cells were pelleted by centrifugation (300 × g, 5 min) and pre-embedded in 2% low-melting-point agarose. The samples were fixed in 1.25% glutaraldehyde overnight at 4 °C and then washed with 0.1 M sodium cacodylate 3 times for 15 min each. Afterward, the samples were dehydrated with ethanol, penetrated and embedded in resin and acetone. Polymerized samples were sectioned (60–80 nm thickness) using an ultramicrotome. Sections were collected on 150-mesh copper grids coated with formvar film. After staining, the cuprum grids were observed under TEM (Hitachi HT7800), and images were taken.

### LC‒MS/MS

Cardiac macrophages were isolated from hearts of WT and *CCR2^-/-^* mouse following DOX treatment. For proteomic analysis, proteins extracted from three biological replicates were digested and labeled using TMT reagents: WT samples with 126, 127N, and 127C; *CCR2^-/-^* samples with 128N,128C and 129N. The labeled peptides were loaded onto an EASY-nLC 1200 system and then analyzed by a Q Exactive Plus mass spectrometer (Thermo Fisher Scientific). Raw MS/MS data were analyzed with MaxQuant (version 1.6.6.0) using the Andromeda search algorithm against the UniProt mouse proteome database. The FDR of peptide and protein were set at 1%. Complete mass spectrometry and software parameters are provided in **Table S3**. The MS/MS raw data have been deposited to the iProX database (https://www.iprox.cn/) with accession number IPX0012756000.

### Co-immunoprecipitation

Mouse heart tissues were homogenized in IP lysis buffer (1 mM EDTA, 20 mM Tris/HCl, 150 mM NaCl, 1 mM MgCl₂, 5% glycerol, 1% NP40, protease inhibitors, pH 7.5). Lysates containing 1 mg of total protein were pre-cleared with protein A/G agarose beads for 1 h at 4 °C, then incubated with 2 μg of anti-IL12B or anti-IL12RB1 antibody overnight at 4 °C, with normal IgG serving as a negative control. Protein A/G agarose beads were subsequently added for 4 h, followed by five times wash. Immunoprecipitated proteins were eluted by boiling with loading buffer and subsequently analyzed by western blot. The HRP-conjugated secondary antibody (Vazyme Cat No: RA1008-01) used here is a conformation-specific secondary antibody that reacts only with native IgG and does not bind to the denatured and reduced rabbit IgG heavy chain or light chain.

### Primary neonatal mouse cardiomyocyte isolation

Primary neonatal mouse cardiomyocytes (NMCM) were isolated from C57BL/6J neonatal mouse (1 to 3 days old). Ventricular tissues were minced and subjected to three 10-min digestion cycles at 37 °C in Hanks' Balanced Salt Solution (Ca²⁺/Mg²⁺ free, 0.05% trypsin-EDTA and 0.1% collagenase type II contained). Dissociated cells were passed through a 70 μm strainer, and pre-plated for 90 min to remove fibroblasts. The non-adherent cardiomyocyte enriched fraction was collected and seeded onto gelatin-coated plates. After 48 h of culture, cells were harvested for subsequent experiments.

### Bone marrow-derived macrophage differentiation

Bone marrow-derived macrophages (BMDMs) were obtained from femurs and tibias of WT and *CCR2^-/-^* mouse (8 weeks old). Bone marrow cavities were flushed with DMEM containing 10% FBS. The harvested marrow cells were passed through a 70 μm strainer, pelleted by centrifugation, and treated with red blood cell lysis buffer. Cells were then plated in DMEM supplemented with 10% FBS and 10 ng/mL M-CSF (MCE, Cat No: HY-P7085). Fresh medium containing M-CSF was added on day 3 and completely replaced on day 5. After 6 days of culture, adherent BMDMs were harvested for further experiments.

### ELISA and indirect ELISA

Serum and cardiac tissue cytokines levels were measured with commercial ELISA kits (IL12A, ABclonal RK00014; IL12B, ABclonal RK00017; IL6, Elabscience E-EL-M0044; IL1β, Elabscience E-EL-M0037; MCP1, Elabscience E-EL-M3001) according to the manufacturer’s protocols. For direct ELISA, 50 μL of serum or cardiac tissue homogenate was added to antibody precoated plates, followed by 50 μL of detection antibody cocktail. Then the plates were incubated at 25 °C for 2 h, and washed three times. Subsequently, 100 μL of tetramethylbenzidine (TMB) substrate was added to each well. After 10 min incubation in dark, the enzymatic reaction was halted, and the optical density was recorded at 450 nm. Protein concentrations were calculated on the basis of standard curves.

To measure antibody titers in mouse serum by indirect ELISA, 96-well plates were coated with 1 μg/mL recombinant IL12A or IL12B protein (MCE, HY-P700099AF and HY-P700100AF). After blocking with 5% BSA, serially diluted serum samples were added and incubated for 2 h at 25 °C. HRP-conjugated goat anti-mouse IgG (1:5000 dilution) served as secondary antibody, and color development was carried out using TMB substrate as described above. The absorbance values at 450 nm were normalized to those of the blank controls.

### Cell viability assay

Cell viability was measured with the Cell Counting Kit-8 (Beyotime Biotechnology C0037). Cells were seeded into 96-well plates, treated with various conditions, and then incubated with CCK-8 solution for 1 h. Absorbance at 450 nm was recorded. All experiments were conduct with three biological replicates.

### Autophagic flux measurement

WT and *CCR2^-/-^* mouse were injected with AAV9 encoding mRFP-GFP-LC3 probe, two weeks later, the mouse received a single injection of DOX (15 mg/kg). Seven days after DOX injection, mouse heart was sectioned transversely to 5 μm thick for visualization of the fluorescence. For *in vitro* experiments, H9C2 cells were infected with an adenovirus expressing the mRFP-GFP-LC3 probe for 24 h. The infected cells were then seeded into confocal dishes and exposed to the indicated treatments. Images were acquired with a confocal microscope (Nikon AX). In the merged images, yellow puncta represent autophagosomes, whereas mRFP-only puncta correspond to autolysosomes.

### Tumor studies

Female BALB/c mouse was injected with 1 × 10⁵ 4T1 breast cancer cells into the right inferior mammary fat pad. One week post injection, mouse bearing tumor larger than 50 mm³ were enrolled in the study. For CCR2 antagonist4 experiments, mice were randomly divided into four groups: vehicle control, DOX alone (4 mg/kg on day 7, 14, and 21), CCR2 antagonist4 alone (10 mg/kg, twice per week), and CCR2 antagonist4 + DOX. For IL12B experiments, another cohort of mice were randomized into four groups: vehicle control, DOX alone (4 mg/kg on day 7, 14, and 21), IL12B alone (3 μg/kg, twice weekly), and IL12B + DOX. Tumor dimensions were monitored weekly for 5 weeks. At the end of the 5-week period, cardiac function was assessed, hearts and tumors were collected for subsequent analyses.

### Statistical analysis

Statistical analysis was performed using GraphPad Prism software (version 8.0.2). For longitudinal data with repeated measurements (e.g., tumor volume, body weight), two-way repeated-measures ANOVA followed by Tukey's multiple comparisons test was used. For two groups comparisons, unpaired two-tailed Student's t-test was used. For comparisons involving three or more groups, one-way ANOVA with Tukey's post-hoc test was performed. All continuous data are presented as mean ± standard deviation. Statistical significance was defined as a *p* value < 0.05.

## Results

### CCR2 is significantly upregulated in DOX-induced cardiomyopathy

**Figure 1:**
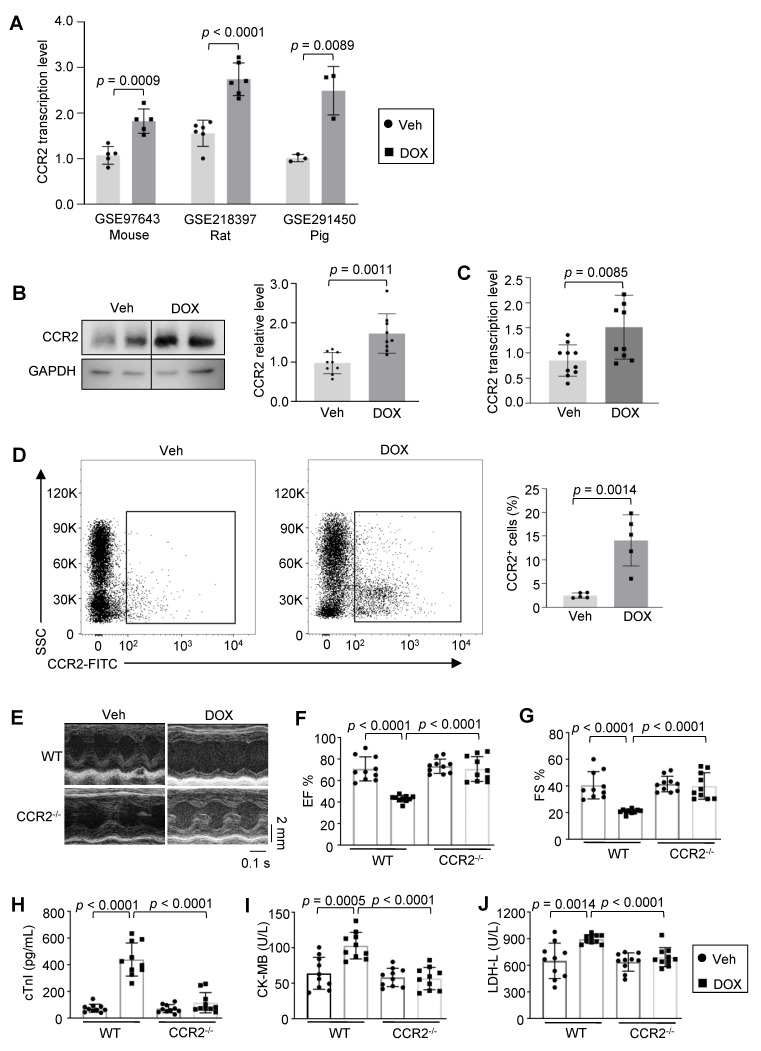
** CCR2 upregulation in DIC and its protective role in acute cardiac injury. (A)** CCR2 mRNA relative levels in mouse, rat, and pig hearts following DOX treatment based on publicly available datasets (GSE97643, n = 5; GSE218397, n = 6; GSE291450, n = 3). **(B)** Western blot results of CCR2 protein in mouse hearts following vehicle or DOX treatment (n = 9). **(C)** Quantitative PCR analysis of CCR2 mRNA levels in mouse hearts after vehicle or DOX administration (n = 10). **(D)** Flow cytometry results of CCR2^+^ cell populations in DOX-exposed hearts compared with control hearts (n = 5). **(E)** Representative M-mode echocardiograms of WT and *CCR2^⁻/⁻^* mouse 7 days post vehicle or DOX injection. Scale bar: 0.1 s, 2 mm.** (F, G)** Quantitative analysis of EF and FS in WT and *CCR2^⁻/⁻^* mouse following vehicle or DOX treatment (n = 10). **(H–J)** The levels of cardiac injury markers in serum, including cTnI, CK-MB, and LDH-L (n = 10).

To investigate the role of CCR2 in DIC, we first assessed CCR2 expression levels under DOX stress using multiple complementary approaches. Analysis of publicly available RNA sequencing datasets (GSE97643, GSE218397, GSE291450) 25, 26 revealed that CCR2 mRNA level was significantly upregulated in heart tissues from multiple species, including mouse, rat, and pig, following DOX treatment (**Figure 1A**). To validate this finding experimentally, we examined CCR2 level at the protein, transcriptional, and cellular levels in our mouse DIC model. Consistent with the bioinformatics results, CCR2 expression was significantly upregulated in DOX-treated hearts across all the following measures: protein abundance (**Figure 1B**), mRNA level (**Figure 1C**), and CCR2^+^ cell population (**Figure 1D**).

### CCR2 deficiency protects against DOX-induced cardiomyopathy in both acute and chronic models

To investigate the therapeutic potential of targeting the CCL2–CCR2 axis in DIC, we first evaluated CCR2 function in an acute DIC model. Echocardiographic analysis revealed that compared with WT control mouse, CCR2 knockout mouse exhibited significant protection against DOX-induced cardiac dysfunction, as evidenced by preserved EF and FS (**Figure 1E-G**). Consistent with these functional improvements, CCR2 deficiency attenuated the DOX-induced increase in serum levels of cardiac injury markers, including cardiac cTnI, CK-MB, and LDH-L (**Figure 1H-J**).

Given that DOX cardiotoxicity is dose dependent, we further evaluated CCR2 function in a clinically relevant chronic low-dose model (5 mg/kg weekly for 3 weeks, **Figure S1A**). While both WT and *CCR2^-/-^* mouse showed comparable ~10% reductions in body weight (**Figure S1B**), *CCR2^-/-^* mouse was protected from the DOX-induced decrease in the heart weight to tibia length (HW/TL) ratio (**Figure S1C**). Echocardiography confirmed preserved cardiac function in *CCR2^-/-^* mouse, with higher EF and FS values than in WT controls (**Figure S1D, S1G-H**), accompanied by reduction levels of cardiac injury markers (**Figure S1I-K**). Histological analysis further demonstrated that *CCR2^-/-^* mouse maintained a normal cardiomyocyte size and reduced fibrosis following DOX treatment (**Figure S1E-F, S1L-M**).

### Hematopoietic CCR2 is necessary and sufficient for DOX-induced cardiomyopathy

**Figure 2:**
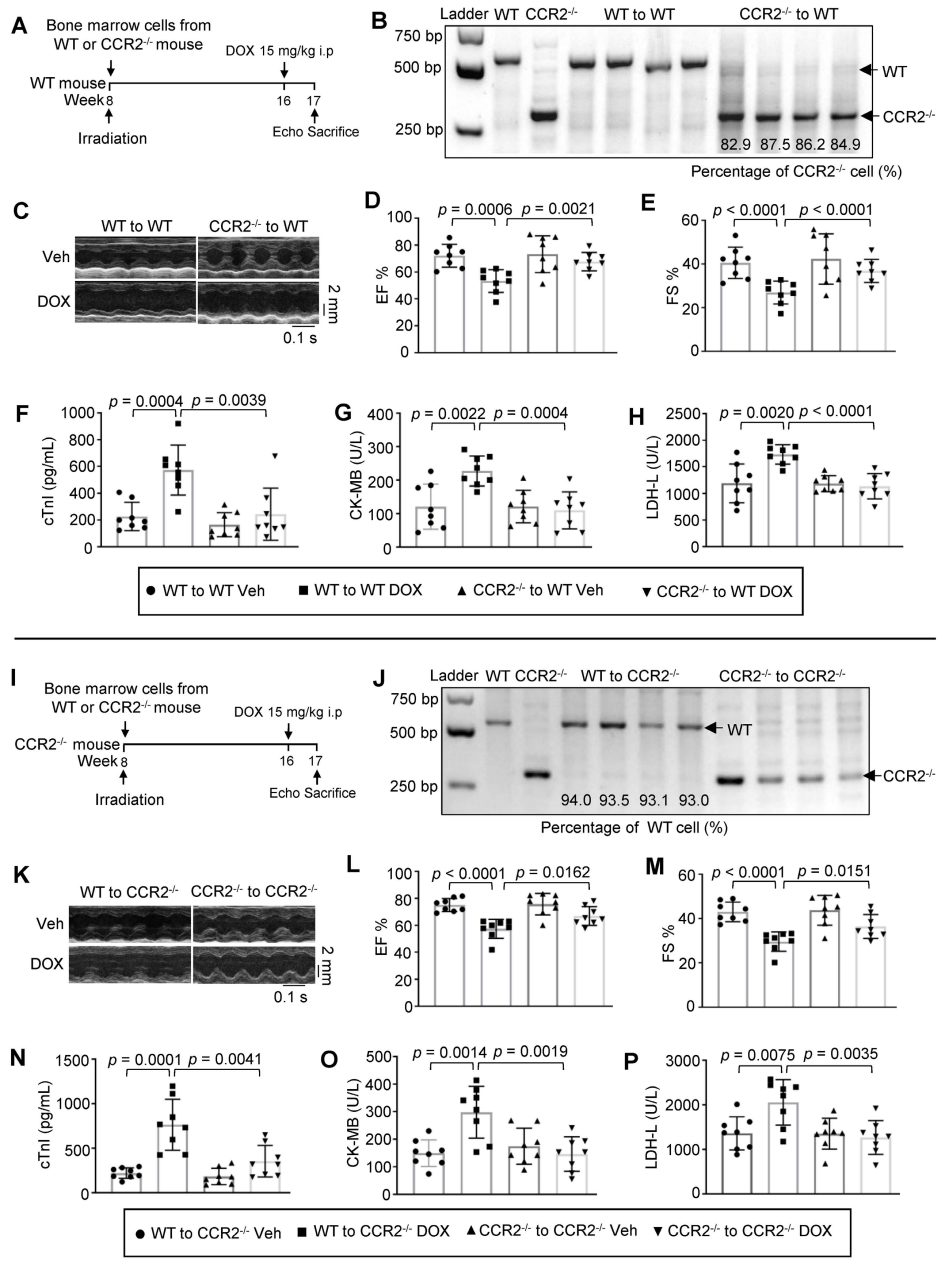
** Hematopoietic CCR2 is necessary and sufficient for DIC. (A)** Schematic illustration of the BMT strategy: lethally irradiated WT recipients were reconstituted with either WT or *CCR2^-/-^* donor bone marrow. After an 8-week period for bone marrow reconstitution, mouse was subjected to the acute DIC model. Cardiac function was then evaluated, and tissues were collected. **(B)** Representative PCR genotyping of bone marrow cells from chimeric mouse confirming successful reconstitution. **(C)** Representative M-mode echocardiographic tracings. Scale bar: 2 mm, 0.1 s. **(D–E)** Quantitative analysis of EF and FS in irradiated WT mouse reconstituted with WT or *CCR2^-/-^* bone marrow treated with vehicle or DOX (n = 8).** (F–H)** The levels of cardiac injury markers in serum, including cTnI, CK-MB, and LDH-L (n = 8). **(I)** Strategy of reciprocal BMT experiment: *CCR2^-/-^* recipients were reconstituted with either WT or *CCR2^-/-^* donor bone marrow. **(J)** Representative PCR genotyping confirming successful reconstitution. **(K)** Representative M-mode echocardiographic tracings. Scale bar: 2 mm, 0.1 s. **(L-M)** Quantitative analysis of EF and FS in irradiated *CCR2^-/-^* mouse reconstituted with WT or *CCR2^-/-^* bone marrow treated with vehicle or DOX (n = 8). **(N–P)** The levels of cardiac injury markers in serum, including cTnI, CK-MB, and LDH-L (n = 8).

To elucidate the hematopoietic origin of the cardioprotective phenotype observed in global *CCR2^-/-^* mouse, we employed bone marrow transplantation (BMT) to generate hematopoietic chimeras. Lethally irradiated WT recipients were reconstituted with either WT or *CCR2^-/-^* donor marrow to dissect the contribution of CCR2 deficiency within the hematopoietic compartment (**Figure 2A**). Genotype PCR analysis confirmed successful reconstitution: WT recipients reconstituted with *CCR2^-/-^* bone marrow exhibited > 80% *CCR2^-/-^* cells in their bone marrow, while recipients of WT bone marrow showed exclusively the WT allele (**Figure 2B**). Functional assessment revealed that irradiated WT mouse reconstituted with *CCR2^-/-^* bone marrow exhibited significant protection against DOX-induced cardiac dysfunction compared with those receiving WT bone marrow, as demonstrated by preserved EF and FS (**Figure 2C-E**). Consistent with these functional improvements, mouse received *CCR2^-/-^* bone marrow attenuated the DOX-induced increase in serum levels of cardiac injury markers, including cTnI, CK-MB, and LDH-L (**Figure 2F-H**).

To further determine whether CCR2 expression on non-hematopoietic cells (e.g., cardiac resident cells) contributes to the phenotype, we performed a reciprocal BMT control in which *CCR2^⁻/⁻^* recipients were reconstituted with WT or *CCR2^-/-^* bone marrow (**Figure 2I**). *CCR2^⁻/⁻^* recipients reconstituted with WT bone marrow (reconstitution efficiency > 90%,** Figure 2J**) developed severe cardiac dysfunction following DOX challenge, comparable to that observed in WT mouse received WT bone marrow (**Figure 2K-M**). In contrast, *CCR2^⁻/⁻^* recipients reconstituted with *CCR2^⁻/⁻^* bone marrow were significantly protected, exhibiting preserved EF, FS and reduced cardiac injury markers (**Figure 2K-P**).

Collectively, these reciprocal BMT experiments provide formal genetic evidence that CCR2 expression on hematopoietic cells, not on non-hematopoietic cardiac resident cells, is both necessary and sufficient for the pathogenesis of DIC. Mouse with *CCR2^⁻/⁻^* hematopoietic cells was protected regardless of the recipient’s genotype, whereas mouse with WT hematopoietic cells developed severe cardiac dysfunction even when the recipient heart was globally *CCR2^⁻/⁻^*.

### CCR2 deficiency attenuates DOX-induced cardiomyopathy through enhanced cardiomyocyte autophagy

**Figure 3:**
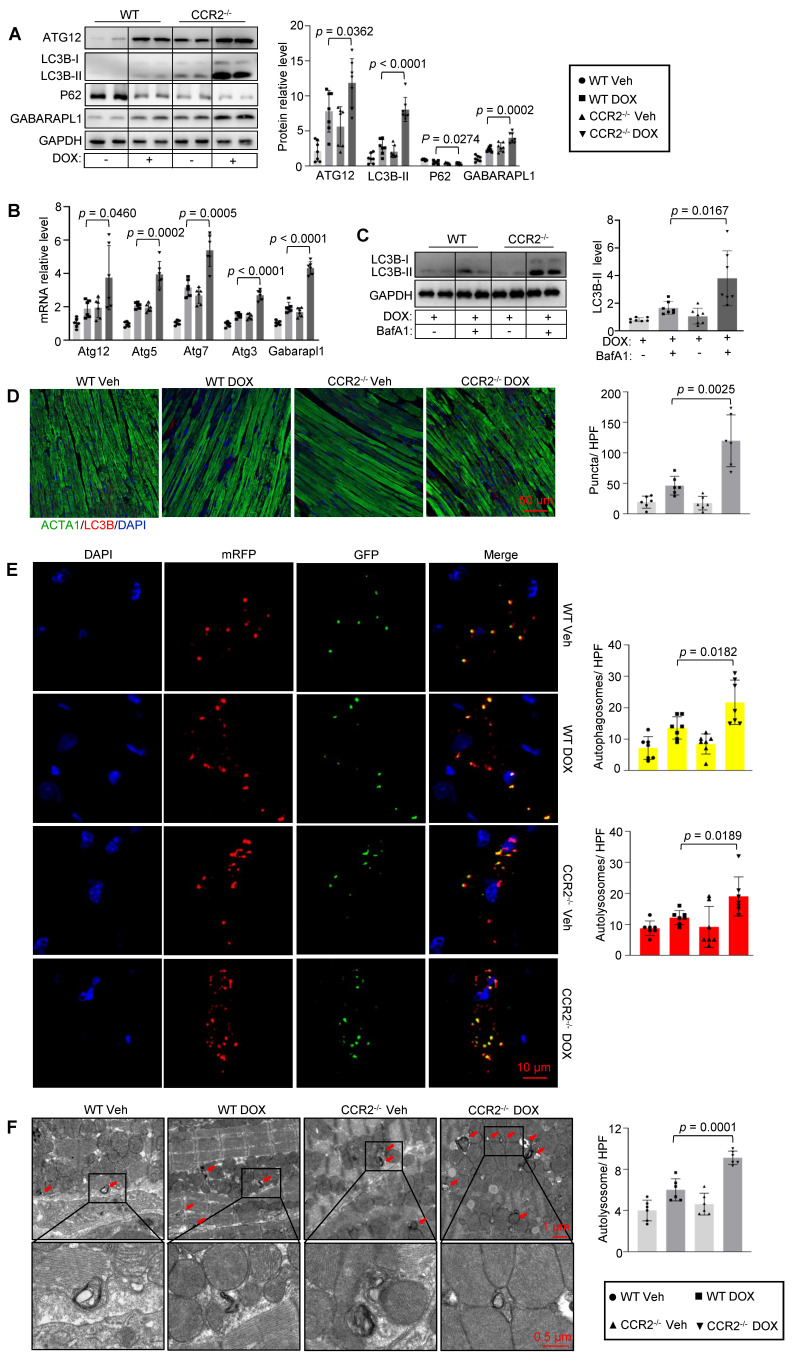
** CCR2 deficiency enhances cardiomyocyte autophagy in DIC. (A)** Western blot results of autophagy markers ATG12, LC3B-II, P62, and GABARAPL1 in cardiac heart from WT and *CCR2^-/-^* mouse following vehicle or DOX treatment (n = 7). **(B)** Quantitative PCR analysis of autophagy genes Atg12, Atg5, Atg7, Atg3, and Gabarapl1 in cardiac heart from the same groups (n = 6)**. (C)** Autophagic flux assessment using bafilomycin A1 pretreatment confirms increased autophagic flux in* CCR2^-/-^* hearts (n = 7). **(D)** Immunofluorescence staining and quantitative analysis of LC3B puncta in cardiac sections. Scale bar: 50 μm. (n = 6). **(E)** *In vivo* autophagic flux monitoring using an AAV9-delivered mRFP-GFP-LC3 reporter. Representative confocal images and quantification of autophagosomes (yellow puncta) and autolysosomes (red puncta). Scale bar: 10 μm. (n = 7). **(F)** Ultrastructural analysis by transmission electron microscopy. The arrows indicate characteristic autolysosomes containing electron dense material. Scale bar: 1 μm, 0.5 μm. (n = 6).

Growing evidence shows that impaired autophagy contributes to DIC pathogenesis. Here, we investigated whether CCR2 is involved in the modulation of autophagy in DIC. Western blot analysis showed significant increased levels of the autophagy markers LC3B-II, ATG12 and GABARAPL1, accompanied by reduced P62 accumulation in *CCR2^-/-^* hearts compared with WT when treated with DOX, suggesting enhanced autophagic flux (**Figure 3A**). Quantitative real-time PCR results further demonstrated that the transcripts of autophagy-related genes Atg12, Atg5, Atg7, Atg3, and Gabarapl1 were increased in *CCR2^-/-^* hearts compared with WT after DOX treatment (**Figure 3B**). These observations were further corroborated through multiple complementary approaches. Bafilomycin A1 treatment led to more LC3B-II accumulation in *CCR2^-/-^* hearts, confirming increased autophagosome formation rather than impaired degradation (**Figure 3C**). Immunofluorescence microscopy revealed a striking 2.6-fold increase in LC3B puncta formation in *CCR2^-/-^* cardiomyocytes (**Figure 3D**), whereas AAV9-mediated expression of the mRFP-GFP-LC3 reporter resulted in concurrent increases in both the number of autophagosomes and autolysosomes (**Figure 3E**). Ultrastructural analysis by transmission electron microscopy provided direct visual evidence of enhanced autophagy, with a 1.5-fold increase in autolysosome density in *CCR2^-/-^* cardiomyocytes (**Figure 3F**).

**Figure 4:**
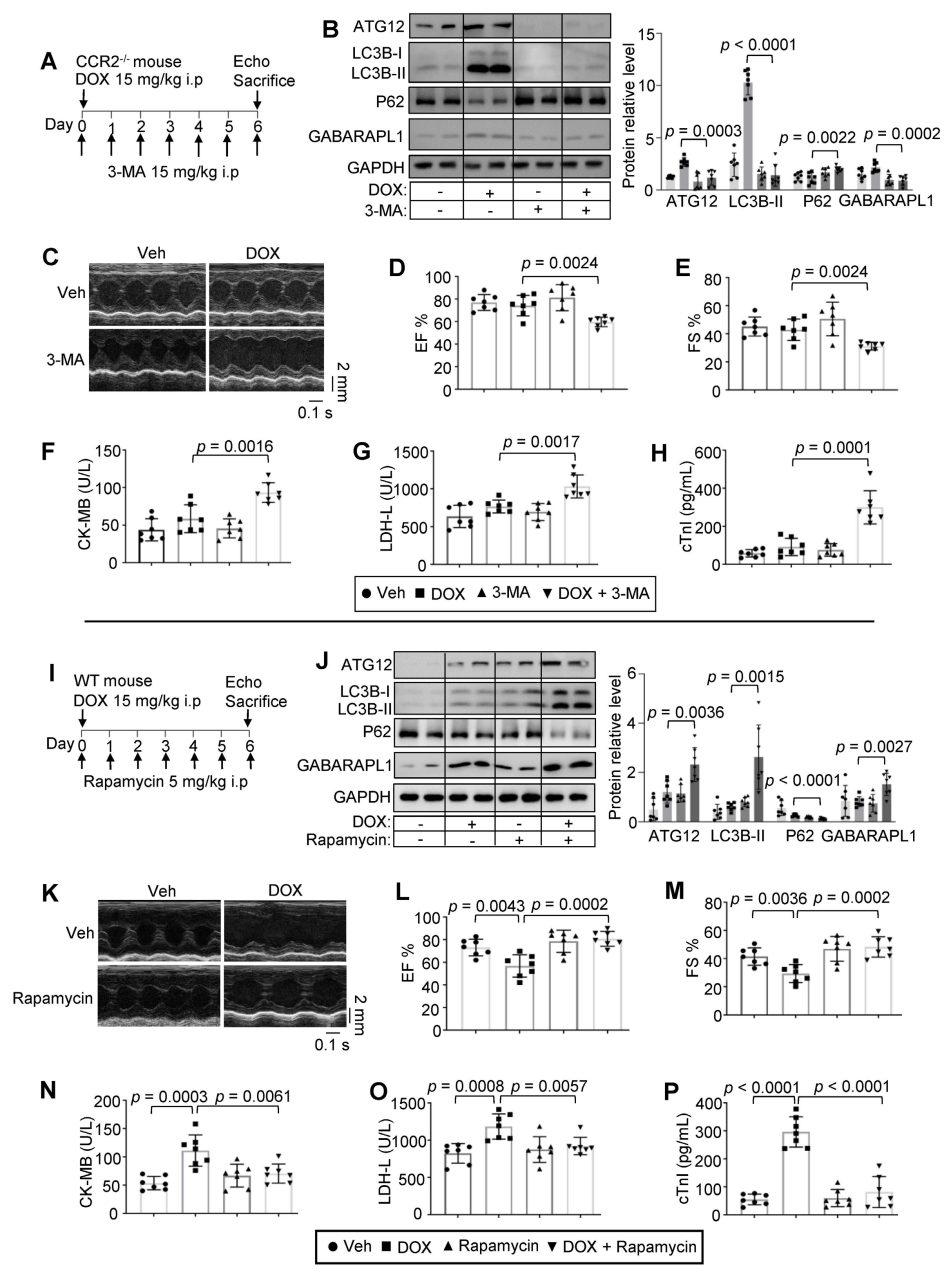
** Autophagy dependency of CCR2-mediated cardioprotection in DIC. (A)** Experimental design for autophagy inhibition studies. *CCR2^-/-^* mouse received daily injections of autophagy inhibitor 3-MA (15 mg/kg, i.p) and a single dose of DOX (15 mg/kg, i.p), and cardiac assessment performed after 7 days.** (B)** Western blot results demonstrating that 3-MA treatment effectively suppressed autophagy in *CCR2^-/-^* hearts (n = 7).** (C–E)** Representative echocardiographic images (Scale bar: 2 mm, 0.1 s) and quantitative results of EF and FS in *CCR2^-/-^* mouse treated with vehicle, DOX, 3-MA and DOX + 3-MA (n = 7).** (F–H)** The levels of cardiac injury markers in serum, including cTnI, CK-MB, and LDH-L (n = 7).** (I)** Experimental timeline for autophagy activation studies.** (J)** Western blot verification of successful autophagy induction by rapamycin in WT hearts (n = 7).** (K–M)** Representative echocardiographic images (Scale bar: 2 mm, 0.1 s) and quantitative results of EF and FS (n = 7) in WT mouse treated with vehicle, DOX, rapamycin and DOX + rapamycin (n = 7).** (N‒P)** The levels of cardiac injury markers in serum, including cTnI, CK-MB, and LDH-L (n = 7).

The functional significance of autophagy enhancement in *CCR2^-/-^*-mediated cardioprotection was established through rigorous pharmacological interventions (**Figure 4A, 4I**). The inhibition of autophagy with 3-methyladenine (3-MA) completely abolished the protective effects of CCR2 deficiency, as evidenced by significant reduction in the expression of autophagic markers (**Figure 4B**) and the complete reversal of the improvements in cardiac function. 3-MA treated* CCR2^-/-^* mouse exhibited reduced EF and FS along with increase in serum levels of cardiac injury markers (**Figure 4C-H**). Conversely, the pharmacological activation of autophagy with rapamycin in WT mouse successfully recapitulated the *CCR2^-/-^* phenotype, with similar improvements in autophagic flux and cardiac function parameters (**Figure 4J-P**). These complementary loss-of-function and gain-of-function experiments provide compelling evidence that enhanced autophagy is a critical mechanism underlying *CCR2^-/-^*-mediated protection against DIC.

### CCR2 deficiency promotes cardiomyocyte autophagy through secretion of IL12B

Given that CCR2 is predominantly expressed on immune cells rather than cardiomyocytes, we sought to identify the mediator responsible for enhanced autophagy in CCR2-deficient hearts. We first performed proteomic profiling of cardiac macrophages isolated from DOX-treated WT and *CCR2^-/-^* mouse. A total of 5,889 proteins were quantified, among which 424 were significantly upregulated and 519 were downregulated (fold change > 1.2,* p*.adjust < 0.01) (**Table S3**), KEGG pathway analysis of differentially expressed proteins revealed significant enrichment of the “cytokine-cytokine receptor interaction” pathway (**Figure S2A, Table S4**). We first validated proteins expression in this pathway by qPCR. Among these, 7 factors showed expression changes consistent with the proteomic data (highlighted in red, **Figure S2B**). For these 7 validated factors, we searched for commercially available intervention factors based on their direction of change in *CCR2^⁻/⁻^* mouse, successfully obtaining Cxcl12 recombinant protein, Ifnar2 neutralizing antibody, Cxcr2 inhibitor SB225002, IL12B neutralizing antibody, and CCR1 inhibitor BX471.

We then designed eight experimental groups (**Figure S2C**) to test whether each candidate factor mediates the protective effect of CCR2 blockade. Due to insufficient breeding of *CCR2^⁻/⁻^* mouse for a study of this scale, we used CCR2 inhibitor (CCR2 antagonist4) to pharmacologically mimic CCR2 deficiency. We first validated that CCR2 antagonist4 treatment recapitulated the cardioprotective phenotype of *CCR2^⁻/⁻^* mouse: DOX-induced heart failure (**Figure S2D-F**, group 1 vs. group 2) was significantly reversed by CCR2 antagonist4 treatment (**Figure S2D-F**, group 2 vs. group 3), confirming that the antagonist is a suitable surrogate. The functional screening revealed that only IL12B neutralizing antibody reversed the cardioprotective effect of CCR2 antagonist4 (**Figure S2D**-**F**, group 3 vs. group 7).

**Figure 5:**
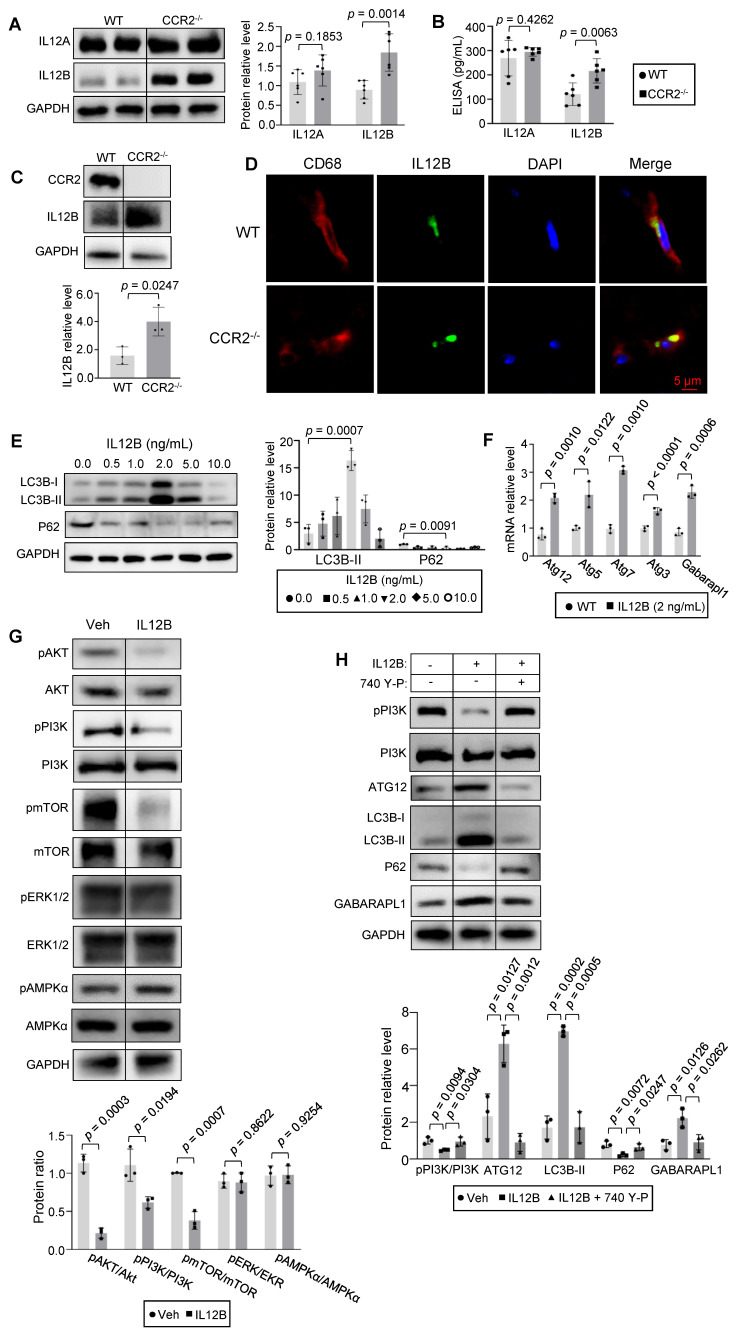
** IL12B is upregulated in CCR2-deficient hearts and activates autophagy through PI3K/Akt/mTOR pathway inhibition. (A)** Western blot results of IL12A and IL12B protein in heart from WT and *CCR2^-/-^* mouse (n = 6). **(B)** ELISA quantification of IL12A and IL12B levels in heart lysates from WT and *CCR2^-/-^* mouse (n = 6). **(C)** Western blot results of IL12B protein in BMDMs differentiated from WT and *CCR2^-/-^* mouse (n = 3 independent experiments). **(D)** Immunofluorescence co-staining of IL12B (green) and the macrophage marker CD68 (red) in heart sections from WT and *CCR2^-/-^* mouse. Scale bar: 5 μm. **(E)** Western blot results of autophagy markers (LC3B-II and P62) in H9C2 cells treated with recombinant IL12B protein (n = 3 independent experiments).** (F)** Quantitative PCR analysis of autophagy-related genes Atg12, Atg5, Atg7, Atg3, and Gabarapl1 in H9C2 cells following vehicle or IL12B treatment (2 ng/mL) (n = 3 independent experiments). **(G)** Western blot results of autophagy-related signaling pathway in H9C2 treated with vehicle or IL12B (2 ng/mL) (n = 3 independent experiments). **(H)** Western blot results of pPI3K, PI3K and autophagy markers (LC3B-II, ATG12, GABARAPL1, and P62) in H9C2 treated with vehicle, IL12B (2 ng/mL) alone, or in combination with the PI3K activator 740 Y-P (3 μM) (n = 3 independent experiments).

We next validated IL12B expression and its cellular source. Western blot and ELISA results confirmed that IL12B, but not IL12A, was specifically upregulated in *CCR2^⁻/⁻^* heart (**Figure 5A-B**). Bone marrow-derived macrophages (BMDMs) differentiated from WT and *CCR2^⁻/⁻^* mouse showed that *CCR2^⁻/⁻^* BMDMs expressed significantly higher levels of IL12B protein (**Figure 5C**). Immunofluorescence co-staining of heart sections revealed clear colocalization of IL12B with the macrophage marker CD68 (**Figure 5D**). We then administered recombinant IL12B protein to investigate whether it directly activates cardiomyocyte autophagy *in vitro*. Results demonstrated that treatment with 2 ng/mL IL12B activated autophagy in H9C2 cells, as indicated by increased LC3B-II level and decreased P62 protein level (**Figure 5E**), and also increase Atg12, Atg5, Atg7, Atg3, and Gabarapl1 transcripts levels (**Figure 5F**). Further examination of autophagy-related signaling showed that IL12B treatment suppressed the activity of the PI3K/Akt/mTOR pathway, a key negative regulator of autophagy initiation 27 (**Figure 5G**). To definitively establish that IL12B activated autophagy through PI3K/Akt/mTOR inhibition, H9C2 cell were treated with recombinant IL12B protein (2 ng/mL) in the presence or absence of PI3K activator 740 Y-P (3 μM). IL12B alone robustly increased LC3B-II, ATG12, and GABARAPL1 while decreased P62 accumulation, and these changes were completely abolished by 740 Y-P co-treatment (**Figure 5H**). This reversal provided functional evidence that IL12B activated H9C2 autophagy through PI3K/Akt/mTOR inhibition.

Finally, we sought to identify the receptor for IL12B on cardiomyocytes. We first examined expression of IL12RB1 and IL12RB2 (two known receptors for IL12B containing cytokines 28) in mouse heart tissue, primary neonatal mouse cardiomyocytes (NMCM), and H9C2 cells by western blot. Both IL12RB1 and IL12RB2 were detectable in all three samples, indicating that the molecular machinery for IL12B signaling exists in cardiomyocytes (**Figure S3A**). Using co-immunoprecipitation assays in mouse heart tissue lysates, we found that IL12B specifically co-precipitated with IL12RB1, but not with IL12RB2 (**Figure S3B**). Reciprocal co-IP using an anti-IL12RB1 antibody also pulled down IL12B (**Figure S3C**). These results demonstrate an interaction between IL12B and IL12RB1 in the heart. To determine whether IL12RB1 is functionally required, we knocked down IL12RB1 in H9C2 with shRNA (GCTGCTGCGTTGAGAAGATAT). As previously reported, IL12B stimulation increased LC3B-II expression, decreased P62, and suppressed phosphorylation of Akt, mTOR, and PI3K. While in IL12RB1 knockdown cells, the effects of IL12B on LC3B-II and P62 were significantly attenuated, and the suppression of the PI3K/Akt/mTOR pathway was relieved, confirming that IL12RB1 is the functional receptor for IL12B on cardiomyocytes (**Figure S3D**).

### Recombinant IL12B treatment enhances cardiomyocyte autophagy and ameliorates DIC *in vitro* and *in vivo*

**Figure 6:**
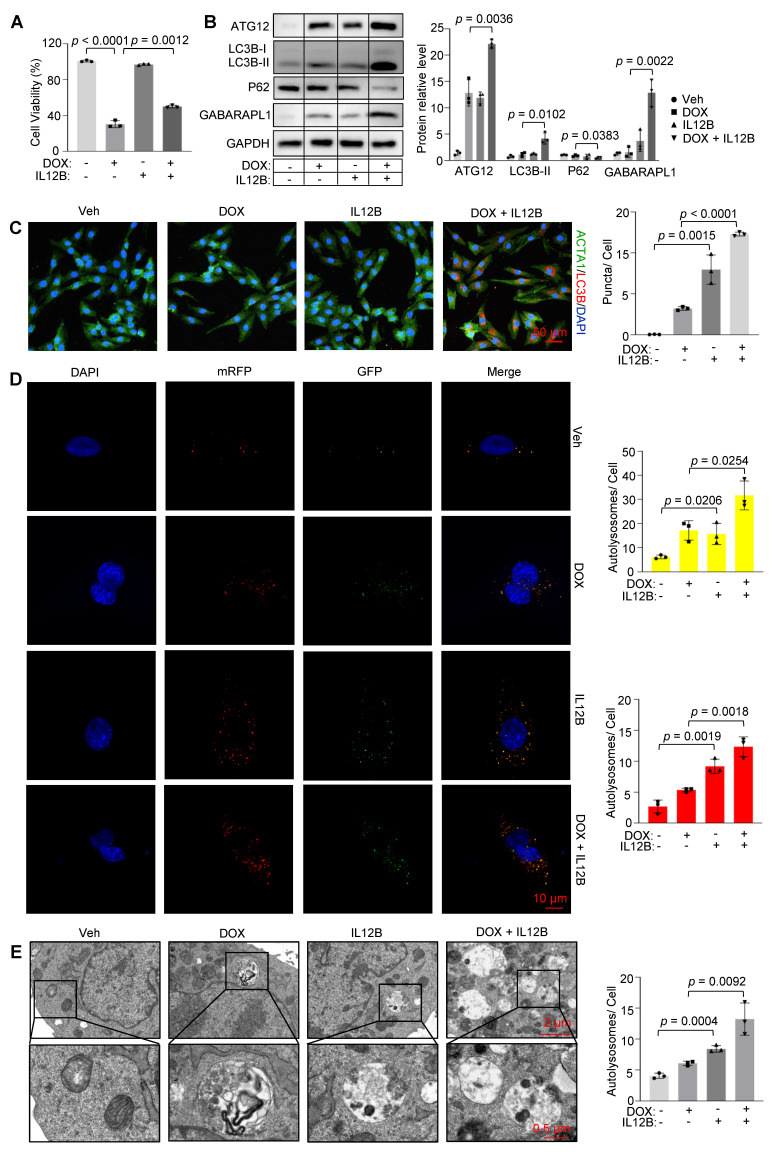
** IL12B promotes cardiomyocyte autophagy *in vitro*. (A)** Cell viability assay of H9C2 treated with DOX (1 μM) in the presence or absence of recombinant IL12B proteins (2 ng/mL) (n = 3 independent experiments).** (B)** Western blot analysis of autophagy markers (LC3B-II, ATG12, GABARAPL1, and P62) in the same groups (n = 3 independent experiments). **(C)** Immunofluorescence staining and quantitative analysis of LC3B puncta in the same groups (n = 3 independent experiments). Scale bar: 20 μm.** (D)** Tandem fluorescence microscopy of H9C2 cells expressing the mRFP-GFP-LC3 reporter. Yellow puncta represent autophagosomes, and red puncta represent autolysosomes. Scale bar: 10 μm (n = 3 independent experiments). **(E)** Transmission electron microscopy of H9C2. Arrows indicate autolysosomes. Scale bar: 2 μm, 0.5 μm (n = 3 independent experiments).

The functional significance of IL12B in mitigating DIC was demonstrated through a series of comprehensive *in vitro* experiments. Treatment with recombinant IL12B significantly increased cardiomyocyte resilience and autophagic activity, as evidenced by (1) increased cell viability under DOX-induced stress (**Figure 6A**); (2) upregulation of the autophagy markers LC3B-II, ATG12, and GABARAPL1, accompanied by a reduction in P62 level (**Figure 6B**); (3) increased formation of LC3B puncta (**Figure 6C**); and (4) improved autophagic flux, as confirmed by both tandem fluorescence microscopy (**Figure 6D**) and transmission electron microscopy (**Figure 6E**). These findings collectively indicate that IL12B not only promotes autophagic activity but also provides functional protection against DOX-induced cardiomyocyte damage *in vitro*.

Building upon our *in vitro* findings, we next investigated whether IL12B protein administration could confer cardioprotection *in vivo*. As IL12B is a pro-inflammatory cytokine subunit, we first conduct safety assessment. Mouse were randomly assigned to receive either vehicle or recombinant IL12B protein (3 μg/kg, i.v.) daily for 7 consecutive days, and sacrificed at three time points: day 0 (2 h post-injection), day 3, and day 6. Serum was collected for cytokine analysis, and major organs including heart, liver, lungs, spleen, and kidneys were collected for histopathological evaluation. Cytokine analysis revealed that IL12B treated mouse exhibited a modest but significant elevation of inflammatory cytokines, including IL-6, IL-1β, and MCP-1 on day 3. However, this increase was transient and self-resolving, with cytokine levels returning to baseline by day 6 (**Figure S4A-C**). Histopathological examination of major organs at day 6 revealed no detectable abnormalities, with no evidence of tissue necrosis, edema, or structural damage in any organ examined (**Figure S4D**). ELISA analysis confirmed a specific increase in circulating IL12B, but not IL12A, following IL12B administration (**Figure S4E**). These data demonstrate that, while IL12B transiently activates mild systemic inflammation, this response is self-limited and well-tolerated, with no evidence of end-organ damage.

**Figure 7:**
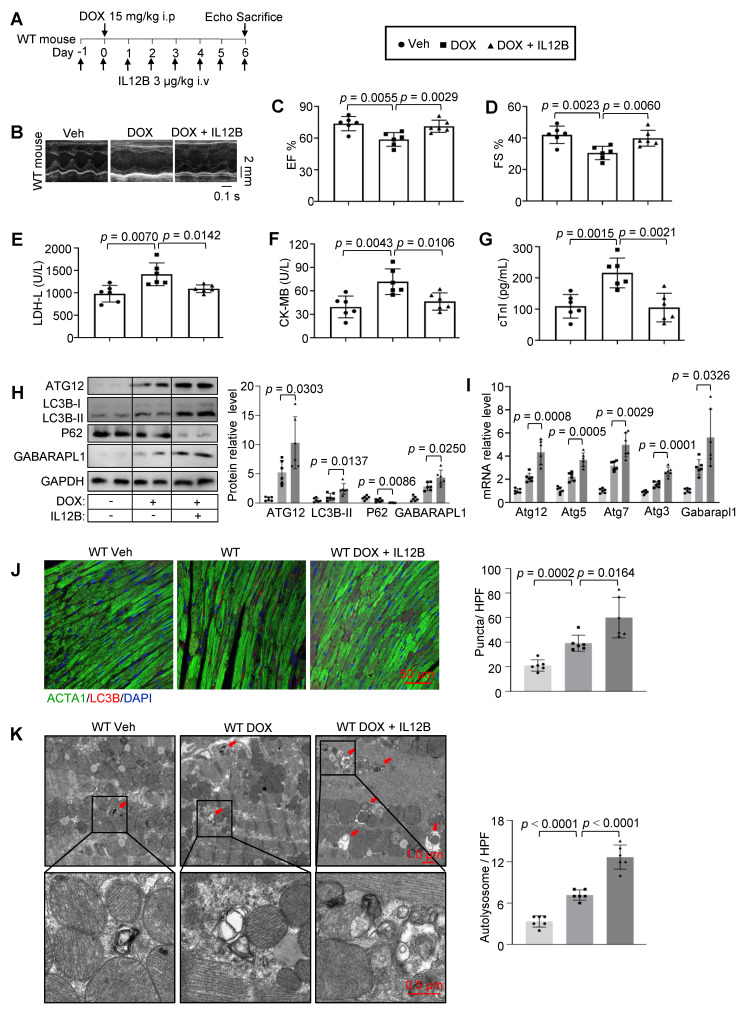
** Recombinant IL12B administration protects against DIC through autophagy activation *in vivo*. (A)** Experimental design for *in vivo* IL12B administration. **(B)** Representative M-mode echocardiographic tracings of mouse from the vehicle, DOX alone, and DOX + IL12B groups. Scale bar: 0.1 s, 2 mm. **(C, D)** Quantitative analysis of EF and FS in the indicated groups (n = 6). **(E–G)** The levels of cardiac injury markers in serum, including LDH-L, CK-MB, and cTnI (n = 6). **(H)** Western blot analysis of autophagy markers (LC3B-II, ATG12, GABARAPL1, and P62) in heart tissues from the same groups (n = 6). **(I)** Quantitative PCR analysis of autophagy-related gene (Atg12, Atg5, Atg7, Atg3, and Gabarapl1) in heart tissues from the same groups (n = 6). **(J)** Immunofluorescence staining and quantitative analysis of LC3B puncta in cardiomyocytes from the same groups. Scale bar: 50 μm (n = 6). **(K)** Transmission electron microscopy analysis of autolysosome density from the same groups. Arrows indicate autolysosomes. Scale bars: 1 μm and 0.5 μm (n = 6).

We then investigated whether IL12B confer cardioprotection *in vivo*. WT mouse received daily IL12B (3 μg/kg) via tail vein injection and an acute DIC model was established (**Figure 7A**). Remarkably, IL12B treatment significantly attenuated DOX-induced cardiac dysfunction, as demonstrated by echocardiographic assessment showing preservation of EF and FS (**Figure 7B-D**). Concurrently, serum levels of cardiac injury markers were markedly reduced in IL12B treated animals (**Figure 7E-G**). Molecular and cellular analyses confirmed that the cardioprotective effects of IL12B were mediated through enhancing autophagy in cardiomyocytes. Western blot analysis revealed significant upregulation of autophagy markers LC3B-II, ATG12 and GABARAPL1, and downregulation of P62 in IL12B treated hearts, compared to hearts treated with DOX only (**Figure 7H**). Quantitative real-time PCR results also demonstrated that the transcripts of Atg12, Atg5, Atg7, Atg3, and Gabarapl1 were increased in IL12B treated hearts (**Figure 7I**). Immunofluorescence microscopy revealed a 1.5-fold increase in LC3B puncta formation in cardiomyocytes from IL12B-treated mouse (**Figure 7J**), whereas transmission electron microscopy revealed a 1.8-fold increase in autolysosome density (**Figure 7K**). These findings provide compelling evidence that IL12B administration activates cardiomyocyte autophagy and confers substantial protection against DIC *in vivo*.

### IL12B mediates the cardioprotective effects of CCR2 deficiency in DIC

**Figure 8:**
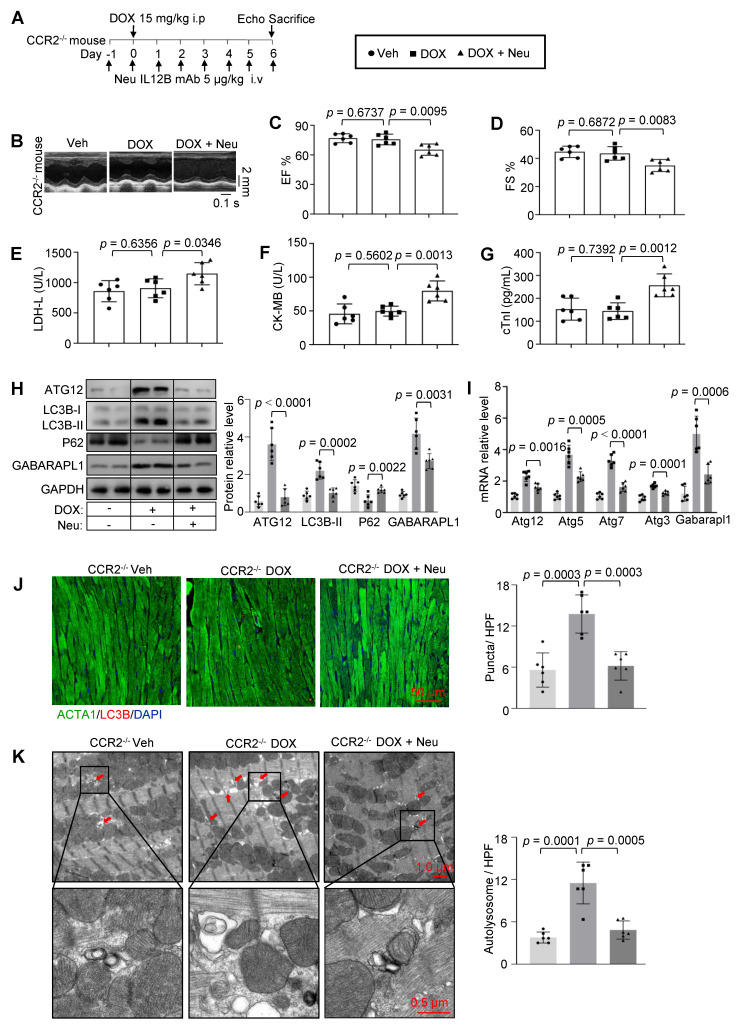
** IL12B neutralization abolishes CCR2 deficiency-mediated cardioprotection *in vivo*. (A)** Experimental design of IL12B neutralization in *CCR2^-/-^* mouse. **(B)** Representative M-mode echocardiographic tracings of *CCR2^-/-^* mouse treated with vehicle, DOX alone, or DOX + anti-IL12B Neu. Scale bar: 0.1 s, 2 mm. **(C, D)** Quantitative results of EF and FS in the indicated groups (n = 6). **(E–G)** The levels of cardiac injury markers in serum, including LDH-L, CK-MB, and cTnI (n = 6). **(H)** Western blot results of autophagy markers (LC3B-II, ATG12, GABARAPL1, and P62) in heart tissues from *CCR2^-/-^* mouse treated with vehicle, DOX alone, or DOX + anti-IL12B Neu (n = 6).** (I)** Quantitative PCR analysis of autophagy-related gene (Atg12, Atg5, Atg7, Atg3, and Gabarapl1) in heart tissues from the same groups (n = 6). **(J)** Immunofluorescence staining and quantitative analysis of LC3B puncta in cardiomyocytes from the same groups. Scale bar: 50 μm (n = 6). **(K)** Transmission electron microscopy analysis of autolysosome density in cardiomyocytes from the same groups. Arrows indicate autolysosomes. Scale bars: 1 μm and 0.5 μm (n = 6).

The critical role of IL12B in mediating CCR2 deficiency protection was definitively established through IL12B neutralization experiments (**Figure 8A**). The administration of anti-IL12B neutralizing antibody (Neu, 5 μg/kg daily) to *CCR2^-/-^* mouse completely abolished the protective effects against DIC, as demonstrated by comprehensive functional and molecular analyses. Echocardiographic assessment revealed significant deterioration of cardiac function in Neu treated *CCR2^-/-^* mouse, with the EF declining from 75.6 ± 4.9% to 65.4 ± 5.2% and FS decreasing from 43.6 ± 4.4% to 35.0 ± 13.9% (**Figure 8B-D**). This functional impairment was accompanied by marked increases in serum levels of cardiac injury markers, including cTnI, CK-MB, and LDH-L (**Figure 8E-G**), indicating complete reversal of the *CCR2^-/-^* protective phenotype. The specificity of the IL12B neutralizing antibody was confirmed by indirect ELISA (**Figure S4F**).

At the molecular level, IL12B neutralization markedly suppressed autophagy activation in *CCR2^-/-^* hearts. This was demonstrated by decreased expression of the autophagy markers LC3B-II, ATG12, and GABARAPL1, along with increased P62 accumulation (**Figure 8H**). Consistently, transcript levels of Atg12, Atg5, Atg7, Atg3, and Gabarapl1 were also reduced following IL12B neutralization (**Figure 8I**). Cellular analyses confirmed these findings, as fewer LC3B puncta were detected by immunofluorescence, and transmission electron microscopy detected a reduction in autolysosome formation in DOX + Neu treated mouse compared to DOX only treated *CCR2^-/-^* mouse (**Figure 8J-K**). These results provide conclusive evidence that IL12B serves as a molecular link between CCR2 deficiency and enhanced cardiomyocyte autophagy, establishing a CCR2-IL12B-autophagy axis for cardioprotection.

### Pharmacological CCR2 inhibition and IL12B supplementation protects against DIC without compromising the antitumor efficacy of doxorubicin

**Figure 9:**
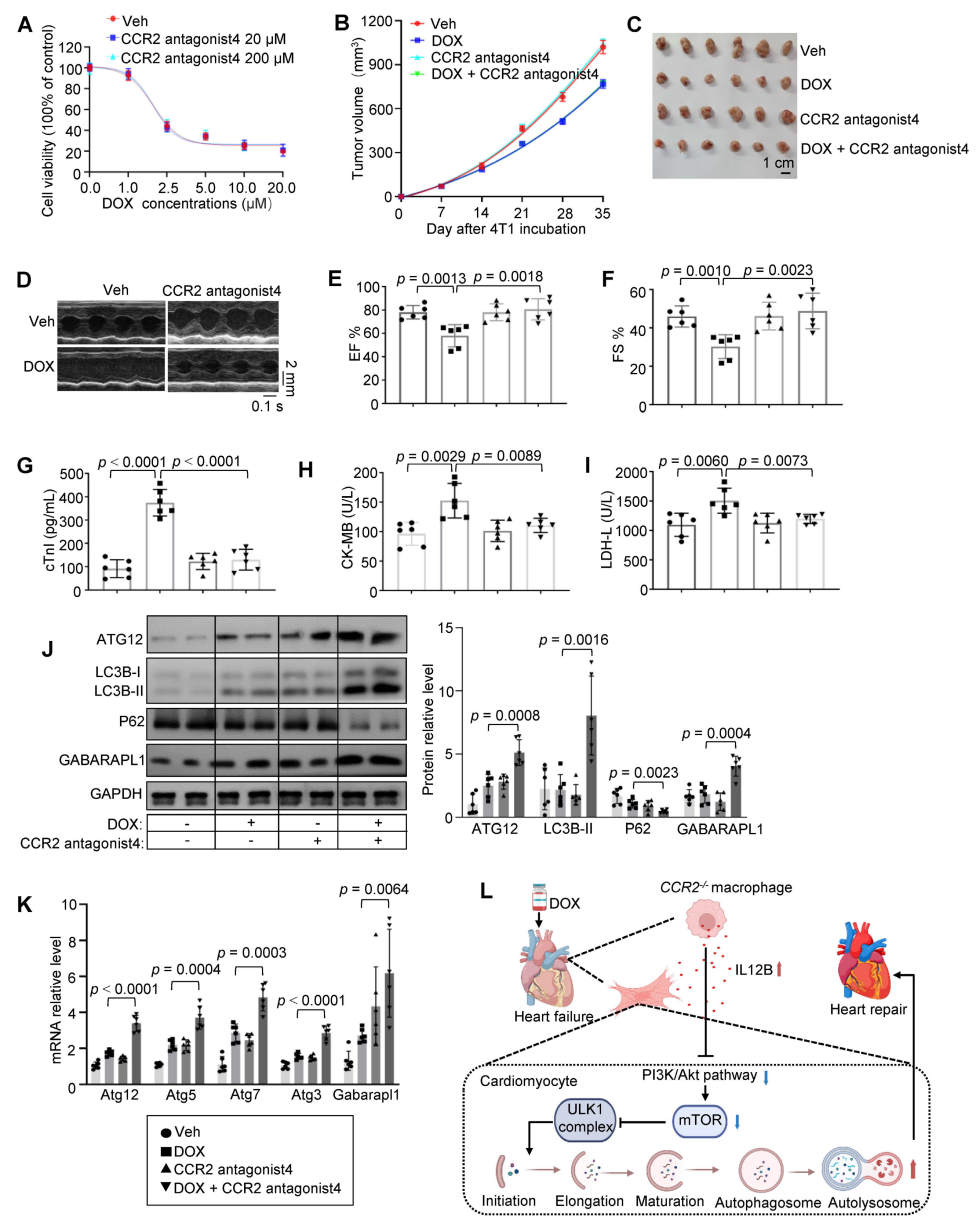
** Pharmacological CCR2 inhibition protects against DIC without compromising the antitumor efficacy of doxorubicin in 4T1 breast cancer model. (A)** Cell viability assay of 4T1 breast cancer cells treated with DOX or in combination with CCR2 antagonist4 (n = 3 independent experiments). **(B, C)** Tumor growth curves and representative tumor images at the study endpoint in a syngeneic 4T1 breast cancer model. Scale bar: 1 cm (n = 6).** (D)** Representative M-mode echocardiographic tracings of tumor bearing mouse treated with vehicle, DOX alone, CCR2 antagonist4 alone or DOX + CCR2 antagonist4. Scale bar: 0.1 s, 2 mm. **(E, F)** Quantitative results of EF and FS in the same groups (n = 6). **(G–I)** The levels of cardiac injury markers in serum, including LDH-L, CK-MB, and cTnI (n = 6). **(J)** Western blot results of autophagy markers (LC3B-II, ATG12, GABARAPL1, and P62) in heart tissues from the same groups (n = 6). **(K)** Quantitative PCR analysis of autophagy-related gene (Atg12, Atg5, Atg7, Atg3, and Gabarapl1) in heart tissues from the same groups (n = 6). **(L)** Working model. DOX administration triggers cardiomyocyte dysfunction, ultimately leading to heart failure. In contrast, loss of function of CCR2 initiates a protective cascade. Specifically, CCR2 deficiency increases the secretion of IL12B. This cytokine directly targets cardiomyocytes, suppressing PI3K/Akt/mTOR signaling to trigger protective autophagy, which clears damaged organelles and toxic proteins, thus sustaining cellular homeostasis and cardiac function.

Given the cardioprotective effects observed in *CCR2^-/-^* mouse, we investigated the therapeutic potential of pharmacological CCR2 inhibition using CCR2 antagonist4. This approach offers significant clinical advantages by potentially preventing DIC while maintaining the crucial antitumor activity of doxorubicin. *In vitro* studies using 4T1 breast cancer cells demonstrated that CCR2 antagonist4 co-treatment did not affect the cytotoxic efficacy of DOX, with comparable reductions in tumor cell viability observed in both the DOX-only and combination treatment groups (**Figure 9A**).

To evaluate this strategy *in vivo*, we established a syngeneic 4T1 breast cancer model in BALB/c mouse. Tumor-bearing mice (tumor volume > 50 mm^3^) were randomized into four treatment groups: vehicle control, DOX alone (5 mg/kg weekly), CCR2 antagonist4 alone (10 mg/kg twice weekly), and combination therapy. Longitudinal monitoring revealed identical tumor growth inhibition in both DOX-treated groups, confirming that CCR2 antagonist4 does not interfere with the chemotherapeutic effects of DOX (**Figure 9B-C**). Furthermore, immunofluorescence analysis of tumor tissue sections revealed a significant reduction in CD11b⁺ myeloid cells and CD68⁺ macrophages within the tumor, indicating that CCR2 blockade effectively reduces myeloid cell recruitment into the tumor microenvironment. Critically, CD8⁺ T cell infiltration remained unchanged compared with DOX-alone controls, demonstrating that CCR2 antagonist4 does not compromise adaptive anti-tumor immunity (**Figure S5**). Importantly, concurrent CCR2 antagonist4 administration provided significant cardioprotection, as evidenced by preserved EF and FS (**Figure 9D-F**) and reduced levels of cardiac injury markers (**Figure 9G-I**). Molecular analysis confirmed that CCR2 antagonist4 mediated cardioprotection through autophagy activation, as indicated by increased LC3B-II, ATG12, and GABARAPL1 expression and a concomitant reduction in P62 expression in cardiac tissue (**Figure 9J**), as well as increased transcripts level of Atg12, Atg5, Atg7, Atg3, and Gabarapl1 (**Figure 9K**).

We next investigated whether IL12B co-treatment affects the cytotoxic efficacy of DOX. IL12B co-treatment did not alter DOX cytotoxicity, as evidenced by comparable reductions in tumor cell viability in both the DOX-only and combination groups (**Figure S6A**). Longitudinal monitoring further revealed identical tumor growth inhibition in both DOX-treated groups (DOX alone vs. DOX + IL12B), confirming that IL12B does not interfere with the chemotherapeutic effects of DOX (**Figure S6B-C**). Moreover, concurrent IL12B administration provided significant cardioprotection, as evidenced by preserved EF, FS (**Figure S6D-F**) and reduced levels of cardiac injury markers (**Figure S6G-I**). Together, these data indicate that IL12B does not compromise the chemotherapeutic efficacy of doxorubicin in this breast cancer model while simultaneously providing cardiac protection.

To extend our findings beyond the 4T1 model, we tested whether CCR2 antagonist4 or IL12B affects doxorubicin cytotoxicity in additional tumor cell lines representing different cancer types: A2780 (human ovarian carcinoma), B16F10 (mouse melanoma), and MC38 (mouse colon carcinoma). In all three cell lines, doxorubicin alone induced dose-dependent cytotoxicity, and co-treatment with either CCR2 antagonist4 or IL12B did not significantly alter killing effect of doxorubicin (**Figure S7A-F**).

## Discussion

On the basis of our findings, we propose a working model delineating the mechanism by which CCR2 deficiency confers protection against doxorubicin-induced cardiotoxicity. DOX administration triggers cardiomyocyte dysfunction and recruit CCR2^+^ monocytes from the bone marrow to injury sites, ultimately leading to progression to heart failure. In contrast, loss of function of CCR2 initiates a protective immunomodulatory-cytokine-autophagy cascade. Specifically, CCR2 deficiency promotes a shift in cardiac macrophages toward a reparative phenotype, resulting in increasing secretion of IL12B. This cytokine acts directly on cardiomyocytes to activate protective autophagy through inhibition of the PI3K/Akt/mTOR pathway. This process promotes the removal of damaged organelles and cytotoxic proteins, thereby maintaining cellular homeostasis and preserving cardiac function (**Figure 9L**).

This model is grounded in the established paradigm wherein resident cardiac macrophages maintain tissue homeostasis, while infiltrating CCR2^+^ monocytes orchestrate detrimental inflammatory cascades that exacerbate cardiac injury 29-31. Although the therapeutic potential of targeting the CCL2-CCR2 axis is well-established in the context of myocardial infarction and heart failure 32, 33, its role in DIC remained less defined. A recent study identified cardiomyocyte GSDME as an upstream activator of CCL2 in DIC and demonstrated that CCL2-CCR2 inhibition is cardioprotective 34. This work further supports the involvement of the CCL2-CCR2 axis in DIC, yet the specific downstream protective mechanisms triggered upon its inhibition within the chemotoxic heart remained undefined. Our study demonstrated that CCR2 inhibition in DIC does not merely attenuate a generic inflammatory response, but engages a distinct, cardioprotective program characterized by a shift in the cardiac macrophage secretome towards IL12B.

At the mechanistic level, we identified autophagy activation as a critical downstream effect of CCR2 inhibition. DIC is characterized by excessive oxidative stress, mitochondrial dysfunction, and impaired autophagic flux 3, 35-37. Our data suggest that CCR2 deficiency promotes IL12B-mediated protective autophagy, which may help clear damaged mitochondria and reduce ROS accumulation, thereby preserving cardiomyocyte viability. However, whether the CCR2-IL12B axis directly interacts with oxidative stress pathways or functions in parallel remains to be determined. Additionally, whether IL12B specifically activates mitophagy versus general autophagy requires future investigation. From a translational perspective, CCR2 antagonists are already being evaluated in clinical trials for inflammatory diseases, raising the possibility of repurposing for cardio-oncology 38, 39. Future studies exploring potential synergy between CCR2 blockade and established cardioprotective agents are warranted.

The functional centrality of autophagy was validated through both loss-of-function experiments, in which autophagy inhibition abolished *CCR2^-/-^* cardioprotection, and gain-of-function studies, in which pharmacological activation of autophagy alone was sufficient to attenuate DIC. Nevertheless, given the off-target effects of 3-MA 40, cardiomyocyte-specific knockout of autophagy genes is warranted to further establish causality. Moreover, it is important to note that the role of autophagy in DIC is complex and context-dependent, exhibiting a well-documented dual nature. While a substantial body of evidence, including our own, supports that timely enhancement of autophagy is protective, other studies using models such as *Beclin1^⁺/⁻^* mouse have reported that partial suppression of autophagy can also improve outcomes 41. This apparent paradox underscores the opposing roles of autophagy in cardiomyocyte fate decisions. Therefore, therapeutic strategies aimed at modulating autophagy must be precisely calibrated.

The discovery of IL12B as the key molecular mediator between CCR2 signaling and autophagy activation represents a significant advance in our understanding of cardiac pathophysiology. While IL12B is best characterized as a subunit of the heterodimeric cytokines IL-12 and IL-23, with well-defined roles in immune regulation and genetic associations with inflammatory diseases 42-44, its function in heart was entirely unexplored. Although prior studies indicated that IL-12 can induce autophagy in immune and cancer cells via pathways such as JAK2 or PI3K/Akt 22, 23, the specific role of the IL12B (p40) subunit remained unclear. Our study expands this paradigm by demonstrating a direct autophagy activating function of IL12B monomer on cardiomyocyte. Experiments with recombinant IL12B protein and its specific neutralization establish that IL12B exerts cardioprotection independently of its partnership with IL12A (p35) or p19. Furthermore, we reposition this cytokine within a novel pathophysiological context: rather than modulating infection or tumor immunity, IL12B here mediates a cardioprotective pathway activation during doxorubicin-induced injury. This work thus reveals a previously unrecognized dimension of IL12B biology, transforming it from a classic immunomodulatory subunit into a mediator of cardio-immune crosstalk and a potential therapeutic agent in cardio-oncology.

Our BMDM and immunofluorescence co-staining experiments provide evidence that cardiac macrophages are a major cellular source of the elevated IL12B in *CCR2^⁻/⁻^* hearts. However, we cannot exclude contributions from other rare immune populations based on these data alone. Additionally, while our study establishes that IL12B directly activates protective autophagy in cardiomyocytes, it remains possible that IL12B also exerts complementary cardioprotective effects by modulating other cell types within the cardiac microenvironment, such as fibroblasts or endothelial cells. Future studies employing cell-type-specific knockout of IL12B or its receptor will be essential to address these questions.

The clinical implications of these findings are substantial. First, they validate CCR2 inhibition as a viable strategy to prevent DIC, with the added benefit of maintaining chemotherapy efficacy. Second, they identify IL12B itself as a potential therapeutic agent. Finally, the elucidation of this CCR2/IL12B/autophagy axis provides multiple potential intervention points for therapeutic development. Together, these results significantly advance our understanding of DIC pathogenesis and provide concrete therapeutic strategies that could substantially improve outcomes for cancer patients receiving anthracycline chemotherapy.

## Supplementary Material

Supplementary figures and tables.

## Data Availability

All data are included within the article or Supplementary material or available from the authors on request.

## Abbreviations

DOX: Doxorubicin
DIC: DOX-induced cardiomyopathy
CCR2: C-C chemokine receptor type 2
IL12B: interleukin-12B
CK-MB: creatine kinase isoenzymes
LDH-L: lactate dehydrogenase
cTnI: cardiac troponin I
3-MA: 3-methyladenine
EF: ejection fraction
FS: fraction Shortening
i.p: intraperitoneal injection
i.v: intravenous injection
