# Supplementary material for: CCR2 deficiency protects against doxorubicin-induced cardiac dysfunction through enhanced IL12B-dependent autophagy
Source: Theranostics. 2026 Jul 22;16(14):8326–46. doi: 10.7150/thno.131005 (PMC13440645; doi:10.7150/thno.131005)
Supplement: Supplementary file 1 [file thnov16p8326s1.pdf]

Supplementary Information

CCR2 deficiency protects against doxorubicin-induced cardiac dysfunction through enhanced IL12B-dependent autophagy

The supplementary file includes 7 supplementary figures and 3 supplementary tables.

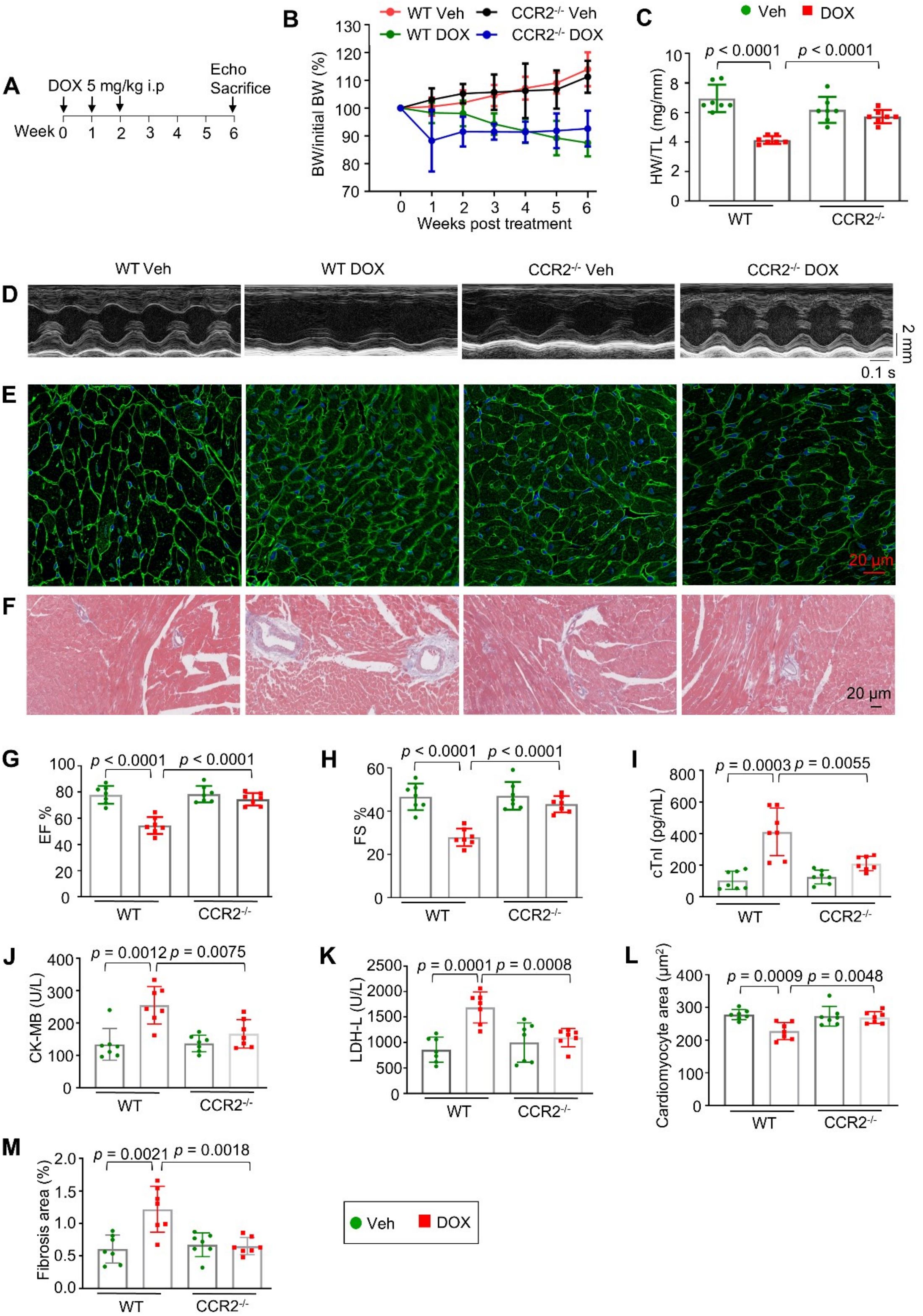

Figure S1 CCR2 deficiency mitigates chronic low-dose doxorubicin-induced cardiotoxicity.

(A) Strategy of the chronic low-dose DOX treatment protocol. WT and *CCR2*$^{-/-}$ mouse received DOX (5 mg/kg weekly) for 3 consecutive weeks. **(B)** Body weight changes

over the course of DOX treatment (n = 7). **(C)** Heart weight to tibia length ratio in WT and *CCR2*$^{-/-}$ mouse following vehicle or chronic DOX treatment (n = 7). **(D)** Representative M-mode echocardiographic tracings of WT and *CCR2*$^{-/-}$ mouse following vehicle or chronic DOX treatment. Scale bar: 0.1 s, 2 mm. **(E, F)** Representative images of WGA staining and Masson's trichrome staining of heart sections from the indicated groups. Scale bars: 20 μm. **(G, H)** Quantitative results of EF and FS in the same groups (n = 7). **(I–K)** The levels of cardiac injury markers in serum, including cTnI, CK-MB, and LDH-L (n = 7). **(L, M)** Quantitative results of cardiomyocyte cross-sectional area and fibrotic area from WGA and Masson's trichrome staining, respectively (n = 7).

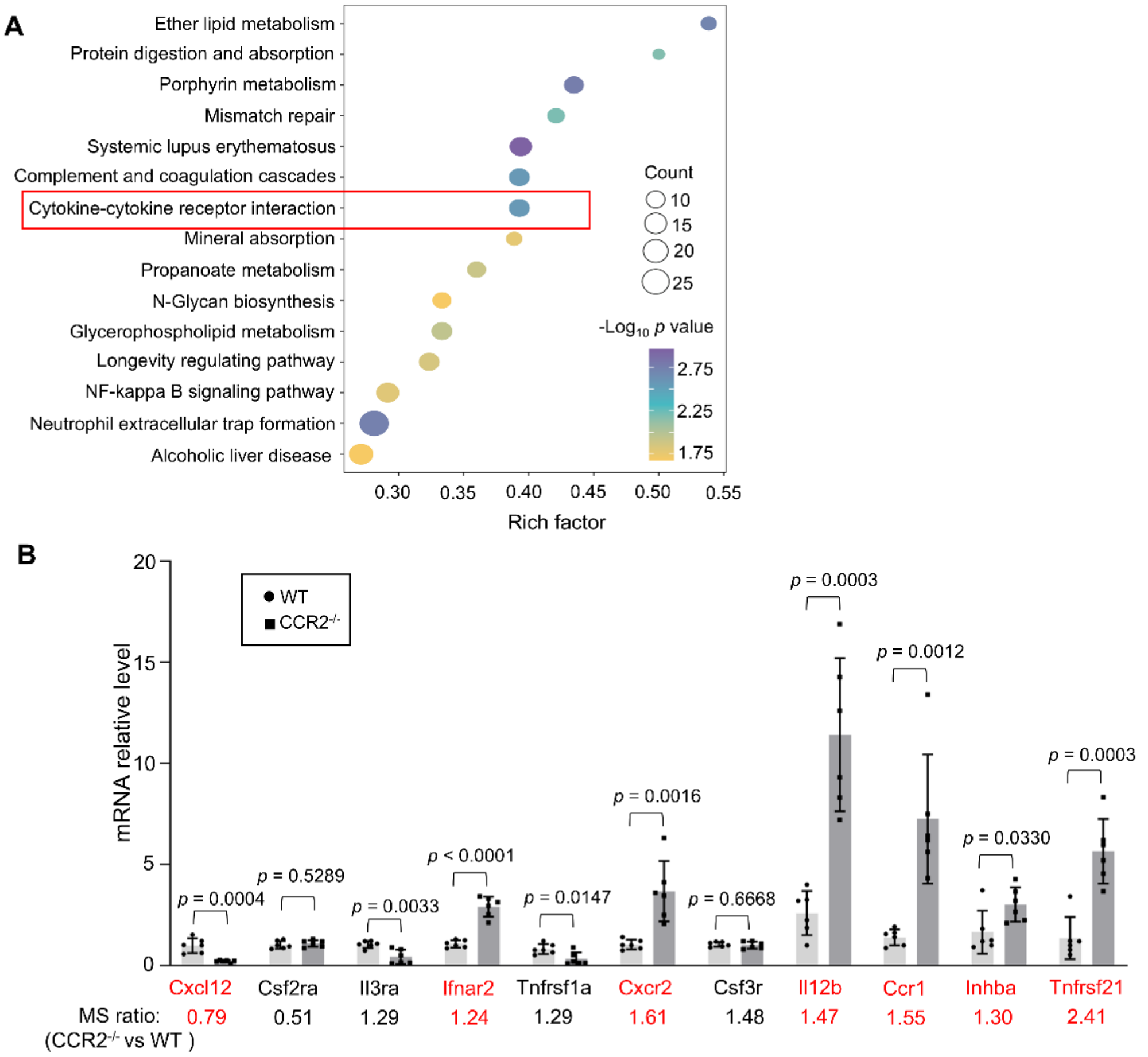

C

Group:	1	2	3	4	5	6	7	8
DOX 15 mg/kg i.p, once	-	+	+	+	+	+	+	+
CCR2 antagonist4 10 mg/kg, i.p, daily	-	-	+	+	+	+	+	+
Cytokine:	-	-	-	Cxcl12 recombinant protein (3 μg/kg, i.v, daily)	Ifnar2 neutralizing antibody (5 μg/kg, i.v, daily)	Cxcr2 inhibitor SB225002 (2 mg/kg, i.p, daily)	IL12B neutralizing antibody (3 μg/kg, i.v, daily)	CCR1 inhibitor BX471 (4 mg/kg, i.p, daily)

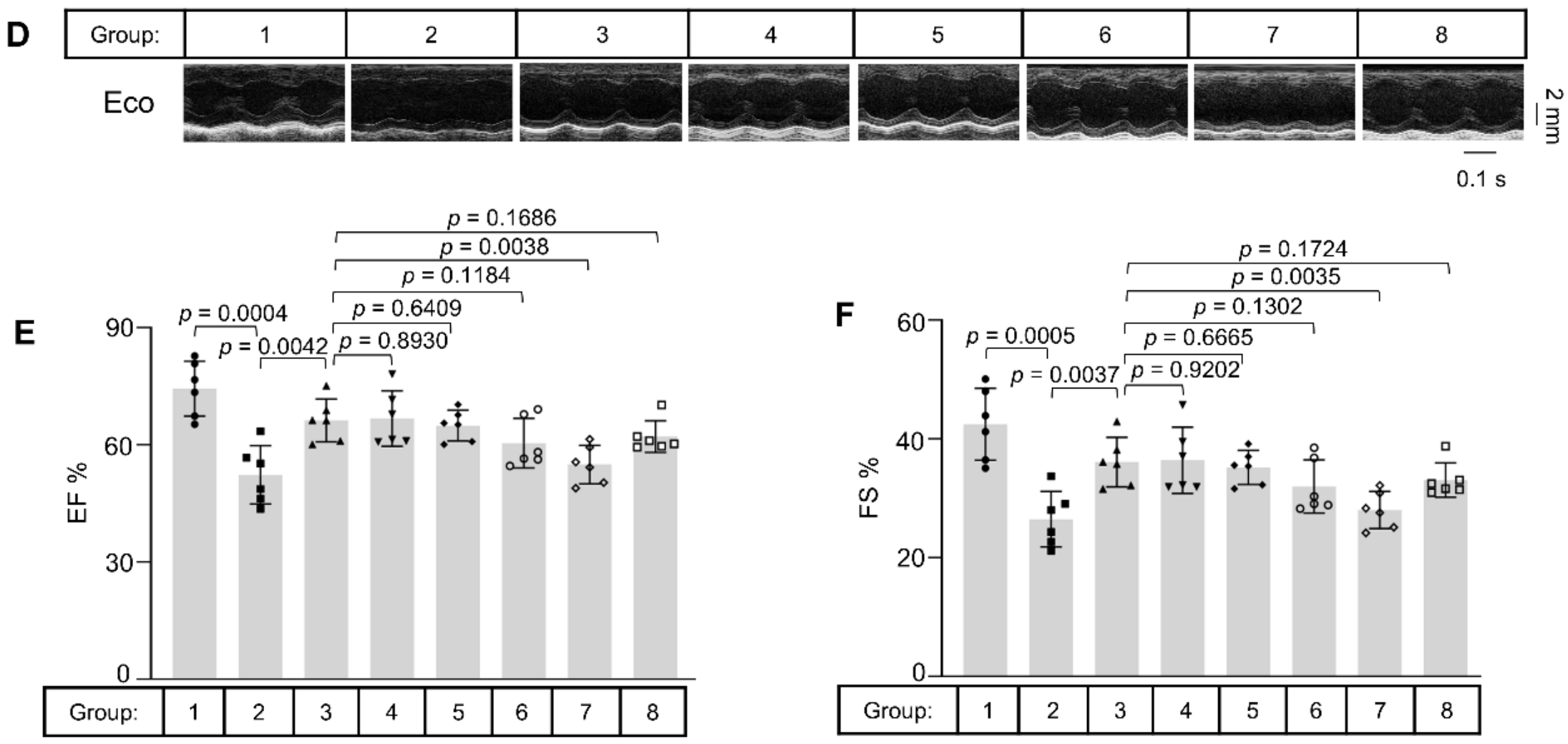

Figure S2. Proteomic screening identifies IL12B as the mediator of CCR2

blockade-induced cardioprotection

(A) KEGG pathway analysis of differentially expressed proteins in cardiac macrophages from DOX-treated WT and *CCR2*$^{-/-}$ mouse. **(B)** Quantitative PCR validation of 11 inflammatory factors or receptors from the cytokine-cytokine receptor pathway. Seven factors showed expression changes consistent with the proteomic data (highlighted in red, n = 6). **(C)** Eight experimental groups designed to test whether each candidate factor mediates the protective effect of CCR2 blockade. **(D-F)** Representative M-mode echocardiograms (scale bar: 0.1 s, 2 mm) and quantitative results of EF and FS in the indicated groups (n = 6).

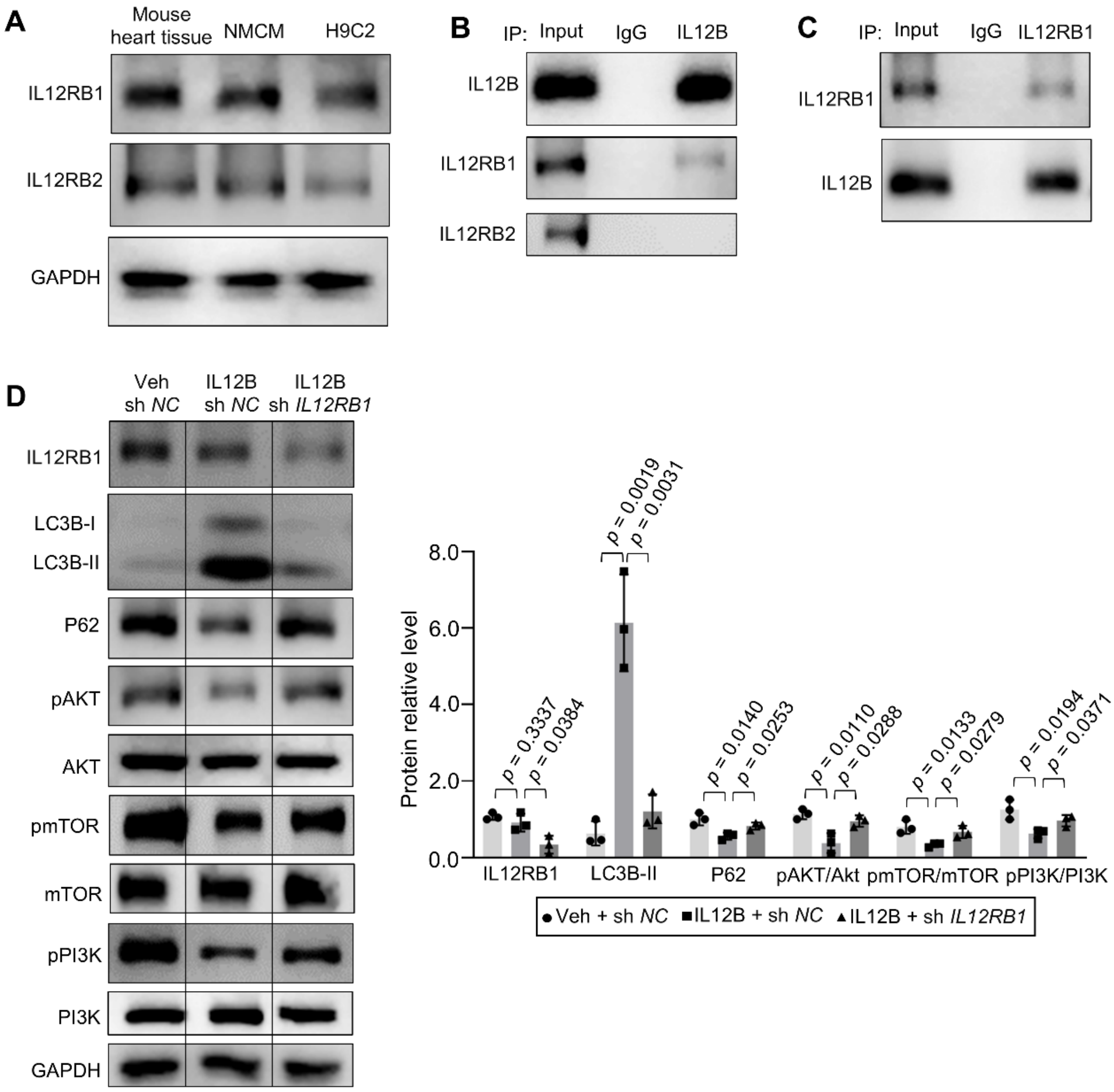

Figure S3 IL12RB1 is the functional receptor for IL12B on cardiomyocytes

(A) Western blot results of IL12RB1 and IL12RB2 protein in mouse heart tissue, primary neonatal mouse cardiomyocytes (NMCM), and H9C2 cells. **(B)** Co-immunoprecipitation assay in mouse heart tissue lysates using an anti-IL12B antibody, followed by western blot for IL12B, IL12RB1, and IL12RB2. IL12B specifically co-precipitated with IL12RB1 but not with IL12RB2. **(C)** Reciprocal co-IP assay using an anti-IL12RB1 antibody, followed by western blot for IL12B, confirming the physical interaction between IL12B and IL12RB1. **(D)** Western blot results of autophagy markers (LC3B, P62) and PI3K/Akt/mTOR signaling components in H9C2 transfected with shRNA targeting Il12rb1 (sh*IL12RB1*), followed by IL12B stimulation (2 ng/mL).

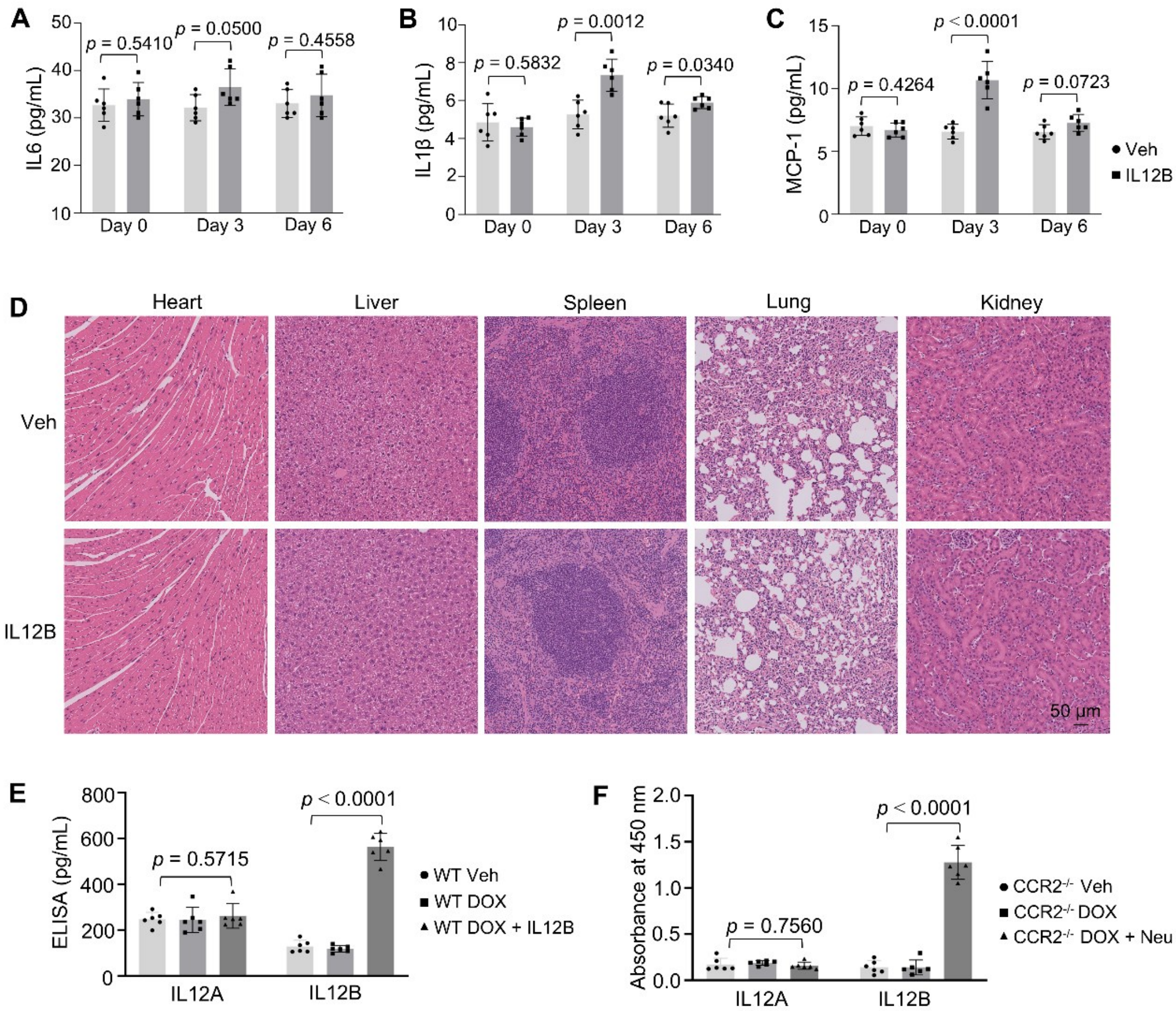

Figure S4 Safety assessment of recombinant IL12B administration in mouse

(A–C) Serum levels of IL-6, IL-1β, and MCP-1 in WT mouse treated with vehicle or recombinant IL12B (3 μg/kg, i.v.) daily for 7 consecutive days. Serum was collected at day 0 (2 h post-injection), day 3, and day 6 (n = 6). **(D)** Representative HE staining of major organs (heart, liver, lung, spleen, and kidney) from mouse treated with vehicle or IL12B at day 6. Scale bar: 50 μm. **(E)** ELISA results of circulating IL12B and IL12A levels following IL12B administration (n = 6). **(F)** Indirect ELISA confirming the specificity of the IL12B neutralizing antibody used in this study (n = 6).

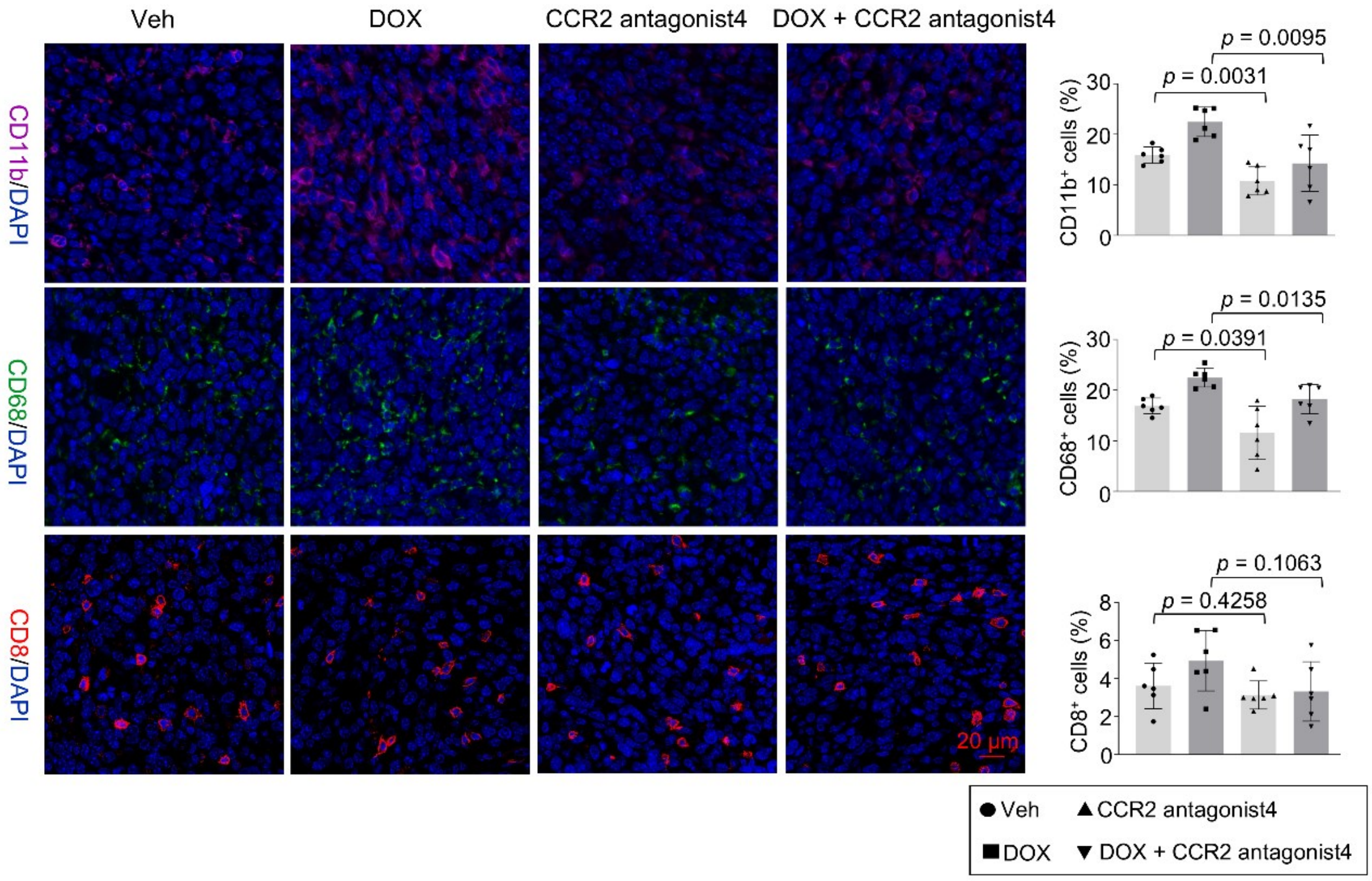

Figure S5 CCR2 antagonist4 modulates the tumor immune microenvironment without compromising adaptive anti-tumor immunity

Immunofluorescence analysis of tumor tissue sections from 4T1 tumor bearing BALB/c mouse treated with vehicle, DOX, CCR2 antagonist4 or DOX + CCR2 antagonist4. Representative images and quantitative analysis revealed that CCR2 antagonist4 does not compromise adaptive anti-tumor immunity. Scale bar: 20 μm (n = 6).

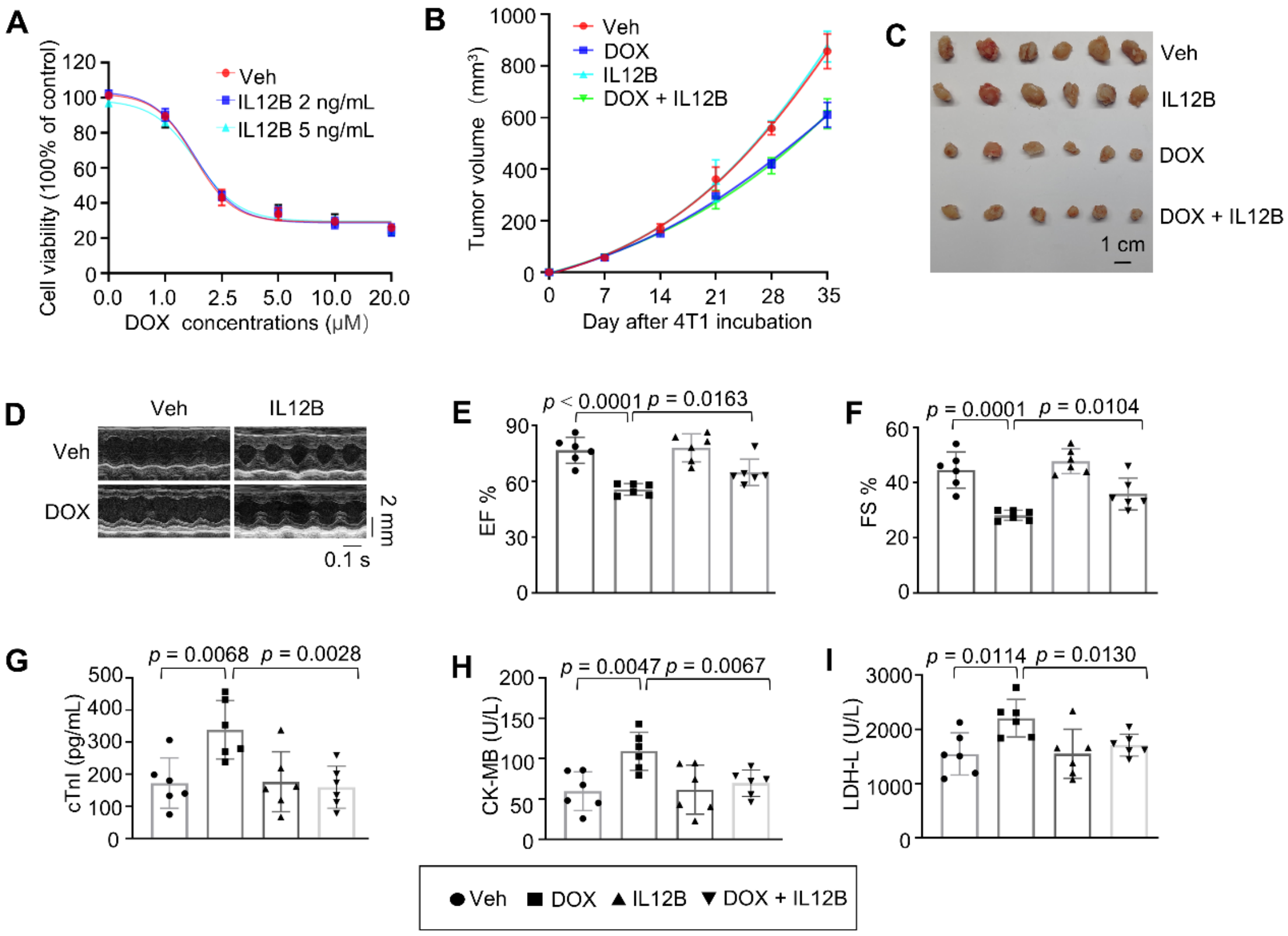

Figure S6 IL12B supplementation does not compromise antitumor efficacy of doxorubicin in the 4T1 breast cancer model

(A) Cell viability assay of 4T1 breast cancer cells treated with DOX alone or in combination with recombinant IL12B protein (n = 3 independent experiments). **(B, C)** Tumor growth curves and representative tumor images at the study endpoint in a syngeneic 4T1 breast cancer model. Scale bar: 1 cm, (n = 6). **(D)** Representative M-mode echocardiographic tracings of tumor-bearing mouse from the indicated groups. Scale bar: 0.1 s, 2 mm. **(E, F)** Quantitative results of EF and FS in the indicated groups (n = 6). **(G–I)** The levels of cardiac injury markers in serum, including cTnI, CK-MB, and LDH-L in the indicated groups (n = 6).

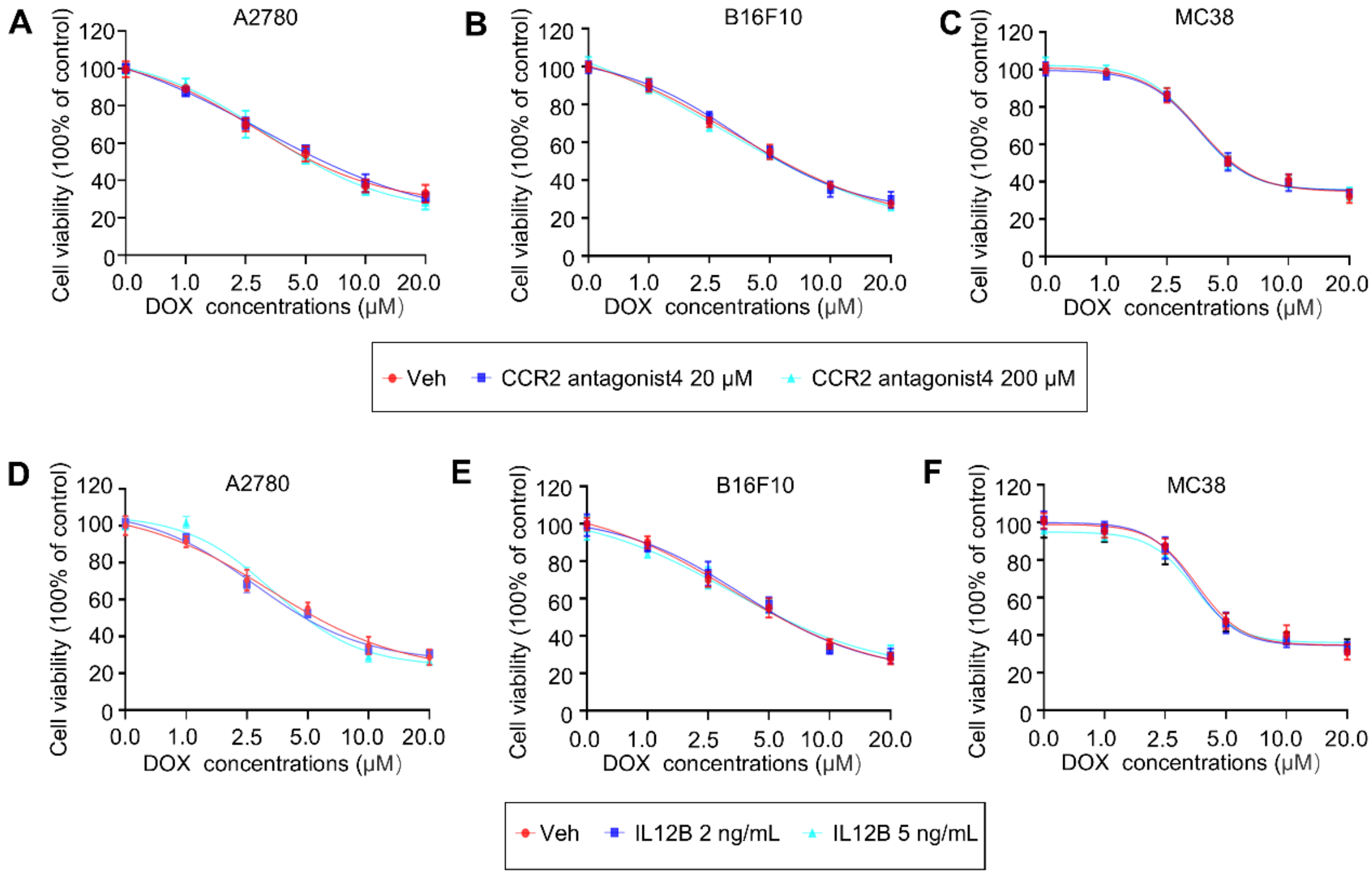

Figure S7 CCR2 antagonist4 and IL12B do not interfere with doxorubicin cytotoxicity across multiple tumor cell lines

(A–C) Cell viability assays of A2780 human ovarian carcinoma cells, B16F10 mouse melanoma cells, and MC38 mouse colon carcinoma cells treated with DOX alone or in combination with CCR2 antagonist4 (n = 3 independent experiments). **(D–F)** Cell viability assays of A2780 cells, B16F10 cells, and MC38 cells treated with DOX alone or in combination with recombinant IL12B protein (n = 3 independent experiments).

Table S1 Primers used in Real-time PCR

Gene	Organism	Primers	Sequence
Cxcl12	Mus musculus	Forword primer	TGCATCAGTGACGGTAAACCA
		Reverse primer	CACAGTTTGGAGTGTTGAGGAT
Cxcr2	Mus musculus	Forword primer	ATGCCCTCTATTCTGCCAGAT
		Reverse primer	GTGCTCCGGTTGTATAAGATGAC
Inhba	Mus musculus	Forword primer	TCCGAAGGATGGACCTAACTC
		Reverse primer	GCTTTCTGATCGCGTTGAGAAG
Ifnar2	Mus musculus	Forword primer	TGTCTGCGAGCCTAGAGACTA
		Reverse primer	AGCCGGGAATTTCGTATTGTTAT
Csf2ra	Mus musculus	Forword primer	TGCTCTTCTCCACGCTACTG
		Reverse primer	GGGGTCGAAGGTCAGGTTG
Il3ra	Mus musculus	Forword primer	CCCGCTGCTCATTCCAGTC
		Reverse primer	CACATCACGCCAGAACATCC
Tnfrsf1a	Mus musculus	Forword primer	GGGGATACATCCATCAGGGGT
		Reverse primer	GCTCGGACAGTCACTCACC
Csf3r	Mus musculus	Forword primer	TGCACCCTGACTGGAGTTAC
		Reverse primer	TGAAATCTCGATGTGTCCACAG
Ccr1	Mus musculus	Forword primer	ACTGCTGTAAGAGCCTTTGGG
		Reverse primer	AGCACCAGAATCACTAGGACA
Tnfrsf21	Mus musculus	Forword primer	GCCATGTTGACCGTACCACT
		Reverse primer	CTATGCCGTTCTCGTGCCTG
Il12b	Mus musculus	Forword primer	TGGGAGTACCCTGACTCCTG
		Reverse primer	AGGAACGCACCTTTCTGGTT
Gabarapl1	Mus musculus	Forword primer	GGACCACCCCTTCGAGTATC
		Reverse primer	CCTCTTATCCAGATCAGGGACC
Atg5	Mus musculus	Forword primer	TGTGCTTCGAGATGTGTGGTT
		Reverse primer	ACCAACGTCAAATAGCTGACTC
Atg12	Mus musculus	Forword primer	TCCCCGGAACGAGGAACTC
		Reverse primer	TTCGCTCCACAGCCCATTTC
Atg7	Mus musculus	Forword primer	ACGCTGGCACTTTATCCTGG
		Reverse primer	ACTGTTCTTACCAGCCTGTAGC
Atg3	Mus musculus	Forword primer	ACACGGTGAAGGGAAAGGC
		Reverse primer	TGGTGGACTAAGTGATCTCCAG
Gabarapl1	Rattus norvegicus	Forword primer	GGAAGTACCTTGTGCCCTCC
		Reverse primer	TAAGGCGTCCTCAGGTCTCA
Atg5	Rattus norvegicus	Forword primer	GACCACAAGCAGCTCTGGAT
		Reverse primer	ACAGGACGGAACAGCTTCTG
Atg12	Rattus norvegicus	Forword primer	GGAGGTGTGGACTGTCAAGG
		Reverse primer	ACGTTGTCCAGCTCTGACTG
Atg7	Rattus norvegicus	Forword primer	CACCTGACAGTGAGGCTGGTA
		Reverse primer	TTAAAGGGGGCGAACTGCAA
Atg3	Rattus norvegicus	Forword primer	TGACATCCTGCTGAAGGCTG
		Reverse primer	GGCCAGCAGTCTGAGGAATT

Table S2. Antibodies used in this study.

Antibody	Company	Catalog number	Application
CCR2	Affinity	DF2711	WB
ATG12	Proteintech Group	11264-1-AP	WB
LC3B	CST	83506	WB, IF
P62	Proteintech Group	18420-1-AP	WB
GABARAPL1	Proteintech Group	11010-1-AP	WB
IL12A	ABclonal	A20383	WB
IL12B	ABclonal	A13024	WB
IL12B	Proteintech Group	17645-1-AP	IF, IP
mTOR	Proteintech Group	66888-1-Ig	WB
phos-mTOR (Ser2448)	Proteintech Group	67778-1-Ig	WB
Akt	CST	9272	WB
phos-Akt (Ser473)	CST	9271	WB
AMPKα	Proteintech Group	10929-2-AP	WB
phos-AMPKα (Thr172)	Proteintech Group	80209-6-RR	WB
ERK1/2	Abmart	T40071	WB
phos-ERK1/2 (T202/Y204,	Abmart	T40072	WB
PI3K	CST	4257	WB
phos-PI3K (Tyr458)	CST	4228	WB
GAPDH	ABclonal	A19056	WB
CD68	Servicebio	GB123150	IF
CD11B	Abcam	ab133357	IF
CD8	Servicebio	GB15068	IF
IL-12RB1	Proteintech Group	13287-1-AP	WB, IP
IL-12RB2	ABclonal	A10348	WB, IP
ACTA1	Proteintech Group	23660-1-AP	IF
Anti-mouse IgG (Alexa Fluor® 488 Conjugate)	CST	4408	IF
Anti-mouse IgG (Alexa Fluor® 555 Conjugate)	CST	4409	IF
Anti-rabbit IgG (Alexa Fluor® 647 Conjugate	CST	4414	IF

Table S3 Proteomic data of cardiac macrophages isolated from DOX-treated WT and CCR2$^{-/-}$ mouse.

Accession	Gene	WT/CCR2$^{-/-}$ ratio	Log_2 WT/CCR2$^{-/-}$ ratio	*p* value	*p* adjust
A0A087WP63	Gm42715	1.251	0.323	0.003	0.011
A0A087WPM0	Selenoh	1.873	0.905	0.001	0.006
A0A087WRY3	Nucks1	1.603	0.681	0.003	0.011
A0A0A6YVR9	Surf1	1.304	0.383	0.003	0.010
A0A0A6YWV7	Ifrd2	1.206	0.270	0.007	0.017
A0A0A6YX26	Rpl31	1.309	0.388	0.004	0.013
A0A0B4J1E2	Snw1	1.215	0.281	0.000	0.004
A0A0G2JDV8	Cnn3	1.218	0.285	0.000	0.004
A0A0G2JE25	Nras	2.175	1.121	0.001	0.007
A0A0G2JES3	Rpl9	1.308	0.388	0.000	0.004
A0A0G2JF26	Bank1	1.610	0.687	0.017	0.035
A0A0G2JF67	Pde5a	1.572	0.653	0.031	0.055
A0A0G2JGF1	Il6ra	1.251	0.323	0.006	0.016
A0A0J9YTY0	Sept11	1.226	0.294	0.009	0.021
A0A0J9YUN2	Ube2a	1.531	0.614	0.000	0.004
A0A0J9YUZ4	Hmgb1	1.207	0.271	0.002	0.008
A0A0N4SUU3	Eefsec	1.217	0.283	0.000	0.004
A0A0N4SVC2	Tra2a	1.336	0.418	0.006	0.017
A0A0R4J011	Tcf3	2.053	1.037	0.001	0.005
A0A0R4J046	Cebpz	1.221	0.288	0.001	0.007
A0A0R4J049	Prmt5	1.211	0.276	0.000	0.003
A0A0R4J083	Acadl	1.203	0.267	0.000	0.004
A0A0R4J0A2	Rhd	2.552	1.351	0.000	0.003
A0A0R4J0I5	Tmem71	1.354	0.437	0.000	0.004
A0A0R4J0K1	Selenof	1.452	0.538	0.015	0.031
A0A0R4J0P2	Haspin	1.210	0.275	0.003	0.010
A0A0R4J187	Xrcc6	1.370	0.455	0.002	0.007
A0A0R4J1G9	Steap3	1.453	0.539	0.003	0.010
A0A0R4J2B5	310022A10R	1.200	0.263	0.000	0.004
A0A0U1RNW7	Kat8	1.461	0.547	0.000	0.005
A0A0U1RNY9	Nfatc2ip	1.421	0.506	0.010	0.022
A0A0U1RP87	Vamp5	1.347	0.430	0.002	0.009
A0A0U1RPK5	Bckdha	2.136	1.095	0.000	0.003
A0A0U1RPP1	St3gal5	1.347	0.430	0.007	0.017
A0A0U1RQ22	Ube2s	1.252	0.324	0.003	0.010
A0A140LHE9	Pigq	1.549	0.632	0.007	0.018
A0A140T8J4	Hebp1	1.825	0.868	0.000	0.004
A0A140T8S5	Champ1	1.335	0.417	0.002	0.008
A0A1B0GR56	Orc6	1.241	0.312	0.002	0.007
A0A1B0GSE5	Cyld	1.241	0.311	0.003	0.010
A0A1L1SRC3	Rbm7	1.366	0.450	0.000	0.003
A0A1L1SRM5	Pou2af1	1.693	0.760	0.001	0.007
A0A1L1SSW1	Lars2	1.209	0.274	0.002	0.009
A0A1L1SVG6	Slc44a2	1.329	0.410	0.000	0.003
A0A1W2P7R5	Tmem19	1.625	0.700	0.001	0.007
A0A1Y7VK19	Isca2	1.233	0.302	0.011	0.024
A0A1Y7VLP0	Atp5mpl	1.732	0.793	0.001	0.007
A0A286YDQ6	Pcgf5	1.420	0.506	0.014	0.029
A0A2I3BPX9	Elf1	1.354	0.437	0.002	0.009
A0A2I3BQL9	Pck2	1.242	0.313	0.004	0.012
A0A2R8VHQ1	Smug1	1.206	0.270	0.001	0.005
A0A3B2W3M7	Hmgn1	1.515	0.599	0.001	0.005
A0A3B2W450	Snrpc	1.261	0.334	0.000	0.003
A0A3B2W812	Cd2ap	1.393	0.478	0.001	0.006
A0A3B2WBC6	Polr2m	4.695	2.231	0.000	0.003
A0A3B2WBH6	Rhag	2.511	1.328	0.004	0.011
A0A3Q4EBK4	Bin1	1.508	0.593	0.003	0.011

A0A3Q4EH04	Srsf7	1.208	0.272	0.001	0.007
A0A494B990	Lpxn	1.344	0.426	0.031	0.055
A0A494B9K4	Add3	1.296	0.374	0.015	0.032
A0A494BA96	Kat5	1.312	0.392	0.003	0.010
A0A494BAG7	Bloc1s2	1.205	0.269	0.004	0.011
A0A494BB84	Sfxn3	1.287	0.365	0.003	0.010
A0A494BBL1	Akap8l	1.433	0.519	0.006	0.017
A0A494BBN2	Mrpl28	1.567	0.648	0.000	0.004
A0A498WFS2	Ubxn1	1.303	0.382	0.004	0.011
A2A3Z2	Zbtb33	1.230	0.299	0.004	0.012
A2A5R8	Stau1	1.215	0.281	0.002	0.009
A2A6Q8	Myl4	2.324	1.216	0.002	0.008
A2A8R7	Tut4	1.271	0.346	0.002	0.008
A2AD40	Tal1	1.390	0.475	0.005	0.015
A2AFM1	Alas2	2.022	1.016	0.002	0.008
A2AFW6	Mtch2	1.215	0.280	0.001	0.005
A2AL12	Hnrnpa3	1.353	0.436	0.003	0.011
A2AP78	Hmgb3	1.306	0.385	0.003	0.010
A2AQ07	Tubb1	1.687	0.755	0.019	0.037
A2ARZ7	Rab22a	1.315	0.395	0.013	0.029
A2AWT5	Ubtf	1.282	0.358	0.000	0.003
A2RSX7	Tyw5	1.419	0.505	0.000	0.003
A3KMP2	Ttc38	1.485	0.570	0.003	0.010
A6PWY4	Wdr76	1.596	0.675	0.011	0.024
A8DUK4	Hbb-bs	2.712	1.439	0.013	0.028
A9YVJ5	Gbp11	1.551	0.633	0.016	0.032
B0QZP7	Tmc8	1.637	0.711	0.001	0.007
B0R031	Lrrc57	1.624	0.700	0.000	0.004
B1AQR8	Lgals9	1.535	0.618	0.003	0.010
B1AUY8	Naa10	1.244	0.315	0.003	0.010
B1AX95	Tprgl	1.271	0.346	0.002	0.009
B1AYU4	Cwc22	1.225	0.293	0.000	0.003
B1AZ46	Baiap2	1.341	0.423	0.003	0.011
B2KG29	Med21	1.599	0.677	0.001	0.007
B2RQS1	Strn3	1.363	0.447	0.000	0.004
B8JJF0	Cep57	1.671	0.740	0.011	0.025
D0QMC3	Mndal	1.314	0.394	0.024	0.044
D0VYV6	Epb41l3	1.538	0.621	0.001	0.005
D3YTQ3	Hnrnpdl	1.395	0.480	0.001	0.005
D3YTQ9	Rps15	1.528	0.612	0.037	0.063
D3YTR5	Mbd3	1.247	0.319	0.004	0.013
D3YUM1	Ndufv1	1.272	0.347	0.005	0.014
D3YWR2	Blnk	1.570	0.651	0.000	0.004
D3YYR5	P2rx4	1.278	0.354	0.004	0.012
D3YZ89	Mtfr1	1.244	0.315	0.018	0.036
D3YZA1	Chtop	1.306	0.385	0.030	0.053
D3YZB0	Fam129c	1.897	0.924	0.000	0.003
D3YZN4	Spg7	1.472	0.558	0.001	0.007
D3Z0K9	Iscu	1.346	0.429	0.001	0.005
D3Z0R8	Ttc7	1.207	0.271	0.033	0.057
D3Z3V3	Sept1	1.370	0.454	0.002	0.007
D3Z555	Jakmip1	1.559	0.641	0.005	0.013
D3Z5T2	Armc10	1.222	0.289	0.001	0.007
D3Z6T3	Ctse	1.578	0.658	0.001	0.007
D3Z774	Ezh2	1.203	0.267	0.007	0.018
D3Z793	Gtf2a2	1.279	0.354	0.003	0.011
D3Z7M9	Anp32a	1.535	0.618	0.000	0.004
D6RD07	Pdpr	1.425	0.511	0.004	0.012
E0CXN5	Gpd1	1.838	0.878	0.000	0.003
E0CXT6	Cmbl	1.815	0.860	0.000	0.004
E9PUF4	Rbm26	1.329	0.410	0.000	0.005

E9PUG6	Ifi203	1.552	0.635	0.019	0.038
E9PV38	Ces2g	1.715	0.778	0.002	0.009
E9PVV1	Tcf3	1.754	0.810	0.001	0.005
E9PVX6	Mki67	1.316	0.396	0.000	0.003
E9PWK1	Ephx1	1.220	0.287	0.009	0.021
E9PXS4	Ptp4a3	1.472	0.558	0.000	0.003
E9Q2Z1	Cecr2	2.500	1.322	0.002	0.008
E9Q397	Sptb	1.850	0.887	0.004	0.013
E9Q421	Pigk	1.268	0.342	0.000	0.005
E9Q479	H2-Ob	1.884	0.914	0.019	0.037
E9Q509	Pklr	1.551	0.633	0.002	0.008
E9Q517	Slc6a9	1.237	0.307	0.014	0.029
E9Q5E0	Mef2d	1.510	0.595	0.000	0.002
E9Q7N5	Gfm2	1.245	0.316	0.007	0.018
E9Q8D0	Dnajc21	1.250	0.322	0.000	0.003
E9Q8N3	Uros	1.383	0.467	0.009	0.020
E9Q9E8	Cd84	1.257	0.330	0.007	0.017
E9QNN1	Dhx9	1.240	0.311	0.000	0.003
F2Z3X0	Capn7	1.360	0.444	0.002	0.008
F6R4N2	Inpp4a	1.408	0.493	0.022	0.041
F6RPD4	Phax	1.618	0.694	0.001	0.006
F6T2S0	Fech	1.978	0.984	0.001	0.006
F6TQP9	Slco2b1	1.224	0.291	0.002	0.007
F6WLY0	Lrmp	1.267	0.341	0.001	0.007
F6WM75	Ccdc167	1.675	0.744	0.009	0.021
F6YBB8	Nob1	1.467	0.553	0.000	0.002
F6Z9L5	Senp3	1.336	0.418	0.000	0.003
F7AAP4	Atp2b4	1.295	0.373	0.006	0.017
F7CB97	Clip1	1.334	0.416	0.006	0.015
F7D1I5	Gypc	1.477	0.563	0.010	0.024
F8VPY2	Taf5	1.237	0.307	0.000	0.004
F8VQC7	Ktn1	1.265	0.339	0.003	0.011
F8VQG7	N4bp2	1.542	0.624	0.000	0.003
F8WGW3	Srbd1	1.217	0.283	0.001	0.005
F8WJB9	Evl	1.525	0.608	0.002	0.009
G3UWY0	Guf1	1.469	0.555	0.002	0.009
G3UXT7	Fus	1.264	0.338	0.000	0.004
G3UYV7	Rps28	1.541	0.624	0.000	0.003
G3UZ60	Gm49325	1.776	0.828	0.000	0.004
G3UZA0	Krr1	1.216	0.283	0.000	0.003
G3UZY2	Txn2	1.202	0.266	0.008	0.019
G3V008	Pax5	1.354	0.437	0.002	0.008
G3X963	Atad2	1.219	0.286	0.001	0.006
G3X987	Gimap9	1.254	0.327	0.018	0.036
G3XA39	Nrf1	1.742	0.801	0.001	0.005
G3XA66	Mtx1	1.290	0.368	0.001	0.006
G5E850	Cyb5a	1.324	0.405	0.004	0.012
G5E8G0	Hnrnpd	1.342	0.424	0.001	0.006
G5E8H3	Ikzf1	1.248	0.319	0.001	0.005
G5E8R3	Pcx	1.244	0.315	0.004	0.013
G5E924	Hnrnpl	1.306	0.385	0.000	0.003
G8JL68	Myef2	1.293	0.370	0.012	0.027
G8JL84	Ank1	1.837	0.877	0.002	0.008
H3BJ75	Dctn5	1.375	0.459	0.004	0.013
H3BKE2	Foxo3	1.417	0.503	0.000	0.003
H3BKX8	Itgad	1.668	0.738	0.001	0.007
H7BWZ1	Ufm1	1.406	0.491	0.002	0.009
H7BX33	933434E20R	1.874	0.906	0.001	0.007
H7BX95	Srsf1	1.731	0.791	0.001	0.005
J3QK52	Noc2l	1.212	0.277	0.002	0.008
J3QMN4	Txnrd2	1.251	0.323	0.020	0.039

J3QNW0	Dnmt1	2.192	1.133	0.000	0.004
J3QQ00	Homer3	1.345	0.427	0.014	0.029
K4DI63	Creg1	1.208	0.273	0.001	0.005
M0QWS4	Ufc1	1.216	0.282	0.004	0.012
O08583	Alyref	1.274	0.349	0.011	0.024
O08586	Pten	1.219	0.286	0.006	0.016
O08688	Capn5	1.217	0.284	0.008	0.019
O08797	Serpinb9	1.204	0.268	0.043	0.071
O08900	Ikzf3	1.919	0.940	0.000	0.005
O09106	Hdac1	1.213	0.279	0.000	0.004
O35326	Srsf5	1.386	0.471	0.000	0.004
O35344	Kpna3	1.247	0.319	0.023	0.043
O35405	Pld3	1.796	0.845	0.000	0.002
O35654	Pold2	1.356	0.440	0.003	0.010
O35683	Ndufa1	1.541	0.624	0.002	0.007
O35943	Fxn	1.341	0.423	0.007	0.017
O54864	Suv39h1	1.229	0.297	0.000	0.003
O54941	Smarce1	1.219	0.286	0.000	0.004
O55042	Snca	1.476	0.562	0.000	0.004
O55128	Sap18	1.406	0.492	0.000	0.003
O70126	Aurkb	1.222	0.289	0.000	0.004
O70152	Dpm1	1.744	0.802	0.002	0.008
O70165	Fcn1	2.377	1.249	0.001	0.007
O70200	Aif1	1.457	0.543	0.006	0.016
O88199	Chst3	1.720	0.782	0.001	0.006
O88441	Mtx2	1.366	0.450	0.000	0.003
O88508	Dnmt3a	1.228	0.296	0.001	0.007
O88551	Cldn1	1.284	0.360	0.008	0.019
O88554	Parp2	1.278	0.353	0.003	0.010
O88895	Hdac3	1.501	0.586	0.003	0.010
O89017	Lgmn	2.584	1.370	0.000	0.005
O89023	Tpp1	1.382	0.467	0.000	0.003
O89086	Rbm3	1.621	0.697	0.000	0.004
P00920	Ca2	1.565	0.646	0.004	0.013
P01872	Ighm	1.893	0.920	0.004	0.013
P02798	Mt2	1.682	0.751	0.008	0.020
P03899	Mtnd3	1.953	0.966	0.000	0.004
P04117	Fabp4	1.897	0.924	0.001	0.006
P04919	Slc4a1	1.863	0.898	0.001	0.006
P05132	Prkaca	1.272	0.348	0.000	0.004
P06240	Lck	1.389	0.475	0.031	0.054
P07309	Ttr	1.554	0.636	0.013	0.029
P07901	Hsp90aa1	1.242	0.313	0.000	0.005
P07903	Ercc1	1.272	0.347	0.005	0.014
P08032	Spta1	2.042	1.030	0.002	0.009
P09405	Ncl	1.312	0.391	0.001	0.005
P09528	Fth1	1.948	0.962	0.000	0.002
P10126	Eef1a1	1.481	0.567	0.010	0.022
P10417	Bcl2	1.475	0.560	0.000	0.003
P10518	Alad	1.267	0.342	0.001	0.007
P10605	Ctsb	1.428	0.514	0.000	0.002
P10833	Rras	1.207	0.271	0.005	0.014
P10852	Slc3a2	1.269	0.344	0.001	0.006
P10922	H1f0	1.202	0.266	0.004	0.013
P11032	Gzma	1.787	0.838	0.004	0.012
P11087	Col1a1	1.407	0.492	0.046	0.074
P11911	Cd79a	2.995	1.582	0.004	0.012
P13634	Ca1	2.196	1.135	0.003	0.010
P14094	Atp1b1	1.422	0.508	0.001	0.006
P14206	Rpsa	1.376	0.461	0.002	0.009
P14733	Lmnb1	1.233	0.302	0.002	0.008

P14901	Hmox1	1.985	0.989	0.002	0.007
P15105	Glul	1.280	0.356	0.001	0.006
P15530	Cd79b	1.926	0.946	0.013	0.029
P15864	Hist1h1c	1.366	0.450	0.003	0.010
P16330	Cnp	1.745	0.804	0.000	0.003
P17679	Gata1	1.736	0.796	0.000	0.004
P17918	Pcna	1.269	0.343	0.000	0.004
P18242	Ctsd	1.534	0.617	0.000	0.002
P18572	Bsg	1.353	0.436	0.007	0.017
P19157	Gstp1	1.532	0.615	0.000	0.004
P19437	Ms4a1	2.170	1.117	0.001	0.007
P20108	Prdx3	1.339	0.421	0.001	0.006
P21447	Abcb1a	1.241	0.312	0.000	0.005
P21550	Eno3	1.312	0.392	0.007	0.018
P21981	Tgm2	1.379	0.463	0.000	0.004
P23611	Irf8	1.204	0.268	0.001	0.007
P24270	Cat	1.264	0.338	0.000	0.004
P24549	Aldh1a1	1.481	0.566	0.022	0.041
P25085	Il1rn	1.284	0.361	0.017	0.035
P26516	Psmd7	1.281	0.357	0.000	0.003
P26638	Sars	1.263	0.337	0.002	0.008
P26645	Marcks	1.512	0.597	0.000	0.004
P27641	Xrcc5	1.288	0.365	0.002	0.007
P27661	H2afx	1.500	0.585	0.000	0.003
P28843	Dpp4	1.372	0.456	0.004	0.012
P29533	Vcam1	1.335	0.417	0.003	0.010
P29758	Oat	1.460	0.546	0.000	0.003
P31428	Dpep1	1.589	0.668	0.001	0.007
P31996	Cd68	1.299	0.377	0.009	0.021
P32067	Ssb	1.215	0.281	0.000	0.004
P35459	Ly6d	2.442	1.288	0.003	0.010
P35550	Fbl	1.217	0.284	0.000	0.004
P35980	Rpl18	1.973	0.981	0.000	0.003
P36423	Tbxas1	1.249	0.321	0.029	0.051
P36552	Cpox	1.724	0.786	0.005	0.014
P38647	Hspa9	1.263	0.336	0.003	0.010
P40124	Cap1	1.254	0.326	0.006	0.017
P40224	Cxcl12	1.261	0.335	0.001	0.006
P40237	Cd82	1.262	0.336	0.003	0.011
P41241	Csk	1.313	0.393	0.000	0.004
P43247	Msh2	1.259	0.332	0.000	0.004
P43274	Hist1h1e	1.426	0.512	0.001	0.006
P43275	Hist1h1a	1.605	0.682	0.000	0.003
P43276	Hist1h1b	1.207	0.271	0.000	0.003
P43277	Hist1h1d	1.352	0.435	0.000	0.003
P43346	Dck	1.244	0.315	0.008	0.019
P45952	Acadm	1.258	0.331	0.000	0.004
P48025	Syk	1.408	0.494	0.001	0.007
P48193	Epb41	1.464	0.550	0.001	0.006
P48678	Lmna	1.372	0.457	0.013	0.028
P48754	Brca1	1.276	0.352	0.000	0.004
P49222	Epb42	1.625	0.700	0.001	0.007
P49813	Tmod1	1.435	0.521	0.001	0.006
P50544	Acadvl	1.226	0.294	0.000	0.004
P51175	Ppox	1.379	0.464	0.001	0.005
P51859	Hdgf	1.262	0.336	0.001	0.005
P51881	Slc25a5	1.284	0.361	0.001	0.007
P52431	Pold1	1.228	0.296	0.001	0.007
P52503	Ndufs6	1.648	0.721	0.006	0.016
P53986	Slc16a1	1.312	0.392	0.000	0.002
P54276	Msh6	1.294	0.372	0.000	0.004

P56212	Arpp19	1.361	0.445	0.001	0.006
P56390	Cks2	1.693	0.760	0.001	0.006
P56480	Atp5f1b	1.589	0.668	0.000	0.004
P57759	Erp29	1.262	0.336	0.003	0.010
P58021	Tm9sf2	1.253	0.325	0.042	0.069
P58283	Rnf216	1.288	0.365	0.007	0.018
P59017	Bcl2l13	1.234	0.303	0.000	0.004
P59708	Sf3b6	1.313	0.393	0.000	0.003
P60122	Ruvbl1	1.234	0.303	0.000	0.003
P60824	Cirbp	1.622	0.698	0.000	0.003
P61089	Ube2n	1.358	0.442	0.000	0.004
P61358	Rpl27	1.608	0.685	0.002	0.009
P62071	Rras2	1.621	0.697	0.004	0.011
P62254	Ube2g1	1.213	0.278	0.010	0.024
P62806	Hist1h4a	2.906	1.539	0.001	0.005
P62821	Rab1A	1.299	0.378	0.002	0.009
P62827	Ran	1.506	0.591	0.000	0.004
P62862	Fau	2.659	1.411	0.000	0.003
P62918	Rpl8	1.259	0.333	0.006	0.015
P63028	Tpt1	1.357	0.441	0.000	0.005
P63038	Hspd1	1.398	0.483	0.000	0.003
P63147	Ube2b	1.372	0.456	0.001	0.007
P63280	Ube2i	1.606	0.683	0.002	0.008
P63325	Rps10	3.273	1.711	0.001	0.005
P67778	Phb	1.527	0.610	0.000	0.003
P68254	Ywhaq	1.330	0.411	0.002	0.008
P68368	Tuba4a	1.299	0.377	0.001	0.006
P68369	Tuba1a	1.508	0.592	0.000	0.004
P68372	Tubb4b	1.241	0.312	0.004	0.012
P68433	Hist1h3a	1.716	0.779	0.000	0.005
P70202	Lxn	1.378	0.463	0.000	0.004
P70224	Gimap1	1.203	0.267	0.018	0.035
P70288	Hdac2	1.206	0.270	0.000	0.004
P70290	Mpp1	1.292	0.370	0.000	0.003
P80314	Cct2	1.204	0.268	0.005	0.014
P83887	Tubg1	1.424	0.510	0.002	0.008
P83917	Cbx1	1.266	0.340	0.002	0.007
P84086	Cplx2	2.205	1.141	0.002	0.008
P84089	Erh	1.548	0.631	0.005	0.014
P84096	Rhog	1.289	0.366	0.001	0.007
P85094	Isoc2a	1.213	0.279	0.010	0.023
P97303	Bach2	1.606	0.683	0.002	0.008
P97372	Psme2	1.221	0.288	0.000	0.004
P97429	Anxa4	1.248	0.319	0.000	0.005
P97494	Gclc	1.365	0.449	0.001	0.006
P97765	Wbp2	1.793	0.842	0.017	0.035
P97807	Fh	1.323	0.404	0.000	0.003
P97855	G3bp1	1.467	0.553	0.000	0.004
P99024	Tubb5	1.473	0.558	0.000	0.004
Q00941	Csf2ra	1.975	0.982	0.002	0.008
Q01149	Col1a2	1.678	0.747	0.047	0.076
Q01320	Top2a	1.255	0.328	0.000	0.003
Q02013	Aqp1	2.867	1.520	0.004	0.012
Q03265	Atp5f1a	1.298	0.377	0.000	0.003
Q04447	Ckb	1.540	0.623	0.006	0.015
Q05117	Acp5	1.730	0.791	0.003	0.010
Q08857	Cd36	1.987	0.991	0.001	0.005
Q08943	Ssrp1	1.365	0.449	0.000	0.003
Q09143	Slc7a1	1.200	0.263	0.007	0.018
Q0VBL3	Rbm15	1.234	0.304	0.002	0.008
Q0VEJ0	Cep76	1.308	0.387	0.021	0.040

Q1HFZ0	Nsun2	1.268	0.343	0.000	0.003
Q2VLH6	Cd163	1.957	0.968	0.000	0.003
Q3T9T1	Tmcc2	1.763	0.818	0.003	0.010
Q3TAA7	Stk11ip	1.229	0.298	0.011	0.024
Q3TE85	Hmga1	1.219	0.285	0.003	0.010
Q3TF41	Nap1l1	1.226	0.294	0.000	0.004
Q3TFP0	Srsf10	1.529	0.612	0.000	0.003
Q3TI69	Apobec3	1.229	0.297	0.004	0.013
Q3TIX9	Usp39	1.498	0.583	0.000	0.005
Q3TWF6	Wdr70	1.212	0.278	0.003	0.010
Q3TWW8	Srsf6	1.244	0.315	0.001	0.006
Q3U125	Prxl2a	1.578	0.658	0.002	0.009
Q3U1J0	Slc38a5	1.471	0.557	0.002	0.009
Q3U3C9	Gse1	1.223	0.291	0.001	0.007
Q3U8Y1	Mrps11	1.340	0.423	0.000	0.004
Q3U9G9	Lbr	1.228	0.297	0.002	0.009
Q3UB58	Cd72	1.391	0.476	0.004	0.012
Q3UGR5	Hdhd2	1.417	0.503	0.001	0.005
Q3UIA2	Arhgap17	1.216	0.282	0.000	0.002
Q3UJK4	Gtpbp2	1.278	0.354	0.017	0.034
Q3UJU9	Rmdn3	1.484	0.570	0.001	0.005
Q3ULJ0	Gpd1l	1.254	0.326	0.000	0.003
Q3UUB6	Ebf3	2.551	1.351	0.000	0.003
Q3UX10	Tubal3	1.414	0.500	0.006	0.016
Q3V3C7	Nmnat3	1.455	0.541	0.000	0.003
Q4FZF3	Ddx49	1.432	0.518	0.006	0.015
Q4VAE3	Tmem65	1.441	0.527	0.000	0.004
Q4VBD2	Tapt1	1.517	0.601	0.000	0.005
Q5HZH2	Tsr3	1.591	0.670	0.007	0.017
Q5STS9	Bcl11a	1.390	0.475	0.000	0.004
Q5SUT0	Ewsr1	1.278	0.354	0.012	0.027
Q5SYD0	Myo1d	1.203	0.267	0.012	0.026
Q5U452	Trp53rka	1.503	0.588	0.004	0.012
Q60611	Satb1	1.441	0.527	0.000	0.004
Q60759	Gcdh	1.273	0.348	0.035	0.060
Q60848	Hells	1.222	0.289	0.000	0.003
Q60854	Serpinb6	1.542	0.625	0.000	0.003
Q60865	Caprin1	1.233	0.302	0.002	0.007
Q60932	Vdac1	1.283	0.359	0.002	0.008
Q60953	Pml	1.552	0.634	0.005	0.015
Q61029	Tmpo	1.593	0.672	0.001	0.005
Q61103	Dpf2	1.248	0.320	0.008	0.019
Q61133	Gstt2	1.212	0.278	0.023	0.044
Q61152	Ptpn18	1.229	0.297	0.004	0.013
Q61171	Prdx2	1.279	0.355	0.001	0.006
Q61191	Hcfc1	1.244	0.316	0.000	0.004
Q61205	Pafah1b3	1.490	0.575	0.000	0.004
Q61286	Tcf12	1.334	0.415	0.005	0.014
Q61458	Ccnh	1.211	0.276	0.001	0.005
Q61470	Cd37	1.243	0.313	0.004	0.013
Q61559	Fcgrt	1.276	0.352	0.013	0.028
Q61635	Ifi47	1.208	0.273	0.020	0.038
Q61686	Cbx5	1.452	0.538	0.003	0.010
Q61771	Kif3b	1.236	0.305	0.022	0.041
Q61830	Mrc1	1.284	0.360	0.002	0.007
Q61879	Myh10	1.654	0.726	0.013	0.029
Q61881	Mcm7	1.220	0.287	0.000	0.003
Q62048	Pea15	1.384	0.469	0.002	0.008
Q62186	Ssr4	1.734	0.794	0.001	0.007
Q62193	Rpa2	1.209	0.274	0.015	0.031
Q62230	Siglec1	1.803	0.850	0.000	0.004

Q62318	Trim28	1.320	0.401	0.000	0.004
Q62351	Tfrc	1.793	0.843	0.001	0.006
Q62418	Dbnl	1.201	0.264	0.000	0.004
Q62422	Ostf1	2.201	1.138	0.001	0.006
Q62425	Ndufa4	1.479	0.565	0.000	0.005
Q64287	Irf4	1.593	0.671	0.006	0.016
Q64433	Hspe1	1.306	0.385	0.001	0.006
Q64511	Top2b	1.225	0.292	0.001	0.005
Q64524	Hist2h2be	1.329	0.410	0.005	0.014
Q64674	Srm	1.215	0.281	0.001	0.005
Q673H1	Tusc1	1.973	0.980	0.001	0.005
Q68ED3	Tent4b	1.252	0.324	0.002	0.008
Q6A0D4	Rftn1	1.266	0.340	0.001	0.006
Q6DFW4	Nop58	1.230	0.299	0.001	0.005
Q6DVA0	Lemd2	1.307	0.387	0.001	0.007
Q6GSS7	Hist2h2aa1	2.733	1.450	0.001	0.005
Q6IR34	Gpsm1	1.212	0.277	0.012	0.027
Q6NZL1	Dhx37	1.246	0.317	0.003	0.010
Q6P3A8	Bckdhb	1.241	0.312	0.018	0.036
Q6PCM2	Ints6	1.288	0.365	0.001	0.005
Q6PEE3	Rrm2b	1.369	0.453	0.005	0.014
Q6PFD6	Kif18b	1.480	0.566	0.001	0.005
Q6ZPJ3	Ube2o	1.427	0.513	0.001	0.005
Q6ZWQ5	Snx12	1.768	0.822	0.003	0.010
Q6ZWQ9	Myl12a	1.308	0.388	0.000	0.004
Q76N33	Stambpl1	1.257	0.329	0.000	0.003
Q78ZA7	Nap1l4	1.370	0.454	0.001	0.005
Q78ZM0	Snx3	1.243	0.314	0.002	0.008
Q793I8	Tifa	1.498	0.583	0.015	0.031
Q7TMM9	Tubb2a	4.262	2.092	0.001	0.005
Q7TMR0	Prcp	1.276	0.351	0.000	0.004
Q7TPN9	Prr14	1.252	0.325	0.000	0.004
Q80SZ7	Gng5	1.342	0.425	0.031	0.054
Q80TR8	Dcaf1	1.330	0.412	0.000	0.004
Q80UE5	Epb41l2	1.306	0.385	0.000	0.004
Q80UU9	Pgrmc2	1.234	0.303	0.000	0.004
Q80V42	Cpm	1.681	0.750	0.001	0.006
Q80VP1	Epn1	1.471	0.557	0.000	0.004
Q80X50	Ubap2l	1.284	0.361	0.000	0.004
Q80XN0	Bdh1	1.486	0.572	0.000	0.004
Q80XP8	Fam76b	1.225	0.293	0.000	0.003
Q80Y14	Glrx5	2.091	1.064	0.000	0.004
Q80Y44	Ddx10	1.292	0.370	0.000	0.003
Q80YA7	Dpp8	1.333	0.415	0.001	0.006
Q80ZM5	H1fx	1.593	0.672	0.001	0.005
Q810A3	Ttc9c	1.300	0.379	0.005	0.013
Q8BFP9	Pdk1	1.344	0.427	0.001	0.007
Q8BFR4	Gns	1.279	0.355	0.000	0.003
Q8BFR5	Tufm	1.225	0.293	0.000	0.004
Q8BGR9	Ublcp1	1.337	0.419	0.001	0.005
Q8BGS0	Mak16	1.283	0.359	0.007	0.018
Q8BGW1	Fto	1.253	0.325	0.004	0.013
Q8BH64	Ehd2	1.922	0.943	0.006	0.015
Q8BH75	Rnf41	1.275	0.350	0.008	0.020
Q8BHJ6	Serinc5	2.131	1.092	0.014	0.030
Q8BHJ9	Slu7	1.276	0.352	0.002	0.007
Q8BHN1	Txlng	1.204	0.268	0.018	0.036
Q8BHX3	Cdca8	1.295	0.373	0.000	0.002
Q8BIW9	Chtf18	1.241	0.311	0.003	0.011
Q8BIX3	Arl14ep	2.276	1.187	0.000	0.003
Q8BK35	Nop53	1.215	0.281	0.001	0.006

Q8BLF1	Nceh1	1.289	0.366	0.001	0.006
Q8BM39	Prpf18	1.209	0.274	0.003	0.011
Q8BMS1	Hadha	1.482	0.568	0.000	0.002
Q8BN51	Tk2	1.273	0.348	0.001	0.007
Q8BP40	Acp6	1.552	0.634	0.001	0.005
Q8BR75	Rsrc1	1.635	0.709	0.005	0.014
Q8BSE7	Clasp2	1.348	0.431	0.006	0.017
Q8BSM7	Slc43a1	1.968	0.976	0.002	0.009
Q8BTS3	Gbp9	1.525	0.609	0.028	0.050
Q8BTU1	Cfap20	1.207	0.272	0.014	0.030
Q8BTW3	Exosc6	2.482	1.312	0.001	0.005
Q8BTX9	Hsdl1	1.283	0.359	0.002	0.009
Q8BUH2	Nme7	1.301	0.380	0.005	0.014
Q8BV49	Pyhin1	1.446	0.532	0.003	0.009
Q8BVY0	Rsl1d1	1.252	0.324	0.005	0.014
Q8BXV2	Bri3bp	1.364	0.448	0.003	0.010
Q8BY71	Hat1	1.480	0.565	0.000	0.003
Q8BYI8	Fam234b	1.947	0.961	0.002	0.008
Q8BYW9	Eogt	1.308	0.387	0.007	0.017
Q8BZ09	Slc25a21	1.670	0.740	0.001	0.005
Q8BZQ2	Crispld2	1.201	0.264	0.042	0.068
Q8C166	Cpne1	1.254	0.327	0.002	0.008
Q8C253	Lgals3	1.543	0.626	0.002	0.009
Q8C4B4	Unc119b	1.598	0.676	0.001	0.005
Q8C4J7	Tbl3	1.202	0.266	0.002	0.009
Q8C6B0	Mettl7a1	1.364	0.448	0.004	0.013
Q8C6B9	Rps19bp1	1.248	0.320	0.022	0.042
Q8C804	Spice1	1.218	0.284	0.015	0.031
Q8CAA7	Pgm2l1	1.860	0.895	0.014	0.030
Q8CAQ8	Immt	1.258	0.331	0.001	0.006
Q8CB44	Gramd4	1.310	0.389	0.000	0.005
Q8CBB9	Rsad2	2.191	1.132	0.017	0.034
Q8CE50	Snx30	1.567	0.648	0.000	0.004
Q8CGA3	Slc43a2	1.492	0.578	0.001	0.006
Q8CGN4	Bcor	1.342	0.425	0.008	0.019
Q8CGP1	Hist1h2bk	1.677	0.746	0.001	0.006
Q8CGP6	Hist1h2ah	1.275	0.351	0.003	0.011
Q8CHH9	Septin8	1.340	0.422	0.021	0.040
Q8CI43	Myl6b	1.330	0.411	0.022	0.042
Q8CI78	Rmnd1	1.673	0.743	0.003	0.011
Q8K015	Cenpo	1.200	0.263	0.002	0.008
Q8K1R3	Pnpt1	1.346	0.428	0.001	0.007
Q8K274	Fn3krp	1.280	0.356	0.004	0.012
Q8K285	Fcho1	1.301	0.379	0.001	0.006
Q8K2B3	Sdha	1.230	0.299	0.024	0.044
Q8K2M0	Mrpl38	1.312	0.391	0.002	0.008
Q8K2T1	Nmral1	1.432	0.518	0.000	0.003
Q8K2T8	Paf1	1.205	0.269	0.001	0.006
Q8K2Y7	Mrpl47	1.202	0.266	0.001	0.007
Q8K411	Pitrm1	1.264	0.338	0.001	0.006
Q8K4F6	Nsun5	1.226	0.295	0.014	0.030
Q8K4L0	Ddx54	1.259	0.332	0.001	0.007
Q8K4Q6	Neil1	1.536	0.619	0.000	0.005
Q8QZT1	Acat1	1.674	0.744	0.000	0.004
Q8R0F5	Rbmx2	1.277	0.353	0.004	0.013
Q8R0F8	Fahd1	1.352	0.436	0.007	0.018
Q8R1F0	D8Ertd738e	1.361	0.444	0.009	0.022
Q8R1G5	Mthfsl	1.252	0.324	0.002	0.009
Q8R2M2	Dnttip2	1.297	0.375	0.000	0.003
Q8R323	Rfc3	1.228	0.297	0.004	0.012
Q8R326	Pspc1	1.208	0.272	0.042	0.070

Q8R3G9	Tspan8	1.343	0.425	0.010	0.023
Q8R3S2	Tspan33	1.708	0.772	0.005	0.014
Q8R4N0	Clybl	1.235	0.305	0.009	0.020
Q8R502	Lrrc8c	1.238	0.308	0.000	0.004
Q8R574	Prpsap2	1.438	0.524	0.000	0.004
Q8R5C5	Actr1b	1.209	0.274	0.001	0.006
Q8R5L1	C1qbp	1.274	0.349	0.029	0.052
Q8VBT0	Tmx1	1.216	0.283	0.000	0.003
Q8VBZ3	Clptm1	1.264	0.338	0.001	0.007
Q8VCF0	Mavs	1.496	0.581	0.000	0.003
Q8VCW8	Acsf2	1.542	0.625	0.001	0.005
Q8VD62	Bles03	1.216	0.283	0.004	0.012
Q8VDF2	Uhrf1	1.509	0.593	0.001	0.006
Q8VDP2		1.357	0.440	0.002	0.008
Q8VDU0	Gpsm2	1.389	0.474	0.000	0.003
Q8VDV8	Mitd1	1.588	0.667	0.007	0.017
Q8VDW0	Ddx39a	1.472	0.557	0.000	0.005
Q8VE19	Mios	1.280	0.356	0.002	0.009
Q8VEE4	Rpa1	1.372	0.457	0.002	0.008
Q8VEK3	Hnrnpu	1.239	0.310	0.000	0.005
Q8VEM8	Slc25a3	1.409	0.495	0.000	0.003
Q8VH51	Rbm39	1.232	0.301	0.004	0.013
Q8VHL0	Slc14a1	1.300	0.379	0.047	0.076
Q8VHM5	Hnrnpr	1.241	0.312	0.005	0.013
Q8VHZ7	Imp4	1.427	0.513	0.000	0.004
Q91V08	Clec2d	1.246	0.317	0.032	0.055
Q91V89	Ppp2r5d	1.220	0.286	0.001	0.005
Q91VA6	Poldip2	1.215	0.281	0.001	0.006
Q91VA7	Idh3b	1.332	0.413	0.000	0.003
Q91VB8	Hba-a1	2.821	1.496	0.005	0.015
Q91VC3	Eif4a3	1.208	0.272	0.001	0.005
Q91VE6	Nifk	1.257	0.330	0.001	0.007
Q91VL8	Terf2ip	1.228	0.297	0.002	0.008
Q91VM5	Rbmxl1	1.459	0.545	0.000	0.004
Q91VT4	Cbr4	1.311	0.391	0.025	0.045
Q91W53	Golga7	1.231	0.299	0.002	0.007
Q91WM1	Strbp	1.429	0.515	0.001	0.006
Q91WN1	Dnajc9	1.229	0.297	0.002	0.008
Q91WR3	Ascc2	1.902	0.927	0.000	0.003
Q91YJ3	Thyn1	1.263	0.336	0.001	0.005
Q91YJ5	Mtif2	1.315	0.395	0.002	0.008
Q91YN9	Bag2	1.318	0.398	0.002	0.009
Q91YY4	Atpaf2	1.305	0.384	0.000	0.004
Q91Z92	B3galt6	1.280	0.356	0.005	0.015
Q91ZA3	Pcca	1.291	0.368	0.001	0.006
Q920A9	Fcrla	1.806	0.853	0.001	0.005
Q920G8	Slc25a37	1.681	0.750	0.001	0.006
Q920Q6	Msi2	1.532	0.616	0.033	0.056
Q921I1	Tf	1.431	0.518	0.043	0.070
Q921J2	Rheb	1.377	0.462	0.001	0.005
Q921K2	Parp1	1.480	0.566	0.000	0.004
Q921N6	Ddx27	1.226	0.294	0.001	0.005
Q921W0	Chmp1a	1.262	0.336	0.002	0.009
Q921Y2	Imp3	1.207	0.272	0.002	0.009
Q922D8	Mthfd1	1.320	0.401	0.000	0.004
Q922H2	Pdk3	1.203	0.266	0.001	0.007
Q922Q1	Marc2	1.262	0.336	0.000	0.004
Q922Q2	Riok1	1.201	0.264	0.010	0.023
Q922R5	Ppp4r3b	1.855	0.892	0.003	0.010
Q922U1	Prpf3	1.206	0.271	0.000	0.003
Q923D2	Blvrb	1.609	0.686	0.000	0.003

Q924Z4	Cers2	1.276	0.352	0.018	0.035
Q99020	Hnrnpab	1.436	0.522	0.000	0.003
Q99J36	Thumpd1	1.281	0.358	0.002	0.009
Q99J62	Rfc4	1.623	0.699	0.000	0.003
Q99J83	Atg5	1.334	0.416	0.002	0.008
Q99J95	Cdk9	1.301	0.379	0.001	0.006
Q99J99	Mpst	1.432	0.518	0.000	0.003
Q99JF8	Psip1	1.316	0.396	0.000	0.004
Q99JR1	Sfxn1	1.243	0.314	0.001	0.005
Q99JT9	Adi1	1.274	0.349	0.006	0.016
Q99JY0	Hadhb	1.621	0.697	0.000	0.003
Q99KK9	Hars2	1.638	0.712	0.000	0.004
Q99KR3	Lactb2	1.228	0.296	0.020	0.039
Q99L47	St13	1.205	0.269	0.002	0.009
Q99LC3	Ndufa10	1.324	0.405	0.001	0.005
Q99LC5	Etfa	1.217	0.283	0.000	0.003
Q99LD8	Ddah2	1.632	0.706	0.001	0.006
Q99LI7	Cstf3	1.339	0.421	0.000	0.004
Q99LM9	Tada1	1.217	0.283	0.001	0.006
Q99M28	Rnps1	1.282	0.358	0.002	0.009
Q99M71	Epdr1	1.737	0.797	0.003	0.010
Q99MN9	Pccb	1.300	0.378	0.009	0.022
Q99N96	Mrpl1	1.238	0.308	0.001	0.007
Q99NB1	Acss1	1.620	0.696	0.001	0.006
Q99P51	Rassf3	1.372	0.456	0.002	0.009
Q9CPP6	Ndufa5	1.775	0.828	0.001	0.007
Q9CPQ3	Tomm22	1.251	0.323	0.013	0.028
Q9CPQ8	Atp5mg	1.274	0.349	0.002	0.009
Q9CPT3	Nanp	1.470	0.556	0.001	0.007
Q9CPU4	Mgst3	2.097	1.068	0.008	0.019
Q9CPX4	Ftl1	2.904	1.538	0.000	0.003
Q9CQ36	Pole4	1.476	0.561	0.000	0.004
Q9CQ43	Dut	1.263	0.337	0.014	0.029
Q9CQ45	Nenf	1.363	0.447	0.003	0.010
Q9CQ71	Rpa3	1.836	0.877	0.001	0.006
Q9CQB4	Uqcrb	1.352	0.435	0.000	0.005
Q9CQC7	Ndufb4	1.694	0.761	0.000	0.003
Q9CQD1	Rab5a	1.236	0.306	0.000	0.005
Q9CQE6	Asf1a	1.207	0.272	0.002	0.008
Q9CQF3	Nudt21	1.231	0.300	0.003	0.011
Q9CQH0	Pdzk1ip1	1.578	0.659	0.004	0.012
Q9CQL1	Magohb	1.248	0.320	0.000	0.003
Q9CQL7	Mrfap1	1.274	0.349	0.001	0.005
Q9CQN6	Tmem14c	1.335	0.416	0.003	0.010
Q9CQN7	Mrpl41	1.216	0.282	0.000	0.004
Q9CQR4	Acot13	1.222	0.289	0.022	0.042
Q9CQS8	Sec61b	2.856	1.514	0.000	0.004
Q9CQV8	Ywhab	1.218	0.284	0.001	0.007
Q9CQX4	Pclaf	1.208	0.273	0.003	0.011
Q9CR24	Nudt8	1.421	0.507	0.005	0.013
Q9CRB9	Chchd3	1.394	0.479	0.000	0.003
Q9CRD0	Ociad1	1.294	0.372	0.000	0.004
Q9CS42	Prps2	1.335	0.417	0.000	0.004
Q9CU65	Zmym2	1.400	0.486	0.001	0.005
Q9CUX1	Thap12	1.214	0.280	0.002	0.008
Q9CVB6	Arpc2	1.271	0.346	0.000	0.005
Q9CWK8	Snx2	1.268	0.343	0.001	0.005
Q9CWX9	Ddx47	1.326	0.407	0.000	0.003
Q9CX60	Lbh	1.248	0.320	0.006	0.016
Q9CX86	Hnrnpa0	1.201	0.264	0.011	0.026
Q9CXE2	Bcl7a	1.363	0.447	0.000	0.003

Q9CXI0	Coq5	1.201	0.264	0.020	0.038
Q9CXR1	Dhrs7	1.265	0.339	0.003	0.011
Q9CXS4	Cenpv	1.242	0.313	0.000	0.004
Q9CXZ1	Ndufs4	1.359	0.443	0.000	0.004
Q9CY02	Ahsp	3.203	1.680	0.006	0.016
Q9CY64	Blvra	1.287	0.364	0.000	0.003
Q9CY97	Ssu72	1.556	0.638	0.000	0.004
Q9CYL5	Glipr2	1.236	0.306	0.009	0.020
Q9CZ30	Ola1	1.206	0.270	0.000	0.005
Q9CZP5	Bcs1l	1.469	0.554	0.000	0.004
Q9D051	Pdhb	1.420	0.506	0.001	0.006
Q9D0B0	Srsf9	1.203	0.267	0.000	0.002
Q9D0M1	Prpsap1	1.224	0.292	0.005	0.015
Q9D0S9	Hint2	1.600	0.678	0.000	0.004
Q9D115	Znf706	1.439	0.525	0.000	0.004
Q9D198	Syf2	1.324	0.405	0.017	0.034
Q9D1C9	Rrp7a	1.383	0.468	0.002	0.008
Q9D1F4	Akt1s1	1.298	0.376	0.003	0.010
Q9D1W4	030006K11R	1.370	0.454	0.002	0.008
Q9D287	Bcas2	1.294	0.372	0.001	0.005
Q9D2G2	Dlst	1.232	0.301	0.006	0.015
Q9D2R6	Coa3	1.969	0.977	0.000	0.003
Q9D2U9	Hist3h2ba	1.876	0.908	0.001	0.005
Q9D338	Mrpl19	1.317	0.397	0.000	0.003
Q9D3Q7	Epsti1	1.410	0.495	0.043	0.070
Q9D5T0	Atad1	1.348	0.431	0.001	0.006
Q9D6K8	Fundc2	1.715	0.778	0.000	0.003
Q9D6T0	Nosip	1.268	0.342	0.004	0.012
Q9D6V8	Paip2	1.470	0.555	0.001	0.005
Q9D7E4		1.369	0.453	0.011	0.024
Q9D7S7	Rpl22l1	1.491	0.577	0.002	0.008
Q9D818	Sapcd2	1.283	0.360	0.001	0.006
Q9D883	U2af1	1.207	0.272	0.000	0.005
Q9D898	Arpc5l	1.419	0.505	0.001	0.005
Q9D8U8	Snx5	1.279	0.355	0.000	0.002
Q9D903	Ebna1bp2	1.202	0.266	0.000	0.004
Q9D920	Borcs5	1.377	0.461	0.000	0.004
Q9D967	Mdp1	1.344	0.426	0.002	0.009
Q9DB20	Atp5po	1.332	0.413	0.001	0.005
Q9DB30	Phkg2	1.312	0.392	0.006	0.017
Q9DB60	Prxl2b	1.349	0.431	0.001	0.006
Q9DB77	Uqcrc2	1.668	0.738	0.000	0.004
Q9DB96	Ngdn	1.453	0.539	0.008	0.019
Q9DBM2	Ehhadh	1.204	0.268	0.001	0.006
Q9DBR7	Ppp1r12a	1.253	0.325	0.003	0.010
Q9DBY8	Nvl	1.287	0.364	0.000	0.003
Q9DC29	Abcb6	1.253	0.326	0.038	0.064
Q9DCC5	Cbx3	1.612	0.689	0.002	0.008
Q9DCH4	Eif3f	1.318	0.398	0.002	0.008
Q9DCN2	Cyb5r3	1.709	0.773	0.002	0.008
Q9DCT2	Ndufs3	1.227	0.295	0.002	0.008
Q9DCX2	Atp5pd	1.932	0.950	0.000	0.005
Q9DCZ1	Gmpr	1.432	0.518	0.049	0.078
Q9EQ20	Aldh6a1	1.412	0.498	0.003	0.010
Q9EQF2	Kel	1.779	0.831	0.002	0.009
Q9EQI8	Mrpl46	1.235	0.305	0.004	0.013
Q9ERH4	Nusap1	1.319	0.399	0.000	0.004
Q9ERN0	Scamp2	1.632	0.706	0.000	0.004
Q9ERS2	Ndufa13	1.609	0.686	0.001	0.007
Q9ERZ0	Hemgn	2.138	1.096	0.001	0.005
Q9ESU7	Slc1a5	1.295	0.373	0.001	0.006

Q9ESW4	Agk	1.466	0.552	0.001	0.005
Q9ESX5	Dkc1	1.243	0.314	0.004	0.012
Q9JHI9	Slc40a1	1.778	0.830	0.000	0.003
Q9JHK5	Plek	1.406	0.492	0.004	0.012
Q9JI13	Utp3	1.484	0.570	0.000	0.003
Q9JI39	Abcb10	1.527	0.610	0.001	0.006
Q9JIK5	Ddx21	1.277	0.352	0.000	0.003
Q9JIQ3	Diablo	1.335	0.417	0.004	0.013
Q9JIW4	Polm	2.056	1.040	0.001	0.007
Q9JJZ4	Ube2j1	1.352	0.435	0.020	0.039
Q9JK30	Orc3	1.293	0.371	0.001	0.006
Q9JK48	Sh3glb1	1.264	0.338	0.001	0.005
Q9JL60	Gmeb1	1.640	0.714	0.000	0.004
Q9JL61	Rfx5	1.256	0.329	0.013	0.029
Q9QUR7	Pin1	1.580	0.660	0.001	0.005
Q9QXV1	Cbx8	1.431	0.517	0.027	0.049
Q9QXW0	Fbxl6	1.249	0.321	0.000	0.004
Q9QXY6	Ehd3	1.676	0.745	0.014	0.029
Q9QY81	Nup210	1.258	0.331	0.003	0.010
Q9QYB8	Add2	1.626	0.701	0.000	0.005
Q9QYR9	Acot2	1.507	0.591	0.000	0.004
Q9QZI9	Serinc3	1.690	0.757	0.002	0.008
Q9R078	Prkab1	1.234	0.304	0.018	0.036
Q9R0P4	Smap	1.287	0.364	0.002	0.008
Q9R0P9	Uchl1	3.825	1.936	0.001	0.006
Q9R1T4	Septin6	1.276	0.351	0.007	0.018
Q9WTI7	Myo1c	1.272	0.347	0.000	0.003
Q9WTM5	Ruvbl2	1.235	0.304	0.000	0.005
Q9WTP7	Ak3	1.646	0.719	0.000	0.004
Q9WTQ5	Akap12	2.234	1.159	0.001	0.007
Q9WTZ0	Uxt	1.833	0.874	0.001	0.007
Q9WU81	Slc37a2	1.369	0.453	0.008	0.020
Q9WUK2	Eif4h	1.520	0.604	0.000	0.004
Q9WUV0	Orc5	1.403	0.488	0.003	0.011
Q9WV03	Fam50a	1.225	0.293	0.001	0.006
Q9WV69	Dmtn	1.576	0.657	0.003	0.010
Q9WV98	Timm9	1.249	0.321	0.000	0.003
Q9WVA2	Timm8a1	1.257	0.330	0.000	0.003
Q9WVA4	Tagln2	1.225	0.292	0.014	0.030
Q9WVJ3	Cpq	1.306	0.385	0.001	0.006
Q9Z0R4	Itsn1	1.389	0.475	0.001	0.006
Q9Z0S9	Rabac1	1.246	0.317	0.001	0.005
Q9Z103	Adnp	1.360	0.444	0.001	0.007
Q9Z127	Slc7a5	1.307	0.386	0.000	0.004
Q9Z1N5	Ddx39b	1.444	0.530	0.000	0.005
Q9Z204	Hnrnpc	1.267	0.341	0.001	0.005
Q9Z2E1	Mbd2	1.221	0.288	0.001	0.006
Q9Z2W1	Stk25	1.307	0.387	0.005	0.014
S4R2E1	Abcg2	1.404	0.490	0.003	0.010
S4R2G9	Akr1b10	1.302	0.380	0.000	0.004
V9GWS2	Slc25a22	1.263	0.336	0.005	0.014
Z4YL55	Cdan1	1.340	0.422	0.000	0.004
A0A075B5K2	Igkv9-124	0.291	(1.778)	0.000	0.004
A0A075B5N7	Igkv6-13	0.458	(1.125)	0.001	0.005
A0A075B5P3	Ighg2b	0.307	(1.703)	0.001	0.005
A0A075B5P4	Ighg1	0.173	(2.531)	0.000	0.004
A0A075B5R7	Ighv14-2	0.241	(2.051)	0.000	0.005
A0A075B5T5	Ighv1-5	0.416	(1.264)	0.009	0.022
A0A075B5V7	Ighv1-43	0.269	(1.892)	0.001	0.005
A0A087WNR6	Atg4b	0.684	(0.549)	0.000	0.003
A0A087WP69	Dstyk	0.814	(0.297)	0.002	0.007

A0A087WP87	Rnf2	0.819	(0.288)	0.031	0.054
A0A087WQT0	Stk16	0.704	(0.506)	0.000	0.004
A0A087WS03	Pign	0.737	(0.441)	0.005	0.014
A0A0A0MQP1	Pkd1l1	0.569	(0.814)	0.000	0.004
A0A0A6YVT4	Pcdhgc5	0.580	(0.787)	0.002	0.008
A0A0A6YW92	Dalrd3	0.705	(0.504)	0.002	0.008
A0A0A6YWF8	Sirpb1b	0.809	(0.306)	0.002	0.009
A0A0A6YX21	Sft2d2	0.830	(0.270)	0.005	0.014
A0A0A6YXM0	Tor4a	0.767	(0.383)	0.001	0.005
A0A0A6YXN4	Ighv1-18	0.381	(1.393)	0.000	0.005
A0A0A6YXW6	Igha	0.160	(2.647)	0.001	0.006
A0A0A6YY53	Ighg2c	0.165	(2.595)	0.001	0.005
A0A0B4J1G0	Fcgr4	0.782	(0.355)	0.002	0.007
A0A0B4J1H7	Igkv1-135	0.431	(1.215)	0.047	0.076
A0A0B4J1J7	Ighv1-82	0.334	(1.583)	0.001	0.007
A0A0G2JDF3	Mapkapk5	0.695	(0.526)	0.000	0.002
A0A0G2JDM4	Abcb4	0.734	(0.446)	0.004	0.012
A0A0G2JE90	Prom1	0.623	(0.682)	0.004	0.012
A0A0G2JEN8	Tfip11	0.592	(0.757)	0.000	0.004
A0A0G2JET2	Camkk2	0.662	(0.594)	0.000	0.004
A0A0G2JGD2	S100a4	0.800	(0.322)	0.006	0.017
A0A0J9YTZ0	330009J07R	0.826	(0.277)	0.001	0.005
A0A0J9YUX0	Fxyd5	0.767	(0.382)	0.019	0.037
A0A0J9YV30	Pxn	0.581	(0.784)	0.000	0.003
A0A0J9YVE6	Mgam	0.679	(0.558)	0.004	0.013
A0A0N4SVH4	Arhgdib	0.407	(1.296)	0.000	0.005
A0A0N4SVL8	Mmrn1	0.797	(0.327)	0.030	0.052
A0A0N4SVR0	Scrn1	0.645	(0.632)	0.014	0.029
A0A0N4SVV6	Rasgrp4	0.725	(0.464)	0.000	0.004
A0A0N4SWB0	Casd1	0.703	(0.508)	0.001	0.006
A0A0R4IZW8	Capns1	0.822	(0.283)	0.001	0.006
A0A0R4J034	Pdxdc1	0.765	(0.386)	0.001	0.007
A0A0R4J0D0	Snx20	0.793	(0.335)	0.002	0.007
A0A0R4J0N9	Mtus1	0.669	(0.581)	0.000	0.004
A0A0R4J0P5	Pstpip1	0.808	(0.307)	0.000	0.005
A0A0R4J0T2	Clec12a	0.823	(0.282)	0.006	0.015
A0A0R4J0U5	Abcd2	0.808	(0.307)	0.005	0.013
A0A0R4J0Y3	Helz	0.758	(0.399)	0.000	0.003
A0A0R4J100	Gpr84	0.771	(0.376)	0.000	0.004
A0A0R4J138	Arsb	0.823	(0.282)	0.002	0.008
A0A0R4J1J1	Pnkd	0.824	(0.280)	0.001	0.007
A0A0R4J1T9	Prodh	0.684	(0.547)	0.001	0.006
A0A0R4J1Z3	Tmem33	0.747	(0.420)	0.026	0.047
A0A0R4J256	Psmb9	0.825	(0.278)	0.003	0.010
A0A0R4J269	Clec5a	0.744	(0.427)	0.000	0.004
A0A0U1RQ24	Ceacam10	0.733	(0.448)	0.001	0.007
A0A0U1RQ27	Mcee	0.819	(0.288)	0.000	0.005
A0A140LIW3	Frmpd3	0.729	(0.456)	0.001	0.006
A0A140LJ55	Slc9a6	0.723	(0.468)	0.008	0.020
A0A140LJ78	Wdr45	0.776	(0.365)	0.014	0.029
A0A140LJK6	930438A08R	0.789	(0.341)	0.007	0.019
A0A1B0GR63	Chmp2a	0.796	(0.329)	0.000	0.003
A0A1B0GS90	Aktip	0.739	(0.435)	0.016	0.033
A0A1B0GSA5	Ddhd2	0.822	(0.283)	0.003	0.011
A0A1B0GSH5	Chid1	0.755	(0.405)	0.005	0.013
A0A1B0GSX0	Ldha	0.824	(0.279)	0.007	0.018
A0A1C7CYV0	Pls3	0.784	(0.352)	0.004	0.012
A0A1D5RLZ8	Cracr2a	0.778	(0.361)	0.001	0.007
A0A1D5RMB5	Dnmbp	0.818	(0.290)	0.000	0.004
A0A1L1SQV7	Pafah1b2	0.802	(0.319)	0.007	0.017
A0A1L1SRR1	Cmtm7	0.799	(0.324)	0.011	0.025

A0A1L1SSA8	Tmem205	0.778	(0.363)	0.002	0.007
A0A1L1STC6	Syne1	0.823	(0.281)	0.000	0.003
A0A1L1SUT0	Acpp	0.650	(0.622)	0.001	0.007
A0A1W2P6K9	Sgpp1	0.747	(0.421)	0.005	0.014
A0A1W2P6U8	932415D10R	0.828	(0.273)	0.009	0.022
A0A1W2P780	Csrp2	0.811	(0.303)	0.001	0.006
A0A1W2P788	Man1a	0.755	(0.406)	0.001	0.005
A0A1W2P793	Gja1	0.812	(0.300)	0.002	0.009
A0A1W2P8C6	Cd63	0.734	(0.445)	0.001	0.005
A0A1Y7VIR0	Ppp2r5c	0.745	(0.424)	0.001	0.007
A0A1Y7VNC5	Nin	0.756	(0.404)	0.000	0.002
A0A286YCZ3	Myof	0.727	(0.460)	0.002	0.009
A0A286YDV6	Slc30a5	0.462	(1.115)	0.001	0.006
A0A286YE23	Il3ra	0.775	(0.367)	0.002	0.008
A0A2I3BPG9	Rpl36a-ps1	0.775	(0.367)	0.012	0.027
A0A2I3BQ03	Ywhaz	0.615	(0.703)	0.000	0.004
A0A2I3BQ98	Kbtbd6	0.717	(0.480)	0.000	0.004
A0A2I3BQE0	Stk3	0.829	(0.271)	0.000	0.003
A0A2I3BR03	App	0.585	(0.774)	0.003	0.010
A0A2I3BR95	Ksr1	0.741	(0.433)	0.001	0.006
A0A2R8VHK6	Smdt1	0.774	(0.369)	0.002	0.008
A0A338P686	Lztr1	0.806	(0.311)	0.001	0.006
A0A338P6P7	Pacsin2	0.402	(1.316)	0.001	0.005
A0A338P7E3	Rnaset2b	0.806	(0.311)	0.013	0.028
A0A3B2WB50	Emilin2	0.590	(0.762)	0.001	0.007
A0A494B923	Papss2	0.783	(0.352)	0.002	0.007
A0A494BA00	Ska1	0.800	(0.323)	0.000	0.003
A0A494BB02	Capn1	0.493	(1.022)	0.000	0.004
A0A494BBJ5	Malt1	0.800	(0.323)	0.006	0.016
A1E5L0	Cytl1	0.485	(1.043)	0.001	0.005
A2A6H1	Lasp1	0.823	(0.280)	0.001	0.007
A2A6Z2	Cd300lf	0.611	(0.710)	0.006	0.016
A2ABV1	Phf8	0.546	(0.874)	0.012	0.027
A2AE95	Sytl1	0.784	(0.352)	0.009	0.021
A2AGE0	Tesk2	0.740	(0.435)	0.001	0.006
A2AH75	Kif1b	0.731	(0.453)	0.000	0.003
A2AI57	Trpm7	0.825	(0.277)	0.026	0.047
A2AIV8	Card9	0.685	(0.545)	0.002	0.007
A2AJK6	Chd7	0.687	(0.541)	0.002	0.008
A2AJW4	Ppp1r3d	0.654	(0.613)	0.002	0.008
A2AK37	AA467197	0.620	(0.689)	0.000	0.003
A2AMS3	Galt	0.799	(0.324)	0.008	0.020
A2AQW0	Map3k15	0.742	(0.431)	0.001	0.006
A2AR78	Tank	0.811	(0.303)	0.003	0.010
A2ARF6	Ak4	0.750	(0.414)	0.000	0.003
A2AVJ7	Rrbp1	0.793	(0.335)	0.002	0.007
A2AWR5	Gm14085	0.576	(0.795)	0.000	0.003
A2CF65	Clec10a	0.540	(0.890)	0.001	0.007
A2RSJ4	Uhrf1bp1l	0.723	(0.468)	0.002	0.009
A3KGB4	Tbc1d8b	0.800	(0.322)	0.001	0.007
A6PWC3	Nrd1	0.833	(0.264)	0.000	0.004
A6XDB1	Pde4d	0.760	(0.397)	0.007	0.018
A8Y5H7	Sec14l1	0.685	(0.545)	0.004	0.013
A9C471	Naip2	0.826	(0.276)	0.003	0.011
B1ASZ3	Gk	0.778	(0.361)	0.006	0.017
B1AT64	Wipi1	0.818	(0.291)	0.004	0.013
B1ATD5	Mtmr3	0.792	(0.337)	0.001	0.006
B1AVH7	Tbc1d2	0.689	(0.538)	0.001	0.005
B1AVI2	Stx17	0.766	(0.385)	0.000	0.003
B1AXH1	Nhsl2	0.793	(0.334)	0.000	0.004
B1AXY5	B4galt1	0.744	(0.428)	0.000	0.004

B1AZF9	Aatk	0.746	(0.423)	0.000	0.003
B2RRC9	F8	0.577	(0.794)	0.001	0.006
B2RUP2	Unc13d	0.812	(0.301)	0.000	0.004
B2RV77	Cstdc4	0.792	(0.336)	0.010	0.023
B2RXR6	Ankrd44	0.816	(0.294)	0.000	0.004
B5THE2	Mgam	0.656	(0.609)	0.020	0.038
B7ZWH4	Pilrb1	0.809	(0.306)	0.001	0.005
B9EHT6	Fn1	0.729	(0.456)	0.003	0.010
B9EJ80	Pdzd8	0.806	(0.312)	0.000	0.003
B9EKI3	Tmf1	0.816	(0.294)	0.001	0.005
D3YTS8	Slc12a8	0.592	(0.756)	0.003	0.011
D3YTY9	Kng1	0.798	(0.325)	0.014	0.030
D3YUG5	Mrtfa	0.821	(0.285)	0.001	0.005
D3YVW2	Golim4	0.745	(0.424)	0.000	0.004
D3YWB8	Rps6kb2	0.811	(0.301)	0.000	0.003
D3YWS5	Gadl1	0.597	(0.744)	0.005	0.014
D3YX52	Tm6sf1	0.818	(0.289)	0.001	0.005
D3YZ98	Pik3c3	0.465	(1.106)	0.006	0.016
D3Z136	Elovl5	0.575	(0.797)	0.020	0.038
D3Z286	Tmppe	0.724	(0.466)	0.006	0.017
D3Z2W9	Ntpcr	0.754	(0.406)	0.003	0.011
D3Z2X8	Zbtb7b	0.639	(0.646)	0.007	0.018
D3Z534	Kalrn	0.607	(0.720)	0.000	0.004
D3Z6X3	Pex3	0.587	(0.768)	0.023	0.043
D3Z7B5	Cip2a	0.814	(0.297)	0.009	0.021
D3Z7F7	Cpped1	0.816	(0.294)	0.001	0.006
D6REI4	L3mbtl4	0.424	(1.237)	0.007	0.017
D6REU0	Mindy3	0.770	(0.378)	0.008	0.020
D6RFU9	Sypl	0.746	(0.423)	0.001	0.006
E0CY07	Tmem167	0.779	(0.361)	0.002	0.007
E0CZE8	Tmbim6	0.829	(0.270)	0.021	0.040
E9PUQ5	Golga2	0.811	(0.302)	0.000	0.005
E9PUQ9	Piezo1	0.688	(0.539)	0.005	0.014
E9PUV2	Fcgr3	0.642	(0.639)	0.000	0.003
E9PW38	Mctp1	0.833	(0.264)	0.001	0.005
E9PWG9	Map3k5	0.828	(0.272)	0.002	0.007
E9PYL6	Ikbke	0.667	(0.585)	0.001	0.007
E9PZY1	Hps3	0.685	(0.546)	0.001	0.006
E9Q0A3	Arhgef11	0.748	(0.420)	0.002	0.009
E9Q0I8	Homer1	0.817	(0.291)	0.002	0.008
E9Q2I3	Ppm1m	0.807	(0.310)	0.000	0.004
E9Q330	Pla2g7	0.645	(0.633)	0.008	0.020
E9Q359	Adam8	0.722	(0.469)	0.000	0.003
E9Q3U1	Ube2h	0.485	(1.044)	0.001	0.006
E9Q3X0	Mvp	0.745	(0.424)	0.001	0.006
E9Q3Z5	Svil	0.826	(0.276)	0.000	0.004
E9Q4G8	Alcam	0.797	(0.328)	0.004	0.012
E9Q512	Trip11	0.749	(0.417)	0.000	0.003
E9Q5F4	Actb	0.725	(0.463)	0.002	0.007
E9Q5R6	Arhgef37	0.691	(0.534)	0.003	0.010
E9Q605	Chuk	0.745	(0.424)	0.003	0.010
E9Q616	Ahnak	0.778	(0.362)	0.001	0.006
E9Q748	Slpi	0.818	(0.290)	0.021	0.040
E9Q7G1	Tmed7	0.799	(0.323)	0.002	0.009
E9Q7P0	Dnah17	0.539	(0.892)	0.000	0.004
E9Q8I9	Fry	0.794	(0.334)	0.001	0.007
E9Q8N1	Ttn	0.630	(0.666)	0.006	0.015
E9Q9B8	Clu	0.765	(0.386)	0.007	0.017
E9Q9J4	Ppip5k2	0.825	(0.277)	0.001	0.005
E9QKG2	Katna1	0.763	(0.390)	0.001	0.007
E9QKK1	Cenpe	0.793	(0.334)	0.002	0.007

E9QLJ3	Med13l	0.772	(0.374)	0.003	0.010
E9QLK3	Brat1	0.776	(0.366)	0.004	0.012
E9QNE5	Pi16	0.717	(0.481)	0.000	0.004
E9QNG1	Itsn2	0.833	(0.264)	0.000	0.003
E9QNL8	Itgal	0.757	(0.402)	0.005	0.015
E9QNX9	Igf1r	0.704	(0.506)	0.001	0.007
E9QP46	Syne2	0.827	(0.274)	0.000	0.005
F2Z409	Dgkg	0.687	(0.541)	0.000	0.004
F2Z427	Pgs1	0.712	(0.489)	0.002	0.009
F6T836	Arhgap26	0.704	(0.507)	0.004	0.011
F6TB64	Ubn2	0.809	(0.305)	0.001	0.005
F6TQF4	Irak1	0.693	(0.529)	0.002	0.008
F6U5V1	Larp1b	0.616	(0.699)	0.001	0.005
F6UND7	Hck	0.753	(0.410)	0.006	0.017
F6V3Y9	Atg2a	0.816	(0.293)	0.001	0.005
F6VFS9	Gucy2d	0.414	(1.271)	0.001	0.006
F6VKM0	Trappc6a	0.824	(0.280)	0.001	0.007
F6WL90	Sp100	0.812	(0.300)	0.021	0.040
F6X6A4	Cyp4b1	0.832	(0.265)	0.006	0.017
F6ZCY7	C2	0.372	(1.425)	0.002	0.007
F6ZLN8	Vps8	0.807	(0.309)	0.002	0.008
F7AZP7	Wdr45b	0.819	(0.287)	0.046	0.075
F7BJ98	Nbeal1	0.531	(0.912)	0.027	0.049
F7CES2	Plekhg1	0.744	(0.426)	0.001	0.005
F8VPK0	Ttc37	0.819	(0.289)	0.000	0.004
F8VPK6	Xylt1	0.702	(0.511)	0.000	0.004
F8VPN4	Agl	0.761	(0.395)	0.000	0.003
F8VQM5	Sis	0.639	(0.646)	0.005	0.015
F8WI14	Ecm1	0.809	(0.305)	0.006	0.016
G3UWY9	Tbc1d14	0.621	(0.687)	0.004	0.011
G3UX13	Snx14	0.772	(0.374)	0.026	0.047
G3UX23	Gne	0.833	(0.264)	0.000	0.004
G3UY45	Kmt2c	0.612	(0.708)	0.047	0.076
G3UY52	Usp9x	0.771	(0.375)	0.005	0.014
G3UYC5	Chek1	0.825	(0.278)	0.001	0.006
G3UYS3	Mettl14	0.683	(0.550)	0.011	0.024
G3X9K3	Arfgef1	0.795	(0.331)	0.000	0.002
G5E884	Pak1	0.765	(0.386)	0.000	0.004
G5E8Q0	Lyst	0.756	(0.404)	0.000	0.004
H3BJ49	Cers5	0.736	(0.442)	0.001	0.007
H3BJR8	Foxp1	0.816	(0.294)	0.036	0.061
H3BJS9	Hnrnpk	0.555	(0.850)	0.001	0.005
H3BKD4	Asap1	0.823	(0.281)	0.003	0.010
H7BWY4	Dlg1	0.787	(0.345)	0.023	0.043
I1E4X8	Stim2	0.814	(0.297)	0.001	0.005
J3QNH8	Gm7694	0.568	(0.815)	0.001	0.007
J3QPY0	600014C10R	0.590	(0.762)	0.004	0.011
J3QQ55	Tiam1	0.765	(0.386)	0.001	0.007
O08692	Ngp	0.797	(0.327)	0.001	0.005
O08908	Pik3r2	0.765	(0.386)	0.000	0.004
O35298	Aoah	0.770	(0.377)	0.001	0.006
O35379	Abcc1	0.809	(0.305)	0.004	0.012
O35627	Nr1i3	0.540	(0.890)	0.002	0.008
O35657	Neu1	0.824	(0.279)	0.003	0.010
O35664	Ifnar2	0.804	(0.316)	0.001	0.007
O35682	Myadm	0.632	(0.662)	0.001	0.005
O35704	Sptlc1	0.811	(0.301)	0.016	0.033
O55229	Chkb	0.812	(0.300)	0.001	0.007
O70274	Ptp4a2	0.823	(0.281)	0.000	0.003
O70370	Ctss	0.504	(0.988)	0.005	0.014
O70480	Vamp4	0.803	(0.317)	0.012	0.027

O70566	Diaph2	0.780	(0.359)	0.000	0.004
O88307	Sorl1	0.778	(0.362)	0.008	0.020
O88593	Pglyrp1	0.730	(0.454)	0.000	0.003
O88783	F5	0.830	(0.269)	0.000	0.003
O88792	F11r	0.726	(0.463)	0.004	0.011
O88855	Ltb4r	0.817	(0.291)	0.000	0.004
O88856	Tpst2	0.814	(0.297)	0.003	0.010
O89001	Cpd	0.712	(0.490)	0.003	0.010
P01592	Jchain	0.211	(2.244)	0.005	0.013
P01647		0.251	(1.996)	0.002	0.007
P01754	Ighv1-62-3	0.320	(1.644)	0.000	0.004
P01837	Igkc	0.363	(1.463)	0.005	0.014
P03958	Ada	0.655	(0.610)	0.003	0.010
P06745	Gpi	0.739	(0.436)	0.003	0.010
P06869	Plau	0.690	(0.535)	0.000	0.004
P07356	Anxa2	0.815	(0.295)	0.003	0.011
P08003	Pdia4	0.810	(0.303)	0.004	0.012
P08101	Fcgr2	0.827	(0.274)	0.019	0.037
P08207	S100a10	0.734	(0.447)	0.002	0.009
P08226	Apoe	0.799	(0.324)	0.000	0.003
P08508	Fcgr3	0.729	(0.455)	0.000	0.005
P09450	Junb	0.791	(0.339)	0.016	0.032
P0C6B7	Igsf6	0.787	(0.345)	0.001	0.006
P0CW03	Ly6c2	0.712	(0.490)	0.001	0.006
P10639	Txn	0.826	(0.275)	0.000	0.003
P11247	Mpo	0.644	(0.635)	0.008	0.019
P11531	Dmd	0.549	(0.864)	0.001	0.005
P11672	Lcn2	0.717	(0.480)	0.004	0.012
P11680	Cfp	0.830	(0.268)	0.002	0.008
P12265	Gusb	0.786	(0.348)	0.002	0.008
P12388	Serpinb2	0.640	(0.645)	0.001	0.006
P12804	Fgl2	0.782	(0.356)	0.019	0.037
P13595	Ncam1	0.654	(0.613)	0.002	0.009
P13609	Srgn	0.703	(0.509)	0.001	0.005
P14069	S100a6	0.734	(0.447)	0.013	0.028
P14211	Calr	0.784	(0.351)	0.005	0.014
P15306	Thbd	0.705	(0.504)	0.000	0.003
P17047	Lamp2	0.753	(0.410)	0.004	0.012
P17809	Slc2a1	0.733	(0.448)	0.034	0.059
P18528		0.244	(2.035)	0.003	0.010
P19467	Muc13	0.693	(0.529)	0.000	0.004
P20612	Gnat1	0.575	(0.799)	0.001	0.006
P20918	Plg	0.831	(0.267)	0.007	0.017
P21460	Cst3	0.811	(0.302)	0.000	0.005
P21995	Emb	0.709	(0.495)	0.009	0.021
P22366	Myd88	0.822	(0.283)	0.001	0.007
P23738	Hdc	0.673	(0.572)	0.001	0.007
P25118	Tnfrsf1a	0.777	(0.363)	0.000	0.003
P27005	S100a8	0.741	(0.432)	0.000	0.004
P27046	Man2a1	0.809	(0.305)	0.000	0.004
P27659	Rpl3	0.816	(0.293)	0.003	0.011
P27784	Ccl6	0.707	(0.500)	0.005	0.013
P27808	Mgat1	0.731	(0.451)	0.000	0.005
P27931	Il1r2	0.534	(0.904)	0.016	0.033
P28033	Cebpb	0.696	(0.524)	0.031	0.054
P28293	Ctsg	0.535	(0.903)	0.001	0.006
P28658	Atxn10	0.749	(0.417)	0.005	0.014
P28667	Marcksl1	0.774	(0.370)	0.021	0.041
P30204	Msr1	0.639	(0.646)	0.007	0.017
P30355	Alox5ap	0.829	(0.270)	0.002	0.008
P30993	C5ar1	0.673	(0.572)	0.001	0.006

P31725	S100a9	0.736	(0.442)	0.001	0.005
P31786	Dbi	0.828	(0.272)	0.002	0.009
P32037	Slc2a3	0.779	(0.361)	0.000	0.003
P35173	Stfa3	0.684	(0.549)	0.032	0.055
P35175	Stfa1	0.548	(0.867)	0.047	0.075
P35290	Rab24	0.786	(0.348)	0.002	0.009
P35343	Cxcr2	0.620	(0.689)	0.002	0.008
P35456	Plaur	0.732	(0.451)	0.002	0.007
P35831	Ptpn12	0.821	(0.285)	0.001	0.006
P35917	Flt4	0.626	(0.675)	0.001	0.007
P39654	Alox15	0.735	(0.444)	0.007	0.018
P39876	Timp3	0.749	(0.417)	0.047	0.076
P40142	Tkt	0.810	(0.304)	0.000	0.003
P40223	Csf3r	0.674	(0.569)	0.000	0.003
P41183	Bcl6	0.738	(0.438)	0.020	0.039
P41245	Mmp9	0.833	(0.263)	0.001	0.007
P42227	Stat3	0.832	(0.266)	0.002	0.008
P43432	Il12b	0.778	(0.362)	0.002	0.007
P45878	Fkbp2	0.708	(0.499)	0.000	0.004
P47757	Capzb	0.743	(0.428)	0.009	0.022
P47791	Gsr	0.725	(0.464)	0.000	0.005
P47856	Gfpt1	0.824	(0.279)	0.000	0.004
P48410	Abcd1	0.754	(0.408)	0.003	0.009
P50172	Hsd11b1	0.768	(0.381)	0.002	0.009
P51675	Ccr1	0.643	(0.636)	0.000	0.004
P52624	Upp1	0.318	(1.655)	0.001	0.006
P53566	Cebpa	0.754	(0.407)	0.002	0.008
P57787	Slc16a3	0.669	(0.581)	0.001	0.005
P58069	Rasa2	0.814	(0.297)	0.000	0.004
P59438	Hps5	0.774	(0.369)	0.007	0.017
P62748	Hpcal1	0.820	(0.286)	0.002	0.008
P63002	Tle5	0.635	(0.655)	0.000	0.002
P70158	Smpdl3a	0.711	(0.491)	0.007	0.018
P70362	Ufd1	0.802	(0.319)	0.007	0.019
P70365	Ncoa1	0.793	(0.335)	0.000	0.004
P81122	Irs2	0.742	(0.431)	0.024	0.044
P83626	Tnfrsf26	0.712	(0.490)	0.003	0.010
P84751		0.531	(0.914)	0.001	0.007
P97287	Mcl1	0.769	(0.379)	0.002	0.008
P97302	Bach1	0.763	(0.389)	0.002	0.008
P97346	Nxn	0.827	(0.275)	0.001	0.006
P97360	Etv6	0.783	(0.353)	0.001	0.007
P97363	Sptlc2	0.758	(0.399)	0.000	0.004
P97370	Atp1b3	0.819	(0.288)	0.000	0.004
P97384	Anxa11	0.831	(0.268)	0.001	0.007
P97425	Ear2	0.720	(0.473)	0.008	0.020
P97484	Lilrb3	0.720	(0.475)	0.002	0.009
P97497	Shbg	0.780	(0.358)	0.021	0.041
P97821	Ctsc	0.716	(0.482)	0.050	0.079
P97858	Slc35b1	0.735	(0.444)	0.001	0.007
P97864	Casp7	0.782	(0.354)	0.006	0.017
P97872	Fmo5	0.811	(0.303)	0.000	0.005
Q00519	Xdh	0.785	(0.350)	0.000	0.004
Q00623	Apoa1	0.750	(0.415)	0.023	0.043
Q02819	Nucb1	0.822	(0.283)	0.002	0.008
Q03157	Aplp1	0.687	(0.543)	0.001	0.006
Q04998	Inhba	0.768	(0.381)	0.001	0.005
Q05921	Rnasel	0.794	(0.334)	0.004	0.012
Q09200	B4galnt1	0.746	(0.423)	0.003	0.010
Q09PK2	Asprv1	0.595	(0.748)	0.030	0.052
Q14CH1	Mocos	0.799	(0.324)	0.000	0.004

Q14DT3	Fmo9	0.554	(0.853)	0.002	0.007
Q2YFS1	Pilrb2	0.797	(0.326)	0.007	0.018
Q2YFS3	Pilra	0.779	(0.361)	0.000	0.004
Q3LFS6	Ceacam1	0.742	(0.430)	0.000	0.003
Q3SXF0	Ifitm6	0.583	(0.778)	0.003	0.011
Q3TA40	430548M08R	0.822	(0.283)	0.002	0.009
Q3TA56	Selplg	0.645	(0.632)	0.000	0.004
Q3TCF3	Sell	0.827	(0.274)	0.000	0.004
Q3TDX8	Cyb5r4	0.789	(0.342)	0.000	0.003
Q3TH34	Fam45a	0.832	(0.266)	0.000	0.003
Q3TIR1	Trappc13	0.811	(0.303)	0.001	0.007
Q3TJD7	Pdlim7	0.817	(0.292)	0.000	0.004
Q3TL44	Nlrx1	0.761	(0.395)	0.003	0.010
Q3TMX0	Sdcbp	0.784	(0.352)	0.000	0.004
Q3TNL8	Itprip	0.800	(0.322)	0.001	0.007
Q3TVI8	Pbxip1	0.694	(0.527)	0.000	0.003
Q3TYD6	Lmtk2	0.817	(0.291)	0.001	0.006
Q3U1F9	Pag1	0.785	(0.349)	0.000	0.003
Q3U1N2	Srebf2	0.668	(0.582)	0.001	0.007
Q3U487	Hectd3	0.755	(0.406)	0.003	0.010
Q3U5N6	Ms4a4c	0.703	(0.509)	0.041	0.068
Q3U8S1	Cd44	0.827	(0.273)	0.001	0.005
Q3UKN6	Nucb2	0.796	(0.329)	0.004	0.013
Q3UL50	Wdr74	0.799	(0.323)	0.002	0.009
Q3UMR5	Mcu	0.748	(0.419)	0.001	0.007
Q3UMW7	Mapkapk3	0.831	(0.267)	0.013	0.029
Q3UP87	Elane	0.732	(0.450)	0.000	0.003
Q3UPH1	Prrc1	0.748	(0.420)	0.003	0.010
Q3UPV6	Kcnab2	0.781	(0.356)	0.001	0.007
Q3UPZ5	Ifi213	0.277	(1.851)	0.021	0.040
Q3UR39	Chm	0.799	(0.323)	0.002	0.008
Q3UR44	Mrvi1	0.820	(0.286)	0.014	0.030
Q3USX5	Irak4	0.801	(0.320)	0.005	0.014
Q3UU43	Chpf2	0.820	(0.286)	0.006	0.016
Q3UV74	Itgb2l	0.788	(0.344)	0.000	0.004
Q3UXJ2	Tgfbi	0.737	(0.440)	0.006	0.016
Q3UZ39	Lrrfip1	0.788	(0.343)	0.000	0.003
Q3V3I2	Gnat3	0.220	(2.182)	0.000	0.004
Q3V3R4	Itga1	0.811	(0.302)	0.005	0.015
Q3V493	Trappc4	0.791	(0.338)	0.004	0.012
Q4VBE8	Wdr18	0.739	(0.436)	0.039	0.065
Q505D7	Opa3	0.810	(0.305)	0.001	0.005
Q542I8	Itgb2	0.831	(0.268)	0.001	0.007
Q543K9	Pnp	0.776	(0.365)	0.002	0.008
Q58NB6	Dhrs9	0.588	(0.767)	0.000	0.004
Q5FWI3	Cemip2	0.726	(0.461)	0.000	0.005
Q5S006	Lrrk2	0.786	(0.347)	0.012	0.027
Q5SS83	Flot2	0.777	(0.363)	0.001	0.007
Q5SSE9	Abca13	0.686	(0.543)	0.000	0.004
Q5SSJ1	Wfdc17	0.367	(1.445)	0.010	0.024
Q5SVL6	Rap1gap2	0.807	(0.310)	0.001	0.005
Q5SVR5	Rnf130	0.731	(0.451)	0.002	0.009
Q5SVT1	Trim16	0.825	(0.278)	0.000	0.004
Q5SWU9	Acaca	0.817	(0.292)	0.002	0.008
Q5SYH2	Tmem199	0.811	(0.302)	0.007	0.018
Q5U4H9	Ncoa4	0.789	(0.342)	0.029	0.051
Q60590	Orm1	0.731	(0.452)	0.004	0.012
Q60767	Ly75	0.768	(0.381)	0.000	0.003
Q60866	Pter	0.733	(0.449)	0.001	0.007
Q60898	Sla	0.750	(0.415)	0.008	0.019
Q60943	Il17ra	0.730	(0.454)	0.005	0.013

Q61096	Prtn3	0.613	(0.705)	0.000	0.003
Q61122	Nab1	0.760	(0.397)	0.002	0.008
Q61235	Sntb2	0.771	(0.376)	0.000	0.004
Q61263	Soat1	0.657	(0.606)	0.027	0.048
Q61333	Tnfaip2	0.765	(0.387)	0.001	0.005
Q61362	Chi3l1	0.788	(0.344)	0.001	0.006
Q61419	Cmah	0.769	(0.378)	0.007	0.018
Q61462	Cyba	0.820	(0.286)	0.004	0.012
Q61646	Hp	0.635	(0.656)	0.001	0.007
Q61805	Lbp	0.798	(0.326)	0.002	0.008
Q61878	Prg2	0.752	(0.411)	0.013	0.029
Q62313	Tgoln1	0.765	(0.387)	0.001	0.006
Q62433	Ndrg1	0.782	(0.356)	0.007	0.018
Q640L3	Ccpg1	0.811	(0.303)	0.004	0.012
Q64281	Lilrb4	0.622	(0.685)	0.006	0.017
Q64727	Vcl	0.816	(0.293)	0.001	0.005
Q69Z37	Samd9l	0.830	(0.269)	0.012	0.027
Q69ZF7	Cnnm4	0.773	(0.372)	0.003	0.011
Q6A037	N4bp1	0.818	(0.290)	0.023	0.043
Q6DID7	Wls	0.750	(0.415)	0.001	0.005
Q6DTY7	Pfkfb4	0.805	(0.313)	0.000	0.004
Q6GV12	Kdsr	0.740	(0.434)	0.002	0.008
Q6NSQ5	Sp140	0.787	(0.345)	0.006	0.016
Q6NVF0	Ocrl	0.722	(0.469)	0.001	0.005
Q6NVF2	Pld1	0.726	(0.461)	0.000	0.004
Q6NXL1	Sec24d	0.777	(0.363)	0.007	0.017
Q6NZM9	Hdac4	0.801	(0.319)	0.001	0.006
Q6P2B1	Tnpo3	0.830	(0.268)	0.001	0.007
Q6P6I8	Sirpa	0.804	(0.314)	0.000	0.004
Q6P8H8	Alg8	0.740	(0.434)	0.008	0.019
Q6P9Q4	Fhod1	0.822	(0.283)	0.000	0.005
Q6PD10	Ip6k1	0.802	(0.319)	0.001	0.007
Q6PHU5	Sort1	0.742	(0.430)	0.001	0.006
Q6PIC6	Atp1a3	0.802	(0.318)	0.000	0.003
Q6R5N8	Tlr13	0.798	(0.325)	0.011	0.025
Q6VNB8	Wdfy3	0.697	(0.521)	0.001	0.006
Q6ZPT1	Klhl9	0.494	(1.018)	0.001	0.007
Q6ZQ29	Taok2	0.773	(0.371)	0.003	0.010
Q6ZQJ5	Dna2	0.802	(0.319)	0.009	0.021
Q6ZQM8	Ugt1a7c	0.807	(0.309)	0.005	0.013
Q78IS1	Tmed3	0.775	(0.367)	0.000	0.004
Q78J03	Msrb2	0.541	(0.887)	0.000	0.003
Q78XF5	Ostc	0.716	(0.481)	0.000	0.004
Q7M759	Abhd17b	0.825	(0.277)	0.034	0.059
Q7TMC8	Fcsk	0.777	(0.365)	0.011	0.025
Q7TNB8	Sbno2	0.712	(0.490)	0.006	0.017
Q80TN4	Dnajc16	0.801	(0.319)	0.004	0.012
Q80TN7	Nav3	0.512	(0.965)	0.002	0.009
Q80TY5	Vps13b	0.755	(0.406)	0.012	0.027
Q80W94	Mogat2	0.734	(0.445)	0.003	0.010
Q80XL7	Mppe1	0.826	(0.276)	0.006	0.017
Q80Y24	Prickle2	0.441	(1.182)	0.000	0.004
Q80Y86	Mapk15	0.658	(0.603)	0.001	0.006
Q80ZD9	Amigo2	0.673	(0.571)	0.035	0.059
Q80ZE2	Siglecf	0.728	(0.459)	0.006	0.017
Q80ZJ1	Rap2a	0.778	(0.361)	0.010	0.024
Q80ZP8	Manf	0.709	(0.495)	0.001	0.005
Q80ZQ9	Abitram	0.558	(0.841)	0.000	0.004
Q8BFY6	Pef1	0.787	(0.346)	0.024	0.044
Q8BG07	Pld4	0.673	(0.572)	0.000	0.004
Q8BGD4	Slco4c1	0.779	(0.360)	0.002	0.008

Q8BGG7	Ubash3b	0.818	(0.290)	0.000	0.003
Q8BGS5	Zscan22	0.779	(0.361)	0.011	0.025
Q8BGZ6	Gla	0.733	(0.448)	0.003	0.010
Q8BH27	Megf9	0.784	(0.351)	0.001	0.006
Q8BH61	F13a1	0.694	(0.527)	0.002	0.008
Q8BHL5	Elmo2	0.821	(0.284)	0.001	0.006
Q8BJT9	Edem2	0.768	(0.381)	0.026	0.047
Q8BKC8	Pi4kb	0.814	(0.298)	0.028	0.050
Q8BLR5	Psd4	0.789	(0.341)	0.001	0.006
Q8BLY7	Hps6	0.802	(0.319)	0.001	0.007
Q8BMD8	Slc25a24	0.813	(0.299)	0.004	0.012
Q8BMK4	Ckap4	0.764	(0.389)	0.001	0.007
Q8BMQ8	Mon1b	0.735	(0.445)	0.001	0.007
Q8BND5	Qsox1	0.659	(0.601)	0.001	0.007
Q8BPN8	Dmxl2	0.755	(0.405)	0.004	0.012
Q8BR63	Fam177a1	0.827	(0.273)	0.000	0.004
Q8BRB6	Tbc1d24	0.819	(0.288)	0.002	0.008
Q8BT14	Cnot4	0.791	(0.338)	0.012	0.026
Q8BTE6	Wfdc21	0.621	(0.688)	0.002	0.007
Q8BTI9	Pik3cb	0.805	(0.312)	0.001	0.005
Q8BTU6	Eif4a2	0.818	(0.290)	0.002	0.009
Q8BUE4	Aifm2	0.818	(0.289)	0.005	0.014
Q8BUM3	Ptpn7	0.800	(0.322)	0.000	0.003
Q8BUR4	Dock1	0.715	(0.483)	0.000	0.004
Q8BV16	Zfp398	0.657	(0.605)	0.030	0.053
Q8BVF2	Pdcl3	0.825	(0.277)	0.034	0.058
Q8BVF7	Aph1a	0.807	(0.309)	0.000	0.004
Q8BVT7	921511C20R	0.632	(0.661)	0.028	0.050
Q8BWG9	Orai1	0.735	(0.445)	0.001	0.005
Q8BWM3	Stfa2l1	0.812	(0.301)	0.008	0.020
Q8BWY4	Mblac1	0.721	(0.471)	0.000	0.003
Q8BX37	Acp7	0.547	(0.871)	0.003	0.010
Q8BXQ2	Pigt	0.832	(0.266)	0.002	0.008
Q8BYI6	Lpcat2	0.773	(0.372)	0.000	0.004
Q8BYP3	Rhof	0.761	(0.394)	0.001	0.005
Q8C0L8	Cog5	0.803	(0.316)	0.005	0.015
Q8C1F4	Csgalnact2	0.798	(0.326)	0.001	0.006
Q8C4Q9	Tmem154	0.676	(0.565)	0.000	0.002
Q8C5R8	Prps1l1	0.804	(0.315)	0.003	0.010
Q8C6L5	Cgas	0.808	(0.308)	0.001	0.005
Q8C7K6	Pcyox1l	0.802	(0.319)	0.003	0.010
Q8C9S4	Ccdc186	0.777	(0.364)	0.002	0.008
Q8CB87	Rab44	0.793	(0.335)	0.000	0.004
Q8CB96	Rassf4	0.809	(0.305)	0.015	0.031
Q8CCI5	Rybp	0.739	(0.437)	0.002	0.008
Q8CCJ3	Ufl1	0.764	(0.389)	0.001	0.007
Q8CDU6	Hectd2	0.443	(1.176)	0.005	0.013
Q8CF66	Lamtor4	0.592	(0.756)	0.010	0.022
Q8CHY3	Dym	0.802	(0.318)	0.003	0.011
Q8CI51	Pdlim5	0.820	(0.286)	0.001	0.005
Q8CJ26	Nradd	0.669	(0.579)	0.001	0.007
Q8JZK9	Hmgcs1	0.827	(0.274)	0.013	0.028
Q8JZN1	Mgl2	0.694	(0.527)	0.000	0.004
Q8K021	Scamp1	0.699	(0.516)	0.002	0.008
Q8K0C1	Ipo13	0.802	(0.318)	0.012	0.027
Q8K0E1	Kctd15	0.617	(0.697)	0.002	0.007
Q8K194	Snrnp27	0.830	(0.268)	0.002	0.009
Q8K199	Cmc2	0.832	(0.266)	0.008	0.020
Q8K1H7	Tcp11l2	0.721	(0.473)	0.021	0.040
Q8K1T1	Lrrc25	0.771	(0.375)	0.000	0.002
Q8K211	Slc31a1	0.792	(0.337)	0.031	0.054

Q8K2P7	Slc38a1	0.806	(0.311)	0.031	0.054
Q8K385	FRRS1	0.812	(0.300)	0.007	0.019
Q8QZZ8	Rab38	0.742	(0.431)	0.001	0.005
Q8R173	Zdhhc3	0.734	(0.445)	0.000	0.004
Q8R1H0	Hopx	0.778	(0.363)	0.027	0.049
Q8R2E9	Ero1b	0.610	(0.714)	0.000	0.003
Q8R2H9	Phospho1	0.763	(0.391)	0.002	0.008
Q8R2I1	Slc35c1	0.729	(0.457)	0.003	0.011
Q8R2R3	Aagab	0.800	(0.322)	0.005	0.014
Q8R2S8	Cd177	0.656	(0.608)	0.001	0.005
Q8R2W9	Pank3	0.762	(0.392)	0.000	0.003
Q8R3D1	Tbc1d13	0.796	(0.329)	0.020	0.039
Q8R460	Il36g	0.762	(0.393)	0.015	0.032
Q8R4G6	Mgat5	0.731	(0.451)	0.001	0.006
Q8VBT6	Apobr	0.762	(0.391)	0.000	0.004
Q8VC30	Tkfc	0.781	(0.356)	0.006	0.016
Q8VC51	Wrap53	0.822	(0.283)	0.001	0.006
Q8VC88	Gca	0.799	(0.324)	0.003	0.011
Q8VDD8	Washc1	0.759	(0.398)	0.001	0.007
Q8VDG7	Pafah2	0.767	(0.384)	0.001	0.007
Q8VDS8	Stx18	0.829	(0.270)	0.004	0.011
Q8VEB3	D1Ertd622e	0.737	(0.440)	0.003	0.010
Q8VEB6	Elac1	0.779	(0.360)	0.000	0.003
Q8VEE0	Rpe	0.794	(0.333)	0.000	0.003
Q8VGR6	Olfr393	0.596	(0.747)	0.012	0.026
Q8VHC3	Selenom	0.553	(0.856)	0.000	0.003
Q91V77	S100a1	0.824	(0.280)	0.005	0.015
Q91V83	Tti1	0.774	(0.369)	0.000	0.003
Q91VI7	Rnh1	0.722	(0.470)	0.001	0.007
Q91VW5	Golga4	0.820	(0.287)	0.000	0.002
Q91W18	Tdrd3	0.778	(0.363)	0.001	0.005
Q91W90	Txndc5	0.631	(0.665)	0.008	0.019
Q91WB7	Ubtd1	0.799	(0.324)	0.007	0.019
Q91WC9	Daglb	0.833	(0.264)	0.004	0.012
Q91WD4		0.822	(0.283)	0.022	0.042
Q91X72	Hpx	0.662	(0.595)	0.003	0.010
Q91XF0	Pnpo	0.820	(0.286)	0.008	0.020
Q91XL1	Lrg1	0.553	(0.856)	0.004	0.012
Q91Y57	Siglec12	0.775	(0.368)	0.010	0.023
Q91YI4	Arrb2	0.826	(0.276)	0.000	0.004
Q91YR9	Ptgr1	0.811	(0.302)	0.015	0.032
Q91YS8	Camk1	0.833	(0.264)	0.029	0.052
Q91ZI0	Celsr3	0.784	(0.350)	0.001	0.007
Q920C4	Ms4a3	0.688	(0.540)	0.008	0.019
Q920E5	Fdps	0.565	(0.824)	0.004	0.013
Q921Q3	Alg1	0.766	(0.385)	0.004	0.012
Q921V5	Mgat2	0.822	(0.283)	0.001	0.006
Q921X9	Pdia5	0.751	(0.414)	0.001	0.005
Q921Y4	Mfsd5	0.814	(0.297)	0.002	0.008
Q922B6	Traf7	0.788	(0.344)	0.001	0.006
Q923B6	Steap4	0.743	(0.429)	0.011	0.025
Q923L6	Ear10	0.818	(0.290)	0.004	0.011
Q923L7	Ear6	0.690	(0.535)	0.001	0.006
Q924A2	Cic	0.782	(0.354)	0.001	0.007
Q99104	Myo5a	0.803	(0.317)	0.003	0.010
Q99J27	Slc33a1	0.684	(0.548)	0.003	0.010
Q99J56	Derl1	0.754	(0.407)	0.002	0.007
Q99J93	Ifitm2	0.586	(0.772)	0.010	0.024
Q99JF5	Mvd	0.772	(0.373)	0.019	0.037
Q99JH8	Kdelr1	0.642	(0.640)	0.001	0.006
Q99K41	Emilin1	0.760	(0.395)	0.005	0.014

Q99KL7	Rab28	0.796	(0.329)	0.000	0.004
Q99KN2	Ciao1	0.810	(0.304)	0.001	0.007
Q99KR8	Fuca2	0.831	(0.266)	0.001	0.007
Q99KV1	Dnajb11	0.788	(0.345)	0.000	0.004
Q99L00	Haus8	0.795	(0.332)	0.001	0.007
Q99LC9	Pex6	0.814	(0.297)	0.025	0.046
Q99LQ1	Mbip	0.741	(0.432)	0.001	0.005
Q99LW0	Ankrd10	0.751	(0.412)	0.026	0.047
Q99M15	Pstpip2	0.779	(0.360)	0.002	0.009
Q99N08	Ms4a6c	0.550	(0.862)	0.016	0.033
Q99N16	Cyp4f3	0.681	(0.555)	0.003	0.011
Q99P30	Nudt7	0.785	(0.349)	0.001	0.006
Q9CPW2	Fdx2	0.804	(0.314)	0.014	0.030
Q9CPZ8	Cmc1	0.724	(0.467)	0.036	0.061
Q9CQ26	Stambp	0.706	(0.502)	0.001	0.005
Q9CQ88	Tspan31	0.786	(0.348)	0.000	0.002
Q9CQF0	Mrpl11	0.700	(0.515)	0.002	0.009
Q9CQI9	Med30	0.744	(0.427)	0.033	0.057
Q9CQX2	Cyb5b	0.751	(0.412)	0.008	0.020
Q9CR25	Dph2	0.789	(0.341)	0.015	0.031
Q9CR89	Ergic2	0.805	(0.312)	0.017	0.035
Q9CRC0	Vkorc1	0.780	(0.359)	0.001	0.005
Q9CWM2	Cdca4	0.445	(1.168)	0.000	0.005
Q9CWR1	Wdr73	0.680	(0.556)	0.001	0.007
Q9CXH7	Sgo1	0.509	(0.974)	0.002	0.009
Q9CY34	Ube2f	0.795	(0.331)	0.006	0.017
Q9CY45	Eef1akmt1	0.813	(0.299)	0.004	0.013
Q9CYA0	Creld2	0.545	(0.876)	0.000	0.003
Q9CZE3	Rab32	0.782	(0.354)	0.000	0.004
Q9D0C1	Rnf115	0.788	(0.344)	0.001	0.007
Q9D0F3	Lman1	0.830	(0.269)	0.000	0.005
Q9D0R4	Ddx56	0.833	(0.264)	0.002	0.007
Q9D103	Ifitm1	0.402	(1.315)	0.037	0.062
Q9D1E6	Tbcb	0.796	(0.329)	0.011	0.025
Q9D1I2	Card19	0.818	(0.290)	0.000	0.004
Q9D1M7	Fkbp11	0.666	(0.587)	0.001	0.007
Q9D1X9	Tmem50b	0.766	(0.385)	0.012	0.027
Q9D2N9	Vps33a	0.803	(0.317)	0.002	0.008
Q9D2R0	Aacs	0.787	(0.346)	0.000	0.004
Q9D2V8	Mfsd10	0.812	(0.301)	0.002	0.007
Q9D3R1	Klra2	0.802	(0.318)	0.003	0.010
Q9D4H2	Gcc1	0.810	(0.304)	0.001	0.006
Q9D659	Vsir	0.792	(0.336)	0.003	0.011
Q9D787	Ppil2	0.832	(0.265)	0.001	0.005
Q9D7I5	Lhpp	0.772	(0.373)	0.009	0.022
Q9D7Q1	Chit1	0.690	(0.535)	0.013	0.028
Q9D8C9	Pnp2	0.679	(0.558)	0.003	0.011
Q9D8D6	Csta2	0.787	(0.346)	0.022	0.042
Q9D8I1	Mzb1	0.587	(0.769)	0.049	0.077
Q9D8T4	Tvp23b	0.823	(0.280)	0.003	0.010
Q9D8U6	Mcemp1	0.742	(0.431)	0.001	0.006
Q9D8Y0	Efhd2	0.777	(0.364)	0.000	0.003
Q9D8Y7	Tnfaip8l2	0.687	(0.541)	0.000	0.003
Q9D964	Gatm	0.729	(0.456)	0.001	0.007
Q9D9M5	Phospho2	0.798	(0.326)	0.027	0.049
Q9DA17	Tsga13	0.811	(0.303)	0.002	0.007
Q9DAS1	Cklf	0.787	(0.345)	0.000	0.004
Q9DAU1	Cnpy3	0.817	(0.291)	0.002	0.008
Q9DBG5	Plin3	0.783	(0.352)	0.005	0.014
Q9DBH5	Lman2	0.803	(0.317)	0.001	0.006
Q9DBL1	Acadsb	0.711	(0.492)	0.004	0.012

Q9DBU3	Riok3	0.708	(0.498)	0.019	0.037
Q9DBX1	Rgcc	0.768	(0.381)	0.005	0.014
Q9DBX5	Pla2g4a	0.831	(0.266)	0.000	0.004
Q9DC50	Crot	0.826	(0.275)	0.004	0.011
Q9DCL2	Ciao2a	0.763	(0.390)	0.000	0.005
Q9DCM0	Ethe1	0.830	(0.269)	0.000	0.004
Q9DCR2	Ap3s1	0.722	(0.469)	0.009	0.021
Q9DD03	Rab13	0.602	(0.731)	0.000	0.004
Q9EPL4	Mettl9	0.705	(0.505)	0.003	0.010
Q9EPL9	Acox3	0.830	(0.269)	0.002	0.007
Q9EPU5	Tnfrsf21	0.414	(1.271)	0.002	0.008
Q9ER39	Tor1a	0.803	(0.317)	0.006	0.016
Q9JHP7	Poglut2	0.830	(0.268)	0.017	0.035
Q9JI08	Bin3	0.701	(0.512)	0.004	0.012
Q9JJ00	Plscr1	0.627	(0.672)	0.006	0.016
Q9JKN1	Slc30a7	0.699	(0.516)	0.000	0.004
Q9JL95	Prg3	0.767	(0.382)	0.016	0.032
Q9JL99	Clec1b	0.821	(0.284)	0.031	0.054
Q9JLJ0	Litaf	0.832	(0.265)	0.004	0.012
Q9JLJ1	Selenok	0.724	(0.465)	0.001	0.006
Q9JMH6	Txnrd1	0.823	(0.281)	0.006	0.016
Q9QXS1	Plec	0.804	(0.314)	0.006	0.017
Q9QYB5	Add3	0.807	(0.309)	0.000	0.004
Q9QZ15	Clec4a	0.806	(0.312)	0.000	0.004
Q9QZF2	Gpc1	0.789	(0.343)	0.001	0.006
Q9QZU3	Gp5	0.790	(0.340)	0.024	0.045
Q9QZU9	Ube2l6	0.817	(0.292)	0.050	0.079
Q9R0E1	Plod3	0.741	(0.432)	0.010	0.024
Q9R111	Gda	0.621	(0.688)	0.001	0.006
Q9R1Q7	Plp2	0.756	(0.404)	0.010	0.023
Q9WTS2	Fut8	0.724	(0.465)	0.000	0.004
Q9WU72	Tnfsf13b	0.813	(0.299)	0.005	0.014
Q9WUZ9	Entpd5	0.815	(0.295)	0.000	0.004
Q9WVG5	Lipg	0.475	(1.075)	0.044	0.072
Q9Z0H7	Bcl10	0.823	(0.282)	0.000	0.004
Q9Z0P4	Palm	0.811	(0.303)	0.000	0.005
Q9Z126	Pf4	0.796	(0.329)	0.032	0.055
Q9Z1A9	Tbc1d8	0.825	(0.278)	0.001	0.006
Q9Z1B7	Mapk13	0.825	(0.278)	0.000	0.003
Q9Z1R4	D17h6s53e	0.815	(0.294)	0.005	0.013
Q9Z2A7	Dgat1	0.794	(0.333)	0.003	0.010
Q9Z2C5	Mtm1	0.741	(0.433)	0.048	0.076
Q9Z2I2	Fkbp1b	0.781	(0.357)	0.000	0.003
Q9Z2X2	Psmd10	0.813	(0.299)	0.000	0.005
S4R1V2	Milr1	0.720	(0.474)	0.001	0.005
S4R1X1	Wdr91	0.340	(1.555)	0.002	0.008
S4R2S6	Tbc1d25	0.792	(0.336)	0.001	0.007
V9GX53	Erc1	0.827	(0.275)	0.017	0.034
A0A075B5Y4	Ighv1-81	0.860	(0.217)	0.039	0.065
A0A087WNU6	Lrrfip1	0.869	(0.203)	0.017	0.034
A0A087WNV1	Agfg1	0.993	(0.010)	0.570	0.626
A0A087WNZ7	Trip12	0.996	(0.005)	0.859	0.884
A0A087WP24	Abhd14b	1.012	0.017	0.830	0.860
A0A087WPI5	Zfp119b	0.858	(0.222)	0.020	0.038
A0A087WPK1	Cbll1	1.059	0.083	0.144	0.191
A0A087WPR9	Retreg2	1.026	0.038	0.402	0.462
A0A087WPZ6	Ppil3	1.070	0.098	0.284	0.343
A0A087WQ31	Clasp1	0.947	(0.079)	0.012	0.027
A0A087WQB0	Morf4l1	0.998	(0.003)	0.962	0.967
A0A087WQE6	Eloc	1.096	0.132	0.102	0.143
A0A087WQH8	Slc35a4	1.020	0.029	0.574	0.630

A0A087WQN6	Eef1akmt2	0.989	(0.016)	0.724	0.767
A0A087WQN7	Xrn1	0.930	(0.104)	0.057	0.088
A0A087WR08	Taf9	1.039	0.055	0.017	0.035
A0A087WRH2	Faim	0.985	(0.021)	0.383	0.444
A0A087WS98	Actr3	0.847	(0.240)	0.002	0.008
A0A087WSB5	Nudt16	1.169	0.225	0.006	0.015
A0A087WSG5	Mnat1	0.927	(0.109)	0.228	0.284
A0A0A0MQ73	Kmt2d	0.911	(0.134)	0.004	0.013
A0A0A0MQ79	Prrc2c	0.942	(0.087)	0.043	0.070
A0A0A0MQ89	Actr5	0.887	(0.173)	0.012	0.027
A0A0A0MQF5	Otud6b	0.893	(0.163)	0.002	0.009
A0A0A0MQL5	Phactr4	0.897	(0.157)	0.123	0.168
A0A0A0MQM0	Eif5a	0.958	(0.062)	0.190	0.243
A0A0A6YVZ8	Map4k4	1.002	0.003	0.927	0.939
A0A0A6YW87	Cnih4	1.029	0.042	0.565	0.621
A0A0A6YWB3	Gpatch4	1.080	0.111	0.059	0.091
A0A0A6YWD3	Lin9	0.934	(0.098)	0.050	0.079
A0A0A6YWP6	Atp6v1h	0.899	(0.154)	0.018	0.036
A0A0A6YWP9	Txndc9	1.197	0.259	0.011	0.026
A0A0A6YWS6	Utp25	1.044	0.062	0.132	0.178
A0A0A6YWZ0	Cryzl2	1.084	0.117	0.229	0.285
A0A0A6YX93	Zc3h11a	0.868	(0.204)	0.016	0.032
A0A0A6YXE3	Mbnl1	1.011	0.015	0.515	0.573
A0A0A6YXG0	Stx6	1.036	0.051	0.253	0.311
A0A0A6YXR0	Cept1	0.863	(0.213)	0.002	0.007
A0A0A6YXS8	Serpinc1	1.004	0.005	0.902	0.920
A0A0A6YXW7	Uck2	1.077	0.107	0.241	0.297
A0A0A6YXX3	Lrba	1.022	0.032	0.317	0.377
A0A0A6YY47	Ncam1	0.852	(0.232)	0.020	0.038
A0A0A6YY75	Cr1l	0.948	(0.078)	0.001	0.005
A0A0B4J1E7	Kpna4	0.967	(0.049)	0.408	0.468
A0A0B4J1F5	Tmem131l	1.228	0.296	0.074	0.110
A0A0B4J1L8	Cd101	0.863	(0.213)	0.014	0.029
A0A0F6AIX5	Ldah	1.011	0.016	0.767	0.804
A0A0G2JDI9	Abcd3	0.875	(0.192)	0.022	0.041
A0A0G2JDK0	Mef2c	1.176	0.234	0.018	0.036
A0A0G2JDW7	Rps27	1.013	0.019	0.751	0.789
A0A0G2JDX4	Tspan2	0.859	(0.219)	0.044	0.072
A0A0G2JE32	Ube2d3	0.893	(0.163)	0.056	0.086
A0A0G2JEG3	Slc35f6	1.005	0.007	0.798	0.832
A0A0G2JEP4	Lrrfip2	0.944	(0.084)	0.035	0.060
A0A0G2JF13	Crlf2	0.878	(0.188)	0.062	0.094
A0A0G2JFF1	Ppp1cc	0.969	(0.045)	0.679	0.727
A0A0G2JFF9	Hax1	1.031	0.044	0.573	0.628
A0A0G2JFH0	Anapc5	1.133	0.180	0.001	0.006
A0A0G2JFJ6	Ints3	0.957	(0.064)	0.079	0.116
A0A0G2JFQ4	Nbeal2	0.888	(0.171)	0.002	0.008
A0A0G2JFY0	Spata5	1.152	0.204	0.057	0.088
A0A0G2JFZ4	Tsc22d2	0.874	(0.194)	0.122	0.167
A0A0G2JGL8	Dhx30	0.857	(0.223)	0.002	0.008
A0A0G2JGT5	Eif4e	1.137	0.185	0.012	0.026
A0A0H2UKB8	Usp33	0.909	(0.138)	0.001	0.005
A0A0J9YTU1	Khk	0.893	(0.163)	0.001	0.005
A0A0J9YTU3	Specc1	0.872	(0.197)	0.000	0.003
A0A0J9YU54	Dcp2	0.839	(0.253)	0.001	0.006
A0A0J9YUA3	Hip1	1.041	0.058	0.061	0.093
A0A0J9YUD5	Nup205	1.168	0.224	0.000	0.003
A0A0J9YUM4	Ctbp1	1.103	0.141	0.009	0.022
A0A0J9YUT8	Rbm19	0.943	(0.084)	0.088	0.127
A0A0J9YV01	Zeb2	1.167	0.223	0.022	0.042
A0A0J9YV20	Sdad1	1.186	0.246	0.014	0.030

A0A0M3HEP7	Atp8a1	0.966	(0.050)	0.026	0.048
A0A0M3HEP8	Vkorc1l1	0.971	(0.042)	0.683	0.730
A0A0N4SUH8	Nfu1	1.072	0.100	0.037	0.063
A0A0N4SUL6	Emg1	1.136	0.184	0.003	0.010
A0A0N4SUS4	Brpf1	1.106	0.146	0.004	0.011
A0A0N4SUY2	Ptpro	1.004	0.006	0.913	0.929
A0A0N4SV05	Ppp4r2	0.955	(0.067)	0.185	0.238
A0A0N4SV10	Etnk1	1.040	0.057	0.622	0.673
A0A0N4SV29	Fancd2	0.909	(0.137)	0.110	0.153
A0A0N4SV66	H2afj	0.985	(0.022)	0.839	0.867
A0A0N4SV80	Zfp638	0.959	(0.060)	0.017	0.034
A0A0N4SVS6	Cnbp	1.008	0.011	0.904	0.921
A0A0N4SVT3	Gng12	0.967	(0.049)	0.582	0.637
A0A0N4SVT8	Dnajb8	0.923	(0.115)	0.014	0.030
A0A0N4SW50	Tmem168	0.934	(0.099)	0.393	0.453
A0A0N4SWB9	Tmcc1	0.916	(0.127)	0.051	0.080
A0A0N5E9G7	Rfc1	1.064	0.089	0.003	0.011
A0A0R3P9C8	Ndufa9	1.089	0.123	0.031	0.054
A0A0R4IZX1	Def6	0.849	(0.236)	0.004	0.011
A0A0R4IZX3	Med6	1.030	0.042	0.456	0.515
A0A0R4IZY0	Thop1	1.039	0.055	0.008	0.020
A0A0R4IZY9	Dus3l	1.046	0.065	0.167	0.218
A0A0R4J023	Auh	0.955	(0.067)	0.309	0.369
A0A0R4J047	Luc7l	1.007	0.010	0.581	0.636
A0A0R4J060	Sap130	1.178	0.236	0.004	0.012
A0A0R4J069	Scly	0.976	(0.035)	0.357	0.418
A0A0R4J078	Ubxn4	0.990	(0.015)	0.627	0.678
A0A0R4J079	Acbd3	0.999	(0.001)	0.924	0.938
A0A0R4J094	Fahd2a	1.077	0.106	0.036	0.060
A0A0R4J0B2	Plbd1	0.889	(0.169)	0.015	0.031
A0A0R4J0B4	Cmas	0.939	(0.090)	0.009	0.022
A0A0R4J0C0	Nsmce1	1.120	0.163	0.002	0.008
A0A0R4J0C8	Eri1	0.857	(0.223)	0.151	0.199
A0A0R4J0D3	Stt3b	0.991	(0.013)	0.758	0.796
A0A0R4J0E4	Ints7	1.168	0.225	0.001	0.005
A0A0R4J0F6	Gak	1.008	0.012	0.781	0.817
A0A0R4J0H7	Ncapd2	1.004	0.005	0.889	0.910
A0A0R4J0I1	Serpina3k	1.087	0.120	0.188	0.241
A0A0R4J0I9	Lrp1	0.837	(0.257)	0.031	0.054
A0A0R4J0J6	Thoc5	1.119	0.162	0.004	0.012
A0A0R4J0L5	Cog6	0.893	(0.163)	0.007	0.018
A0A0R4J0M1	Tbcc	0.933	(0.100)	0.090	0.129
A0A0R4J0M9	Vcpip1	1.010	0.014	0.487	0.545
A0A0R4J0R3	Isg20l2	1.168	0.224	0.002	0.009
A0A0R4J0T0	Hscb	1.034	0.048	0.332	0.393
A0A0R4J0T4	Ikbkb	0.909	(0.138)	0.002	0.007
A0A0R4J0U7	Commd5	0.950	(0.074)	0.309	0.370
A0A0R4J0V5	Polr2a	1.154	0.207	0.000	0.004
A0A0R4J0W6	Lrrc40	0.963	(0.054)	0.264	0.322
A0A0R4J0X5	Serpina1c	1.157	0.210	0.002	0.008
A0A0R4J135	Selenbp2	1.010	0.014	0.732	0.773
A0A0R4J140	Cluh	1.046	0.065	0.272	0.331
A0A0R4J150	Osbpl8	1.104	0.142	0.007	0.018
A0A0R4J170	Smarca4	1.133	0.181	0.000	0.003
A0A0R4J174	Ecsit	1.122	0.166	0.004	0.012
A0A0R4J195	Rprd1b	1.112	0.154	0.004	0.012
A0A0R4J1B6	Dennd5a	1.028	0.039	0.456	0.516
A0A0R4J1B9	Dap3	0.911	(0.135)	0.150	0.199
A0A0R4J1E8	Tatdn1	0.902	(0.148)	0.104	0.146
A0A0R4J1F6	Uap1	0.945	(0.081)	0.075	0.111
A0A0R4J1F8	Wdhd1	1.024	0.035	0.234	0.290

A0A0R4J1H6	Golga3	0.897	(0.157)	0.000	0.004
A0A0R4J1K4	Slc4a2	1.140	0.188	0.007	0.017
A0A0R4J1N8	Rdh13	1.013	0.019	0.720	0.764
A0A0R4J1P8	Matk	0.876	(0.192)	0.010	0.023
A0A0R4J1R7	Pcbd2	1.166	0.221	0.001	0.006
A0A0R4J1U7	Rasal3	0.964	(0.053)	0.230	0.286
A0A0R4J1Y3	Osgep	1.092	0.127	0.005	0.014
A0A0R4J212	Nedd9	0.957	(0.064)	0.182	0.234
A0A0R4J227	Mark2	0.909	(0.137)	0.015	0.032
A0A0R4J244	Papola	1.126	0.171	0.005	0.013
A0A0R4J260	Otud4	0.974	(0.038)	0.224	0.280
A0A0R4J293	Tgm1	1.049	0.069	0.376	0.437
A0A0R4J2B0	Celf2	1.125	0.171	0.102	0.143
A0A0R4J2B2	Kctd12	1.105	0.144	0.048	0.077
A0A0R4J2B6	Rbbp5	1.022	0.032	0.531	0.588
A0A0U1RNH9	Pik3c2a	0.952	(0.071)	0.443	0.503
A0A0U1RNI6	Cdipt	0.952	(0.072)	0.030	0.053
A0A0U1RNK7	Dock7	0.905	(0.144)	0.061	0.093
A0A0U1RNL9	Maz	1.072	0.101	0.489	0.547
A0A0U1RNN5	Spn	0.885	(0.176)	0.048	0.077
A0A0U1RNV6	Kdm3a	0.941	(0.088)	0.004	0.013
A0A0U1RP06	Xpc	1.002	0.003	0.940	0.949
A0A0U1RP81	Immt	1.079	0.110	0.040	0.066
A0A0U1RP93	Mylpf	0.985	(0.021)	0.960	0.966
A0A0U1RPD8	Gtf3c1	1.030	0.043	0.315	0.375
A0A0U1RPF3	Cib1	0.895	(0.160)	0.030	0.053
A0A0U1RPI8	Selenos	0.911	(0.135)	0.024	0.045
A0A0U1RPM2	Fes	0.869	(0.202)	0.006	0.016
A0A0U1RPU3	Iqgap1	1.024	0.035	0.017	0.034
A0A0U1RPX7	Myo7a	0.888	(0.172)	0.028	0.050
A0A0U1RPY8	Coro1a	0.872	(0.198)	0.003	0.010
A0A140LHA2	Bub3	1.153	0.205	0.001	0.005
A0A140LHL5	Sirt2	0.931	(0.103)	0.007	0.018
A0A140LHM2	Psenen	1.109	0.149	0.007	0.018
A0A140LHR3	Spcs2	0.905	(0.144)	0.061	0.093
A0A140LHT6	Mrpl48	0.920	(0.120)	0.005	0.014
A0A140LHY7	Pold3	1.167	0.223	0.000	0.004
A0A140LI69	Zfp444	1.044	0.062	0.044	0.071
A0A140LJF3	Zfand6	0.922	(0.117)	0.034	0.059
A0A140LJJ5	Akap13	0.945	(0.082)	0.033	0.057
A0A140T8R7	Rcor1	1.049	0.069	0.246	0.303
A0A140T8R8	Dcun1d1	1.167	0.223	0.006	0.016
A0A140T8V9	Fyco1	0.956	(0.065)	0.208	0.263
A0A171KXD3	Prmt1	1.067	0.093	0.046	0.074
A0A1B0GQV4	Pih1d1	1.081	0.112	0.161	0.211
A0A1B0GQZ1	Mrpl23	1.044	0.062	0.277	0.336
A0A1B0GR08	Tubgcp2	1.007	0.009	0.897	0.916
A0A1B0GR54	Wrn	1.046	0.064	0.097	0.137
A0A1B0GR84	Tmem160	0.864	(0.211)	0.001	0.006
A0A1B0GRJ5	Gm45799	1.060	0.084	0.170	0.222
A0A1B0GRL4	Trak1	0.867	(0.206)	0.003	0.011
A0A1B0GRM0	Rbl2	0.942	(0.087)	0.112	0.155
A0A1B0GRU8	Prpf40a	1.090	0.124	0.001	0.007
A0A1B0GRV3	Rfx1	0.972	(0.041)	0.532	0.590
A0A1B0GRZ8	Elmod2	1.185	0.245	0.011	0.025
A0A1B0GSG3	Nsd3	0.924	(0.114)	0.015	0.032
A0A1B0GSK5	Rcn3	1.235	0.304	0.165	0.216
A0A1B0GSP9	Ing1	0.894	(0.161)	0.020	0.038
A0A1B0GSV1	Mrpl17	0.989	(0.017)	0.557	0.614
A0A1B0GSX7	Nup98	1.049	0.069	0.023	0.043
A0A1B0GT44	Prmt9	0.883	(0.180)	0.000	0.003

A0A1B0GT81	Bax	0.991	(0.013)	0.611	0.663
A0A1D5RLM8	Gm11639	0.921	(0.118)	0.000	0.004
A0A1D5RLS1	Eps15l1	0.923	(0.116)	0.001	0.007
A0A1D5RLU2	Fam192a	0.979	(0.030)	0.055	0.086
A0A1D5RLV1	Epb41	1.107	0.147	0.019	0.037
A0A1D5RM25	Sin3b	0.943	(0.084)	0.027	0.049
A0A1D5RM65	Rassf5	1.141	0.190	0.014	0.030
A0A1D5RM90	Tcf25	0.888	(0.172)	0.002	0.009
A0A1D5RM92	Wwp2	0.937	(0.094)	0.040	0.066
A0A1D5RMC8	Acsf3	1.002	0.002	0.965	0.969
A0A1D5RMG4	Agap3	0.938	(0.092)	0.307	0.368
A0A1I7Q4G8	Gatad2a	1.003	0.005	0.845	0.872
A0A1L1SQS9	Kmt2a	1.158	0.211	0.003	0.011
A0A1L1SS44	Aplp2	0.938	(0.093)	0.046	0.074
A0A1L1SS90	Acy1	0.941	(0.088)	0.138	0.184
A0A1L1SSE2	Cnot10	0.962	(0.056)	0.062	0.094
A0A1L1SSV8	Taok2	1.033	0.047	0.539	0.596
A0A1L1ST73	Senp8	1.177	0.235	0.018	0.035
A0A1L1STF0	Mindy2	0.849	(0.235)	0.002	0.008
A0A1L1STM0	Mtfr2	1.166	0.221	0.063	0.096
A0A1L1SU24	Mrpl4	1.016	0.023	0.390	0.450
A0A1L1SUQ2	Poc1a	0.877	(0.189)	0.008	0.019
A0A1L1SUS7	Cdk17	0.985	(0.021)	0.020	0.038
A0A1L1SUX8	Thy1	0.884	(0.178)	0.389	0.450
A0A1L1SV73	Usp47	0.937	(0.093)	0.056	0.087
A0A1L1SV84	Spc24	0.986	(0.021)	0.874	0.897
A0A1L1SVC7	Commd4	0.932	(0.101)	0.146	0.194
A0A1N9M4K1	Siae	0.992	(0.011)	0.795	0.829
A0A1W2P6R8	Hs1bp3	0.962	(0.056)	0.343	0.404
A0A1W2P6V6	Mdm1	0.986	(0.021)	0.643	0.693
A0A1W2P737	Pcnt	0.893	(0.164)	0.007	0.017
A0A1W2P794	Sppl2b	1.183	0.243	0.028	0.050
A0A1W2P7V0	Gopc	1.007	0.010	0.455	0.515
A0A1W2P7X0	Abracl	1.316	0.396	0.076	0.112
A0A1W2P7Z1	Pofut2	0.869	(0.203)	0.001	0.006
A0A1W2P8F6	Pik3cg	0.838	(0.254)	0.001	0.006
A0A1Y7VJ48	Tbca	0.965	(0.051)	0.383	0.444
A0A1Y7VJY8	Sirt5	0.887	(0.173)	0.208	0.263
A0A1Y7VKX7	Ddx24	1.056	0.079	0.303	0.363
A0A1Y7VKY1	Gm11361	1.037	0.053	0.392	0.452
A0A1Y7VLX9	Nrde2	0.983	(0.025)	0.683	0.730
A0A1Y7VM77	Rnaseh1	0.976	(0.035)	0.455	0.515
A0A1Y7VMH3	Vrk1	1.001	0.001	0.980	0.983
A0A1Y7VN66	Pole2	1.121	0.165	0.009	0.022
A0A1Y7VNF8	Kidins220	0.914	(0.130)	0.025	0.046
A0A1Y7VP67	Elmsan1	0.866	(0.207)	0.000	0.003
A0A1Y7VP73	Dtnbp1	0.867	(0.206)	0.009	0.022
A0A286YCI0	Ngly1	1.101	0.138	0.027	0.049
A0A286YCP6	Mbd1	1.025	0.036	0.682	0.730
A0A286YCX6	Trappc8	0.969	(0.046)	0.002	0.009
A0A286YD03	Amer2	0.922	(0.118)	0.090	0.128
A0A286YD15	Cetn3	1.070	0.098	0.107	0.149
A0A286YDA2	Nolc1	1.117	0.160	0.004	0.012
A0A286YDC0	Cdk7	1.070	0.097	0.021	0.040
A0A286YDF2	Vti1a	0.868	(0.205)	0.000	0.004
A0A286YDN1	Eml3	0.916	(0.127)	0.000	0.003
A0A286YDT0	Thoc7	1.065	0.091	0.033	0.057
A0A286YDT6	Btrc	0.910	(0.137)	0.015	0.031
A0A286YDX6	Kif13a	1.015	0.021	0.807	0.839
A0A286YE10	Grk6	0.882	(0.182)	0.000	0.004
A0A286YE16	Slc35b2	0.880	(0.185)	0.010	0.023

A0A286YEB7	Rps24	1.067	0.094	0.168	0.220
A0A2C9F2B5	Pxk	1.031	0.044	0.380	0.441
A0A2C9F2D2	Anxa7	0.958	(0.062)	0.347	0.407
A0A2I3BPE9	Tsc2	0.906	(0.142)	0.031	0.054
A0A2I3BPN6	Taf4	1.034	0.048	0.042	0.069
A0A2I3BQ16	Lrch1	0.916	(0.126)	0.037	0.062
A0A2I3BQ54	Mettl6	0.891	(0.167)	0.089	0.127
A0A2I3BQ91	Tmem260	0.940	(0.089)	0.005	0.014
A0A2I3BQA9	Wbp4	0.960	(0.059)	0.419	0.479
A0A2I3BQD7	Cog3	0.937	(0.094)	0.000	0.003
A0A2I3BQE3	Gnpnat1	0.906	(0.143)	0.036	0.061
A0A2I3BQS1	Dlgap5	1.096	0.132	0.069	0.103
A0A2I3BR94	Gmfb	1.099	0.136	0.014	0.029
A0A2I3BRI7	Tinf2	1.156	0.209	0.003	0.010
A0A2I3BRK6	Eny2	1.012	0.017	0.758	0.796
A0A2I3BRW0	Spcs1	1.083	0.115	0.197	0.251
A0A2K6EDK3	Nudcd1	0.871	(0.200)	0.003	0.011
A0A2R8VHC0	Naga	1.023	0.033	0.729	0.771
A0A2R8VHH1	Nup155	1.095	0.131	0.003	0.010
A0A2R8VHH6	Ptpra	0.973	(0.039)	0.161	0.211
A0A2R8VHX0	Csad	1.067	0.093	0.114	0.157
A0A2R8VJW0	Aco2	0.891	(0.166)	0.088	0.126
A0A2R8VK38	Cenpj	0.919	(0.122)	0.039	0.065
A0A2R8VKP7	Ccdc134	1.123	0.168	0.002	0.009
A0A2R8VKS6	Vps28	0.864	(0.211)	0.000	0.004
A0A2R8W6I7	Gm49510	0.872	(0.197)	0.002	0.007
A0A2R8W6L8	Hsf1	1.018	0.026	0.388	0.448
A0A2R8W6P0	Cd47	0.923	(0.116)	0.092	0.131
A0A2R8W6Y5	Larp4	1.078	0.108	0.136	0.183
A0A2R8W747	Zfp740	1.017	0.025	0.400	0.460
A0A2U3TZ82	Triobp	0.854	(0.228)	0.000	0.004
A0A338P6A9	Med15	1.091	0.126	0.020	0.039
A0A338P6B4	Polr2h	1.162	0.217	0.002	0.008
A0A338P6G6	Hmga1	1.027	0.039	0.802	0.836
A0A338P6K3	Zfp148	1.067	0.094	0.080	0.116
A0A338P6W1	Rbbp6	1.110	0.150	0.001	0.007
A0A338P7B7	Dynlt1a	1.013	0.019	0.136	0.183
A0A338P7E5	Ube2l3	1.050	0.070	0.197	0.251
A0A338P7L1	Smpd4	1.075	0.105	0.008	0.020
A0A3B2W864	Gm49804	1.049	0.069	0.321	0.382
A0A3B2WAS6	Ndc80	0.915	(0.129)	0.010	0.024
A0A3B2WC92	Spast	0.935	(0.097)	0.036	0.061
A0A3B2WCD8	Khsrp	1.048	0.068	0.002	0.009
A0A3B2WCL2	Tcp10c	1.010	0.014	0.359	0.420
A0A3B2WCL5	Ranbp3	1.012	0.018	0.491	0.549
A0A3B2WDD2	Rpl10a	1.109	0.150	0.048	0.076
A0A3F2YNL8	Memo1	1.140	0.189	0.001	0.007
A0A3Q4EBV4	Fam98a	1.160	0.214	0.000	0.003
A0A3Q4EBZ8	Thumpd2	1.152	0.204	0.019	0.037
A0A3Q4EG48	Kctd1	0.922	(0.117)	0.200	0.254
A0A3Q4EGA7	Nubp2	0.920	(0.120)	0.007	0.017
A0A3Q4EGQ6	Ss18	1.059	0.083	0.068	0.102
A0A3Q4EGT0	Commd10	0.957	(0.063)	0.096	0.136
A0A3Q4EHL9	Ammecr1l	0.908	(0.140)	0.102	0.143
A0A3Q4EHV9	Eml4	1.156	0.209	0.001	0.005
A0A494B952	Isoc1	1.020	0.029	0.217	0.273
A0A494B978	B3gat3	1.019	0.028	0.901	0.919
A0A494B9B9	Sdhaf2	0.995	(0.008)	0.925	0.938
A0A494B9C7	Rnaseh2c	0.945	(0.082)	0.095	0.135
A0A494B9P5	Nme4	0.890	(0.168)	0.027	0.049
A0A494B9U1	Zfp516	1.040	0.057	0.312	0.372

A0A494BA29	Cep78	0.879	(0.186)	0.006	0.017
A0A494BA34	Trub1	1.059	0.083	0.424	0.483
A0A494BA39	Nedd4l	1.027	0.038	0.123	0.168
A0A494BA44	Rela	0.928	(0.108)	0.005	0.014
A0A494BA87	Ciao3	0.889	(0.170)	0.245	0.301
A0A494BAJ6	Arl3	1.078	0.108	0.261	0.320
A0A494BAM2	Rcl1	1.114	0.156	0.000	0.004
A0A494BAQ2	Nfkb2	1.039	0.055	0.267	0.325
A0A494BAR0	Phf1	0.967	(0.048)	0.185	0.238
A0A494BB09	Cbwd1	0.894	(0.162)	0.209	0.264
A0A494BB10	Asdurf	0.965	(0.052)	0.396	0.456
A0A494BB58	Ccs	0.869	(0.203)	0.006	0.017
A0A494BB75	Wdr26	0.968	(0.047)	0.055	0.086
A0A494BB93	Cuedc2	1.051	0.072	0.189	0.242
A0A494BBA2	Sra1	0.879	(0.185)	0.017	0.034
A0A494BBF6	Stub1	0.897	(0.157)	0.017	0.034
A0A494BBL9	Tmem173	0.915	(0.129)	0.301	0.361
A0A498WGK2	Nars	1.195	0.258	0.001	0.005
A0A498WGK6	Tm9sf3	0.951	(0.072)	0.069	0.103
A1L314	Mpeg1	0.837	(0.256)	0.040	0.066
A2A432	Cul4b	1.083	0.115	0.010	0.024
A2A4A6	Plcg1	0.901	(0.150)	0.059	0.090
A2A4P0	Dhx8	0.858	(0.220)	0.004	0.013
A2A547	Rpl19	0.897	(0.157)	0.247	0.303
A2A5I7	Ttpal	1.173	0.231	0.002	0.007
A2A5L7	Cd40	0.921	(0.119)	0.079	0.115
A2A5R2	Arfgef2	0.879	(0.186)	0.001	0.006
A2A6A1	Gpatch8	1.019	0.027	0.090	0.129
A2A6J2	Tnnt3	1.035	0.049	0.931	0.943
A2A6J7	Lsp1	0.924	(0.114)	0.065	0.099
A2A6K0	Tnni2	0.965	(0.051)	0.920	0.934
A2A6Q5	Cdc27	0.968	(0.047)	0.157	0.206
A2A6S1	Tmem104	1.160	0.214	0.019	0.037
A2A6U3	Sept9	1.065	0.090	0.028	0.050
A2A7A7	H6pd	0.840	(0.251)	0.000	0.004
A2A7B5	Prdm2	1.011	0.016	0.625	0.676
A2A7G9	Fbxo6	0.967	(0.048)	0.234	0.290
A2A7S7	Yars	0.994	(0.008)	0.856	0.881
A2A9C6	Med8	0.861	(0.216)	0.004	0.012
A2A9I0	Gosr2	0.924	(0.114)	0.051	0.081
A2A9W3	Mif4gd	0.884	(0.178)	0.001	0.007
A2A9X5	Nt5c	1.116	0.158	0.002	0.008
A2AA85	Szrd1	0.918	(0.123)	0.046	0.074
A2AAN0	Exoc7	1.010	0.014	0.525	0.583
A2AB59	Arhgap27	0.883	(0.180)	0.003	0.011
A2AB97	932438A13R	1.014	0.019	0.771	0.808
A2ABV5	Med14	0.932	(0.101)	0.002	0.009
A2ABY3	Pcyt2	0.880	(0.184)	0.013	0.028
A2AC29	Ebp	0.855	(0.226)	0.000	0.004
A2ACG7	Rpn2	1.156	0.209	0.007	0.017
A2ACM0	Rptor	0.967	(0.049)	0.098	0.138
A2ADB1	Spen	0.939	(0.090)	0.001	0.006
A2ADR8	Ppp1r8	1.109	0.149	0.001	0.006
A2ADY9	Ddi2	0.865	(0.209)	0.053	0.083
A2AE98	Wdtc1	0.842	(0.249)	0.001	0.005
A2AEM2	Clcc1	0.894	(0.162)	0.000	0.004
A2AES3	Otud5	0.913	(0.132)	0.169	0.220
A2AF47	Dock11	1.022	0.031	0.269	0.328
A2AFF6	Stag2	1.064	0.090	0.001	0.005
A2AFG7	L1cam	0.836	(0.259)	0.005	0.014
A2AFQ0	Huwe1	0.948	(0.077)	0.040	0.066

A2AFQ2	Hsd17b10	1.119	0.163	0.007	0.018
A2AFQ9	Gemin5	1.066	0.092	0.001	0.007
A2AGH8	Med12	0.955	(0.067)	0.033	0.057
A2AGS6	Trp53i11	0.949	(0.076)	0.250	0.307
A2AHX8	Bcl2l1	0.961	(0.058)	0.251	0.308
A2AIG8	Accs	0.864	(0.211)	0.009	0.021
A2AIM4	Tpm2	0.964	(0.053)	0.769	0.806
A2AIV2	Virma	1.027	0.038	0.096	0.136
A2AIV6	Snapc4	1.531	0.614	0.116	0.159
A2AJ15	Man1b1	0.862	(0.215)	0.002	0.009
A2AJ72	Fubp3	1.052	0.073	0.024	0.044
A2AJ88	Pnpla7	0.927	(0.109)	0.068	0.101
A2AJG0	Rpp38	1.081	0.113	0.003	0.011
A2AJI1	Map7d1	0.881	(0.183)	0.030	0.053
A2AJT5	Pnisr	0.971	(0.043)	0.047	0.076
A2AKV7	Alkbh3	1.078	0.109	0.089	0.127
A2ALA4	Med22	0.936	(0.096)	0.021	0.040
A2ALB1	Rexo4	1.085	0.118	0.218	0.274
A2ALR9	Usp48	1.158	0.212	0.001	0.006
A2AMC3	Pofut1	0.886	(0.174)	0.010	0.023
A2AMW0	Capzb	0.990	(0.014)	0.430	0.490
A2AMY5	Ubap2	0.923	(0.116)	0.137	0.184
A2AN08	Ubr4	0.899	(0.154)	0.000	0.005
A2AN91	Susd1	0.921	(0.119)	0.064	0.097
A2ANX3	Atp8b4	0.953	(0.070)	0.002	0.008
A2ANY6	Mdn1	0.992	(0.012)	0.605	0.658
A2AP32	Ndufb6	1.027	0.038	0.438	0.497
A2AP93	Map3k7	1.032	0.046	0.221	0.277
A2APB8	Tpx2	1.066	0.092	0.037	0.062
A2APF7	Zbp1	0.860	(0.217)	0.226	0.282
A2API3	Ints8	1.011	0.016	0.483	0.542
A2APY7	Ndufaf5	0.852	(0.232)	0.000	0.004
A2AQ17	Ndufaf1	0.976	(0.035)	0.120	0.165
A2AQ19	Rtf1	1.091	0.125	0.001	0.007
A2AQ43	Fnbp1	0.973	(0.039)	0.198	0.252
A2AQA7	Aqr	1.055	0.077	0.000	0.003
A2AQE4	Cops2	0.916	(0.126)	0.001	0.007
A2AQY8	Kyat1	1.003	0.004	0.894	0.914
A2AR02	Ppig	1.011	0.016	0.461	0.520
A2ARJ0	Stam	0.986	(0.021)	0.858	0.882
A2AS05	Helz2	0.924	(0.115)	0.522	0.580
A2AS70	Mllt10	0.976	(0.035)	0.536	0.593
A2AT37	Upf2	1.062	0.087	0.310	0.370
A2ATI9	Gorasp2	0.861	(0.216)	0.003	0.011
A2ATY4	Zbtb34	0.997	(0.005)	0.914	0.929
A2AU52	Commd7	1.114	0.156	0.034	0.058
A2AU62	Raly	1.090	0.124	0.111	0.154
A2AUE1	Dnajc5	0.909	(0.138)	0.017	0.035
A2AUF7	Rabepk	0.951	(0.072)	0.195	0.249
A2AUM1	Eif2ak4	0.873	(0.195)	0.001	0.007
A2AUM9	Cep152	0.996	(0.005)	0.818	0.849
A2AUT1	Spc25	0.994	(0.009)	0.812	0.844
A2AUY4	Baz2b	0.992	(0.012)	0.584	0.639
A2AVP3	Pdcl	0.866	(0.208)	0.034	0.058
A2AVZ9	Slc43a3	0.934	(0.099)	0.114	0.158
A2AWA9	Rabgap1	1.075	0.104	0.004	0.013
A2AWF8	Ptprj	1.002	0.003	0.955	0.962
A2AWI9	Sh3glb2	1.082	0.113	0.005	0.014
A2AWS5	Hdac5	0.993	(0.010)	0.856	0.881
A2AWT6	Ubtf	1.170	0.227	0.021	0.039
A2BDX3	Mocs3	0.962	(0.056)	0.267	0.326

A2BE28	Las1l	1.006	0.008	0.595	0.649
A2BFF8	Dync1i2	0.944	(0.083)	0.197	0.251
A2BGN7	Manbal	0.902	(0.148)	0.325	0.385
A2BH40	Arid1a	0.935	(0.098)	0.002	0.009
A2BHN6	Fbxo3	1.010	0.015	0.632	0.682
A2BI12	Psip1	1.205	0.269	0.062	0.095
A2CG63	Arid4b	1.112	0.153	0.011	0.026
A2CG76	Ehmt2	0.988	(0.017)	0.480	0.539
A2RSY1	Kansl3	1.081	0.112	0.050	0.080
A2RSY6	Trmt1l	0.971	(0.043)	0.091	0.130
A2RT67	Dennd3	0.896	(0.159)	0.002	0.009
A2RTH5	Lcmt1	1.095	0.130	0.129	0.174
A3KG40	Ikbkg	1.081	0.113	0.025	0.045
A3KGG8	Knstrn	0.923	(0.116)	0.083	0.120
A3KGV1	Odf2	0.978	(0.033)	0.158	0.208
A6H5Y3	Mtr	1.159	0.213	0.011	0.024
A6H611	Mipep	0.868	(0.204)	0.025	0.045
A6H630	Armt1	0.973	(0.040)	0.066	0.100
A6H6A9	Rabgap1l	1.162	0.216	0.027	0.049
A6H8H2	Dennd4c	0.940	(0.090)	0.186	0.239
A6PW28	Ripor2	0.867	(0.207)	0.001	0.006
A6PWP8	Madd	0.923	(0.115)	0.015	0.032
A6PWQ1	Cenps	0.895	(0.160)	0.044	0.072
A6PWS5	Gsn	0.858	(0.221)	0.022	0.041
A6XA75	Treml1	0.901	(0.151)	0.344	0.405
A7UAK4	Pfkfb3	0.946	(0.080)	0.002	0.008
A8C756	Thada	0.946	(0.080)	0.283	0.342
A8Y5J3	Ints11	0.954	(0.068)	0.062	0.094
B0QZL5	Pik3cd	0.853	(0.229)	0.002	0.009
B0QZM2	Ube2j2	0.919	(0.121)	0.060	0.093
B0QZX1	Il2rg	0.923	(0.116)	0.345	0.406
B0QZX9	Dcakd	0.835	(0.261)	0.003	0.010
B0V2Q9	Lclat1	0.958	(0.063)	0.269	0.328
B0V3P3	Ap4b1	0.857	(0.223)	0.008	0.019
B1APX2	)31439G07R	1.115	0.158	0.012	0.027
B1AQH6	Med1	1.042	0.060	0.315	0.375
B1AR17	Chd3	0.980	(0.029)	0.601	0.654
B1AR50	Gabarap	0.913	(0.132)	0.001	0.006
B1ARD6	Slfn9	1.027	0.038	0.375	0.436
B1ARG9	Smg6	1.086	0.118	0.016	0.033
B1ART2	Vps13d	0.855	(0.225)	0.007	0.018
B1ARW4	Ndufs5	1.018	0.026	0.466	0.525
B1ASP2	Jak1	0.890	(0.167)	0.014	0.029
B1ASW5	Trappc1	0.872	(0.197)	0.001	0.007
B1AT03	Lig3	0.886	(0.175)	0.003	0.010
B1ATB3	Tmc6	1.062	0.086	0.101	0.142
B1ATI9	Gas7	1.046	0.065	0.098	0.138
B1ATS5	Atp2a3	0.995	(0.007)	0.862	0.886
B1ATZ0	Hgs	0.875	(0.192)	0.002	0.008
B1AU25	Aifm1	1.191	0.253	0.000	0.003
B1AU74	Mospd2	0.852	(0.232)	0.000	0.003
B1AUB6	Nfia	0.876	(0.191)	0.310	0.370
B1AUN3	Eif2b3	0.875	(0.192)	0.029	0.052
B1AUQ7	Cetn2	0.983	(0.024)	0.462	0.521
B1AVF2	Tsc22d3	1.007	0.010	0.946	0.954
B1AVH5	Coro2a	0.963	(0.054)	0.178	0.230
B1AW40	Arhgap15	0.864	(0.211)	0.001	0.006
B1AWE0	Clta	1.097	0.134	0.007	0.017
B1AWI3	Gba2	1.211	0.276	0.068	0.102
B1AXI9	Cep131	0.950	(0.074)	0.015	0.031
B1AXN9	Rps6ka3	1.071	0.099	0.004	0.012

B1AXP6	Tomm5	1.009	0.012	0.778	0.814
B1AY13	Usp24	0.885	(0.177)	0.001	0.006
B1AZ42	Chmp6	0.894	(0.162)	0.000	0.004
B1AZA5	Tmem245	0.904	(0.145)	0.001	0.005
B1AZI6	Thoc2	1.085	0.118	0.002	0.009
B1B0E9	Zrsr2	0.889	(0.169)	0.008	0.019
B2KGR2	Lancl1	1.075	0.105	0.349	0.409
B2M1R6	Hnrnpk	1.065	0.091	0.006	0.015
B2RQC7	Dip2b	0.972	(0.040)	0.140	0.188
B2RQG2	Phf3	1.097	0.134	0.015	0.031
B2RR83	Ythdc2	1.190	0.251	0.013	0.028
B2RT41	Zfc3h1	1.063	0.088	0.034	0.059
B2RUJ2	Erbin	0.971	(0.043)	0.227	0.283
B2RUR8	Otud7b	1.007	0.010	0.569	0.625
B2RWS6	Ep300	0.942	(0.086)	0.000	0.003
B2RXC1	Trappc11	1.137	0.185	0.026	0.047
B2RXC2	Itpkb	0.985	(0.022)	0.681	0.728
B2RXM2	Gm6793	1.015	0.022	0.465	0.524
B2RXP1	Lrch3	1.122	0.167	0.002	0.009
B2RXS4	Plxnb2	1.051	0.072	0.188	0.241
B2RXW8	Ppfia1	1.033	0.047	0.579	0.635
B2RY04	Dock5	0.852	(0.231)	0.001	0.006
B2RY56	Rbm25	1.152	0.204	0.001	0.007
B2Z894	Gm29427	0.883	(0.179)	0.005	0.014
B5B2U1	Nfatc3	1.216	0.282	0.109	0.152
B7FAU9	Flna	0.862	(0.215)	0.012	0.026
B7ZC85	Tlk2	1.032	0.045	0.626	0.676
B7ZCU2	Abi1	1.008	0.012	0.671	0.719
B7ZCV4	Coq4	0.956	(0.065)	0.268	0.326
B7ZMP1	Xpnpep3	1.068	0.095	0.051	0.080
B7ZNP0	Edem3	0.901	(0.150)	0.011	0.024
B7ZP47	Wapl	0.943	(0.084)	0.003	0.010
B7ZWL1	Cnot1	1.021	0.030	0.106	0.147
B8JJC1	Tdp1	1.002	0.003	0.953	0.960
B9EHX4	Atm	0.837	(0.256)	0.009	0.022
B9EJR8	Dnaaf5	0.935	(0.097)	0.023	0.044
B9EKS2	Kdm3b	1.064	0.090	0.013	0.028
C0HKD9	Mfap1b	1.065	0.091	0.104	0.146
D3YTT4	Acad8	0.881	(0.183)	0.004	0.012
D3YTU2	Ogfod1	1.103	0.142	0.118	0.162
D3YTU7	Sike1	0.966	(0.050)	0.043	0.070
D3YTZ7	Josd2	1.009	0.013	0.704	0.749
D3YUB9	Pabpn1	1.170	0.226	0.044	0.071
D3YUP1	Carm1	0.898	(0.156)	0.008	0.019
D3YV77	Mvk	0.743	(0.428)	0.056	0.087
D3YVI7	Trim30b	0.724	(0.466)	0.423	0.483
D3YVM5	Rplp0	1.076	0.106	0.296	0.356
D3YVQ5	Sirt3	0.970	(0.044)	0.815	0.847
D3YVU1	Usp16	0.870	(0.201)	0.009	0.022
D3YVU4	Kdm2b	1.039	0.056	0.103	0.144
D3YWR1	Gnpda2	1.071	0.098	0.136	0.183
D3YWR7	Qdpr	0.929	(0.106)	0.054	0.085
D3YWT0	Sec11a	1.176	0.234	0.000	0.003
D3YWT1	Hnrnph3	1.119	0.162	0.010	0.023
D3YWU4	Spns1	0.873	(0.196)	0.014	0.030
D3YWX2	Ylpm1	1.022	0.032	0.126	0.171
D3YWY6	Mpc1	1.099	0.137	0.392	0.453
D3YX13	Ing3	0.989	(0.015)	0.624	0.675
D3YXA2	Dbr1	0.986	(0.020)	0.525	0.582
D3YXQ1	Tceanc2	1.125	0.170	0.016	0.033
D3YY07	Zdhhc13	0.849	(0.236)	0.034	0.058

D3YY50	Cutc	1.015	0.021	0.347	0.408
D3YY63	Pisd	0.886	(0.175)	0.161	0.211
D3YY85	Zmynd11	0.946	(0.080)	0.043	0.071
D3YYA0	Ash2l	1.148	0.199	0.000	0.004
D3YYD2	Fam111a	0.867	(0.207)	0.002	0.008
D3YYH5	Pqlc1	0.978	(0.032)	0.682	0.729
D3YYK8	Mapre2	0.900	(0.152)	0.016	0.033
D3YZ09	Gar1	1.146	0.197	0.081	0.118
D3YZ20	Polr2j	1.141	0.190	0.021	0.040
D3YZ54	Hacl1	0.865	(0.210)	0.000	0.005
D3YZ99	Ntan1	0.899	(0.154)	0.001	0.006
D3YZC7	Brd8	1.051	0.072	0.042	0.069
D3YZD6	Cryzl1	0.881	(0.182)	0.008	0.019
D3YZP9	Ccdc6	1.028	0.040	0.272	0.330
D3Z041	Acsl1	1.026	0.037	0.343	0.404
D3Z061	Uba6	1.077	0.106	0.013	0.028
D3Z0B9	Aldh16a1	0.961	(0.058)	0.014	0.029
D3Z0L8	Actn4	0.986	(0.021)	0.862	0.886
D3Z0M9	Ddx23	1.131	0.178	0.020	0.038
D3Z0T0	Taf6	1.081	0.112	0.000	0.003
D3Z0V2	Arhgef7	0.943	(0.084)	0.009	0.022
D3Z131	Thoc6	1.158	0.212	0.030	0.053
D3Z191	Tmem106b	1.040	0.057	0.203	0.257
D3Z1G7	Mmab	1.068	0.094	0.002	0.007
D3Z1T8	Ttc33	1.066	0.092	0.016	0.033
D3Z223	Bpgm	1.408	0.494	0.052	0.082
D3Z2B3	Acad12	0.983	(0.025)	0.457	0.517
D3Z2E3	Reps1	0.908	(0.139)	0.016	0.033
D3Z2E7	Ifi207	0.844	(0.244)	0.333	0.394
D3Z2H7	Ctnnd1	1.037	0.053	0.164	0.214
D3Z2H9	Tpm3-rs7	0.850	(0.235)	0.001	0.006
D3Z2J6	Tmx2	1.082	0.114	0.421	0.480
D3Z2K2	Mrps14	0.878	(0.188)	0.135	0.182
D3Z2Q2	Stxbp5	0.898	(0.155)	0.001	0.006
D3Z2W0	Tnip1	0.983	(0.024)	0.491	0.549
D3Z358	Nfkbib	0.968	(0.048)	0.061	0.094
D3Z3A0	Ppp1r2	0.873	(0.196)	0.004	0.013
D3Z3C3	Hpse	0.949	(0.075)	0.328	0.389
D3Z3E8	Mov10	0.878	(0.187)	0.168	0.219
D3Z3G6	Mapk3	1.099	0.136	0.064	0.096
D3Z3L3	Trim12c	0.900	(0.152)	0.078	0.114
D3Z440	Cops7a	1.109	0.149	0.005	0.014
D3Z497	Gm7075	0.917	(0.125)	0.013	0.028
D3Z4C3	Zbtb7a	1.124	0.169	0.003	0.010
D3Z4D1	Jtb	0.953	(0.069)	0.159	0.208
D3Z4I3	Rbm24	1.054	0.076	0.132	0.178
D3Z4S3	Ptrhd1	1.185	0.245	0.000	0.004
D3Z4T3	Psph	0.870	(0.201)	0.019	0.037
D3Z4U0	Zranb2	1.047	0.066	0.111	0.153
D3Z4U9	Irf7	0.913	(0.131)	0.705	0.749
D3Z5I1	Zc3hav1	1.003	0.005	0.822	0.852
D3Z5I5	Eps8	0.986	(0.021)	0.909	0.925
D3Z5I9	Rbm33	1.001	0.002	0.973	0.976
D3Z5N5	Pikfyve	0.967	(0.048)	0.087	0.126
D3Z5P5	Ighmbp2	0.938	(0.092)	0.220	0.276
D3Z5P7	Cdca2	0.963	(0.055)	0.228	0.284
D3Z5V3	Knop1	1.135	0.182	0.023	0.043
D3Z5W7	Gstt1	0.862	(0.214)	0.086	0.124
D3Z600	Pex5	0.976	(0.036)	0.601	0.654
D3Z627	Itgal	0.883	(0.180)	0.003	0.010
D3Z645	Vps29	0.936	(0.096)	0.006	0.015

D3Z653	Atg16l2	0.848	(0.238)	0.013	0.028
D3Z6H3	Dctn6	0.929	(0.107)	0.040	0.067
D3Z6S1	Tmem214	0.892	(0.165)	0.028	0.050
D3Z6W2	Ptpn2	0.916	(0.126)	0.027	0.049
D3Z712	Rps15a	1.175	0.233	0.088	0.127
D3Z780	Eif2b4	0.970	(0.044)	0.782	0.817
D3Z7J8	Ccdc61	0.966	(0.050)	0.717	0.760
D3Z7P3	Gls	1.069	0.096	0.053	0.084
D3Z7X7	Tpd52	0.927	(0.109)	0.242	0.299
D6RDC1	Tmem41b	0.977	(0.034)	0.685	0.731
D6RDC6	Gtpbp3	1.171	0.228	0.002	0.007
D6REG4	Grhpr	0.930	(0.105)	0.067	0.100
D6RET6	Gpatch1	1.015	0.022	0.554	0.610
D6RFU2	Abhd12	0.995	(0.008)	0.846	0.873
D6RG44	Tmem126a	1.020	0.028	0.676	0.724
D6RG56	Rsbn1	1.069	0.096	0.144	0.192
D6RH37	Pkn1	0.940	(0.090)	0.025	0.045
D6RI95	Slc29a3	1.053	0.074	0.069	0.103
D6RJ84	Uckl1	1.146	0.197	0.011	0.024
E0CXC7	Sidt2	0.934	(0.098)	0.035	0.060
E0CXD9	Surf4	0.875	(0.193)	0.033	0.058
E0CXE7	Ctc1	0.870	(0.201)	0.012	0.027
E0CXV5	Gtf2h4	1.134	0.181	0.010	0.023
E0CXW6	Olr1	0.837	(0.256)	0.002	0.009
E0CXZ5	Samhd1	0.884	(0.179)	0.024	0.045
E0CY18	Zfand2b	0.862	(0.213)	0.032	0.056
E0CYD7	Unkl	1.002	0.003	0.939	0.948
E0CYE2	Impdh1	1.009	0.012	0.803	0.837
E0CYH0	Wtap	1.061	0.086	0.000	0.004
E0CYI3	Mff	1.174	0.232	0.022	0.042
E0CYI9	Gcc2	0.876	(0.191)	0.000	0.005
E0CYJ0	Mob4	0.895	(0.160)	0.001	0.006
E0CYK6	Hddc2	1.120	0.163	0.139	0.185
E0CYU9	Znrd2	1.022	0.031	0.403	0.463
E0CYW1	Ubl5	1.185	0.245	0.014	0.030
E0CZ22	Mroh1	0.854	(0.228)	0.000	0.003
E0CZ28	Lrmda	0.937	(0.094)	0.139	0.186
E0CZ61	Umad1	0.866	(0.208)	0.049	0.078
E0CZ69	Fgd4	0.858	(0.222)	0.009	0.021
E0CZ72	Kif2a	1.123	0.167	0.008	0.019
E0CZ78	Ppp3cb	0.912	(0.134)	0.018	0.036
E0CZE0	Nae1	0.988	(0.017)	0.249	0.307
E0CZF3	Alg9	0.845	(0.244)	0.013	0.028
E9PUA2	Tut7	0.928	(0.107)	0.001	0.006
E9PUA5	Kifc5b	1.042	0.060	0.040	0.066
E9PUB7	Msto1	0.955	(0.066)	0.291	0.350
E9PUD2	Dnm1l	0.986	(0.021)	0.667	0.715
E9PUE7	Abr	0.895	(0.161)	0.001	0.006
E9PUF7	Arhgef1	0.906	(0.143)	0.003	0.011
E9PUM4	Tln2	0.877	(0.189)	0.104	0.145
E9PV24	Fga	0.856	(0.224)	0.029	0.052
E9PV28	Lin54	0.917	(0.125)	0.061	0.093
E9PV44	Atpif1	1.145	0.195	0.008	0.019
E9PV48	Ifit3b	1.222	0.290	0.652	0.702
E9PV69	Cacul1	1.174	0.232	0.001	0.007
E9PVA6	Git2	1.005	0.007	0.809	0.841
E9PVA8	Gcn1	0.917	(0.125)	0.010	0.022
E9PVS0	Ifrd1	1.047	0.066	0.515	0.573
E9PVX5	Hltf	0.850	(0.235)	0.002	0.008
E9PVZ8	Golgb1	0.850	(0.234)	0.000	0.004
E9PW03	Gpaa1	0.921	(0.119)	0.041	0.068

E9PWG6	Ncapg	1.092	0.127	0.001	0.005
E9PWT4	H2-Q7	0.915	(0.128)	0.306	0.366
E9PWV3	Rps6ka1	0.964	(0.053)	0.248	0.305
E9PWW6	Zc3h7a	0.920	(0.120)	0.009	0.021
E9PWW9	Rsf1	1.076	0.105	0.003	0.011
E9PWX0	Med16	1.010	0.015	0.603	0.656
E9PWY7	Slc29a1	1.149	0.201	0.004	0.011
E9PX53	Ppp4r1	0.925	(0.112)	0.031	0.054
E9PX94	Cc2d1a	0.867	(0.207)	0.001	0.007
E9PXC2	Usf2	0.835	(0.260)	0.003	0.011
E9PXE6	Fmnl2	1.014	0.020	0.724	0.766
E9PXG9	Vps53	0.867	(0.206)	0.003	0.010
E9PXV7	Diaph1	0.897	(0.156)	0.001	0.006
E9PY16	Adap1	1.019	0.027	0.393	0.453
E9PY43	Erich1	0.931	(0.104)	0.107	0.149
E9PYF7	Ap1ar	0.927	(0.110)	0.042	0.069
E9PYG6	Rasa1	0.976	(0.034)	0.429	0.488
E9PYH6	Setd1a	1.181	0.239	0.002	0.007
E9PYI3	Plcb2	0.877	(0.189)	0.003	0.011
E9PYI8	Usp14	0.935	(0.096)	0.051	0.080
E9PYJ6	Focad	1.014	0.020	0.685	0.731
E9PYK3	Parp4	0.868	(0.204)	0.002	0.009
E9PYL4	Clcn7	1.017	0.024	0.765	0.802
E9PYS6	Arhgap4	0.977	(0.034)	0.259	0.317
E9PYT3	Atl3	1.005	0.008	0.822	0.852
E9PZ00	Psap	0.874	(0.195)	0.003	0.010
E9PZ16	Hspg2	0.993	(0.010)	0.877	0.899
E9PZ41	Trappc9	1.090	0.125	0.062	0.094
E9PZ88	Man2c1	0.954	(0.068)	0.004	0.012
E9PZA4	Pnpla2	0.911	(0.135)	0.002	0.008
E9PZA7	Trrap	1.008	0.012	0.319	0.380
E9PZF0	Gm20390	0.877	(0.190)	0.026	0.047
E9PZJ8	Ascc3	1.043	0.060	0.135	0.182
E9PZL9	Uvssa	0.912	(0.133)	0.002	0.008
E9PZM4	Chd2	0.933	(0.100)	0.009	0.021
E9PZM7	Scaf11	1.117	0.160	0.003	0.010
E9PZP8	Herc1	0.911	(0.134)	0.037	0.062
E9Q026	C2cd5	0.944	(0.084)	0.001	0.006
E9Q035	Gm20425	1.144	0.194	0.048	0.076
E9Q0A7	Oxr1	0.857	(0.223)	0.004	0.012
E9Q0G1	Denr	0.965	(0.052)	0.366	0.427
E9Q0X9	Mettl17	1.049	0.069	0.339	0.399
E9Q0Y4	Sipa1	0.958	(0.062)	0.136	0.183
E9Q0Z7	Sumf1	0.923	(0.115)	0.025	0.045
E9Q109	Nip7	0.959	(0.060)	0.423	0.483
E9Q137	Tex264	0.967	(0.048)	0.345	0.406
E9Q166	Atad2b	1.010	0.015	0.244	0.301
E9Q175	Myo6	1.022	0.031	0.746	0.785
E9Q1I2	Nabp2	0.943	(0.085)	0.098	0.139
E9Q1M6	Ankhd1	1.119	0.162	0.000	0.004
E9Q1P8	Irf2bp2	0.932	(0.101)	0.121	0.165
E9Q1S3	Sec23a	0.930	(0.105)	0.004	0.012
E9Q1U8	Trim24	1.059	0.083	0.107	0.149
E9Q236	Abcc4	0.991	(0.013)	0.565	0.621
E9Q242	Adsl	1.011	0.015	0.233	0.289
E9Q2A6	Ptk2b	0.987	(0.018)	0.053	0.084
E9Q2D4	Cxcr4	1.073	0.102	0.318	0.378
E9Q2E4	Hectd4	0.943	(0.084)	0.071	0.105
E9Q2H1	Ubr5	1.073	0.102	0.014	0.029
E9Q2H8	Hagh	0.952	(0.072)	0.035	0.060
E9Q2M9	Wdfy4	1.179	0.238	0.001	0.007

E9Q2S4	Pramef6	0.709	(0.495)	0.178	0.231
E9Q2S9	Ccdc43	1.005	0.006	0.938	0.948
E9Q2X6	Smc4	1.055	0.077	0.045	0.074
E9Q309	Cep350	1.042	0.059	0.605	0.658
E9Q361	Ints10	1.168	0.224	0.001	0.007
E9Q3G8	Nup153	1.123	0.168	0.013	0.029
E9Q3L2	Pi4ka	0.978	(0.032)	0.025	0.046
E9Q3M0	Smad2	1.058	0.082	0.018	0.035
E9Q3N0	Recql	1.049	0.070	0.078	0.114
E9Q3T0	Gm10073	1.098	0.135	0.115	0.158
E9Q3T3	Kif14	0.984	(0.024)	0.505	0.563
E9Q411	Nbas	0.865	(0.209)	0.000	0.004
E9Q446	Rnf25	0.931	(0.103)	0.071	0.106
E9Q450	Tpm1	0.976	(0.035)	0.442	0.503
E9Q452	Tpm1	0.981	(0.027)	0.895	0.915
E9Q489	Casq1	0.866	(0.208)	0.668	0.716
E9Q496	Use1	1.029	0.042	0.113	0.156
E9Q4F7	Ankrd11	1.090	0.124	0.152	0.200
E9Q4H1	Apc	1.022	0.031	0.679	0.727
E9Q4K7	Kif13b	0.950	(0.074)	0.031	0.054
E9Q4M2	Lipe	1.115	0.157	0.091	0.130
E9Q4M4	Chchd6	1.107	0.146	0.029	0.052
E9Q4N6	Arid1b	1.121	0.164	0.019	0.037
E9Q4P0	Kxd1	0.880	(0.184)	0.015	0.031
E9Q4Q2	Sf1	0.990	(0.014)	0.228	0.284
E9Q4Y4	Cep192	1.000	0.000	0.992	0.993
E9Q555	Rnf213	0.966	(0.050)	0.714	0.758
E9Q577	Uimc1	1.015	0.021	0.284	0.343
E9Q585	Slc4a1ap	1.013	0.018	0.353	0.414
E9Q586	Dctn1	1.011	0.016	0.533	0.590
E9Q5D6	Ranbp9	0.836	(0.258)	0.003	0.010
E9Q5F9	Setd2	0.973	(0.040)	0.036	0.061
E9Q5G3	Kif23	1.120	0.164	0.004	0.011
E9Q5J9	Tpm3	1.148	0.200	0.583	0.638
E9Q5K9	Ythdc1	0.886	(0.175)	0.082	0.119
E9Q5W5	Zzef1	0.895	(0.159)	0.002	0.008
E9Q6A7	Bptf	0.942	(0.087)	0.001	0.006
E9Q6H6	Alox5	0.979	(0.031)	0.502	0.561
E9Q6I2	Il1rap	0.972	(0.041)	0.426	0.485
E9Q6J5	Bod1l	1.060	0.085	0.030	0.053
E9Q6K1	Ercc2	1.081	0.112	0.003	0.010
E9Q6P4	Tubgcp6	1.089	0.124	0.068	0.102
E9Q6R7	Utrn	0.876	(0.191)	0.000	0.003
E9Q6U4	Pfdn4	0.962	(0.055)	0.330	0.391
E9Q784	Zc3h13	1.027	0.038	0.457	0.517
E9Q7A8	Eci2	1.062	0.087	0.053	0.083
E9Q7C3	Plekhg4	0.861	(0.215)	0.009	0.022
E9Q7E2	Arid2	1.185	0.245	0.001	0.005
E9Q7G0	Numa1	1.108	0.148	0.002	0.009
E9Q7H6	Tars2	1.079	0.109	0.001	0.006
E9Q7L3	Pbrm1	1.188	0.249	0.001	0.005
E9Q7Q3	Tpm3	1.050	0.071	0.057	0.088
E9Q855	Scamp3	0.893	(0.164)	0.020	0.039
E9Q8D5	Cnot2	1.059	0.083	0.040	0.066
E9Q8I7	Nfxl1	0.911	(0.134)	0.009	0.020
E9Q8N5	Clasp2	1.016	0.022	0.458	0.518
E9Q8T7	Dnah1	1.073	0.102	0.524	0.582
E9Q8V6	Dennd4a	0.862	(0.215)	0.015	0.031
E9Q919	Dctn3	0.998	(0.002)	0.949	0.956
E9Q980	Zfp788	0.949	(0.076)	0.144	0.192
E9Q9E4	Cdk4	1.119	0.163	0.013	0.028

E9Q9M5	Usp19	1.053	0.075	0.066	0.099
E9Q9T6	Ddx55	0.874	(0.195)	0.021	0.040
E9Q9V3	Ncor2	0.991	(0.013)	0.544	0.600
E9QA63	Macf1	0.942	(0.086)	0.005	0.014
E9QAE3	Btaf1	0.982	(0.026)	0.597	0.651
E9QAG2	Phf6	1.156	0.209	0.009	0.021
E9QAI5	Cad	1.044	0.063	0.090	0.129
E9QAS4	Chd4	1.050	0.070	0.001	0.005
E9QAT0	Fmr1	1.160	0.215	0.004	0.012
E9QAT4	Sec16a	0.889	(0.170)	0.000	0.002
E9QAY5	Gm37240	1.010	0.014	0.754	0.792
E9QB01	Ncam1	1.049	0.069	0.313	0.374
E9QJS0	Mrps10	0.924	(0.114)	0.038	0.064
E9QJS1	Tyk2	0.963	(0.054)	0.128	0.174
E9QJV4	Prpf39	1.096	0.132	0.003	0.010
E9QK89	Mdc1	1.066	0.092	0.007	0.018
E9QKA4	Srrm1	0.975	(0.036)	0.047	0.075
E9QKE9	Cux1	0.868	(0.204)	0.049	0.078
E9QKG3	Trmt11	0.931	(0.103)	0.005	0.014
E9QKG6	Ankrd17	0.957	(0.064)	0.184	0.237
E9QKK8	Atp11c	0.869	(0.203)	0.001	0.006
E9QKL6	Ifi204	0.866	(0.207)	0.488	0.546
E9QKV6	Myo9b	0.942	(0.086)	0.005	0.014
E9QKZ2	Ipo9	1.007	0.010	0.670	0.719
E9QL53	Cit	0.947	(0.079)	0.015	0.031
E9QLB8	Coq8b	0.921	(0.118)	0.060	0.092
E9QLL2	Dnm3	1.198	0.261	0.001	0.007
E9QLM4	Pms2	1.041	0.057	0.278	0.336
E9QLQ7	Rab29	0.883	(0.180)	0.000	0.004
E9QLX1	Cd1d2	1.151	0.203	0.000	0.004
E9QM76	Pgpep1l	0.884	(0.177)	0.193	0.247
E9QM80	Cbfa2t3	1.091	0.126	0.061	0.094
E9QME5	Trim33	0.966	(0.050)	0.044	0.072
E9QMJ5	Adgre5	0.895	(0.160)	0.011	0.025
E9QMX7	Arhgap30	0.921	(0.119)	0.017	0.035
E9QN31	Nop2	1.128	0.174	0.007	0.018
E9QNH6	Myo1b	0.932	(0.101)	0.194	0.248
E9QNQ4	Rapgef2	1.021	0.030	0.699	0.744
E9QP49	Ehbp1l1	1.003	0.004	0.801	0.835
E9QP59	Lemd3	0.984	(0.023)	0.517	0.574
E9QPU1	Vwf	0.898	(0.156)	0.280	0.339
E9QQ99	Syvn1	0.995	(0.007)	0.843	0.870
F2Z400	Pdpk1	0.939	(0.090)	0.002	0.007
F2Z471	Vdac1	1.079	0.110	0.155	0.205
F2Z4B3	Cdk2ap1	1.000	(0.000)	0.999	1.000
F6QC57	Ubr3	0.984	(0.023)	0.601	0.654
F6QCI0	Taf15	1.140	0.189	0.014	0.029
F6QHD1	Mtdh	0.940	(0.090)	0.149	0.197
F6QK97	Polr3c	1.011	0.015	0.680	0.728
F6QM56	Pgghg	0.931	(0.104)	0.053	0.084
F6RBY3	Alg2	0.906	(0.142)	0.029	0.052
F6RGI3	Gm340	0.977	(0.033)	0.695	0.741
F6RJ39	Acin1	1.146	0.197	0.000	0.003
F6RJV6	Lancl2	0.913	(0.131)	0.000	0.004
F6RK81	Ergic3	0.940	(0.089)	0.085	0.123
F6RN19	Tmem259	0.962	(0.056)	0.527	0.584
F6RPJ9	Ide	0.979	(0.031)	0.708	0.752
F6S0H0	Fam98c	0.890	(0.168)	0.013	0.028
F6S1R2	Ahsa2	0.862	(0.215)	0.024	0.045
F6S5B6	Ciz1	1.025	0.035	0.687	0.733
F6S5I0	Mprip	1.055	0.078	0.216	0.272

F6SFF5	Coq9	1.111	0.152	0.001	0.006
F6SLP4	Ergic1	0.958	(0.062)	0.042	0.070
F6SMY7	Mycbp2	0.881	(0.183)	0.000	0.003
F6SUV7	Tbc1d4	1.050	0.070	0.063	0.095
F6T9K1	Drap1	0.936	(0.096)	0.255	0.312
F6TBV1	Magt1	1.033	0.046	0.114	0.158
F6TCF9	Bag1	0.953	(0.070)	0.009	0.022
F6TLV3	Fndc3a	0.959	(0.060)	0.482	0.541
F6U6X3	Dyrk1a	0.938	(0.093)	0.018	0.036
F6UHR6	Osbpl9	1.044	0.062	0.105	0.147
F6UK66	Ccdc50	0.994	(0.009)	0.848	0.875
F6VG18	Lztfl1	0.919	(0.122)	0.000	0.004
F6VII1	Rbck1	0.992	(0.012)	0.813	0.845
F6W2Q5	Eps15	0.977	(0.034)	0.379	0.440
F6W5Q8	Vta1	0.983	(0.024)	0.142	0.190
F6WYM0	Sept2	1.069	0.096	0.012	0.026
F6X7Z3	Cpeb1	0.928	(0.107)	0.103	0.144
F6XX07	Gm21970	0.967	(0.049)	0.413	0.473
F6YIT4	Smg9	0.998	(0.003)	0.942	0.950
F6YRW4	Kdm2a	1.074	0.102	0.002	0.008
F6YWB5	Tmem256	1.096	0.132	0.011	0.025
F6ZBR8	Wiz	1.157	0.210	0.038	0.064
F6ZDE5	Rapgef1	0.993	(0.010)	0.903	0.921
F6ZDS4	Tpr	1.074	0.103	0.000	0.003
F6ZFT1	Ndufab1	0.950	(0.074)	0.153	0.201
F6ZQA3	Numa1	1.001	0.001	0.967	0.971
F7A1B4	Eng	0.970	(0.043)	0.332	0.392
F7ACR9	Macf1	0.917	(0.124)	0.014	0.030
F7AI87	Psmd4	0.931	(0.104)	0.087	0.126
F7AN16	Rnf10	0.916	(0.126)	0.131	0.177
F7APP3	Nmt2	0.932	(0.101)	0.022	0.042
F7ARZ1	Mrps23	1.131	0.178	0.040	0.067
F7B6Z5	Cpsf4	1.089	0.123	0.145	0.193
F7B7K7	Aspscr1	1.069	0.096	0.078	0.115
F7BE84	Ankrd50	1.032	0.045	0.412	0.472
F7BFR0	Mier3	1.090	0.125	0.016	0.033
F7BGR7	Gm21992	0.953	(0.070)	0.019	0.038
F7BJB9	Morc3	0.883	(0.179)	0.001	0.007
F7BT68	Ankrd52	0.868	(0.204)	0.040	0.066
F7BTP0	Nhej1	1.010	0.014	0.877	0.899
F7BX26	Ppp5c	0.923	(0.115)	0.039	0.065
F7C9N6	Mms19	0.913	(0.131)	0.028	0.051
F7CBP1	Eif4g2	0.932	(0.101)	0.050	0.079
F7CC56	Rb1cc1	0.929	(0.107)	0.005	0.014
F7CGP0	Ralgapa2	0.899	(0.153)	0.007	0.017
F7CGS5	Myt1	1.322	0.403	0.062	0.095
F7CJY7	Stx16	1.026	0.037	0.347	0.407
F7CVW0	Sbno1	0.943	(0.085)	0.122	0.166
F7CXG9	Mrpl13	1.002	0.003	0.896	0.915
F7CXI4	Engase	0.976	(0.035)	0.189	0.242
F7D0L1	Pogz	0.973	(0.039)	0.526	0.583
F7D3P8	Atp5o	0.895	(0.160)	0.030	0.053
F7D4H5	Phactr2	0.834	(0.262)	0.009	0.021
F8VPK5	Rock2	0.946	(0.080)	0.002	0.009
F8VPM0	Baz2a	0.959	(0.060)	0.040	0.066
F8VPQ2	Arid4a	0.989	(0.016)	0.615	0.667
F8VPR5	Crebbp	1.079	0.110	0.007	0.018
F8VPX1	Usp7	1.146	0.197	0.005	0.013
F8VPZ9	Bicra	1.004	0.006	0.836	0.865
F8VQ05	Fryl	1.020	0.028	0.199	0.254
F8VQ29	Iqgap3	0.881	(0.183)	0.040	0.066

F8VQ72	Map3k1	1.129	0.176	0.140	0.187
F8VQ93	Phip	1.073	0.102	0.015	0.031
F8VQC1	Srp72	1.012	0.017	0.739	0.780
F8VQE2	Kif21b	0.860	(0.218)	0.000	0.003
F8VQF9	Trappc10	0.897	(0.157)	0.004	0.012
F8VQG4	H2-T24	0.967	(0.049)	0.740	0.781
F8VQM4	Osbpl7	0.877	(0.189)	0.001	0.006
F8WGB3	Sdhc	1.095	0.131	0.131	0.177
F8WGD9	Agpat5	0.857	(0.223)	0.006	0.017
F8WGM8	Actg1	1.020	0.029	0.074	0.109
F8WGT1	Ahcyl2	0.899	(0.154)	0.082	0.119
F8WH20	Osbpl3	0.891	(0.166)	0.037	0.062
F8WH46	610010K14R	0.890	(0.169)	0.000	0.004
F8WH72	Slfn5	1.140	0.189	0.134	0.181
F8WHJ1	Fam160a2	0.961	(0.057)	0.221	0.277
F8WHK6	Phykpl	1.062	0.087	0.369	0.429
F8WHM5	Glg1	0.836	(0.258)	0.002	0.008
F8WHP8	Atp5j2	1.163	0.218	0.002	0.009
F8WHS8	Pias2	1.131	0.178	0.001	0.007
F8WHU7	Stag1	1.124	0.169	0.015	0.031
F8WHU9	Zpr1	0.933	(0.100)	0.122	0.167
F8WHW3	Pip4p1	1.089	0.123	0.063	0.095
F8WHY8	Mta1	1.129	0.175	0.000	0.003
F8WI35	H3f3a	1.124	0.169	0.000	0.004
F8WIB1	Arl1	0.891	(0.166)	0.190	0.243
F8WID5	Tpm1	1.049	0.069	0.390	0.450
F8WIE5	Hectd1	0.877	(0.189)	0.004	0.012
F8WIH0	Slmap	0.899	(0.154)	0.005	0.015
F8WIK0	Ciapin1	0.857	(0.222)	0.012	0.026
F8WIN2	Arid3b	0.965	(0.051)	0.694	0.740
F8WIT2	Anxa6	1.079	0.110	0.060	0.092
F8WIU9	Smim7	0.926	(0.111)	0.145	0.193
F8WJE3	Kansl2	1.127	0.173	0.020	0.038
F8WJG3	Tra2b	1.360	0.444	0.104	0.146
F8WJI3	Ccdc58	0.915	(0.128)	0.014	0.029
G3UVU2	Sf3a2	1.031	0.044	0.476	0.535
G3UVU9	Jmjd6	0.949	(0.076)	0.253	0.311
G3UVV3	Zfp407	1.151	0.202	0.027	0.049
G3UVV4	Hk1	0.882	(0.180)	0.003	0.010
G3UW82	Myh2	1.276	0.352	0.688	0.733
G3UWA1	Ccdc125	1.136	0.184	0.037	0.062
G3UWD2	Runx1	1.072	0.101	0.038	0.064
G3UWD4	Pphln1	0.992	(0.012)	0.721	0.764
G3UWH9	Cog4	0.892	(0.165)	0.007	0.017
G3UWI7	B3gnt2	0.855	(0.225)	0.061	0.094
G3UWJ1	Ube2i	0.968	(0.047)	0.599	0.653
G3UWR9	H2-Aa	1.264	0.339	0.056	0.087
G3UWZ0	Baz1a	1.001	0.002	0.919	0.933
G3UX26	Vdac2	1.076	0.106	0.139	0.186
G3UX44	H2-Ke6	1.024	0.034	0.779	0.815
G3UX45	Urb2	1.193	0.254	0.002	0.009
G3UX48	Prrc2a	0.928	(0.108)	0.005	0.014
G3UX80	Ccdc28a	0.973	(0.040)	0.630	0.680
G3UX81	Hmgxb4	0.978	(0.032)	0.550	0.606
G3UXL2	Prps1l3	1.054	0.076	0.240	0.297
G3UXX3	Spr	1.144	0.195	0.023	0.043
G3UXX5	Polr1c	1.154	0.207	0.003	0.011
G3UXZ5	Psme1	1.073	0.102	0.010	0.022
G3UY33	Vps52	0.912	(0.133)	0.010	0.024
G3UY39	Nelfe	0.918	(0.123)	0.042	0.068
G3UYD0	Gtf2i	1.174	0.231	0.001	0.006

G3UYF0	Banp	0.974	(0.038)	0.537	0.593
G3UYG6	Gigyf2	0.984	(0.024)	0.480	0.539
G3UYL6	Gstt3	0.868	(0.204)	0.002	0.008
G3UYM0	Stard10	0.958	(0.062)	0.255	0.312
G3UYP2	Dxo	0.892	(0.165)	0.007	0.017
G3UYQ2	Bag6	0.982	(0.026)	0.305	0.365
G3UZ26	Shmt1	0.982	(0.026)	0.290	0.350
G3UZ30	Ppp1r11	0.930	(0.104)	0.064	0.096
G3UZ34	Eftud2	0.929	(0.107)	0.271	0.329
G3UZF0	Usp3	1.054	0.076	0.058	0.089
G3UZG5	Klhdc4	0.879	(0.185)	0.002	0.008
G3UZG6	Cyb5r1	0.877	(0.189)	0.056	0.087
G3UZI2	Syncrip	1.041	0.058	0.076	0.112
G3UZJ5	Csnk2b	1.010	0.014	0.777	0.813
G3UZJ9	Rbms3	0.961	(0.057)	0.144	0.192
G3UZM9	Pkn2	0.977	(0.033)	0.311	0.371
G3UZX2	Ube2g2	0.848	(0.237)	0.012	0.026
G3X8Q0	Sp1	1.039	0.055	0.384	0.444
G3X8Q1	Cabin1	0.999	(0.001)	0.931	0.942
G3X8R0	Reep5	1.079	0.110	0.065	0.098
G3X8R4	Gtf2h1	0.926	(0.111)	0.073	0.108
G3X8R5	Qrich1	0.940	(0.089)	0.001	0.006
G3X8R7	Zfp212	1.024	0.034	0.420	0.480
G3X8S2	Brf1	1.000	0.001	0.989	0.990
G3X8U3	210016F16R	0.986	(0.020)	0.569	0.625
G3X8U8	Parg	0.992	(0.012)	0.648	0.698
G3X8W7	Cdc23	1.030	0.042	0.213	0.268
G3X8X7	Vps16	0.920	(0.121)	0.015	0.032
G3X8Y3	Naa15	1.003	0.004	0.928	0.940
G3X8Y8	Tlr2	0.897	(0.156)	0.078	0.114
G3X920	Armc8	0.956	(0.065)	0.036	0.061
G3X922	Dnajc13	0.921	(0.119)	0.005	0.014
G3X928	Sec23ip	0.853	(0.229)	0.008	0.020
G3X937	Slc41a3	0.933	(0.100)	0.128	0.174
G3X940	Kat6a	1.027	0.039	0.329	0.390
G3X949	Traf3ip3	0.980	(0.029)	0.017	0.034
G3X956	Supt16	1.193	0.255	0.000	0.003
G3X972	Sec24c	0.932	(0.102)	0.045	0.074
G3X975	Cars2	1.044	0.063	0.005	0.013
G3X9B1	Heatr1	1.026	0.037	0.130	0.177
G3X9F7	Ddx60	0.862	(0.214)	0.233	0.290
G3X9I1	Fam3c	0.880	(0.184)	0.001	0.006
G3X9T8	Cp	0.970	(0.044)	0.654	0.703
G3X9Y5	Ube4a	0.900	(0.151)	0.004	0.012
G3X9Z4	Pcf11	1.017	0.024	0.132	0.178
G3XA25	Acat2	0.950	(0.074)	0.445	0.505
G3XA30	Nsmce4a	0.958	(0.063)	0.346	0.407
G3XA79	Prc1	1.164	0.219	0.003	0.010
G5E829	Atp2b1	0.960	(0.059)	0.078	0.115
G5E837	Hcfc2	1.122	0.166	0.001	0.005
G5E852	Jak2	1.032	0.046	0.464	0.523
G5E881	Tamm41	1.154	0.207	0.000	0.004
G5E896	Edc4	1.033	0.046	0.145	0.192
G5E8E1	Lrrfip1	0.847	(0.239)	0.002	0.008
G5E8H4	Uxs1	0.951	(0.072)	0.217	0.273
G5E8J3	Wdr11	0.943	(0.084)	0.005	0.013
G5E8L1	Cdk19	0.934	(0.099)	0.278	0.337
G5E8M9	Icam2	0.949	(0.075)	0.046	0.074
G5E8N3	Setdb1	0.949	(0.075)	0.267	0.326
G5E8N7	Pnkp	0.929	(0.107)	0.073	0.108
G5E8P1	Brd1	1.132	0.179	0.016	0.033

G5E8R1	Tpm1	1.049	0.068	0.105	0.146
G5E8R8	Ubxn7	1.091	0.125	0.048	0.076
G5E8S8	Mpp7	1.061	0.085	0.068	0.102
G5E8T3	Srp19	0.848	(0.238)	0.018	0.036
G8JL74	Ptbp3	1.041	0.058	0.125	0.170
H3BJ02	Stx5a	1.096	0.133	0.001	0.007
H3BJ12	Golph3l	0.850	(0.234)	0.000	0.003
H3BJ31	Hmbox1	0.983	(0.025)	0.475	0.534
H3BJ37	Comtd1	0.875	(0.192)	0.069	0.103
H3BJ52	Zwilch	0.854	(0.227)	0.068	0.102
H3BJE0	Grn	0.912	(0.133)	0.060	0.092
H3BJE9	Rnf141	0.862	(0.215)	0.010	0.023
H3BJH7	Aftph	0.966	(0.049)	0.061	0.093
H3BJI6	Mia2	0.897	(0.157)	0.001	0.005
H3BJJ1	Gmnn	1.006	0.009	0.814	0.846
H3BJR7	Wdr37	1.159	0.213	0.015	0.031
H3BJU7	Arhgef2	0.865	(0.210)	0.015	0.031
H3BJY1	Syngr2	0.914	(0.130)	0.168	0.220
H3BKL5	Acaa1a	0.934	(0.099)	0.121	0.166
H3BKN2	Agps	0.997	(0.004)	0.891	0.912
H3BKS4	Wdr90	0.956	(0.065)	0.205	0.260
H3BKS8	Trmt1	0.927	(0.110)	0.013	0.028
H3BKT0	Cux1	0.877	(0.189)	0.005	0.015
H3BKY1	Glmp	0.899	(0.153)	0.033	0.057
H3BLB9	Pan3	1.008	0.012	0.730	0.772
H3BLH0	Smarca2	0.866	(0.208)	0.004	0.011
H3BLL3	Lrch4	0.851	(0.232)	0.005	0.014
H3BLR8	Nudt3	1.127	0.173	0.015	0.031
H7BWY2	Ap3m1	0.990	(0.014)	0.607	0.660
H7BWY5	Parp9	0.883	(0.179)	0.064	0.096
H7BX26	Cep170	1.028	0.040	0.053	0.083
H7BX88	Crat	1.005	0.008	0.800	0.834
H7BXB1	Csnk1a1	0.997	(0.004)	0.880	0.901
H7BXB9	700037H04R	0.856	(0.225)	0.033	0.058
H9KV01	Son	1.079	0.110	0.005	0.014
I1E4X0	Dlgap4	1.005	0.007	0.782	0.817
I1E4Y2	Erg	0.891	(0.166)	0.022	0.041
I3ITR1	AK157302	1.106	0.146	0.081	0.117
I7HLV2	Rpl10	0.878	(0.187)	0.041	0.068
I7HPY1	Fam3a	0.880	(0.184)	0.008	0.019
J3JS23	Zfp62	1.085	0.118	0.196	0.249
J3JS94	Lage3	1.037	0.052	0.034	0.058
J3KMT7	Tle3	0.903	(0.147)	0.001	0.006
J3QJX3	Sel1l	0.836	(0.259)	0.003	0.011
J3QM81	Ankzf1	0.849	(0.236)	0.002	0.008
J3QMA8	Snrnp48	1.073	0.102	0.040	0.066
J3QMM7	Naxd	0.974	(0.038)	0.653	0.702
J3QN35	Elp5	1.103	0.141	0.002	0.009
J3QNE8	Slc38a10	0.842	(0.248)	0.001	0.005
J3QP56	Lypla1	1.081	0.112	0.019	0.038
J7NUP1	Irgm1	1.012	0.017	0.929	0.942
K3W4L0	Myo18a	0.957	(0.064)	0.026	0.048
L7N451	Gvin1	0.845	(0.243)	0.265	0.324
O08528	Hk2	0.886	(0.175)	0.008	0.020
O08529	Capn2	0.912	(0.133)	0.024	0.045
O08547	Sec22b	1.096	0.132	0.064	0.096
O08553	Dpysl2	1.133	0.180	0.077	0.113
O08579	Emd	1.173	0.230	0.000	0.004
O08582	Gtpbp1	1.049	0.070	0.043	0.070
O08600	Endog	1.061	0.085	0.042	0.069
O08648	Map3k4	0.886	(0.175)	0.001	0.007

O08663	Metap2	0.967	(0.049)	0.264	0.322
O08664	Bcl7c	1.003	0.005	0.939	0.948
O08734	Bak1	1.170	0.226	0.017	0.034
O08738	Casp6	1.105	0.144	0.019	0.037
O08739	Ampd3	0.931	(0.103)	0.086	0.124
O08749	Dld	1.119	0.162	0.363	0.424
O08759	Ube3a	1.023	0.032	0.248	0.305
O08760	Ogg1	1.083	0.115	0.262	0.321
O08784	Tcof1	1.101	0.139	0.003	0.010
O08795	Prkcsh	0.897	(0.157)	0.015	0.031
O08796	Eef2k	1.107	0.147	0.009	0.022
O08807	Prdx4	0.980	(0.028)	0.401	0.461
O08810	Eftud2	1.147	0.198	0.001	0.005
O08856	Ell	1.005	0.008	0.752	0.791
O08912	Galnt1	0.925	(0.113)	0.034	0.058
O08915	Aip	0.893	(0.164)	0.002	0.008
O08917	Flot1	0.840	(0.251)	0.005	0.013
O08997	Atox1	0.950	(0.074)	0.459	0.518
O09000	Ncoa3	0.978	(0.032)	0.610	0.663
O09044	Snap23	0.881	(0.183)	0.001	0.006
O09061	Psmb1	0.886	(0.174)	0.000	0.003
O09110	Map2k3	0.909	(0.137)	0.098	0.139
O09111	Ndufb11	0.910	(0.137)	0.018	0.036
O09126	Sema4d	0.862	(0.214)	0.003	0.011
O09131	Gsto1	0.942	(0.086)	0.015	0.031
O09159	Man2b1	0.881	(0.182)	0.020	0.039
O09172	Gclm	0.894	(0.162)	0.167	0.218
O35071	Kif1c	0.845	(0.244)	0.003	0.010
O35075	Vps26c	0.888	(0.172)	0.007	0.017
O35114	Scarb2	1.161	0.216	0.004	0.012
O35127	Grcc10	1.029	0.042	0.407	0.467
O35129	Phb2	1.110	0.151	0.021	0.040
O35134	Polr1a	0.991	(0.013)	0.366	0.427
O35218	Cpsf2	1.176	0.233	0.001	0.005
O35226	Psmd4	1.155	0.208	0.000	0.004
O35239	Ptpn9	0.927	(0.109)	0.029	0.052
O35286	Dhx15	0.973	(0.039)	0.177	0.229
O35295	Purb	0.989	(0.015)	0.616	0.667
O35309	Nmi	0.932	(0.101)	0.319	0.380
O35350	Capn1	0.945	(0.081)	0.042	0.069
O35382	Exoc4	0.905	(0.144)	0.001	0.006
O35435	Dhodh	0.993	(0.010)	0.768	0.804
O35459	Ech1	1.025	0.036	0.552	0.608
O35465	Fkbp8	1.152	0.205	0.060	0.092
O35492	Clk3	0.951	(0.073)	0.215	0.270
O35516	Notch2	0.871	(0.199)	0.000	0.004
O35551	Rabep1	1.045	0.064	0.555	0.611
O35566	Cd151	0.859	(0.220)	0.082	0.119
O35593	Psmd14	0.986	(0.020)	0.716	0.760
O35598	Adam10	0.885	(0.176)	0.002	0.008
O35601	Fyb1	0.948	(0.076)	0.051	0.080
O35604	Npc1	1.105	0.144	0.016	0.034
O35615	Zfpm1	1.149	0.201	0.041	0.067
O35623	Bet1	1.019	0.027	0.747	0.786
O35639	Anxa3	1.091	0.126	0.031	0.054
O35685	Nudc	0.974	(0.038)	0.494	0.552
O35730	Ring1	1.044	0.062	0.134	0.181
O35737	Hnrnph1	1.147	0.198	0.001	0.007
O35744	Chil3	1.045	0.064	0.493	0.552
O35841	Api5	1.058	0.082	0.002	0.009
O35855	Bcat2	1.023	0.033	0.396	0.456

O35857	Timm44	1.169	0.226	0.001	0.005
O35864	Cops5	0.906	(0.143)	0.020	0.038
O35887	Calu	0.840	(0.251)	0.002	0.009
O35900	Lsm2	1.083	0.115	0.020	0.039
O35930	Gp1ba	0.953	(0.070)	0.456	0.516
O35954	Pitpnm1	1.158	0.212	0.055	0.086
O35955	Psmb10	0.873	(0.196)	0.021	0.040
O54692	Zw10	0.968	(0.047)	0.131	0.177
O54734	Ddost	1.107	0.147	0.010	0.022
O54774	Ap3d1	0.878	(0.187)	0.000	0.004
O54781	Srpk2	0.962	(0.056)	0.163	0.214
O54782	Man2b2	0.924	(0.114)	0.005	0.014
O54784	Dapk3	1.031	0.044	0.175	0.227
O54786	Dffa	1.056	0.078	0.173	0.225
O54790	Mafg	0.847	(0.239)	0.002	0.009
O54824	Il16	1.139	0.187	0.030	0.053
O54825	Bysl	1.038	0.054	0.174	0.226
O54833	Csnk2a2	1.043	0.061	0.022	0.042
O54890	Itgb3	0.905	(0.144)	0.349	0.410
O54918	Bcl2l11	1.080	0.111	0.485	0.544
O54946	Dnajb6	0.897	(0.156)	0.029	0.051
O54950	Prkag1	1.068	0.095	0.082	0.119
O54962	Banf1	1.128	0.174	0.000	0.004
O54984	Asna1	0.937	(0.094)	0.012	0.026
O54988	Slk	0.840	(0.251)	0.002	0.008
O55013	Trappc3	0.855	(0.225)	0.012	0.027
O55022	Pgrmc1	1.303	0.381	0.106	0.148
O55029	Copb2	0.855	(0.225)	0.013	0.028
O55057	Pde6d	1.099	0.137	0.189	0.243
O55098	Stk10	0.988	(0.018)	0.382	0.442
O55102	Bloc1s1	0.918	(0.123)	0.018	0.036
O55106	Strn	0.964	(0.053)	0.207	0.262
O55131	Septin7	1.139	0.188	0.009	0.022
O55135	Eif6	0.917	(0.124)	0.000	0.004
O55142	Rpl35a	1.026	0.037	0.436	0.496
O55143	Atp2a2	0.938	(0.092)	0.159	0.209
O55187	Cbx4	0.971	(0.042)	0.613	0.665
O55201	Supt5h	1.119	0.162	0.000	0.002
O55222	Ilk	0.992	(0.012)	0.666	0.715
O55234	Psmb5	0.923	(0.116)	0.098	0.139
O55236	Rngtt	0.944	(0.083)	0.188	0.241
O70138	Mmp8	0.900	(0.151)	0.061	0.093
O70145	Ncf2	0.920	(0.120)	0.089	0.128
O70172	Pip4k2a	1.037	0.052	0.099	0.139
O70194	Eif3d	0.977	(0.034)	0.399	0.460
O70201	Birc5	1.154	0.207	0.023	0.043
O70230	Znf143	0.976	(0.036)	0.295	0.355
O70251	Eef1b	1.083	0.115	0.186	0.239
O70252	Hmox2	1.110	0.151	0.004	0.012
O70279	Ess2	0.882	(0.180)	0.022	0.041
O70305	Atxn2	0.988	(0.018)	0.548	0.605
O70309	Itgb5	1.538	0.621	0.071	0.105
O70310	Nmt1	1.033	0.047	0.267	0.325
O70362	Gpld1	1.013	0.018	0.768	0.805
O70378	Emc8	1.137	0.186	0.003	0.010
O70400	Pdlim1	1.150	0.202	0.070	0.104
O70404	Vamp8	0.967	(0.049)	0.152	0.200
O70435	Psma3	0.894	(0.161)	0.019	0.037
O70443	Gnaz	0.809	(0.305)	0.089	0.127
O70456	Sfn	1.031	0.044	0.246	0.303
O70469	Dok2	0.879	(0.186)	0.006	0.015

O70475	Ugdh	1.007	0.010	0.842	0.869
O70494	Sp3	1.076	0.105	0.260	0.318
O70497	Fcn2	0.969	(0.046)	0.382	0.443
O70503	Hsd17b12	1.148	0.199	0.024	0.045
O70546	Kdm6a	0.954	(0.068)	0.115	0.158
O70551	Srpk1	0.840	(0.251)	0.001	0.006
O70572	Smpd2	0.911	(0.135)	0.039	0.065
O70591	Pfdn2	0.934	(0.098)	0.063	0.096
O88186	Gp9	0.836	(0.258)	0.088	0.126
O88188	Ly86	1.051	0.071	0.360	0.421
O88271	Cfdp1	1.107	0.147	0.021	0.040
O88291	Znf326	1.048	0.068	0.018	0.035
O88325	Naglu	0.978	(0.032)	0.138	0.184
O88342	Wdr1	0.848	(0.237)	0.000	0.003
O88448	Klc2	0.982	(0.027)	0.365	0.426
O88491	Nsd1	1.023	0.032	0.167	0.219
O88512	Ap1g2	1.042	0.059	0.145	0.192
O88520	Shoc2	0.983	(0.024)	0.113	0.156
O88531	Ppt1	1.033	0.046	0.488	0.546
O88532	Zfr	0.972	(0.042)	0.179	0.232
O88536	Fpr2	0.848	(0.237)	0.044	0.072
O88543	Cops3	0.985	(0.022)	0.749	0.788
O88544	Cops4	1.089	0.124	0.011	0.024
O88545	Cops6	0.917	(0.126)	0.002	0.008
O88559	Men1	1.060	0.083	0.018	0.036
O88569	Hnrnpa2b1	1.140	0.189	0.038	0.063
O88574	Sap30	1.029	0.041	0.064	0.096
O88587	Comt	1.055	0.077	0.103	0.145
O88597	Becn1	0.868	(0.204)	0.012	0.026
O88630	Gosr1	0.930	(0.104)	0.058	0.090
O88634	F2rl3	0.927	(0.110)	0.040	0.066
O88653	Lamtor3	0.975	(0.036)	0.509	0.567
O88665	Brd7	1.089	0.123	0.001	0.005
O88685	Psmc3	1.068	0.095	0.024	0.045
O88696	Clpp	1.005	0.007	0.845	0.872
O88700	Blm	1.131	0.178	0.005	0.014
O88736	Hsd17b7	1.001	0.002	0.972	0.976
O88746	Tom1	0.934	(0.099)	0.006	0.016
O88811	Stam2	0.912	(0.132)	0.022	0.042
O88844	Idh1	0.931	(0.103)	0.011	0.025
O88845	Akap10	0.885	(0.176)	0.044	0.071
O88874	Ccnk	1.021	0.030	0.239	0.295
O88878	Zfand5	0.865	(0.210)	0.014	0.029
O88879	Apaf1	0.936	(0.095)	0.007	0.018
O88952	Lin7c	0.874	(0.194)	0.003	0.010
O88958	Gnpda1	0.979	(0.030)	0.262	0.321
O88967	Yme1l1	1.059	0.083	0.021	0.040
O88968	Tcn2	0.907	(0.140)	0.021	0.040
O88983	Stx8	0.931	(0.102)	0.024	0.045
O89050	Mkln1	1.027	0.039	0.109	0.151
O89051	Itm2b	0.902	(0.148)	0.079	0.115
O89053	Coro1a	0.900	(0.152)	0.001	0.006
O89079	Cope	1.186	0.246	0.047	0.075
O89100	Grap2	1.197	0.259	0.056	0.087
O89103	Cd93	1.151	0.203	0.017	0.034
O89106	Fhit	1.188	0.249	0.030	0.053
O89110	Casp8	0.920	(0.120)	0.009	0.021
P00158	Mt-Cyb	0.963	(0.054)	0.078	0.115
P00375	Dhfr	1.145	0.195	0.008	0.019
P00405	Mtco2	1.052	0.073	0.424	0.483
P00493	Hprt1	0.962	(0.055)	0.006	0.017

P01027	C3	0.856	(0.224)	0.002	0.008
P01887	B2m	1.050	0.071	0.356	0.417
P01898	H2-Q10	0.975	(0.037)	0.217	0.273
P01899	H2-D1	1.018	0.026	0.739	0.780
P01901	H2-K1	0.958	(0.063)	0.517	0.574
P03888	Mtnd1	1.085	0.118	0.050	0.080
P03893	Mtnd2	1.079	0.110	0.212	0.267
P03911	Mtnd4	0.955	(0.067)	0.366	0.426
P03921	Mtnd5	1.064	0.090	0.179	0.231
P03930	Mtatp8	1.031	0.044	0.132	0.178
P03975	Iap	1.024	0.034	0.553	0.609
P04184	Tk1	1.106	0.145	0.099	0.139
P04202	Tgfb1	0.905	(0.143)	0.030	0.052
P04441	Cd74	1.172	0.229	0.141	0.189
P04627	Araf	0.968	(0.048)	0.235	0.291
P05063	Aldoc	0.966	(0.050)	0.789	0.824
P05064	Aldoa	0.871	(0.198)	0.003	0.010
P05201	Got1	0.858	(0.221)	0.001	0.005
P05202	Got2	0.954	(0.068)	0.055	0.086
P05214	Tuba3a	0.724	(0.466)	0.074	0.109
P05532	Kit	1.306	0.385	0.075	0.111
P05977	Myl1	0.948	(0.076)	0.887	0.908
P06339	H2-T23	0.994	(0.008)	0.937	0.947
P06537	Nr3c1	0.999	(0.002)	0.957	0.963
P06797	Ctsl	0.919	(0.122)	0.008	0.020
P06800	Ptprc	0.965	(0.051)	0.100	0.141
P07607	Tyms	1.061	0.086	0.251	0.309
P07724	Alb	0.934	(0.098)	0.209	0.264
P07742	Rrm1	1.044	0.062	0.433	0.493
P08030	Aprt	0.959	(0.060)	0.190	0.243
P08071	Ltf	0.852	(0.231)	0.004	0.013
P08113	Hsp90b1	0.965	(0.051)	0.152	0.201
P08228	Sod1	1.001	0.001	0.994	0.995
P08249	Mdh2	0.992	(0.011)	0.722	0.765
P08752	Gnai2	1.093	0.128	0.033	0.057
P08905	Lyz2	0.808	(0.307)	0.080	0.117
P09055	Itgb1	1.046	0.064	0.271	0.330
P09103	P4hb	0.868	(0.205)	0.004	0.013
P09242	Alpl	1.303	0.382	0.081	0.118
P09411	Pgk1	1.051	0.072	0.131	0.177
P09602	Hmgn2	0.837	(0.256)	0.004	0.012
P09671	Sod2	1.118	0.161	0.006	0.016
P09813	Apoa2	0.845	(0.243)	0.068	0.102
P0C7N9	Psmg4	0.897	(0.157)	0.062	0.095
P0C8B4	Gon7	1.047	0.066	0.299	0.359
P0DN34	Ndufb1	1.102	0.140	0.073	0.108
P0DP28	Calm3	0.931	(0.103)	0.027	0.048
P10107	Anxa1	0.909	(0.138)	0.051	0.080
P10404		1.173	0.230	0.015	0.032
P10649	Gstm1	0.930	(0.104)	0.173	0.225
P10711	Tcea1	1.093	0.128	0.004	0.012
P11031	Sub1	0.955	(0.066)	0.036	0.060
P11157	Rrm2	1.064	0.090	0.152	0.200
P11352	Gpx1	0.918	(0.124)	0.019	0.038
P11438	Lamp1	0.904	(0.145)	0.055	0.086
P11440	Cdk1	1.114	0.156	0.078	0.114
P11499	Hsp90ab1	1.021	0.030	0.589	0.643
P11688	Itga5	0.944	(0.083)	0.069	0.103
P11881	Itpr1	0.849	(0.236)	0.004	0.013
P11928	Oas1a	0.845	(0.242)	0.362	0.423
P11983	Tcp1	1.031	0.044	0.122	0.167

P12382	Pfkl	0.954	(0.068)	0.144	0.192
P12787	Cox5a	0.933	(0.100)	0.223	0.279
P12815	Pdcd6	0.986	(0.020)	0.177	0.229
P12970	Rpl7a	1.051	0.072	0.188	0.241
P13405	Rb1	1.098	0.135	0.002	0.009
P13439	Umps	1.052	0.074	0.102	0.143
P13541	Myh3	1.111	0.152	0.820	0.851
P13597	Icam1	0.983	(0.025)	0.744	0.784
P13705	Msh3	1.149	0.200	0.021	0.040
P13864	Dnmt1	1.184	0.244	0.001	0.005
P14106	C1qb	1.084	0.116	0.223	0.279
P14115	Rpl27a	0.904	(0.146)	0.084	0.121
P14131	Rps16	0.927	(0.109)	0.134	0.181
P14148	Rpl7	1.084	0.116	0.263	0.322
P14152	Mdh1	1.025	0.035	0.082	0.119
P14234	Fgr	0.836	(0.258)	0.005	0.015
P14483	H2-Ab1	1.015	0.021	0.825	0.855
P14576	Srp54	1.057	0.080	0.126	0.171
P14685	Psmd3	1.002	0.003	0.928	0.941
P14869	Rplp0	0.988	(0.018)	0.627	0.677
P15307	Rel	1.060	0.084	0.363	0.424
P15375	Myf6	1.115	0.157	0.041	0.068
P15532	Nme1	0.913	(0.131)	0.050	0.079
P15533	Trim30a	0.838	(0.255)	0.393	0.453
P15920	Atp6v0a2	0.991	(0.014)	0.798	0.832
P16045	Lgals1	1.155	0.208	0.010	0.023
P16125	Ldhb	0.916	(0.126)	0.052	0.081
P16254	Srp14	0.950	(0.074)	0.207	0.262
P16332	Mmut	1.175	0.232	0.003	0.011
P16460	Ass1	0.916	(0.127)	0.155	0.204
P16546	Sptan1	0.956	(0.065)	0.197	0.251
P16675	Ctsa	1.017	0.024	0.651	0.701
P16858	Gapdh	1.112	0.153	0.121	0.165
P17012	Zfx	1.070	0.098	0.052	0.082
P17156	Hspa2	1.162	0.217	0.040	0.067
P17182	Eno1	1.188	0.249	0.017	0.035
P17426	Ap2a1	1.015	0.021	0.425	0.484
P17427	Ap2a2	1.055	0.077	0.005	0.014
P17433	Spi1	1.016	0.023	0.720	0.764
P17439	Gba	0.962	(0.056)	0.366	0.426
P17665	Cox7c	0.976	(0.035)	0.014	0.029
P17742	Ppia	0.963	(0.054)	0.205	0.260
P17751	Tpi1	0.909	(0.138)	0.017	0.034
P18155	Mthfd2	1.030	0.043	0.590	0.645
P18181	Cd48	0.973	(0.039)	0.364	0.424
P18760	Cfl1	0.996	(0.006)	0.906	0.923
P19096	Fasn	0.868	(0.205)	0.012	0.027
P19253	Rpl13a	1.050	0.071	0.342	0.403
P19324	Serpinh1	1.513	0.597	0.082	0.119
P19536	Cox5b	1.047	0.066	0.018	0.035
P19783	Cox4i1	1.038	0.053	0.288	0.348
P19973	Lsp1	0.927	(0.110)	0.101	0.142
P20029	Hspa5	0.851	(0.233)	0.008	0.019
P20060	Hexb	0.922	(0.117)	0.032	0.056
P20152	Vim	0.853	(0.229)	0.001	0.005
P20491	Fcer1g	0.908	(0.139)	0.218	0.273
P20664	Prim1	1.131	0.178	0.003	0.010
P21126	Ubl4a	0.979	(0.031)	0.381	0.441
P21279	Gnaq	1.003	0.004	0.935	0.946
P21614	Gc	0.835	(0.260)	0.004	0.013
P21619	Lmnb2	1.112	0.153	0.006	0.016

P21958	Tap1	1.045	0.064	0.241	0.298
P22437	Ptgs1	0.928	(0.108)	0.012	0.027
P22682	Cbl	0.836	(0.258)	0.003	0.010
P22892	Ap1g1	0.875	(0.192)	0.004	0.013
P22907	Hmbs	1.020	0.029	0.513	0.572
P23116	Eif3a	1.017	0.024	0.611	0.663
P23506	Pcmt1	1.020	0.029	0.820	0.851
P23591	Tsta3	1.109	0.149	0.002	0.008
P23780	Glb1	1.154	0.206	0.001	0.007
P23949	Zfp36l2	1.015	0.022	0.884	0.905
P23950	Zfp36l1	1.028	0.040	0.516	0.574
P23953	Ces1c	0.985	(0.022)	0.376	0.437
P24369	Ppib	0.971	(0.043)	0.157	0.207
P24527	Lta4h	0.880	(0.185)	0.009	0.021
P24547	Impdh2	1.158	0.212	0.018	0.035
P24638	Acp2	1.135	0.183	0.005	0.015
P24668	M6pr	1.010	0.014	0.233	0.289
P24788	Cdk11b	1.146	0.197	0.000	0.004
P24860	Ccnb1	1.061	0.085	0.324	0.385
P25206	Mcm3	1.179	0.237	0.003	0.010
P25444	Rps2	1.091	0.126	0.128	0.174
P25785	Timp2	0.838	(0.256)	0.005	0.015
P25799	Nfkb1	0.915	(0.129)	0.008	0.020
P25911	Lyn	0.943	(0.085)	0.071	0.105
P26011	Itgb7	0.935	(0.096)	0.085	0.122
P26039	Tln1	0.886	(0.175)	0.001	0.007
P26040	Ezr	1.093	0.129	0.001	0.006
P26041	Msn	1.036	0.052	0.013	0.029
P26043	Rdx	0.962	(0.056)	0.128	0.174
P26231	Ctnna1	0.892	(0.164)	0.005	0.014
P26323	Fli1	0.904	(0.146)	0.013	0.028
P26350	Ptma	1.080	0.111	0.080	0.117
P26443	Glud1	1.003	0.004	0.917	0.931
P26450	Pik3r1	1.043	0.061	0.016	0.032
P26883	Fkbp1a	1.051	0.072	0.170	0.222
P27546	Map4	0.909	(0.137)	0.000	0.004
P27601	Gna13	0.931	(0.104)	0.003	0.010
P27612	Plaa	0.952	(0.072)	0.016	0.033
P27773	Pdia3	0.971	(0.043)	0.244	0.301
P27870	Vav1	0.909	(0.137)	0.001	0.006
P28063	Psmb8	0.921	(0.119)	0.041	0.067
P28271	Aco1	0.984	(0.024)	0.311	0.371
P28352	Apex1	1.120	0.164	0.003	0.010
P28474	Adh5	1.016	0.024	0.433	0.493
P28574	Max	0.989	(0.016)	0.408	0.468
P28650	Adssl1	0.960	(0.059)	0.227	0.283
P28653	Bgn	1.085	0.117	0.668	0.717
P28659	Celf1	1.113	0.155	0.011	0.024
P28665	Mug1	0.980	(0.030)	0.764	0.801
P28867	Prkcd	0.972	(0.041)	0.028	0.051
P29341	Pabpc1	1.001	0.002	0.964	0.969
P29351	Ptpn6	1.037	0.052	0.019	0.038
P29416	Hexa	1.091	0.125	0.046	0.074
P29452	Casp1	0.842	(0.248)	0.001	0.006
P29594	Casp2	0.988	(0.017)	0.722	0.765
P29595	Nedd8	0.965	(0.052)	0.278	0.337
P30282	Ccnd3	0.873	(0.196)	0.019	0.037
P30412	Ppic	0.861	(0.216)	0.051	0.081
P30416	Fkbp4	0.974	(0.038)	0.511	0.570
P30681	Hmgb2	1.029	0.042	0.290	0.349
P31230	Aimp1	1.080	0.111	0.007	0.018

P31324	Prkar2b	1.010	0.014	0.881	0.903
P31750	Akt1	0.915	(0.128)	0.003	0.010
P31938	Map2k1	0.927	(0.109)	0.007	0.017
P32020	Scp2	0.986	(0.021)	0.716	0.760
P32233	Drg1	0.959	(0.060)	0.119	0.163
P32883	Kras	0.841	(0.250)	0.000	0.003
P32921	Wars	1.009	0.013	0.550	0.607
P33174	Kif4	1.133	0.180	0.002	0.008
P33215	Nedd1	0.987	(0.020)	0.628	0.678
P33609	Pola1	1.135	0.183	0.000	0.002
P33610	Prim2	1.130	0.176	0.001	0.007
P33766	Fpr1	0.838	(0.255)	0.027	0.048
P34022	Ranbp1	1.179	0.237	0.000	0.004
P34884	Mif	1.012	0.017	0.606	0.659
P34928	Apoc1	0.991	(0.013)	0.841	0.869
P35123	Usp4	1.135	0.182	0.002	0.008
P35235	Ptpn11	0.997	(0.004)	0.676	0.724
P35276	Rab3d	0.977	(0.034)	0.612	0.664
P35278	Rab5c	1.144	0.194	0.002	0.008
P35279	Rab6a	1.051	0.071	0.089	0.128
P35282	Rab21	0.946	(0.080)	0.183	0.236
P35283	Rab12	0.945	(0.082)	0.222	0.278
P35293	Rab18	0.947	(0.078)	0.019	0.038
P35329	Cd22	1.183	0.242	0.008	0.019
P35486	Pdha1	1.147	0.198	0.012	0.027
P35564	Canx	1.155	0.208	0.003	0.010
P35585	Ap1m1	0.974	(0.038)	0.237	0.294
P35700	Prdx1	1.178	0.237	0.000	0.004
P35821	Ptpn1	0.890	(0.168)	0.027	0.049
P35979	Rpl12	0.951	(0.073)	0.294	0.353
P35991	Btk	0.966	(0.049)	0.140	0.187
P36371	Tap2	1.030	0.043	0.414	0.474
P36916	Gnl1	0.971	(0.042)	0.398	0.458
P36993	Ppm1b	0.943	(0.085)	0.250	0.307
P37040	Por	1.141	0.191	0.001	0.005
P38060	Hmgcl	1.150	0.201	0.007	0.017
P39429	Traf2	0.894	(0.161)	0.004	0.012
P40201	Chd1	0.925	(0.112)	0.032	0.055
P40240	Cd9	0.925	(0.112)	0.265	0.324
P40336	Vps26a	0.992	(0.012)	0.586	0.641
P40630	Tfam	1.162	0.217	0.001	0.007
P41105	Rpl28	0.927	(0.110)	0.319	0.380
P42125	Eci1	1.115	0.157	0.001	0.005
P42128	Foxk1	1.090	0.125	0.299	0.359
P42230	Stat5a	1.146	0.197	0.019	0.037
P42232	Stat5b	0.991	(0.013)	0.708	0.753
P42669	Pura	1.093	0.129	0.001	0.007
P42859	Htt	0.993	(0.010)	0.305	0.366
P42867	Dpagt1	0.850	(0.235)	0.018	0.036
P42932	Cct8	1.113	0.155	0.007	0.018
P43024	Cox6a1	0.921	(0.119)	0.400	0.460
P43406	Itgav	1.039	0.056	0.341	0.402
P43430	Mcpt8	1.078	0.108	0.153	0.201
P45376	Akr1b1	1.167	0.223	0.002	0.009
P46061	Rangap1	1.197	0.260	0.001	0.007
P46414	Cdkn1b	0.992	(0.012)	0.600	0.653
P46460	Nsf	1.025	0.035	0.229	0.285
P46467	Vps4b	0.898	(0.155)	0.038	0.063
P46471	Psmc2	1.116	0.158	0.003	0.010
P46638	Rab11b	1.033	0.047	0.061	0.093
P46664	Adss	1.056	0.079	0.062	0.094

P46718	Pdcd2	1.050	0.071	0.056	0.087
P46737	Brcc3	1.015	0.022	0.589	0.643
P46935	Nedd4	1.053	0.075	0.117	0.161
P46978	Stt3a	0.930	(0.104)	0.030	0.053
P47738	Aldh2	1.042	0.059	0.134	0.180
P47740	Aldh3a2	0.891	(0.166)	0.001	0.006
P47754	Capza2	1.100	0.138	0.013	0.028
P47809	Map2k4	0.886	(0.175)	0.027	0.049
P47811	Mapk14	0.873	(0.196)	0.001	0.006
P47857	Pfkm	1.144	0.194	0.015	0.031
P47879	Igfbp4	0.744	(0.427)	0.066	0.099
P47911	Rpl6	0.955	(0.067)	0.467	0.526
P47941	Crkl	1.128	0.173	0.001	0.007
P47962	Rpl5	1.011	0.016	0.805	0.838
P47963	Rpl13	1.094	0.130	0.121	0.166
P47968	Rpia	0.913	(0.132)	0.021	0.040
P48024	Eif1	1.107	0.147	0.039	0.065
P48036	Anxa5	0.939	(0.090)	0.041	0.068
P48722	Hspa4l	0.920	(0.120)	0.003	0.010
P48758	Cbr1	1.179	0.238	0.002	0.008
P48771	Cox7a2	1.196	0.258	0.004	0.012
P48774	Gstm5	1.143	0.192	0.017	0.035
P48962	Slc25a4	1.180	0.239	0.019	0.037
P49135	Ercc3	1.031	0.044	0.023	0.043
P49138	Mapkapk2	0.877	(0.189)	0.008	0.019
P49290	Epx	0.780	(0.359)	0.209	0.264
P49442	Inpp1	0.987	(0.020)	0.729	0.771
P49446	Ptpre	0.866	(0.208)	0.002	0.009
P49452	Cenpc	1.191	0.252	0.007	0.017
P49586	Pcyt1a	0.936	(0.095)	0.218	0.274
P49615	Cdk5	1.040	0.057	0.585	0.640
P49710	Hcls1	1.028	0.039	0.227	0.283
P49717	Mcm4	1.182	0.241	0.002	0.009
P49718	Mcm5	1.160	0.214	0.000	0.005
P49722	Psma2	0.899	(0.153)	0.010	0.022
P49769	Psen1	0.974	(0.038)	0.413	0.473
P49935	Ctsh	1.070	0.098	0.033	0.058
P49962	Srp9	0.859	(0.219)	0.001	0.005
P50247	Ahcy	1.075	0.105	0.019	0.038
P50295	Nat2	1.147	0.198	0.004	0.013
P50396	Gdi1	1.008	0.012	0.845	0.873
P50427	Sts	1.060	0.084	0.069	0.103
P50516	Atp6v1a	0.912	(0.132)	0.062	0.095
P50518	Atp6v1e1	0.929	(0.106)	0.110	0.152
P50543	S100a11	0.876	(0.191)	0.034	0.058
P51125	Cast	0.897	(0.158)	0.000	0.003
P51141	Dvl1	0.921	(0.119)	0.131	0.177
P51150	Rab7a	0.973	(0.039)	0.419	0.479
P51437	Camp	1.098	0.134	0.414	0.474
P51660	Hsd17b4	1.086	0.119	0.037	0.063
P51855	Gss	0.912	(0.133)	0.028	0.050
P51863	Atp6v0d1	0.942	(0.086)	0.030	0.053
P51943	Ccna2	0.959	(0.060)	0.141	0.189
P52196	Tst	0.942	(0.086)	0.624	0.675
P52293	Kpna2	1.116	0.158	0.113	0.156
P52479	Usp10	1.170	0.226	0.018	0.036
P52480	Pkm	0.998	(0.003)	0.968	0.972
P52483	Ube2e3	1.150	0.202	0.007	0.018
P52633	Stat6	0.968	(0.047)	0.138	0.184
P52760	Rida	0.945	(0.081)	0.185	0.238
P52825	Cpt2	1.090	0.124	0.026	0.047

P52850	Ndst2	1.099	0.136	0.144	0.191
P53026	Rpl10a	0.862	(0.214)	0.022	0.041
P53395	Dbt	1.112	0.153	0.027	0.049
P53702	Hccs	0.988	(0.017)	0.630	0.680
P53810	Pitpna	0.985	(0.022)	0.113	0.156
P53811	Pitpnb	1.004	0.005	0.902	0.920
P53994	Rab2a	0.960	(0.058)	0.018	0.035
P53995	Anapc1	1.057	0.081	0.017	0.035
P53996	Cnbp	1.020	0.029	0.285	0.344
P54071	Idh2	1.094	0.130	0.004	0.013
P54103	Dnajc2	0.997	(0.004)	0.863	0.887
P54116	Stom	1.040	0.057	0.016	0.032
P54227	Stmn1	1.042	0.059	0.301	0.361
P54728	Rad23b	0.952	(0.071)	0.175	0.227
P54729	Nub1	0.953	(0.069)	0.115	0.158
P54731	Faf1	1.072	0.100	0.008	0.020
P54775	Psmc4	1.163	0.217	0.005	0.014
P54818	Galc	0.865	(0.210)	0.006	0.016
P54823	Ddx6	1.010	0.014	0.776	0.812
P54923	Adprh	0.919	(0.121)	0.001	0.007
P55041	Gem	0.870	(0.200)	0.039	0.065
P55097	Ctsk	1.054	0.075	0.639	0.689
P55194	Sh3bp1	0.883	(0.179)	0.000	0.004
P55258	Rab8a	1.148	0.199	0.010	0.022
P55264	Adk	1.024	0.034	0.339	0.400
P55302	Lrpap1	1.029	0.041	0.489	0.547
P55772	Entpd1	0.968	(0.047)	0.168	0.219
P55821	Stmn2	0.963	(0.054)	0.347	0.407
P56183	Rrp1	1.116	0.159	0.000	0.003
P56213	Gfer	0.873	(0.197)	0.011	0.024
P56376	Acyp1	1.124	0.168	0.013	0.029
P56382	Atp5f1e	1.051	0.071	0.188	0.241
P56391	Cox6b1	0.903	(0.146)	0.003	0.011
P56394	Cox17	0.962	(0.056)	0.219	0.275
P56400	Gp1bb	0.982	(0.026)	0.666	0.715
P56477	Irf5	1.052	0.073	0.559	0.615
P56528	Cd38	1.089	0.123	0.069	0.103
P56542	Dnase2	1.059	0.083	0.150	0.199
P56812	Pdcd5	0.987	(0.019)	0.710	0.754
P56931	E2f2	0.989	(0.016)	0.619	0.670
P56960	Exosc10	1.049	0.070	0.000	0.003
P57080	Usp25	0.910	(0.136)	0.060	0.092
P57716	Ncstn	0.865	(0.209)	0.003	0.011
P57725	Samsn1	0.843	(0.246)	0.000	0.003
P57746	Atp6v1d	1.044	0.062	0.054	0.084
P57780	Actn4	0.900	(0.152)	0.001	0.007
P57784	Snrpa1	1.106	0.146	0.000	0.004
P58044	Idi1	0.903	(0.147)	0.027	0.048
P58058	Nadk	0.878	(0.187)	0.042	0.069
P58064	Mrps6	1.066	0.092	0.226	0.282
P58137	Acot8	0.995	(0.008)	0.719	0.763
P58252	Eef2	0.975	(0.036)	0.309	0.369
P58281	Opa1	1.091	0.126	0.064	0.096
P58389	Ptpa	1.047	0.066	0.043	0.071
P58404	Strn4	1.050	0.070	0.074	0.110
P58466	Ctdsp1	0.908	(0.139)	0.034	0.059
P58501	Paxbp1	1.044	0.063	0.112	0.155
P58681	Tlr7	1.090	0.125	0.310	0.370
P58742	Aaas	0.945	(0.082)	0.033	0.057
P58802	Tbc1d10a	1.048	0.067	0.060	0.092
P58854	Tubgcp3	1.085	0.118	0.002	0.009

P59016	Vps33b	1.024	0.034	0.608	0.661
P59048	Pdrg1	1.050	0.070	0.178	0.230
P59108	Cpne2	0.848	(0.238)	0.002	0.009
P59114	Pcif1	0.944	(0.083)	0.009	0.021
P59235	Nup43	0.996	(0.006)	0.951	0.959
P59325	Eif5	1.057	0.080	0.056	0.087
P59326	Ythdf1	0.937	(0.094)	0.099	0.139
P59481	Lman2l	0.902	(0.150)	0.003	0.010
P59729	Rin3	0.860	(0.218)	0.004	0.012
P59999	Arpc4	1.040	0.057	0.104	0.145
P60060	Sec61g	0.856	(0.224)	0.074	0.109
P60229	Eif3e	1.099	0.136	0.038	0.064
P60335	Pcbp1	0.975	(0.037)	0.508	0.567
P60487	Pdxp	0.984	(0.024)	0.618	0.669
P60521	Gabarapl2	0.925	(0.113)	0.137	0.183
P60603	Romo1	0.846	(0.242)	0.086	0.124
P60670	Nploc4	0.927	(0.109)	0.007	0.017
P60710	Actb	1.067	0.093	0.050	0.079
P60762	Morf4l1	1.191	0.252	0.007	0.018
P60766	Cdc42	0.970	(0.044)	0.130	0.177
P60840	Ensa	0.992	(0.012)	0.659	0.708
P60843	Eif4a1	1.120	0.164	0.071	0.105
P60853	Lzts1	0.748	(0.418)	0.095	0.135
P60867	Rps20	0.940	(0.089)	0.094	0.133
P60898	Polr2i	0.956	(0.066)	0.096	0.136
P61021	Rab5b	1.109	0.149	0.046	0.074
P61022	Chp1	0.909	(0.138)	0.005	0.014
P61027	Rab10	0.975	(0.037)	0.218	0.273
P61028	Rab8b	1.103	0.142	0.007	0.017
P61080	Ube2d1	0.946	(0.081)	0.102	0.143
P61082	Ube2m	1.112	0.154	0.019	0.037
P61087	Ube2k	1.087	0.121	0.006	0.016
P61161	Actr2	0.967	(0.049)	0.113	0.156
P61164	Actr1a	0.893	(0.163)	0.001	0.006
P61205	Arf3	1.189	0.250	0.186	0.239
P61222	Abce1	1.127	0.173	0.003	0.010
P61226	Rap2b	1.004	0.006	0.904	0.921
P61255	Rpl26	1.115	0.157	0.075	0.110
P61290	Psme3	1.066	0.092	0.043	0.070
P61294	Rab6b	1.021	0.030	0.594	0.648
P61514	Rpl37a	0.861	(0.216)	0.011	0.025
P61600	Naa20	0.843	(0.247)	0.031	0.055
P61620	Sec61a1	0.916	(0.126)	0.225	0.281
P61750	Arf4	1.095	0.131	0.001	0.007
P61759	Vbp1	0.946	(0.080)	0.035	0.060
P61804	Dad1	0.852	(0.231)	0.003	0.010
P61924	Copz1	0.995	(0.007)	0.815	0.847
P61963	Dcaf7	1.021	0.030	0.686	0.732
P61965	Wdr5	0.920	(0.120)	0.008	0.020
P61967	Ap1s1	0.891	(0.167)	0.025	0.046
P61971	Nutf2	1.023	0.033	0.180	0.232
P61982	Ywhag	1.126	0.172	0.007	0.018
P62073	Timm10	0.910	(0.136)	0.004	0.013
P62075	Timm13	0.984	(0.024)	0.386	0.446
P62077	Timm8b	0.914	(0.130)	0.041	0.068
P62082	Rps7	1.138	0.187	0.034	0.058
P62137	Ppp1ca	0.977	(0.034)	0.060	0.092
P62141	Ppp1cb	0.895	(0.159)	0.069	0.103
P62192	Psmc1	1.101	0.138	0.004	0.012
P62196	Psmc5	1.076	0.106	0.018	0.035
P62242	Rps8	1.078	0.108	0.050	0.079

P62259	Ywhae	1.034	0.048	0.128	0.174
P62264	Rps14	1.175	0.233	0.044	0.072
P62267	Rps23	1.005	0.007	0.865	0.889
P62281	Rps11	1.172	0.229	0.009	0.022
P62301	Rps13	0.920	(0.121)	0.208	0.263
P62305	Snrpe	1.087	0.121	0.017	0.034
P62307	Snrpf	1.079	0.109	0.394	0.454
P62309	Snrpg	1.026	0.037	0.461	0.520
P62311	Lsm3	0.908	(0.140)	0.022	0.041
P62313	Lsm6	1.081	0.112	0.024	0.045
P62315	Snrpd1	1.119	0.163	0.034	0.059
P62317	Snrpd2	1.025	0.036	0.118	0.162
P62320	Snrpd3	1.032	0.045	0.744	0.784
P62322	Lsm5	1.075	0.105	0.045	0.073
P62331	Arf6	1.118	0.161	0.001	0.005
P62334	Psmc6	1.060	0.084	0.071	0.105
P62342	Selenot	0.994	(0.008)	0.766	0.803
P62488	Polr2g	1.090	0.124	0.001	0.006
P62627	Dynlrb1	0.850	(0.234)	0.013	0.028
P62700	Ypel5	1.082	0.114	0.032	0.056
P62702	Rps4x	1.167	0.223	0.021	0.041
P62715	Ppp2cb	0.960	(0.059)	0.008	0.020
P62717	Rpl18a	1.120	0.163	0.077	0.113
P62743	Ap2s1	0.963	(0.055)	0.052	0.081
P62746	Rhob	0.958	(0.062)	0.066	0.099
P62751	Rpl23a	0.946	(0.080)	0.404	0.465
P62754	Rps6	0.953	(0.069)	0.329	0.390
P62774	Mtpn	1.024	0.034	0.262	0.320
P62814	Atp6v1b2	1.047	0.066	0.102	0.143
P62830	Rpl23	0.971	(0.043)	0.626	0.676
P62835	Rap1a	1.194	0.256	0.006	0.017
P62852	Rps25	1.184	0.243	0.034	0.059
P62855	Rps26	0.867	(0.205)	0.017	0.034
P62869	Elob	1.003	0.004	0.884	0.905
P62874	Gnb1	0.933	(0.100)	0.205	0.260
P62876	Polr2l	1.035	0.050	0.388	0.449
P62878	Rbx1	1.127	0.173	0.451	0.511
P62880	Gnb2	0.853	(0.229)	0.030	0.053
P62889	Rpl30	0.935	(0.097)	0.140	0.187
P62908	Rps3	0.979	(0.031)	0.587	0.641
P62911	Rpl32	0.980	(0.029)	0.673	0.722
P62915	Gtf2b	1.108	0.147	0.015	0.031
P62960	Ybx1	0.874	(0.194)	0.186	0.239
P62983	Rps27a	0.987	(0.018)	0.602	0.655
P63001	Rac1	1.009	0.013	0.811	0.844
P63005	Pafah1b1	0.880	(0.185)	0.002	0.008
P63011	Rab3a	1.068	0.095	0.497	0.555
P63017	Hspa8	1.088	0.122	0.035	0.060
P63024	Vamp3	1.112	0.153	0.000	0.003
P63037	Dnaja1	1.195	0.257	0.001	0.006
P63044	Vamp2	1.162	0.217	0.178	0.230
P63085	Mapk1	1.061	0.086	0.021	0.040
P63087	Ppp1cc	1.003	0.005	0.849	0.875
P63094	Gnas	1.002	0.002	0.957	0.963
P63101	Ywhaz	1.115	0.157	0.025	0.046
P63154	Crnkl1	1.042	0.059	0.001	0.007
P63163	Snrpn	1.090	0.125	0.001	0.005
P63166	Sumo1	1.112	0.153	0.064	0.096
P63213	Gng2	0.866	(0.208)	0.305	0.366
P63254	Crip1	1.213	0.279	0.113	0.156
P63260	Actg1	0.736	(0.443)	0.082	0.119

P63276	Rps17	1.008	0.012	0.896	0.915
P63321	Rala	0.940	(0.089)	0.104	0.146
P63328	Ppp3ca	0.943	(0.085)	0.033	0.057
P63330	Ppp2ca	0.977	(0.034)	0.481	0.540
P67984	Rpl22	0.985	(0.022)	0.754	0.792
P68040	Rack1	0.985	(0.021)	0.748	0.786
P68134	Acta1	1.181	0.240	0.638	0.688
P68181	Prkacb	0.906	(0.142)	0.008	0.020
P68404	Prkcb	1.186	0.246	0.005	0.014
P68510	Ywhah	1.187	0.247	0.000	0.004
P70122	Sbds	0.995	(0.007)	0.854	0.880
P70168	Kpnb1	1.023	0.033	0.208	0.263
P70195	Psmb7	0.885	(0.176)	0.015	0.031
P70196	Traf6	1.131	0.177	0.120	0.165
P70218	Map4k1	0.964	(0.053)	0.057	0.089
P70227	Itpr3	1.133	0.180	0.119	0.163
P70236	Map2k6	1.051	0.071	0.271	0.329
P70255	Nfic	0.974	(0.038)	0.482	0.541
P70279	Surf6	1.186	0.246	0.000	0.004
P70280	Vamp7	1.044	0.062	0.112	0.154
P70295	Aup1	0.924	(0.114)	0.098	0.139
P70296	Pebp1	0.937	(0.094)	0.101	0.143
P70302	Stim1	0.943	(0.085)	0.014	0.030
P70303	Ctps2	1.018	0.025	0.448	0.508
P70315	Was	0.981	(0.028)	0.455	0.515
P70318	Tial1	1.031	0.044	0.196	0.250
P70333	Hnrnph2	1.019	0.028	0.351	0.411
P70335	Rock1	0.931	(0.103)	0.001	0.007
P70349	Hint1	1.019	0.028	0.709	0.753
P70353	Nfyc	0.941	(0.088)	0.093	0.132
P70371	Terf1	1.196	0.258	0.000	0.003
P70372	Elavl1	1.141	0.190	0.001	0.006
P70399	Tp53bp1	1.131	0.177	0.004	0.013
P70404	Idh3g	1.037	0.053	0.144	0.192
P70441	Slc9a3r1	0.875	(0.193)	0.001	0.006
P70444	Bid	0.849	(0.236)	0.003	0.010
P70445	Eif4ebp2	0.971	(0.043)	0.280	0.339
P70452	Stx4	0.894	(0.161)	0.003	0.011
P70460	Vasp	0.889	(0.170)	0.002	0.007
P70671	Irf3	0.920	(0.121)	0.030	0.053
P70677	Casp3	1.066	0.092	0.049	0.078
P70697	Urod	1.145	0.196	0.002	0.008
P70698	Ctps1	1.088	0.121	0.008	0.020
P70699	Gaa	0.873	(0.197)	0.025	0.046
P80313	Cct7	1.179	0.237	0.004	0.012
P80315	Cct4	1.054	0.075	0.137	0.184
P80316	Cct5	1.077	0.107	0.085	0.122
P80317	Cct6a	1.186	0.247	0.003	0.011
P80318	Cct3	1.144	0.195	0.009	0.021
P81269	Atf1	0.965	(0.051)	0.073	0.108
P83741	Wnk1	0.933	(0.101)	0.004	0.011
P83870	Phf5a	1.136	0.184	0.006	0.015
P83877	Txnl4a	0.969	(0.045)	0.657	0.706
P84078	Arf1	1.058	0.082	0.064	0.096
P84084	Arf5	0.862	(0.214)	0.025	0.046
P84091	Ap2m1	1.005	0.007	0.630	0.680
P84102	Serf2	1.010	0.014	0.832	0.861
P84104	Srsf3	1.135	0.183	0.007	0.018
P97300	Nptn	0.914	(0.130)	0.079	0.115
P97304	Polr1d	0.917	(0.125)	0.059	0.091
P97310	Mcm2	1.120	0.164	0.070	0.105

P97311	Mcm6	1.145	0.196	0.002	0.007
P97313	Prkdc	1.060	0.084	0.058	0.089
P97315	Csrp1	1.160	0.214	0.106	0.148
P97329	Kif20a	1.129	0.175	0.005	0.015
P97351	Rps3a	0.920	(0.120)	0.053	0.084
P97352	S100a13	0.965	(0.052)	0.325	0.385
P97355	Sms	0.997	(0.005)	0.870	0.893
P97369	Ncf4	0.978	(0.032)	0.328	0.388
P97376	Frg1	1.044	0.062	0.016	0.033
P97377	Cdk2	1.084	0.116	0.011	0.024
P97379	G3bp2	1.087	0.120	0.020	0.038
P97390	Vps45	1.134	0.181	0.004	0.012
P97401	Frzb	0.917	(0.125)	0.222	0.278
P97426	Ear1	0.844	(0.244)	0.070	0.104
P97450	Atp5pf	1.101	0.139	0.047	0.076
P97452	Bop1	1.044	0.063	0.114	0.157
P97465	Dok1	0.965	(0.052)	0.017	0.034
P97470	Ppp4c	0.886	(0.174)	0.017	0.035
P97471	Smad4	0.903	(0.148)	0.015	0.032
P97477	Aurka	0.891	(0.167)	0.003	0.010
P97478	Coq7	1.109	0.149	0.005	0.015
P97492	Rgs14	0.894	(0.162)	0.004	0.012
P97496	Smarcc1	1.162	0.217	0.002	0.009
P97504	Bmx	0.839	(0.254)	0.002	0.008
P97742	Cpt1a	1.025	0.036	0.446	0.506
P97760	Polr2c	1.178	0.236	0.000	0.004
P97762	rp9	0.984	(0.024)	0.443	0.503
P97770	Thumpd3	1.135	0.183	0.000	0.004
P97822	Anp32e	1.115	0.158	0.000	0.004
P97825	Jpt1	1.021	0.030	0.561	0.617
P97865	Pex7	0.882	(0.181)	0.003	0.011
P97930	Dtymk	1.062	0.086	0.177	0.229
P98083	Shc1	1.199	0.262	0.040	0.066
P99026	Psmb4	0.879	(0.185)	0.003	0.010
P99027	Rplp2	0.940	(0.090)	0.211	0.266
P99028	Uqcrh	0.940	(0.089)	0.024	0.045
P99029	Prdx5	0.863	(0.213)	0.015	0.031
Q00422	Gabpa	1.080	0.111	0.008	0.020
Q00547	Hmmr	1.047	0.067	0.221	0.276
Q00558	F8a1	1.056	0.079	0.008	0.020
Q00612	G6pdx	0.845	(0.243)	0.018	0.036
Q00898	Serpina1e	1.078	0.109	0.346	0.407
Q00899	Yy1	1.191	0.252	0.014	0.030
Q00915	Rbp1	1.221	0.288	0.061	0.093
Q00PI9	Hnrnpul2	1.010	0.015	0.445	0.505
Q01853	Vcp	0.944	(0.084)	0.287	0.346
Q01965	Ly9	1.143	0.193	0.023	0.043
Q02053	Uba1	1.106	0.146	0.002	0.008
Q02395	Mtf2	0.988	(0.017)	0.753	0.791
Q02614	Sap30bp	1.078	0.108	0.000	0.005
Q03958	Pfdn6	0.949	(0.075)	0.031	0.054
Q03963	Eif2ak2	0.818	(0.289)	0.205	0.260
Q04692	Smarcad1	1.071	0.099	0.036	0.061
Q04736	Yes1	1.047	0.066	0.064	0.097
Q04750	Top1	1.080	0.110	0.010	0.024
Q05144	Rac2	1.039	0.055	0.387	0.447
Q05186	Rcn1	1.079	0.110	0.213	0.268
Q05816	Fabp5	0.986	(0.021)	0.589	0.643
Q05BH3	Dhx16	0.966	(0.050)	0.212	0.267
Q05CL8	Larp7	1.045	0.064	0.082	0.119
Q05D44	Eif5b	1.016	0.023	0.551	0.608

Q05DV2	Gnpat	0.938	(0.093)	0.009	0.021
Q06138	Cab39	0.955	(0.066)	0.142	0.190
Q06185	Atp5me	0.938	(0.093)	0.167	0.218
Q07113	Igf2r	0.871	(0.199)	0.002	0.008
Q07235	Serpine2	0.903	(0.146)	0.169	0.221
Q07417	Acads	1.091	0.126	0.004	0.013
Q07797	Lgals3bp	1.143	0.192	0.325	0.386
Q07832	Plk1	0.947	(0.078)	0.394	0.454
Q08024	Cbfb	0.927	(0.110)	0.053	0.082
Q08093	Cnn2	0.837	(0.257)	0.001	0.006
Q08288	Lyar	1.061	0.085	0.245	0.302
Q08376	Zbtb14	1.096	0.132	0.054	0.084
Q08642	Padi2	1.154	0.207	0.072	0.106
Q08761	Pros1	0.868	(0.205)	0.007	0.017
Q09XV5	Chd8	0.909	(0.137)	0.046	0.075
Q0GNC1	Inf2	0.936	(0.096)	0.024	0.045
Q0P678	Zc3h18	1.139	0.187	0.001	0.006
Q11011	Npepps	0.888	(0.171)	0.018	0.035
Q11136	Pepd	0.939	(0.091)	0.039	0.065
Q149F1	Rpusd2	1.047	0.066	0.109	0.151
Q149L6	Dnajb14	1.086	0.119	0.077	0.113
Q14AI0	DSCC1	0.819	(0.288)	0.200	0.255
Q14AX6	Cdk12	1.016	0.022	0.236	0.292
Q14B01	Rnf113a2	0.923	(0.115)	0.001	0.007
Q14B65	Cep95	1.003	0.005	0.901	0.919
Q14C51	Ptcd3	0.987	(0.018)	0.702	0.747
Q2EMV9	Parp14	1.013	0.018	0.895	0.914
Q2NL51	Gsk3a	1.147	0.198	0.002	0.009
Q2TBA3	Malt1	0.956	(0.064)	0.222	0.278
Q2TBE6	Pi4k2a	0.943	(0.085)	0.034	0.059
Q2TPA8	Hsdl2	1.009	0.013	0.678	0.726
Q31093	H2-M3	1.070	0.098	0.368	0.428
Q31125	Slc39a7	0.838	(0.254)	0.004	0.012
Q32NY4	Cnnm3	0.923	(0.116)	0.056	0.087
Q3B7Z2	Osbp	0.935	(0.098)	0.001	0.007
Q3LFS7	Ceacam1	0.849	(0.236)	0.096	0.135
Q3T9A5	Glmn	1.108	0.148	0.000	0.003
Q3T9E4	Tgtp2	1.131	0.177	0.505	0.563
Q3T9X3	Dnm2	0.991	(0.013)	0.253	0.310
Q3TA68	Wdr36	1.016	0.022	0.474	0.533
Q3TAI4	Tpd52l2	0.999	(0.001)	0.970	0.974
Q3TAS6	Emc10	0.983	(0.024)	0.821	0.852
Q3TBD2	Arhgap45	0.916	(0.127)	0.000	0.004
Q3TBU7	Agfg2	0.896	(0.158)	0.023	0.044
Q3TCH7	Cul4a	1.080	0.112	0.034	0.059
Q3TCJ1	Abraxas2	0.893	(0.163)	0.001	0.006
Q3TCN2	Plbd2	0.913	(0.131)	0.003	0.010
Q3TDD9	Ppp1r21	0.873	(0.196)	0.000	0.004
Q3TDN2	Faf2	1.186	0.246	0.000	0.004
Q3TFD2	Lpcat1	1.059	0.082	0.061	0.093
Q3TG45	Psmd8	1.040	0.057	0.096	0.136
Q3TGF2	Fam107b	0.980	(0.029)	0.734	0.776
Q3TGG2	Atxn2l	1.004	0.006	0.851	0.877
Q3TGM7	Hbs1l	0.946	(0.080)	0.010	0.023
Q3TGU7	Pa2g4	1.069	0.097	0.009	0.022
Q3THE2	Myl12b	0.879	(0.187)	0.289	0.349
Q3THG9	Aarsd1	1.069	0.096	0.031	0.054
Q3THK3	Gtf2f1	1.023	0.032	0.051	0.080
Q3THK7	Gmps	1.105	0.144	0.008	0.019
Q3THS6	Mat2a	0.982	(0.027)	0.123	0.168
Q3THW5	H2afv	0.978	(0.032)	0.252	0.309

Q3TIR3	Ric8a	0.914	(0.130)	0.073	0.108
Q3TIU4	Pde12	1.178	0.236	0.035	0.059
Q3TIV5	Zc3h15	0.978	(0.031)	0.265	0.324
Q3TIV9	Ap1s2	0.959	(0.060)	0.102	0.143
Q3TJ55	Stx2	0.907	(0.141)	0.000	0.004
Q3TJY1	Mcrs1	0.928	(0.107)	0.089	0.127
Q3TKY6	Cwc27	1.019	0.027	0.300	0.360
Q3TMH2	Scrn3	0.888	(0.171)	0.026	0.047
Q3TML0	Pdia6	0.879	(0.187)	0.046	0.075
Q3TMP1	Gtf3c3	1.098	0.135	0.001	0.007
Q3TN85	Rad23a	0.937	(0.094)	0.030	0.053
Q3TNB8	Fgd3	0.891	(0.167)	0.002	0.008
Q3TNH5	Fam172a	1.054	0.076	0.118	0.162
Q3TPE9	Ankmy2	0.723	(0.468)	0.172	0.224
Q3TPX4	Exoc5	0.915	(0.128)	0.001	0.005
Q3TQ29	Pum2	0.956	(0.064)	0.005	0.015
Q3TQI7		1.045	0.063	0.247	0.304
Q3TQP0	Gm10767	1.138	0.186	0.036	0.061
Q3TR20	Cog7	0.916	(0.126)	0.086	0.123
Q3TRM8	Hk3	0.876	(0.191)	0.003	0.010
Q3TSG4	Alkbh5	1.145	0.195	0.005	0.014
Q3TSR0	Extl2	0.991	(0.014)	0.819	0.850
Q3TTF2	Ubxn8	0.935	(0.097)	0.221	0.277
Q3TU36	Rap1gds1	0.968	(0.047)	0.063	0.095
Q3TUE1	Fubp1	1.089	0.123	0.004	0.012
Q3TUQ5	Pnn	1.049	0.069	0.008	0.020
Q3TUQ7	Prkaa1	0.978	(0.032)	0.486	0.545
Q3TUU5	Tex30	1.045	0.063	0.041	0.067
Q3TV70	Nr2c2ap	1.082	0.114	0.049	0.078
Q3TVC7	Ccndbp1	0.860	(0.217)	0.286	0.345
Q3TVF4	Rabggtb	0.935	(0.097)	0.121	0.165
Q3TW96	Uap1l1	0.934	(0.099)	0.037	0.063
Q3TXS7	Psmd1	0.937	(0.094)	0.016	0.033
Q3TXX3	Zfyve27	0.887	(0.173)	0.024	0.044
Q3TYA6	Mphosph8	0.997	(0.004)	0.907	0.923
Q3TYS2	Cybc1	0.964	(0.053)	0.037	0.062
Q3TYX3	Smyd5	0.967	(0.048)	0.108	0.149
Q3TZZ7	Esyt2	0.968	(0.047)	0.089	0.128
Q3U0J8	Tbc1d2b	0.849	(0.236)	0.003	0.011
Q3U0V2	Tradd	0.960	(0.058)	0.027	0.049
Q3U186	Rars2	0.911	(0.134)	0.122	0.166
Q3U1J4	Ddb1	1.082	0.113	0.046	0.074
Q3U1T9	Dennd1b	0.940	(0.089)	0.282	0.341
Q3U1U4	Gm49368	0.902	(0.149)	0.047	0.075
Q3U1V6	Uevld	1.127	0.172	0.011	0.026
Q3U1Y4	Dennd4b	1.105	0.145	0.458	0.517
Q3U1Z5	Gpsm3	0.877	(0.189)	0.020	0.038
Q3U2A8	Vars2	1.126	0.171	0.006	0.017
Q3U2G2	Hspa4	1.122	0.166	0.001	0.006
Q3U2P1	Sec24a	0.871	(0.199)	0.006	0.017
Q3U2S8	Hvcn1	0.848	(0.238)	0.003	0.010
Q3U308	Ctu2	0.991	(0.013)	0.546	0.603
Q3U319	Rnf40	1.044	0.062	0.008	0.019
Q3U3A7	Mta3	1.065	0.091	0.179	0.231
Q3U3Q1	Ulk3	1.025	0.036	0.662	0.711
Q3U422	Ndufv3	0.927	(0.109)	0.024	0.044
Q3U435	Mmp25	0.857	(0.223)	0.005	0.013
Q3U4G0		0.872	(0.197)	0.002	0.008
Q3U4G3	Xxylt1	0.923	(0.116)	0.041	0.068
Q3U4H6	Hexd	0.953	(0.069)	0.497	0.555
Q3U4S0	Pank2	1.032	0.045	0.229	0.285

Q3U4W8	Usp5	0.977	(0.034)	0.161	0.211
Q3U4X8	Lig1	1.109	0.150	0.008	0.019
Q3U5Q7	Cmpk2	0.992	(0.012)	0.930	0.942
Q3U5V8	Stx11	0.970	(0.044)	0.540	0.596
Q3U687	Ifit1bl2	0.867	(0.205)	0.453	0.512
Q3U6F1	Rbpj	0.881	(0.183)	0.027	0.049
Q3U6Q4	Pik3r6	0.958	(0.062)	0.431	0.490
Q3U741	Ddx17	1.174	0.232	0.001	0.006
Q3U7K7	Trim21	1.097	0.134	0.194	0.248
Q3U7R1	Esyt1	1.056	0.079	0.160	0.211
Q3U821	Wdr75	1.000	0.000	0.994	0.995
Q3U9N9	Slc16a10	1.194	0.256	0.007	0.019
Q3UA06	Trip13	1.164	0.219	0.019	0.037
Q3UB74	Tbrg1	0.887	(0.172)	0.001	0.006
Q3UBX0	Tmem109	1.021	0.030	0.529	0.586
Q3UCV8	Otulin	0.985	(0.021)	0.503	0.561
Q3UDE2	Ttll12	1.128	0.174	0.004	0.013
Q3UDK1	Trafd1	0.837	(0.258)	0.219	0.275
Q3UDR8	Yipf3	0.957	(0.063)	0.138	0.184
Q3UDS7	Adpgk	0.865	(0.209)	0.000	0.003
Q3UDW8	Hgsnat	1.099	0.136	0.003	0.010
Q3UE37	Ube2z	1.010	0.015	0.746	0.786
Q3UE92	Xpnpep1	0.936	(0.095)	0.012	0.027
Q3UEB3	Puf60	1.188	0.249	0.004	0.012
Q3UFK8	Frmd8	0.887	(0.172)	0.003	0.010
Q3UFN1	Mgll	1.196	0.258	0.021	0.040
Q3UFS0	Zyg11b	0.848	(0.237)	0.012	0.026
Q3UFS4	Gpatch11	0.893	(0.164)	0.068	0.102
Q3UFY7	Nt5c3b	1.036	0.051	0.202	0.257
Q3UFY8	Trmt10c	1.120	0.163	0.056	0.087
Q3UGC7	Eif3j1	0.972	(0.040)	0.525	0.583
Q3UH06	Rreb1	0.911	(0.135)	0.003	0.010
Q3UH28	Zmynd8	1.056	0.079	0.029	0.051
Q3UHC2	Lrrk1	0.926	(0.111)	0.008	0.020
Q3UHD6	Snx27	0.951	(0.073)	0.025	0.047
Q3UHD9	Agap2	0.851	(0.232)	0.004	0.012
Q3UHJ0	Aak1	1.065	0.091	0.051	0.081
Q3UHP6	Anks1	0.939	(0.090)	0.043	0.070
Q3UHX0	Nol8	1.016	0.023	0.486	0.545
Q3UHX2	Pdap1	0.951	(0.072)	0.216	0.272
Q3UHX9	Spout1	0.986	(0.021)	0.544	0.600
Q3UI43	Babam1	1.041	0.058	0.227	0.283
Q3UID0	Smarcc2	1.017	0.024	0.386	0.446
Q3UIR3	Dtx3l	1.017	0.025	0.833	0.862
Q3UIX4	Srsf11	1.059	0.083	0.011	0.024
Q3UJB0	Sf3b2	1.062	0.087	0.061	0.093
Q3UJP5		0.908	(0.140)	0.081	0.118
Q3UKC1	Tax1bp1	0.939	(0.091)	0.030	0.053
Q3UKJ7	Smu1	1.067	0.093	0.008	0.019
Q3UKP1	Sirt6	1.028	0.040	0.416	0.476
Q3UL36	Arglu1	1.024	0.034	0.276	0.335
Q3ULB1	Tes	0.961	(0.058)	0.045	0.073
Q3ULD5	Mccc2	1.108	0.147	0.057	0.088
Q3ULL6	Upf3b	0.887	(0.174)	0.016	0.032
Q3ULW8	Parp3	1.144	0.195	0.007	0.017
Q3UM18	Lsg1	0.865	(0.208)	0.009	0.021
Q3UM45	Ppp1r7	1.034	0.049	0.074	0.109
Q3UMB9	Washc4	0.894	(0.161)	0.000	0.004
Q3UMP4	Serbp1	1.079	0.110	0.111	0.153
Q3UMR0	Ankrd27	0.967	(0.048)	0.486	0.545
Q3UMT1	Ppp1r12c	0.850	(0.234)	0.002	0.008

Q3UMU9	Hdgfl2	0.987	(0.019)	0.413	0.473
Q3UMW8	Cln5	0.962	(0.056)	0.012	0.027
Q3UN90	Lyrm9	1.020	0.029	0.676	0.724
Q3UND0	Skap2	0.856	(0.224)	0.003	0.010
Q3UPL0	Sec31a	0.877	(0.189)	0.000	0.005
Q3UQ44	Iqgap2	0.897	(0.157)	0.000	0.003
Q3UQN2	Fcho2	0.947	(0.079)	0.011	0.025
Q3UQU0	Brd9	0.910	(0.136)	0.007	0.018
Q3UR70	Tgfbrap1	0.897	(0.157)	0.069	0.103
Q3URQ0	Tex10	0.892	(0.165)	0.001	0.006
Q3URS9	Ccdc51	1.072	0.100	0.146	0.194
Q3USH5	Sfswap	1.086	0.119	0.003	0.011
Q3USJ8	Fchsd2	0.962	(0.056)	0.299	0.359
Q3UT69	Gabpb1	0.924	(0.114)	0.111	0.153
Q3UTD9	Smim15	0.905	(0.144)	0.009	0.022
Q3UV17	Krt76	1.305	0.384	0.683	0.730
Q3UVG3	Fam91a1	0.998	(0.003)	0.974	0.977
Q3UVK0	Ermp1	0.897	(0.157)	0.005	0.013
Q3UVL4	Vps51	0.895	(0.160)	0.028	0.051
Q3UW53	Niban1	1.008	0.011	0.713	0.757
Q3UXR7	Pot1a	1.131	0.178	0.053	0.083
Q3UXU8	Stn1	1.075	0.104	0.016	0.033
Q3UXZ9	Kdm5a	1.180	0.239	0.025	0.047
Q3UYC0	Ppm1h	0.963	(0.054)	0.409	0.470
Q3UYU2	Trmt2a	1.050	0.070	0.106	0.148
Q3UYV9	Ncbp1	1.134	0.181	0.001	0.007
Q3UZA1	Rcsd1	0.882	(0.181)	0.005	0.013
Q3UZP4	Svip	0.920	(0.121)	0.043	0.070
Q3UZZ4	Olfm4	0.909	(0.137)	0.384	0.444
Q3V009	Tmed1	1.014	0.020	0.450	0.510
Q3V117	Acly	0.947	(0.079)	0.019	0.038
Q3V157	Stat4	0.942	(0.087)	0.018	0.035
Q3V1G4	Olfml2b	0.877	(0.190)	0.099	0.139
Q3V1L4	Nt5c2	1.018	0.026	0.324	0.385
Q3V300	Kif22	1.171	0.228	0.001	0.006
Q3V384	Afg1l	0.932	(0.101)	0.078	0.114
Q3V3E1	Ubash3a	0.844	(0.246)	0.003	0.011
Q3V3R1	Mthfd1l	1.167	0.223	0.001	0.007
Q3V4B5	Commd6	0.923	(0.116)	0.014	0.029
Q3ZAQ4	Atg9a	0.939	(0.091)	0.019	0.037
Q3ZAR9	Nr2c2	1.134	0.182	0.000	0.004
Q45VK5	Ilf3	1.117	0.160	0.002	0.007
Q497J0	Cstdc5	0.476	(1.072)	0.071	0.105
Q4FCQ7	Ate1	0.944	(0.083)	0.004	0.012
Q4FE56	Usp9x	0.910	(0.135)	0.004	0.012
Q4FK66	Prpf38a	1.075	0.104	0.010	0.023
Q4FZC9	Syne3	0.875	(0.193)	0.001	0.006
Q4G0F8	Ubn1	1.100	0.137	0.004	0.013
Q4LDD4	Arap1	0.931	(0.103)	0.001	0.006
Q4PJX1	Odr4	0.848	(0.238)	0.012	0.026
Q4QRL3	Ccdc88b	0.866	(0.208)	0.001	0.007
Q4VA53	Pds5b	1.040	0.057	0.031	0.054
Q4VA93	Prkca	0.883	(0.179)	0.293	0.352
Q4VAA2	Cdv3	1.022	0.031	0.459	0.518
Q4VBF8	Sipa1l1	0.917	(0.125)	0.044	0.072
Q4VC33	Maea	0.880	(0.184)	0.040	0.067
Q505B7	Zbtb8os	1.066	0.092	0.030	0.053
Q505F5	Lrrc47	1.031	0.044	0.040	0.067
Q52KE7	Ccnl1	0.933	(0.101)	0.040	0.066
Q542V3	Srsf4	1.195	0.257	0.000	0.004
Q569Z5	Ddx46	1.073	0.102	0.004	0.013

Q569Z6	Thrap3	1.190	0.251	0.000	0.003
Q56A08	Gpkow	0.999	(0.001)	0.975	0.978
Q571E4	Galns	0.844	(0.245)	0.000	0.004
Q571H0	Urb1	0.844	(0.245)	0.045	0.073
Q58A65	Spag9	1.039	0.055	0.121	0.165
Q59J78	Ndufaf2	0.956	(0.064)	0.075	0.111
Q5DTM8	Rnf20	0.996	(0.005)	0.715	0.759
Q5DU31	Ipcef1	0.995	(0.008)	0.834	0.863
Q5EBP8	Hnrnpa1	1.128	0.174	0.000	0.004
Q5F258	Git1	0.902	(0.148)	0.004	0.012
Q5F284	Plscr3	0.872	(0.198)	0.048	0.076
Q5F2E7	Nufip2	0.903	(0.147)	0.010	0.022
Q5F2E8	Taok1	0.869	(0.202)	0.076	0.112
Q5FWK3	Arhgap1	1.030	0.042	0.238	0.294
Q5H8C4	Vps13a	1.003	0.004	0.849	0.875
Q5HZJ0	Drosha	0.855	(0.226)	0.032	0.056
Q5KU39	Vps41	0.924	(0.114)	0.021	0.041
Q5M8M8	Rpl29	0.957	(0.063)	0.567	0.623
Q5NC03	Limk2	0.919	(0.121)	0.085	0.122
Q5NC05	Ttf2	0.874	(0.194)	0.007	0.019
Q5NCE8	Mrs2	0.859	(0.218)	0.026	0.047
Q5NCJ0	Rapgef6	0.921	(0.119)	0.000	0.003
Q5NCQ5	Dph1	0.980	(0.030)	0.593	0.647
Q5NCU4	Sparc	1.246	0.317	0.131	0.177
Q5ND34	Wdr81	1.052	0.072	0.081	0.118
Q5PPR2	Exoc1	0.908	(0.140)	0.000	0.004
Q5PR72	Pde2a	1.003	0.004	0.916	0.930
Q5PRE5	Proser1	0.915	(0.128)	0.218	0.273
Q5PRF0	Heatr5a	0.856	(0.224)	0.063	0.095
Q5RKN9	Capza1	1.093	0.129	0.091	0.130
Q5RKZ7	Mocs1	1.006	0.008	0.693	0.739
Q5RL20	Mrpl43	1.081	0.112	0.165	0.216
Q5SFM8	Rbm27	1.142	0.192	0.006	0.016
Q5SNZ0	Ccdc88a	0.967	(0.048)	0.398	0.459
Q5SQX6	Cyfip2	1.068	0.095	0.006	0.015
Q5SSI6	Utp18	1.086	0.120	0.004	0.012
Q5SSW2	Psme4	0.979	(0.031)	0.051	0.081
Q5SSZ5	Tns3	0.956	(0.065)	0.137	0.183
Q5SU73	Coil	1.055	0.077	0.132	0.179
Q5SUA5	Myo1g	0.920	(0.120)	0.064	0.096
Q5SUC9	Sco1	1.052	0.073	0.144	0.192
Q5SUF2	Luc7l3	1.048	0.068	0.032	0.055
Q5SUH7	Clint1	0.918	(0.123)	0.034	0.058
Q5SUR0	Pfas	1.186	0.246	0.000	0.003
Q5SV02	Rad50	1.067	0.094	0.010	0.023
Q5SVD0	Rflnb	0.911	(0.134)	0.021	0.041
Q5SVG5	Ap1b1	1.069	0.097	0.011	0.024
Q5SVP2	)30111J21Ril	1.013	0.019	0.859	0.884
Q5SVQ0	Kat7	1.010	0.015	0.735	0.776
Q5SVR0	Tbc1d9b	1.061	0.085	0.134	0.181
Q5SW15	Mettl16	1.160	0.214	0.009	0.021
Q5SW75	Ssh2	0.882	(0.181)	0.004	0.013
Q5SWD9	Tsr1	0.919	(0.122)	0.026	0.048
Q5SWW4	Med13	0.867	(0.206)	0.001	0.007
Q5SX39	Myh4	1.094	0.130	0.821	0.852
Q5SX40	Myh1	0.960	(0.059)	0.911	0.927
Q5SXC4	Vezf1	1.014	0.020	0.688	0.734
Q5SXR4	Dhx40	0.916	(0.127)	0.193	0.246
Q5SXR6	Cltc	1.180	0.239	0.002	0.008
Q5U3K5	Rabl6	0.936	(0.095)	0.003	0.010
Q5U3L0	Sh3bp2	0.843	(0.247)	0.001	0.006

Q5U458	Dnajc11	1.061	0.085	0.013	0.028
Q5U4C3	Scaf1	1.050	0.070	0.023	0.044
Q5U5M8	Bloc1s3	0.996	(0.005)	0.967	0.971
Q5UAK0	Mier1	0.993	(0.010)	0.654	0.703
Q5UE59	Klc1	1.042	0.060	0.000	0.003
Q5XG71	Utp20	1.011	0.015	0.673	0.721
Q5XJE5	Leo1	1.011	0.016	0.650	0.700
Q5XJV5	Ncoa6	0.915	(0.128)	0.003	0.010
Q5XJY5	Arcn1	1.004	0.006	0.702	0.747
Q5Y5T1	Zdhhc20	1.178	0.237	0.012	0.027
Q60520	Sin3a	1.066	0.092	0.009	0.022
Q60596	Xrcc1	1.020	0.029	0.669	0.718
Q60597	Ogdh	1.154	0.207	0.005	0.014
Q60604	Scin	1.143	0.192	0.054	0.084
Q60605	Myl6	1.019	0.027	0.231	0.288
Q60631	Grb2	1.022	0.031	0.225	0.281
Q60648	Gm2a	0.901	(0.150)	0.010	0.023
Q60649	Clpb	0.883	(0.179)	0.030	0.053
Q60692	Psmb6	0.860	(0.218)	0.008	0.020
Q60710	Samhd1	1.076	0.106	0.145	0.193
Q60715	P4ha1	1.017	0.024	0.863	0.887
Q60737	Csnk2a1	1.059	0.082	0.093	0.132
Q60749	Khdrbs1	1.022	0.031	0.451	0.511
Q60770	Stxbp3	1.052	0.074	0.004	0.011
Q60772	Cdkn2c	0.924	(0.114)	0.058	0.090
Q60787	Lcp2	0.872	(0.198)	0.016	0.033
Q60790	Rasa3	1.026	0.038	0.367	0.427
Q60809	Cnot7	0.946	(0.081)	0.024	0.045
Q60817	Naca	0.976	(0.035)	0.533	0.590
Q60823	Akt2	0.918	(0.123)	0.009	0.022
Q60855	Ripk1	0.854	(0.227)	0.018	0.035
Q60862	Orc2	1.031	0.044	0.319	0.380
Q60864	Stip1	1.005	0.008	0.739	0.780
Q60872	Eif1a	0.836	(0.259)	0.004	0.013
Q60876	Eif4ebp1	0.885	(0.176)	0.101	0.142
Q60931	Vdac3	1.109	0.150	0.003	0.010
Q60949	Tbc1d1	0.880	(0.185)	0.001	0.007
Q60952	Cep250	0.939	(0.090)	0.004	0.012
Q60960	Kpna1	0.986	(0.021)	0.148	0.196
Q60972	Rbbp4	1.094	0.130	0.018	0.036
Q60973	Rbbp7	0.986	(0.021)	0.734	0.775
Q60974	Ncor1	1.025	0.035	0.060	0.093
Q60996	Ppp2r5c	1.061	0.085	0.076	0.111
Q61024	Asns	1.014	0.020	0.784	0.819
Q61026	Ncoa2	0.979	(0.030)	0.120	0.164
Q61033	Tmpo	1.116	0.158	0.000	0.004
Q61035	Hars	0.977	(0.034)	0.417	0.476
Q61074	Ppm1g	1.086	0.119	0.038	0.063
Q61081	Cdc37	0.953	(0.069)	0.219	0.274
Q61093	Cybb	0.936	(0.096)	0.016	0.032
Q61102	Abcb7	1.077	0.107	0.027	0.049
Q61107	Gbp4	0.866	(0.207)	0.026	0.047
Q61112	Sdf4	0.923	(0.116)	0.006	0.016
Q61136	Prpf4b	1.106	0.145	0.000	0.002
Q61142	Spin1	1.233	0.302	0.066	0.099
Q61151	Ppp2r5e	0.977	(0.034)	0.772	0.808
Q61160	Fadd	0.956	(0.065)	0.198	0.252
Q61161	Map4k2	1.095	0.131	0.043	0.070
Q61164	Ctcf	1.147	0.198	0.061	0.093
Q61165	Slc9a1	1.006	0.008	0.803	0.837
Q61166	Mapre1	1.035	0.050	0.233	0.289

Q61179	Irf9	0.933	(0.100)	0.581	0.637
Q61187	Tsg101	0.985	(0.022)	0.737	0.778
Q61193	Rgl2	0.893	(0.163)	0.004	0.013
Q61216	Mre11	1.166	0.221	0.000	0.003
Q61233	Lcp1	0.842	(0.248)	0.001	0.006
Q61239	Fnta	1.134	0.182	0.158	0.208
Q61249	Igbp1	0.902	(0.149)	0.043	0.070
Q61334	Bcap29	1.087	0.120	0.050	0.079
Q61335	Bcap31	1.024	0.034	0.301	0.361
Q61425	Hadh	1.038	0.054	0.066	0.099
Q61466	Smarcd1	1.172	0.229	0.001	0.006
Q61503	Nt5e	1.006	0.009	0.802	0.835
Q61510	Trim25	0.959	(0.060)	0.243	0.300
Q61549	Adgre1	1.123	0.168	0.018	0.035
Q61550	Rad21	1.025	0.035	0.016	0.033
Q61554	Fbn1	0.917	(0.125)	0.412	0.472
Q61578	Fdxr	1.031	0.045	0.225	0.281
Q61584	Fxr1	0.892	(0.165)	0.001	0.005
Q61598	Gdi2	1.093	0.128	0.002	0.008
Q61599	Arhgdib	1.056	0.078	0.534	0.590
Q61655	Ddx19a	0.969	(0.045)	0.343	0.404
Q61656	Ddx5	1.173	0.231	0.001	0.007
Q61687	Atrx	1.053	0.075	0.016	0.032
Q61696	Hspa1a	0.897	(0.156)	0.149	0.198
Q61699	Hsph1	1.161	0.215	0.000	0.004
Q61712	Dnajc1	0.839	(0.253)	0.000	0.002
Q61733	Mrps31	1.118	0.161	0.001	0.007
Q61735	Cd47	1.108	0.148	0.076	0.111
Q61739	Itga6	0.901	(0.150)	0.019	0.037
Q61753	Phgdh	1.096	0.132	0.175	0.227
Q61768	Kif5b	0.918	(0.123)	0.001	0.007
Q61792	Lasp1	1.028	0.040	0.328	0.388
Q61818	Rai1	1.100	0.138	0.010	0.023
Q61823	Pdcd4	1.151	0.203	0.025	0.045
Q61838	Pzp	0.853	(0.230)	0.029	0.052
Q61937	Npm1	1.158	0.212	0.005	0.014
Q61990	Pcbp2	0.944	(0.082)	0.052	0.081
Q62018	Ctr9	0.962	(0.056)	0.039	0.065
Q62074	Prkci	0.939	(0.091)	0.267	0.325
Q62086	Pon2	1.113	0.155	0.009	0.021
Q62087	Pon3	0.887	(0.174)	0.104	0.145
Q62093	Srsf2	1.110	0.150	0.014	0.029
Q62095	Ddx3y	1.187	0.248	0.004	0.012
Q62167	Ddx3x	0.918	(0.124)	0.313	0.374
Q62172	Ralbp1	0.840	(0.252)	0.002	0.008
Q62178	Sema4a	0.886	(0.175)	0.001	0.005
Q62187	Ttf1	1.083	0.115	0.024	0.044
Q62189	Snrpa	1.062	0.086	0.078	0.114
Q62192	Cd180	0.997	(0.005)	0.934	0.945
Q62261	Sptbn1	0.933	(0.100)	0.008	0.019
Q62312	Tgfbr2	1.170	0.227	0.003	0.010
Q62347	Creb1	1.088	0.122	0.018	0.036
Q62348	Tsn	1.074	0.103	0.007	0.018
Q62376	Snrnp70	1.128	0.174	0.002	0.008
Q62383	Supt6h	1.037	0.052	0.377	0.438
Q62419	Sh3gl1	0.952	(0.071)	0.013	0.028
Q62426	Cstb	1.028	0.040	0.325	0.385
Q62431	Arid3a	0.876	(0.190)	0.025	0.046
Q62446	Fkbp3	1.133	0.181	0.032	0.056
Q62465	Vat1	1.021	0.030	0.410	0.470
Q62469	Itga2	1.516	0.601	0.112	0.154

Q62523	Zyx	0.926	(0.111)	0.045	0.074
Q63810	Ppp3r1	0.869	(0.203)	0.000	0.003
Q63829	Commd3	0.982	(0.026)	0.363	0.424
Q63850	Nup62	1.029	0.042	0.317	0.377
Q63918	Cavin2	1.035	0.049	0.767	0.804
Q64010	Crk	1.185	0.245	0.004	0.011
Q640M1	Utp14a	1.160	0.214	0.001	0.005
Q64131	Runx3	1.054	0.076	0.435	0.494
Q64133	Maoa	0.851	(0.233)	0.001	0.007
Q64152	Btf3	1.005	0.007	0.948	0.955
Q64261	Cdk6	1.109	0.149	0.032	0.055
Q64282	Ifit1	0.817	(0.292)	0.310	0.370
Q64314	Cd34	0.938	(0.093)	0.086	0.123
Q64324	Stxbp2	0.958	(0.062)	0.276	0.335
Q64337	Sqstm1	0.888	(0.171)	0.003	0.010
Q64339	Isg15	1.253	0.325	0.352	0.412
Q64378	Fkbp5	0.945	(0.081)	0.206	0.261
Q64442	Sord	0.933	(0.100)	0.003	0.010
Q64514	Tpp2	1.111	0.152	0.000	0.003
Q64520	Guk1	0.930	(0.104)	0.021	0.040
Q64521	Gpd2	0.926	(0.111)	0.002	0.008
Q64523	Hist2h2ac	1.034	0.049	0.270	0.328
Q64701	Rbl1	0.957	(0.063)	0.002	0.008
Q64704	Stx3	0.843	(0.246)	0.002	0.009
Q64737	Gart	1.095	0.131	0.054	0.084
Q66JV4	Rbm12b2	1.051	0.071	0.058	0.089
Q66JX5	Fgfr1op	1.022	0.031	0.352	0.413
Q68FE6	Ripor1	0.944	(0.084)	0.109	0.152
Q68FG3	Spty2d1	0.897	(0.157)	0.021	0.039
Q68FH4	Galk2	0.893	(0.164)	0.004	0.012
Q68FL6	Mars	1.012	0.018	0.698	0.743
Q69Z66	Mysm1	1.055	0.077	0.217	0.273
Q69Z99	Znf512	1.143	0.193	0.007	0.019
Q69ZA1	Cdk13	0.953	(0.069)	0.008	0.019
Q69ZC8	Gpalpp1	0.917	(0.126)	0.127	0.172
Q69ZK0	Prex1	0.957	(0.063)	0.083	0.120
Q69ZK6	Jmjd1c	1.051	0.071	0.031	0.054
Q69ZN6	Gnptab	1.009	0.014	0.744	0.784
Q69ZQ2	Isy1	1.100	0.138	0.036	0.061
Q69ZR9	Tasor	0.914	(0.130)	0.175	0.227
Q69ZX6	Morc2a	0.940	(0.089)	0.048	0.076
Q6A009	Ltn1	0.962	(0.056)	0.541	0.597
Q6A026	Pds5a	1.010	0.014	0.538	0.595
Q6A028	Swap70	1.104	0.143	0.003	0.010
Q6A068	Cdc5l	1.199	0.261	0.001	0.007
Q6A0A2	Larp4b	0.900	(0.152)	0.018	0.036
Q6A0A9	FAM120A	1.052	0.074	0.064	0.096
Q6BCL1	Pram1	0.859	(0.219)	0.014	0.029
Q6DFV1	Ncapg2	0.979	(0.031)	0.281	0.340
Q6DFW5	Atp11b	0.846	(0.242)	0.000	0.003
Q6DFZ1	Gbf1	0.865	(0.210)	0.009	0.021
Q6DID3	Scaf8	1.089	0.123	0.013	0.028
Q6GQT6	Scap	0.922	(0.117)	0.074	0.109
Q6GQT9	Nomo1	0.912	(0.133)	0.002	0.008
Q6GT24	Prdx6	0.959	(0.060)	0.225	0.281
Q6GYP7	Ralgapa1	0.898	(0.154)	0.002	0.008
Q6IE82	Jade3	1.128	0.174	0.064	0.097
Q6IRU2	Tpm4	1.090	0.124	0.081	0.118
Q6IRU5	Cltb	0.980	(0.028)	0.206	0.261
Q6KAR6	Exoc3	0.893	(0.163)	0.003	0.010
Q6KCD5	Nipbl	1.066	0.092	0.005	0.014

Q6NS46	Pdcd11	1.168	0.224	0.001	0.007
Q6NSQ7	Ltv1	1.062	0.087	0.251	0.308
Q6NSR8	Npepl1	1.098	0.135	0.026	0.047
Q6NT99	Dusp23	0.947	(0.078)	0.107	0.149
Q6NV52	Tacc3	0.902	(0.150)	0.007	0.018
Q6NV83	U2surp	1.073	0.102	0.002	0.009
Q6NV99	Haus6	0.846	(0.241)	0.008	0.020
Q6NVE8	Wdr44	0.975	(0.037)	0.048	0.077
Q6NVF4	Helb	1.033	0.047	0.104	0.146
Q6NVF9	Cpsf6	1.141	0.191	0.003	0.010
Q6NXH2	Manea	0.899	(0.153)	0.067	0.101
Q6NXI6	Rprd2	0.899	(0.153)	0.008	0.019
Q6NZB0	Dnajc8	1.099	0.137	0.006	0.016
Q6NZF1	Zc3h11a	1.053	0.074	0.021	0.041
Q6NZJ6	Eif4g1	1.009	0.013	0.713	0.757
Q6NZL6	Tonsl	0.940	(0.089)	0.027	0.049
Q6NZM8	Papss1	0.890	(0.168)	0.023	0.043
Q6NZQ2	Ddx31	1.001	0.002	0.964	0.969
Q6NZR5	Skiv2l	0.910	(0.135)	0.001	0.007
Q6P069	Sri	0.876	(0.191)	0.001	0.006
Q6P1E1	Zmiz1	0.964	(0.053)	0.080	0.116
Q6P1F6	Ppp2r2a	1.010	0.015	0.354	0.415
Q6P1G0	Heatr6	0.837	(0.256)	0.010	0.023
Q6P1H6	Ankle2	0.938	(0.093)	0.017	0.035
Q6P2K6	Ppp4r3a	1.057	0.080	0.050	0.079
Q6P3A9	Arl11	1.073	0.102	0.197	0.251
Q6P3E7	Hdac10	1.096	0.132	0.095	0.135
Q6P3Y5	Znf280c	0.999	(0.002)	0.957	0.963
Q6P4S8	Ints1	0.938	(0.092)	0.012	0.027
Q6P4T2	Snrnp200	1.143	0.193	0.000	0.003
Q6P542	Abcf1	1.096	0.133	0.007	0.018
Q6P5B0	Rrp12	1.140	0.189	0.003	0.010
Q6P5B5	Fxr2	1.050	0.070	0.065	0.098
Q6P5D3	Dhx57	0.917	(0.124)	0.056	0.087
Q6P5D4	Cep135	1.085	0.118	0.053	0.083
Q6P5D8	Smchd1	1.166	0.222	0.000	0.004
Q6P5E4	Uggt1	0.859	(0.219)	0.000	0.004
Q6P5E6	Gga2	1.137	0.185	0.007	0.017
Q6P5F7	Ttyh3	0.889	(0.169)	0.072	0.107
Q6P5F9	Xpo1	1.077	0.107	0.001	0.007
Q6P6I6	Polr2m	0.968	(0.047)	0.077	0.113
Q6P6L6	Gemin4	1.035	0.050	0.515	0.573
Q6P6M7	Sepsecs	1.124	0.169	0.038	0.064
Q6P6P5	Gm21985	1.073	0.102	0.050	0.079
Q6P8I4	Pcnp	0.939	(0.091)	0.171	0.223
Q6P8X1	Snx6	0.957	(0.064)	0.213	0.268
Q6P9J9	Ano6	1.049	0.069	0.158	0.207
Q6P9L6	Kif15	0.914	(0.129)	0.025	0.046
Q6P9P6	Kif11	1.008	0.012	0.841	0.869
Q6P9Q6	Fkbp15	0.935	(0.097)	0.000	0.004
Q6P9R1	Ddx51	1.173	0.231	0.000	0.005
Q6P9R2	Oxsr1	1.116	0.158	0.000	0.004
Q6P9R4	Arhgef18	0.843	(0.246)	0.001	0.005
Q6PA06	Atl2	1.182	0.241	0.011	0.025
Q6PAC3	Dcaf13	0.996	(0.006)	0.906	0.923
Q6PAJ1	Bcr	1.065	0.091	0.061	0.093
Q6PAM1	Txlna	1.028	0.039	0.189	0.243
Q6PAR5	Gapvd1	1.013	0.018	0.446	0.505
Q6PAV2	Herc4	1.031	0.043	0.087	0.125
Q6PB44	Ptpn23	0.922	(0.118)	0.016	0.032
Q6PB51	Ccdc117	1.072	0.101	0.041	0.068

Q6PB66	Lrpprc	1.106	0.146	0.008	0.020
Q6PB93	Galnt2	0.874	(0.194)	0.001	0.005
Q6PD03	Ppp2r5a	1.082	0.113	0.032	0.056
Q6PD19	Armh3	0.931	(0.104)	0.018	0.035
Q6PD26	Pigs	0.935	(0.097)	0.082	0.119
Q6PDI5	Ecpas	0.921	(0.118)	0.038	0.064
Q6PDJ6	Fbxo42	0.930	(0.105)	0.004	0.013
Q6PDL0	Dync1li2	1.016	0.022	0.245	0.302
Q6PDN3	Mylk	0.880	(0.185)	0.012	0.026
Q6PDY2	Ado	0.981	(0.028)	0.201	0.256
Q6PE01	Snrnp40	1.070	0.098	0.008	0.019
Q6PE15	Abhd10	0.997	(0.004)	0.836	0.864
Q6PFB2	Rcc1	1.183	0.243	0.001	0.005
Q6PFQ7	Rasa4	0.985	(0.022)	0.597	0.650
Q6PFY1	Fchsd1	0.926	(0.112)	0.071	0.106
Q6PGB6	Naa50	1.122	0.167	0.004	0.012
Q6PGC1	Dhx29	0.980	(0.029)	0.256	0.314
Q6PGF5	Bms1	0.929	(0.107)	0.100	0.141
Q6PGF7	Exoc8	0.882	(0.180)	0.000	0.003
Q6PGG2	Gmip	0.874	(0.193)	0.001	0.005
Q6PGG6	Gnl3l	1.164	0.219	0.012	0.026
Q6PGH1	Bud31	0.989	(0.015)	0.742	0.782
Q6PGH2	Jpt2	0.930	(0.104)	0.102	0.143
Q6PGL7	Washc2	0.878	(0.187)	0.007	0.018
Q6PHN9	Rab35	1.177	0.235	0.004	0.011
Q6PHQ8	Naa35	0.963	(0.055)	0.028	0.050
Q6PHQ9	Pabpc4	1.146	0.196	0.008	0.020
Q6PIU9		0.954	(0.068)	0.057	0.088
Q6PR54	Rif1	1.129	0.175	0.001	0.005
Q6PZD9	Cebpe	0.852	(0.232)	0.006	0.017
Q6Q783	Kmt5c	1.010	0.015	0.698	0.743
Q6Q899	Ddx58	1.005	0.007	0.946	0.954
Q6QD59	Bnip1	1.117	0.160	0.003	0.010
Q6R891	Ppp1r9b	1.010	0.015	0.393	0.453
Q6S5J6	Krit1	0.913	(0.132)	0.210	0.265
Q6SKR2	N6amt1	1.100	0.137	0.017	0.034
Q6URW6	Myh14	0.909	(0.137)	0.085	0.123
Q6V595	Klhl6	0.935	(0.097)	0.405	0.465
Q6VGS5	Ccdc88c	0.900	(0.152)	0.016	0.033
Q6VN19	Ranbp10	1.095	0.131	0.092	0.131
Q6VYH9	Hsh2d	1.121	0.165	0.369	0.430
Q6WKZ8	Ubr2	0.945	(0.081)	0.017	0.034
Q6X893	Slc44a1	0.999	(0.002)	0.986	0.988
Q6XLQ8	Calu	1.042	0.060	0.307	0.368
Q6Y685	Tacc1	0.875	(0.192)	0.000	0.003
Q6ZPE2	Sbf1	0.978	(0.032)	0.063	0.095
Q6ZPJ0	Tex2	0.903	(0.147)	0.091	0.130
Q6ZPR6	Ibtk	0.988	(0.018)	0.754	0.792
Q6ZPU9	Kif1bp	0.927	(0.109)	0.089	0.128
Q6ZPY2	Smg5	0.890	(0.168)	0.102	0.144
Q6ZPZ3	Zc3h4	1.079	0.110	0.017	0.035
Q6ZQ03	Fnbp4	0.998	(0.003)	0.908	0.924
Q6ZQ06	Cep162	0.879	(0.186)	0.276	0.335
Q6ZQ38	Cand1	0.992	(0.012)	0.658	0.707
Q6ZQ58	Larp1	0.914	(0.129)	0.172	0.224
Q6ZQ73	Cand2	0.517	(0.952)	0.098	0.138
Q6ZQ88	Kdm1a	0.988	(0.017)	0.666	0.715
Q6ZQ93	Usp34	0.839	(0.253)	0.000	0.004
Q6ZQE4	Nemp1	1.103	0.141	0.000	0.004
Q6ZQF0	Topbp1	1.075	0.104	0.040	0.067
Q6ZQH8	Nup188	0.957	(0.064)	0.209	0.264

Q6ZQI3	Mlec	0.865	(0.210)	0.002	0.008
Q6ZQK0	Ncapd3	1.018	0.026	0.615	0.667
Q6ZQK5	Acap2	1.024	0.034	0.421	0.480
Q6ZQL4	Wdr43	1.032	0.045	0.076	0.112
Q6ZWM4	Lsm8	0.932	(0.102)	0.001	0.005
Q6ZWN5	Rps9	0.906	(0.142)	0.220	0.276
Q6ZWQ7	Spcs3	0.881	(0.183)	0.002	0.009
Q6ZWR6	Syne1	0.853	(0.229)	0.002	0.007
Q6ZWV7	Rpl35	0.926	(0.110)	0.175	0.227
Q6ZWX6	Eif2s1	1.051	0.071	0.193	0.246
Q6ZWY3	Rps27l	0.894	(0.162)	0.004	0.012
Q6ZWY8	Tmsb10	0.879	(0.186)	0.049	0.078
Q6ZWY9	Hist1h2bc	1.046	0.065	0.030	0.053
Q6ZWZ4	Rpl36	1.045	0.064	0.583	0.638
Q6ZWZ6	Rps12-ps3	0.961	(0.057)	0.463	0.522
Q70FJ1	Akap9	0.917	(0.125)	0.001	0.007
Q71RI9	Kyat3	0.907	(0.141)	0.004	0.012
Q76LS9	Mindy1	0.839	(0.254)	0.006	0.016
Q76MZ3	Ppp2r1a	0.989	(0.016)	0.641	0.691
Q78HU3	Mvb12a	1.029	0.041	0.128	0.174
Q78IK2	Atp5md	1.070	0.098	0.157	0.207
Q78IK4	Apool	1.153	0.206	0.006	0.017
Q78JE5	Fbxo22	0.978	(0.032)	0.338	0.398
Q78JT3	Haao	1.036	0.051	0.518	0.575
Q78JW9	Ubfd1	1.031	0.044	0.372	0.433
Q78PG9	Ccdc25	0.916	(0.127)	0.041	0.068
Q78PY7	Snd1	0.995	(0.007)	0.918	0.933
Q78RX3	Smim12	1.001	0.002	0.986	0.988
Q78T54	Vma21	1.001	0.001	0.961	0.966
Q792F9	Itga4	1.074	0.103	0.043	0.070
Q792Z1	Try10	0.877	(0.189)	0.010	0.023
Q7JJ13	Brd2	1.153	0.206	0.004	0.012
Q7M6W1	Rtn1	0.932	(0.102)	0.174	0.226
Q7M6Y3	Picalm	1.004	0.006	0.885	0.906
Q7TMB8	Cyfip1	1.065	0.091	0.034	0.058
Q7TMF3	Ndufa12	1.041	0.057	0.425	0.484
Q7TMG8	Nipsnap2	0.956	(0.065)	0.035	0.060
Q7TMY7	Ipo8	1.042	0.059	0.303	0.364
Q7TN29	Smap2	0.994	(0.009)	0.680	0.728
Q7TNC4	Luc7l2	1.032	0.046	0.076	0.112
Q7TNC9	Inpp5a	0.852	(0.230)	0.004	0.013
Q7TNE3	Spag7	0.931	(0.104)	0.037	0.062
Q7TNG5	Eml2	0.967	(0.048)	0.360	0.421
Q7TNP2	Ppp2r1b	0.923	(0.115)	0.020	0.038
Q7TNS2	Micos10	1.098	0.134	0.058	0.090
Q7TNV0	Dek	1.162	0.216	0.001	0.005
Q7TPE5	Slc7a6os	0.979	(0.031)	0.625	0.675
Q7TPR4	Actn1	0.908	(0.139)	0.057	0.088
Q7TPV4	Mybbp1a	1.131	0.177	0.019	0.037
Q7TQ95	Lnpk	0.951	(0.073)	0.011	0.024
Q7TQI3	Otub1	1.052	0.073	0.013	0.029
Q7TQK4	Exosc3	0.973	(0.039)	0.496	0.555
Q7TQK5	Ccdc93	0.934	(0.098)	0.001	0.007
Q7TS68	Nsun6	1.022	0.032	0.110	0.152
Q7TSG1	Cep120	0.998	(0.003)	0.931	0.942
Q7TSG2	Ctdp1	1.059	0.083	0.003	0.010
Q7TSH2	Phkb	0.862	(0.214)	0.003	0.010
Q7TSH6	Scaf4	1.139	0.188	0.001	0.005
Q7TSI1	Plekhm1	1.166	0.221	0.017	0.034
Q7TSI3	Ppp6r1	0.894	(0.162)	0.004	0.012
Q7TSI6	Vav2	1.175	0.233	0.020	0.038

Q7TSS2	Ube2q1	0.915	(0.129)	0.006	0.015
Q7TSV4	Pgm2	0.902	(0.149)	0.001	0.007
Q7TSZ8	Nacc1	1.153	0.205	0.002	0.009
Q7TT18	Atf7ip	1.028	0.040	0.197	0.251
Q7TT37	Elp1	1.041	0.057	0.002	0.009
Q80SW1	Ahcyl1	0.834	(0.262)	0.009	0.021
Q80SY5	Prpf38b	0.948	(0.077)	0.042	0.068
Q80T06	Eef1d	1.018	0.025	0.688	0.734
Q80T85	Dcaf5	0.967	(0.048)	0.733	0.774
Q80TA6	Mtmr12	0.941	(0.088)	0.008	0.020
Q80TA9	Epg5	0.889	(0.170)	0.000	0.003
Q80TE0	Rpap1	1.044	0.062	0.285	0.344
Q80TG1	Kansl1	0.990	(0.015)	0.673	0.721
Q80TL7	Mon2	0.850	(0.234)	0.004	0.012
Q80TM9	Nisch	1.004	0.006	0.823	0.853
Q80U56	Avl9	0.808	(0.307)	0.080	0.117
Q80U63	Mfn2	0.953	(0.070)	0.085	0.122
Q80U70	Suz12	1.119	0.163	0.002	0.008
Q80U72	Scrib	1.088	0.121	0.003	0.010
Q80U78	Pum1	1.004	0.006	0.873	0.896
Q80U87	Usp8	0.853	(0.229)	0.000	0.003
Q80U93	Nup214	1.037	0.052	0.053	0.083
Q80U95	Ube3c	0.911	(0.135)	0.024	0.045
Q80UF4	Sdccag8	0.925	(0.113)	0.112	0.155
Q80UJ7	Rab3gap1	0.865	(0.209)	0.010	0.023
Q80UK8	Ints2	0.956	(0.065)	0.144	0.191
Q80UM7	Mogs	0.964	(0.053)	0.124	0.169
Q80UN9	Trit1	1.335	0.416	0.102	0.143
Q80UP3	Dgkz	1.002	0.003	0.914	0.929
Q80UP5	Ankrd13a	0.933	(0.099)	0.001	0.005
Q80UR4	Prss34	1.108	0.148	0.027	0.049
Q80UW8	Polr2e	1.117	0.160	0.000	0.005
Q80V26	Impad1	1.008	0.012	0.792	0.827
Q80V94	Ap4e1	0.867	(0.206)	0.007	0.017
Q80VA0	Galnt7	1.024	0.034	0.058	0.090
Q80VD1	Fam98b	1.105	0.145	0.008	0.019
Q80VI1	Trim56	0.971	(0.042)	0.138	0.185
Q80VJ3	Dnph1	1.107	0.146	0.038	0.064
Q80VP0	Tecpr1	1.138	0.187	0.023	0.043
Q80VP5		1.040	0.056	0.063	0.096
Q80VQ0	Aldh3b1	0.951	(0.073)	0.138	0.184
Q80VW7	Akna	0.898	(0.155)	0.098	0.138
Q80W00	Ppp1r10	1.026	0.037	0.134	0.181
Q80W54	Zmpste24	0.875	(0.192)	0.004	0.012
Q80WE4	Kif20b	0.967	(0.049)	0.082	0.119
Q80WG5	Lrrc8a	0.988	(0.017)	0.514	0.572
Q80WJ7	Mtdh	0.965	(0.051)	0.022	0.042
Q80WQ2	Vac14	0.966	(0.050)	0.001	0.007
Q80WQ6	Rhbdf2	0.835	(0.259)	0.002	0.009
Q80WQ8	Mis18bp1	1.109	0.149	0.010	0.024
Q80WR5		1.140	0.189	0.001	0.006
Q80WW9	Ddrgk1	0.914	(0.129)	0.022	0.042
Q80X73	Pelo	0.944	(0.084)	0.058	0.089
Q80X82	Sympk	1.090	0.124	0.003	0.010
Q80X85	Mrps7	1.069	0.097	0.121	0.166
Q80X90	Flnb	0.853	(0.229)	0.002	0.008
Q80X95	Rraga	1.054	0.075	0.000	0.004
Q80X98	Dhx38	0.895	(0.161)	0.018	0.036
Q80XH1	Kxd1	0.873	(0.196)	0.172	0.224
Q80XI4	Pip4k2b	1.027	0.039	0.094	0.134
Q80XK6	Atg2b	0.866	(0.207)	0.001	0.005

Q80XQ2	Tbc1d5	0.836	(0.258)	0.018	0.036
Q80XR5	U2af2	0.951	(0.073)	0.034	0.058
Q80Y55	Bsdc1	0.911	(0.134)	0.002	0.007
Q80Y81	Elac2	1.057	0.080	0.001	0.006
Q80YD1	Supv3l1	1.098	0.135	0.002	0.009
Q80YQ1	Thbs1	1.050	0.071	0.535	0.592
Q80YQ2	Med23	0.952	(0.071)	0.161	0.211
Q80YQ8	Rmnd5a	0.960	(0.058)	0.244	0.301
Q80YR4	Znf598	0.972	(0.041)	0.179	0.232
Q80YR5	Safb2	1.051	0.072	0.001	0.005
Q80YV2	Zc3hc1	1.173	0.230	0.006	0.016
Q80YV4	Pank4	1.005	0.007	0.849	0.875
Q80YW0	Cyth4	0.854	(0.227)	0.020	0.039
Q80ZS3	Mrps26	1.055	0.077	0.074	0.110
Q80ZV0	Rnaseh2b	0.972	(0.041)	0.617	0.668
Q80ZW2	Them6	1.097	0.134	0.018	0.036
Q80ZX0	Sec24b	1.006	0.008	0.624	0.675
Q810A7	Ddx42	1.030	0.043	0.015	0.031
Q810B6	Ankfy1	0.928	(0.109)	0.029	0.052
Q810D6	Grwd1	1.161	0.215	0.002	0.008
Q810V0	Mphosph10	1.067	0.093	0.002	0.008
Q811S7	Ubp1	0.939	(0.090)	0.247	0.303
Q811U4	Mfn1	0.902	(0.148)	0.294	0.354
Q8BFQ4	Wdr82	0.951	(0.072)	0.003	0.010
Q8BFQ8	Gatd1	0.956	(0.065)	0.280	0.339
Q8BFT2	Haus4	0.854	(0.229)	0.000	0.005
Q8BFU3	Rnf214	0.880	(0.185)	0.002	0.008
Q8BFU4	Rgs19	0.904	(0.146)	0.021	0.040
Q8BFV2	Pcid2	1.167	0.222	0.002	0.008
Q8BFW4	Trim65	1.004	0.006	0.835	0.864
Q8BFW7	Lpp	1.097	0.133	0.070	0.104
Q8BFY9	Tnpo1	1.062	0.086	0.030	0.053
Q8BFZ3	Actbl2	1.204	0.268	0.219	0.275
Q8BFZ9	Erlin2	0.984	(0.024)	0.270	0.329
Q8BG15	Ctdspl2	0.924	(0.114)	0.002	0.007
Q8BG17	Nol12	0.942	(0.086)	0.033	0.057
Q8BG30	Nelfa	0.955	(0.067)	0.057	0.088
Q8BG32	Psmd11	1.085	0.117	0.049	0.078
Q8BG48	Stk17b	0.869	(0.202)	0.001	0.006
Q8BG51	Rhot1	1.012	0.018	0.684	0.730
Q8BG67	Efr3a	0.959	(0.061)	0.120	0.165
Q8BG79	Cwf19l2	1.126	0.172	0.009	0.021
Q8BG81	Poldip3	1.195	0.257	0.002	0.008
Q8BGA9	Oxa1l	1.103	0.141	0.252	0.309
Q8BGB5	Limd2	1.037	0.052	0.152	0.201
Q8BGB7	Enoph1	1.024	0.034	0.599	0.652
Q8BGC0	Htatsf1	0.956	(0.065)	0.026	0.047
Q8BGC4	Zadh2	0.923	(0.116)	0.003	0.011
Q8BGD6	Slc38a9	1.157	0.210	0.001	0.006
Q8BGD9	Eif4b	0.943	(0.085)	0.082	0.119
Q8BGF3	Wdr92	0.997	(0.005)	0.795	0.829
Q8BGF7	Pan2	1.020	0.029	0.674	0.722
Q8BGH2	Samm50	1.047	0.066	0.232	0.288
Q8BGJ5	Ptbp1	1.071	0.099	0.003	0.011
Q8BGQ1	Vipas39	1.063	0.088	0.033	0.057
Q8BGQ7	Aars	0.934	(0.098)	0.243	0.300
Q8BGR1	Pdzph1	1.181	0.240	0.086	0.123
Q8BGR8	Gskip	0.866	(0.208)	0.061	0.093
Q8BGS2	Bola2	0.895	(0.161)	0.055	0.085
Q8BGT7	Smndc1	1.047	0.066	0.006	0.016
Q8BGU5	Ccny	0.990	(0.015)	0.661	0.710

Q8BGV0	Nars2	1.039	0.056	0.163	0.214
Q8BGV4	Tti2	1.066	0.092	0.339	0.400
Q8BGX0	Trim23	1.165	0.221	0.003	0.010
Q8BGX2	Timm29	1.115	0.156	0.027	0.049
Q8BH07	Arl6ip6	0.881	(0.183)	0.008	0.020
Q8BH24	Tm9sf4	1.038	0.053	0.243	0.300
Q8BH26	Tmem251	0.871	(0.199)	0.015	0.031
Q8BH40	Stx7	1.009	0.014	0.337	0.397
Q8BH43	Wasf2	1.116	0.158	0.014	0.029
Q8BH48	Ubap1	0.898	(0.155)	0.000	0.004
Q8BH50		1.128	0.173	0.117	0.161
Q8BH57	Wdr48	0.963	(0.054)	0.076	0.112
Q8BH58	Tiprl	1.150	0.201	0.002	0.008
Q8BH59	Slc25a12	1.131	0.177	0.001	0.007
Q8BH69	Sephs1	1.034	0.048	0.147	0.196
Q8BH74	Nup107	1.054	0.076	0.029	0.052
Q8BH79	Ano10	0.914	(0.130)	0.034	0.058
Q8BH80	Vapb	1.069	0.096	0.002	0.008
Q8BH86	Dglucy	0.895	(0.160)	0.010	0.023
Q8BH95	Echs1	1.047	0.067	0.060	0.093
Q8BHA3	Dtd2	0.975	(0.037)	0.255	0.313
Q8BHB4	Wdr3	1.020	0.028	0.561	0.617
Q8BHE8	Maip1	0.964	(0.053)	0.233	0.289
Q8BHG2	Czib	1.188	0.249	0.010	0.022
Q8BHG9	Cggbp1	1.072	0.100	0.207	0.262
Q8BHJ5	Tbl1xr1	0.977	(0.033)	0.107	0.149
Q8BHK9	Ercc6l	0.924	(0.115)	0.019	0.038
Q8BHL3	Tbc1d10b	0.943	(0.084)	0.009	0.021
Q8BHL8	Psmf1	1.188	0.249	0.015	0.031
Q8BHN3	Ganab	0.873	(0.196)	0.002	0.008
Q8BHN5	Rbm45	0.971	(0.042)	0.064	0.097
Q8BHS3	Rbm22	1.159	0.213	0.000	0.003
Q8BHX1	Haus1	0.915	(0.128)	0.152	0.201
Q8BHY2	Noc4l	1.096	0.132	0.001	0.005
Q8BHZ0	Fam49a	1.133	0.180	0.013	0.029
Q8BHZ4	Znf592	1.132	0.178	0.009	0.022
Q8BI22	Cep128	0.881	(0.183)	0.000	0.004
Q8BI72	Cdkn2aip	1.088	0.122	0.004	0.012
Q8BI84	Mia3	0.858	(0.221)	0.001	0.005
Q8BIF9	Znf787	0.994	(0.009)	0.836	0.865
Q8BIG4	Fbxo28	0.955	(0.066)	0.194	0.247
Q8BIJ6	Iars2	1.170	0.227	0.053	0.083
Q8BIJ7	Rufy1	0.953	(0.069)	0.001	0.007
Q8BIP0	Dars2	1.169	0.225	0.003	0.011
Q8BIQ5	Cstf2	0.998	(0.002)	0.881	0.902
Q8BIQ6	Zfp947	0.948	(0.076)	0.415	0.475
Q8BIQ9	Prkag2	0.853	(0.230)	0.001	0.005
Q8BIW1	Prune1	0.966	(0.050)	0.135	0.181
Q8BIZ6	Snip1	1.150	0.201	0.003	0.010
Q8BJ03	Cox15	1.106	0.146	0.006	0.016
Q8BJ05	Zc3h14	1.027	0.038	0.504	0.563
Q8BJ48	Nagpa	0.894	(0.161)	0.036	0.061
Q8BJ64	Chdh	0.950	(0.074)	0.032	0.056
Q8BJ71	Nup93	1.106	0.146	0.000	0.004
Q8BJD1	Itih5	0.952	(0.070)	0.284	0.343
Q8BJF9	Chmp2b	0.949	(0.075)	0.282	0.341
Q8BJL0	Smarcal1	0.889	(0.170)	0.008	0.020
Q8BJL1	Fbxo30	1.002	0.002	0.943	0.952
Q8BJM7	Tyw1	0.952	(0.071)	0.008	0.019
Q8BJS4	Sun2	0.924	(0.114)	0.021	0.040
Q8BJU0	Sgta	1.030	0.042	0.315	0.375

Q8BJW5	Nol11	1.131	0.178	0.059	0.091
Q8BJW6	Eif2a	1.100	0.137	0.002	0.007
Q8BJY1	Psmd5	0.964	(0.053)	0.125	0.170
Q8BJZ4	Mrps35	1.071	0.099	0.034	0.058
Q8BK64	Ahsa1	0.954	(0.067)	0.416	0.476
Q8BK67	Rcc2	1.179	0.238	0.000	0.004
Q8BK72	Mrps27	1.023	0.033	0.589	0.644
Q8BKC5	Ipo5	1.039	0.055	0.119	0.163
Q8BKE6	Cyp20a1	1.078	0.108	0.067	0.101
Q8BKI2	Tnrc6b	0.932	(0.101)	0.014	0.030
Q8BKL6	Tubgcp4	1.008	0.011	0.906	0.923
Q8BKS9	Pum3	1.060	0.085	0.141	0.189
Q8BKT3	Gcfc2	0.903	(0.147)	0.005	0.014
Q8BKT8	Haus7	0.935	(0.097)	0.016	0.033
Q8BKX6	Smg1	0.902	(0.149)	0.008	0.019
Q8BKZ9	Pdhx	0.939	(0.091)	0.072	0.106
Q8BL48	Unk	1.057	0.080	0.007	0.019
Q8BL66	Eea1	0.940	(0.090)	0.002	0.008
Q8BLF6	Idua	0.966	(0.050)	0.074	0.110
Q8BLH7	Hirip3	1.106	0.146	0.048	0.077
Q8BLR9	Hif1an	1.117	0.159	0.000	0.003
Q8BLY2	Tarsl2	1.151	0.202	0.011	0.024
Q8BM72	Hspa13	0.844	(0.245)	0.001	0.005
Q8BM85	Tbck	0.849	(0.236)	0.001	0.005
Q8BM88	Ctso	0.914	(0.130)	0.159	0.209
Q8BMA6	Srp68	0.970	(0.044)	0.401	0.461
Q8BMC4	Nop9	1.049	0.069	0.081	0.117
Q8BMD5	Cdadc1	0.880	(0.185)	0.075	0.110
Q8BMF4	Dlat	1.061	0.085	0.173	0.225
Q8BMG7	Rab3gap2	1.004	0.006	0.826	0.856
Q8BMI0	Fbxo38	1.148	0.200	0.000	0.003
Q8BMJ2	Lars	1.108	0.148	0.003	0.009
Q8BMJ3	Eif1ax	1.033	0.047	0.376	0.437
Q8BMK1	Mettl2	0.969	(0.046)	0.185	0.238
Q8BML9	Qars	1.002	0.003	0.865	0.889
Q8BMQ2	Gtf3c4	1.170	0.227	0.003	0.011
Q8BMS4	Coq3	0.954	(0.068)	0.400	0.460
Q8BMS7	Atp2c1	0.870	(0.202)	0.002	0.009
Q8BMS9	Rassf2	0.890	(0.168)	0.001	0.006
Q8BNQ4	Sp2	1.146	0.197	0.015	0.031
Q8BNU0	Armc6	0.957	(0.063)	0.006	0.016
Q8BNW9	Kbtbd11	1.015	0.021	0.395	0.455
Q8BP27	Sfr1	1.003	0.004	0.936	0.947
Q8BP48	Metap1	0.905	(0.145)	0.000	0.003
Q8BP67	Rpl24	0.956	(0.065)	0.310	0.370
Q8BP92	Rcn2	1.067	0.094	0.000	0.004
Q8BPB0	Mob1b	0.909	(0.137)	0.005	0.014
Q8BPG6	Sumf2	1.030	0.042	0.595	0.649
Q8BPM0	Daam1	0.916	(0.126)	0.023	0.043
Q8BPU7	Elmo1	0.903	(0.148)	0.001	0.005
Q8BQ30	Ppp1r18	1.078	0.108	0.002	0.007
Q8BQ47	Cnpy4	1.111	0.152	0.049	0.078
Q8BQM4	Heatr3	0.974	(0.037)	0.512	0.570
Q8BQX0	Rab37	0.919	(0.122)	0.024	0.044
Q8BQX5	Tmco6	0.896	(0.159)	0.003	0.010
Q8BQZ4	Ralgapb	0.937	(0.095)	0.090	0.129
Q8BR65	Suds3	1.103	0.141	0.117	0.160
Q8BRF7	Scfd1	1.078	0.109	0.000	0.004
Q8BRH0	Tmtc3	0.940	(0.089)	0.108	0.150
Q8BRN9	Cc2d1b	0.836	(0.259)	0.003	0.010
Q8BSK8	Rps6kb1	0.834	(0.262)	0.036	0.061

Q8BSL7	Arf2	1.143	0.193	0.009	0.021
Q8BSP2	Ncaph2	1.032	0.045	0.337	0.397
Q8BSU7	Mob3a	0.946	(0.080)	0.100	0.141
Q8BT07	Cep55	0.886	(0.175)	0.026	0.047
Q8BT51	COA4	0.880	(0.185)	0.015	0.031
Q8BT60	Cpne3	0.901	(0.151)	0.011	0.025
Q8BTE0	Sdhaf4	1.200	0.263	0.002	0.008
Q8BTI8	Srrm2	1.112	0.153	0.001	0.005
Q8BTJ4	Enpp4	0.906	(0.143)	0.037	0.062
Q8BTS4	Nup54	1.015	0.021	0.529	0.586
Q8BTV2	Cpsf7	0.937	(0.094)	0.144	0.192
Q8BTY8	Scfd2	0.865	(0.210)	0.000	0.004
Q8BTZ7	Gmppb	0.948	(0.078)	0.100	0.141
Q8BU03	Pwp2	0.903	(0.148)	0.018	0.036
Q8BU04	Ubr7	1.111	0.151	0.002	0.008
Q8BU11	Tox4	0.951	(0.072)	0.002	0.009
Q8BU14	Sec62	0.896	(0.159)	0.006	0.016
Q8BU30	Iars	1.004	0.006	0.892	0.912
Q8BU31	Rap2c	0.992	(0.011)	0.603	0.656
Q8BU88	Mrpl22	0.879	(0.186)	0.004	0.012
Q8BUB4	Wdfy2	0.843	(0.246)	0.001	0.005
Q8BUH8	Senp7	1.130	0.177	0.062	0.095
Q8BUI3	LRWD1	1.107	0.147	0.014	0.029
Q8BUK6	Hook3	0.909	(0.138)	0.013	0.029
Q8BUV3	Gphn	0.873	(0.196)	0.000	0.003
Q8BUY9	Pggt1b	0.926	(0.110)	0.013	0.028
Q8BV13	Cops7b	0.971	(0.042)	0.009	0.022
Q8BVE8	Nsd2	1.089	0.124	0.012	0.026
Q8BVG4	Dpp9	0.898	(0.155)	0.025	0.046
Q8BVK9	Sp110	1.160	0.214	0.016	0.033
Q8BVL3	Snx17	1.027	0.038	0.182	0.234
Q8BVN4	Mterf4	0.872	(0.197)	0.043	0.071
Q8BVQ0	Wdr61	1.077	0.106	0.006	0.016
Q8BVQ5	Ppme1	0.992	(0.011)	0.569	0.625
Q8BVU5	Nudt9	1.160	0.214	0.000	0.003
Q8BW70	Usp38	0.868	(0.203)	0.009	0.022
Q8BW87	Ap1s2	1.073	0.102	0.107	0.149
Q8BWG8	Arrb1	0.939	(0.091)	0.175	0.227
Q8BWJ3	Phka2	0.871	(0.200)	0.001	0.007
Q8BWM0	Ptges2	1.121	0.164	0.008	0.019
Q8BWQ6	Vps35l	0.940	(0.090)	0.121	0.165
Q8BWR2	Pithd1	1.093	0.128	0.047	0.075
Q8BWT1	Acaa2	1.125	0.170	0.003	0.010
Q8BWW3	Pgm3	1.092	0.127	0.103	0.145
Q8BWY3	Etf1	0.896	(0.159)	0.002	0.007
Q8BWY7	Slc39a11	0.897	(0.157)	0.005	0.014
Q8BWZ3	Naa25	0.952	(0.070)	0.125	0.171
Q8BX10	Pgam5	1.111	0.152	0.002	0.008
Q8BX70	Vps13c	0.888	(0.172)	0.008	0.020
Q8BX94	Osbpl2	0.875	(0.193)	0.006	0.017
Q8BXC6	Commd2	0.926	(0.111)	0.032	0.055
Q8BXL7	Arfrp1	0.895	(0.160)	0.005	0.015
Q8BXN9	Tmem87a	0.928	(0.108)	0.045	0.073
Q8BXZ1	Tmx3	1.022	0.032	0.178	0.231
Q8BY02	Nkrf	1.177	0.235	0.000	0.003
Q8BY05	Papolg	1.002	0.003	0.927	0.940
Q8BY89	Slc44a2	1.000	0.000	0.993	0.994
Q8BYA0	Tbcd	1.000	0.000	0.995	0.996
Q8BYB9	Poglut1	0.926	(0.111)	0.051	0.080
Q8BYC6	Taok3	0.933	(0.100)	0.013	0.028
Q8BYH7	Tbc1d17	1.007	0.010	0.822	0.852

Q8BYK4	Rdh12	0.935	(0.096)	0.107	0.149
Q8BYK6	Ythdf3	0.840	(0.252)	0.002	0.009
Q8BYL4	Yars2	0.958	(0.062)	0.124	0.168
Q8BYW1	Arhgap25	1.118	0.161	0.010	0.024
Q8BYZ1	Abi3	0.952	(0.071)	0.416	0.476
Q8BZ03	Prkd2	1.011	0.016	0.557	0.613
Q8BZ20	Parp12	0.773	(0.371)	0.201	0.256
Q8BZN6	Dock10	0.945	(0.081)	0.000	0.003
Q8BZQ7	Anapc2	1.041	0.058	0.189	0.242
Q8BZR9	Ncbp3	1.181	0.240	0.033	0.058
Q8BZW8	Nhlrc2	0.907	(0.141)	0.048	0.077
Q8BZX4	Srek1	0.986	(0.020)	0.725	0.767
Q8C048	Atp2b3	0.911	(0.134)	0.108	0.150
Q8C050	Rps6ka5	0.985	(0.021)	0.368	0.429
Q8C052	Map1s	0.913	(0.131)	0.011	0.025
Q8C079	Strip1	1.007	0.010	0.747	0.786
Q8C0C0	Zhx2	1.137	0.185	0.001	0.006
Q8C0C7	Farsa	0.993	(0.010)	0.877	0.899
Q8C0D5	Efl1	0.907	(0.141)	0.001	0.005
Q8C0D7	Ing4	0.887	(0.173)	0.024	0.044
Q8C0E2	Vps26b	0.847	(0.239)	0.000	0.004
Q8C0J2	Atg16l1	0.855	(0.225)	0.002	0.009
Q8C0J6	Sowahc	0.912	(0.132)	0.002	0.009
Q8C0L0	Tmx4	0.881	(0.183)	0.014	0.030
Q8C0L6	Paox	0.942	(0.087)	0.001	0.006
Q8C0L9	Gpcpd1	1.065	0.091	0.026	0.047
Q8C0M9	Asrgl1	1.067	0.093	0.001	0.005
Q8C0S1	Dis3l	1.092	0.127	0.012	0.026
Q8C0V0	Tlk1	1.146	0.197	0.003	0.010
Q8C0W0	Tmsb15l	0.971	(0.043)	0.331	0.391
Q8C129	Lnpep	0.947	(0.078)	0.009	0.022
Q8C142	Ldlrap1	0.967	(0.049)	0.122	0.167
Q8C147	Dock8	0.937	(0.094)	0.012	0.026
Q8C156	Ncaph	1.095	0.130	0.107	0.149
Q8C163	Exog	1.017	0.024	0.645	0.695
Q8C176	Taf2	1.004	0.006	0.855	0.880
Q8C1D8	Iws1	1.130	0.176	0.018	0.035
Q8C1Y8	Ccz1	0.909	(0.138)	0.000	0.003
Q8C214	Tmem209	1.039	0.055	0.077	0.113
Q8C263	Ska3	0.933	(0.101)	0.035	0.060
Q8C2A2	Tsen54	0.934	(0.099)	0.095	0.135
Q8C2E4	Ptcd1	1.074	0.103	0.010	0.023
Q8C2E7	Washc5	0.995	(0.008)	0.806	0.839
Q8C2Q3	Rbm14	1.169	0.226	0.000	0.004
Q8C2Q8	Atp5c1	1.136	0.184	0.007	0.018
Q8C2T6	Pola2	1.087	0.121	0.010	0.022
Q8C2T8	Gabpb2	1.161	0.215	0.001	0.005
Q8C3I8	Hgh1	0.891	(0.166)	0.020	0.038
Q8C3J5	Dock2	0.989	(0.016)	0.171	0.223
Q8C3S2	Tango6	0.934	(0.099)	0.117	0.161
Q8C3V4	Stat1	1.012	0.018	0.896	0.915
Q8C3X2	Ccdc90b	1.013	0.018	0.528	0.585
Q8C3X8	Lmf2	0.834	(0.262)	0.008	0.020
Q8C3Y4	Kntc1	0.838	(0.255)	0.011	0.026
Q8C405	Sp100	1.008	0.011	0.911	0.927
Q8C4C9	Dnajb12	0.991	(0.013)	0.266	0.324
Q8C4M7	Cenpu	1.120	0.164	0.040	0.066
Q8C4Y3	Nelfb	0.976	(0.035)	0.309	0.369
Q8C522	Endod1	1.189	0.250	0.005	0.015
Q8C547	Heatr5b	0.953	(0.069)	0.005	0.014
Q8C570	Rae1	1.050	0.070	0.028	0.050

Q8C587	Alox12	1.084	0.117	0.270	0.328
Q8C5K5		0.943	(0.084)	0.045	0.074
Q8C5L6	Inpp5k	1.007	0.011	0.833	0.862
Q8C5P5	Nt5dc1	0.988	(0.018)	0.331	0.392
Q8C5Q4	Grsf1	1.074	0.103	0.139	0.186
Q8C5W3	Tbcel	1.096	0.132	0.004	0.012
Q8C605	Pfkp	0.882	(0.181)	0.036	0.062
Q8C6E0	Cfap36	0.998	(0.003)	0.938	0.948
Q8C6H0	Lgals8	0.906	(0.142)	0.004	0.011
Q8C708		0.912	(0.132)	0.186	0.239
Q8C788	Snx18	1.174	0.231	0.004	0.012
Q8C7D2	Crbn	1.001	0.001	0.971	0.975
Q8C7E9	Cstf2t	1.108	0.148	0.274	0.332
Q8C7V3	Utp15	1.089	0.123	0.136	0.183
Q8C863	Itch	0.965	(0.051)	0.033	0.057
Q8C878	Uba3	0.965	(0.052)	0.060	0.092
Q8C8I0	Plcb3	0.837	(0.256)	0.008	0.020
Q8C8I3	Eif2d	1.042	0.060	0.092	0.131
Q8C8T8	Tsr2	0.924	(0.114)	0.064	0.096
Q8C9B9	Dido1	1.048	0.068	0.254	0.311
Q8C9V1	Tbc1d10c	0.915	(0.128)	0.007	0.018
Q8CB27	Yod1	0.951	(0.072)	0.195	0.249
Q8CB62	Cntrob	0.851	(0.233)	0.002	0.009
Q8CB77	Eloa	1.036	0.052	0.078	0.115
Q8CBC4	Cnst	0.987	(0.019)	0.932	0.943
Q8CBM2	Asph	1.178	0.237	0.014	0.030
Q8CBY3	Leng8	0.971	(0.042)	0.291	0.350
Q8CBY8	Dctn4	0.972	(0.041)	0.405	0.465
Q8CC88	Vwa8	0.956	(0.064)	0.058	0.089
Q8CCF0	Prpf31	1.076	0.105	0.031	0.054
Q8CCJ9	Phf20l1	1.128	0.173	0.069	0.103
Q8CCK0	H2afy2	0.840	(0.252)	0.006	0.015
Q8CCM0	Camk2d	1.060	0.084	0.414	0.474
Q8CCP0	Nemf	0.948	(0.077)	0.089	0.127
Q8CD10	Micu2	0.945	(0.081)	0.162	0.212
Q8CD15	Riox2	1.108	0.148	0.000	0.005
Q8CD92	Ttc27	1.078	0.108	0.026	0.047
Q8CDA1	Inpp5f	0.974	(0.038)	0.451	0.511
Q8CDM8	Fam160b1	0.857	(0.223)	0.000	0.003
Q8CDN6	Txnl1	1.157	0.210	0.000	0.004
Q8CE46	Pus7l	0.894	(0.161)	0.059	0.091
Q8CE64	Znf276	0.877	(0.189)	0.001	0.005
Q8CE96	Trmt6	1.132	0.179	0.014	0.029
Q8CEC0	Nup88	1.033	0.047	0.151	0.199
Q8CEC6	Ppwd1	1.052	0.073	0.008	0.020
Q8CEG8	Usp27	0.962	(0.056)	0.001	0.006
Q8CF89	Tab1	1.037	0.052	0.331	0.392
Q8CFE2	Hpf1	1.023	0.033	0.311	0.371
Q8CFE4	Scyl2	1.028	0.040	0.090	0.129
Q8CFI7	Polr2b	1.079	0.109	0.001	0.006
Q8CFK6	Dennd1c	1.153	0.205	0.455	0.515
Q8CFT2	Setd1b	1.056	0.079	0.541	0.597
Q8CFY5	Cox10	0.891	(0.167)	0.024	0.045
Q8CG29	Myo1f	0.932	(0.102)	0.008	0.019
Q8CG46	Smc5	1.043	0.061	0.108	0.150
Q8CG48	Smc2	1.035	0.050	0.080	0.117
Q8CG50	Rab43	1.062	0.087	0.156	0.206
Q8CG72	Adprhl2	0.955	(0.066)	0.030	0.053
Q8CG76	Akr7a2	1.013	0.019	0.376	0.437
Q8CGA0	Ppm1f	1.163	0.218	0.038	0.063
Q8CGC6	Rbm28	0.998	(0.003)	0.945	0.953

Q8CGC7	Eprs	1.073	0.102	0.020	0.039
Q8CGF7	Tcerg1	1.082	0.114	0.006	0.016
Q8CGK3	Lonp1	1.055	0.077	0.009	0.021
Q8CGY8	Ogt	1.022	0.032	0.223	0.279
Q8CGZ0	Cherp	1.103	0.141	0.001	0.007
Q8CH02	Sugp1	1.006	0.009	0.699	0.744
Q8CH18	Ccar1	1.071	0.099	0.000	0.003
Q8CH25	Sltm	1.027	0.038	0.102	0.143
Q8CHC4	Synj1	0.852	(0.231)	0.001	0.006
Q8CHH5	Bicral	1.033	0.047	0.613	0.665
Q8CHI8	Ep400	1.032	0.046	0.241	0.298
Q8CHK3	Mboat7	0.907	(0.141)	0.030	0.053
Q8CHP5	Pym1	0.955	(0.066)	0.143	0.190
Q8CHP8	Pgp	0.923	(0.116)	0.036	0.061
Q8CHS8	Vps37a	0.952	(0.070)	0.208	0.263
Q8CHT0	Aldh4a1	1.178	0.236	0.003	0.011
Q8CHT3	Ints5	0.919	(0.122)	0.004	0.012
Q8CHW4	Eif2b5	0.921	(0.119)	0.104	0.146
Q8CI08	Slain2	0.895	(0.160)	0.005	0.013
Q8CI11	Gnl3	1.009	0.013	0.530	0.587
Q8CI32	Bag5	0.888	(0.171)	0.040	0.066
Q8CI33	Cwf19l1	0.940	(0.089)	0.185	0.238
Q8CI61	Bag4	0.880	(0.184)	0.019	0.037
Q8CI71	Vps50	1.006	0.009	0.873	0.896
Q8CI75	Dis3l2	1.039	0.055	0.076	0.112
Q8CI94	Pygb	1.003	0.004	0.269	0.327
Q8CI95	Osbpl11	0.935	(0.097)	0.076	0.112
Q8CIA9	Mfsd14b	0.929	(0.106)	0.472	0.531
Q8CIE4	Parp10	0.882	(0.181)	0.074	0.110
Q8CIE6	Copa	0.974	(0.039)	0.026	0.047
Q8CIH5	Plcg2	1.036	0.051	0.137	0.183
Q8CIH9	Ppat	1.024	0.035	0.467	0.526
Q8CII2	Cdc123	1.006	0.009	0.926	0.939
Q8CII5	Tia1	1.091	0.126	0.012	0.027
Q8CIM8	Ints4	1.149	0.201	0.001	0.005
Q8CIN4	Pak2	0.938	(0.092)	0.042	0.069
Q8CIS0	Card11	0.948	(0.077)	0.285	0.344
Q8CIV8	Tbce	0.902	(0.148)	0.012	0.026
Q8CJ40	Crocc	0.862	(0.215)	0.021	0.040
Q8CJF7	Ahctf1	0.942	(0.086)	0.073	0.108
Q8CJG0	Ago2	1.135	0.182	0.044	0.071
Q8HWB2	H2-Q4	0.943	(0.085)	0.420	0.479
Q8JZL3	Thtpa	0.930	(0.105)	0.095	0.135
Q8JZM7	Cdc73	1.086	0.119	0.003	0.010
Q8JZN5	Acad9	1.045	0.063	0.001	0.006
Q8JZN7	Rhot2	1.077	0.107	0.059	0.090
Q8JZQ2	Afg3l2	1.198	0.261	0.000	0.003
Q8JZQ9	Eif3b	1.069	0.096	0.006	0.015
Q8JZR0	Acsl5	0.999	(0.001)	0.953	0.960
Q8JZU2	Slc25a1	0.887	(0.172)	0.034	0.058
Q8JZV7	Amdhd2	0.876	(0.191)	0.000	0.004
Q8JZX4	Rbm17	1.039	0.056	0.103	0.145
Q8K003	Tma7	0.960	(0.059)	0.317	0.377
Q8K019	Bclaf1	1.107	0.146	0.000	0.004
Q8K039		1.066	0.092	0.000	0.003
Q8K0B2	Lmbrd1	0.926	(0.111)	0.085	0.122
Q8K0C4	Cyp51a1	1.161	0.215	0.029	0.052
Q8K0C9	Gmds	0.943	(0.085)	0.011	0.024
Q8K0D5	Gfm1	1.073	0.102	0.050	0.080
Q8K0E8	Fgb	0.895	(0.160)	0.139	0.186
Q8K0F1	Tbc1d23	0.984	(0.023)	0.533	0.590

Q8K0G5	Eipr1	0.985	(0.022)	0.216	0.272
Q8K0H5	Taf10	1.016	0.022	0.567	0.623
Q8K0Q5	Arhgap18	0.970	(0.044)	0.407	0.467
Q8K0V4	Cnot3	1.111	0.152	0.000	0.004
Q8K0Z7	Taco1	1.079	0.109	0.013	0.029
Q8K124	Plekho2	0.888	(0.171)	0.013	0.028
Q8K135	Kiaa0319l	1.024	0.034	0.571	0.626
Q8K157	Galm	0.846	(0.241)	0.004	0.012
Q8K183	Pdxk	0.978	(0.031)	0.461	0.520
Q8K1B8	Fermt3	0.900	(0.152)	0.002	0.009
Q8K1G9	Dhx35	1.025	0.036	0.468	0.527
Q8K1H1	Tdrd7	0.877	(0.190)	0.151	0.200
Q8K1I7	Wipf1	0.847	(0.239)	0.008	0.020
Q8K1J5	Sde2	0.873	(0.196)	0.028	0.050
Q8K1J6	Trnt1	0.973	(0.039)	0.072	0.106
Q8K1K4	Cenpi	0.989	(0.016)	0.738	0.779
Q8K1M3	Prkar2a	0.977	(0.034)	0.024	0.045
Q8K1R7	Nek9	0.924	(0.114)	0.003	0.010
Q8K1X4	Nckap1l	0.999	(0.002)	0.925	0.939
Q8K212	Pacs1	0.860	(0.217)	0.000	0.004
Q8K215	Lyrm4	1.126	0.171	0.017	0.034
Q8K224	Nat10	1.100	0.138	0.011	0.025
Q8K245	Uvrag	0.933	(0.100)	0.000	0.004
Q8K268	Abcf3	0.963	(0.054)	0.090	0.128
Q8K273	Mmgt1	0.930	(0.105)	0.097	0.137
Q8K297	Colgalt1	0.924	(0.115)	0.038	0.064
Q8K298	Anln	0.936	(0.096)	0.062	0.094
Q8K2C7	Os9	0.847	(0.239)	0.005	0.015
Q8K2C9	Hacd3	0.916	(0.127)	0.022	0.042
Q8K2D3	Edc3	1.183	0.242	0.007	0.017
Q8K2F0	Brd3	1.143	0.193	0.009	0.021
Q8K2F8	Lsm14a	1.118	0.160	0.004	0.012
Q8K2H4	Acap1	0.950	(0.075)	0.024	0.045
Q8K2H6	Anapc10	0.960	(0.060)	0.334	0.394
Q8K2I1	Fntb	0.976	(0.035)	0.469	0.527
Q8K2I4	Manba	0.907	(0.141)	0.001	0.006
Q8K2L8	Trappc12	1.102	0.140	0.003	0.010
Q8K2Q0	Commd9	0.936	(0.095)	0.055	0.086
Q8K2Q7	Brox	1.040	0.057	0.019	0.037
Q8K2V6	Ipo11	0.985	(0.021)	0.735	0.776
Q8K2Y9	Ccm2	1.068	0.095	0.235	0.291
Q8K301	Ddx52	1.115	0.157	0.025	0.046
Q8K310	Matr3	1.062	0.087	0.003	0.010
Q8K337	Inpp5b	0.911	(0.134)	0.001	0.005
Q8K339	Kin	1.034	0.049	0.358	0.419
Q8K352	Sash3	0.976	(0.036)	0.318	0.378
Q8K358	Pigu	1.119	0.163	0.046	0.075
Q8K363	Ddx18	1.155	0.208	0.002	0.008
Q8K368	Fanci	1.155	0.208	0.005	0.014
Q8K382	Dennd1a	1.109	0.149	0.010	0.024
Q8K389	Cdk5rap2	0.836	(0.258)	0.000	0.002
Q8K394	Plcl2	0.971	(0.042)	0.219	0.275
Q8K3A9	Mepce	0.939	(0.091)	0.018	0.036
Q8K3C3	Lzic	1.076	0.106	0.007	0.018
Q8K3D3	Swi5	1.141	0.190	0.011	0.025
Q8K3G5	Vrk3	1.073	0.101	0.052	0.082
Q8K3H0	Appl1	1.038	0.054	0.437	0.497
Q8K3J1	Ndufs8	1.135	0.183	0.007	0.017
Q8K3K7	Agpat2	0.930	(0.105)	0.043	0.071
Q8K3K8	Optn	0.896	(0.158)	0.030	0.052
Q8K3W0	Babam2	1.052	0.073	0.018	0.036

Q8K3W3	Casc3	1.107	0.146	0.052	0.081
Q8K3Z9	Pom121	0.945	(0.081)	0.052	0.081
Q8K426	Retnlg	1.012	0.018	0.906	0.923
Q8K449	Abca9	0.947	(0.078)	0.421	0.480
Q8K4B2	Irak3	0.877	(0.189)	0.003	0.009
Q8K4I3	Arhgef6	0.920	(0.120)	0.004	0.012
Q8K4M5	Commd1	0.912	(0.133)	0.032	0.056
Q8K4P0	Wdr33	1.153	0.206	0.000	0.004
Q8K4R4	Pitpnc1	0.893	(0.164)	0.002	0.008
Q8K4W8	Clcn3	1.117	0.160	0.036	0.061
Q8K4Z3	Naxe	0.974	(0.038)	0.098	0.139
Q8K4Z5	Sf3a1	1.045	0.064	0.008	0.020
Q8N7N5	Dcaf8	1.054	0.076	0.138	0.184
Q8QZS1	Hibch	0.966	(0.050)	0.107	0.148
Q8QZS3	Flcn	0.986	(0.020)	0.606	0.659
Q8QZV7	IntS13	1.092	0.127	0.000	0.004
Q8QZW8	Arhgap9	0.911	(0.134)	0.004	0.011
Q8QZX2	Haus3	1.031	0.044	0.121	0.166
Q8QZY1	Eif3l	0.988	(0.017)	0.766	0.803
Q8QZY6	Tspan14	0.882	(0.181)	0.006	0.015
Q8QZZ7	Tprkb	0.938	(0.093)	0.390	0.450
Q8R016	Blmh	0.958	(0.062)	0.000	0.004
Q8R034	Anapc13	1.035	0.049	0.361	0.422
Q8R035	Mrpl58	1.061	0.085	0.120	0.164
Q8R050	Gspt1	1.018	0.025	0.350	0.410
Q8R059	Gale	0.988	(0.018)	0.528	0.585
Q8R079	Bfar	0.960	(0.059)	0.259	0.317
Q8R086	Suox	1.106	0.145	0.027	0.049
Q8R0A0	Gtf2f2	1.032	0.046	0.465	0.524
Q8R0F6	Ilkap	1.094	0.129	0.023	0.042
Q8R0G9	Nup133	1.184	0.243	0.000	0.003
Q8R0H9	Gga1	0.940	(0.089)	0.090	0.129
Q8R0J2	Mpdu1	0.983	(0.024)	0.379	0.440
Q8R0J7	Vps37b	0.992	(0.012)	0.853	0.879
Q8R0K9	E2f4	1.004	0.006	0.871	0.894
Q8R0P4	Aamdc	0.928	(0.108)	0.027	0.049
Q8R0S1	Atf7	0.990	(0.014)	0.725	0.767
Q8R0S2	Iqsec1	1.091	0.126	0.006	0.015
Q8R0T6	Adgrg3	0.925	(0.112)	0.221	0.277
Q8R0X7	Sgpl1	1.094	0.130	0.013	0.028
Q8R105	Vps37c	1.094	0.130	0.513	0.572
Q8R123	Flad1	0.925	(0.113)	0.044	0.072
Q8R146	Apeh	0.993	(0.011)	0.804	0.837
Q8R149	Bud13	1.118	0.161	0.001	0.005
Q8R151	Znfx1	0.869	(0.203)	0.021	0.040
Q8R164	Bphl	1.088	0.121	0.018	0.036
Q8R180	Ero1a	0.840	(0.252)	0.003	0.010
Q8R1B4	Eif3c	0.916	(0.127)	0.019	0.037
Q8R1F1	Niban2	0.852	(0.232)	0.050	0.080
Q8R1G6	Pdlim2	0.952	(0.072)	0.035	0.059
Q8R1I1	Uqcr10	1.146	0.197	0.016	0.032
Q8R1K1	Ubac2	1.028	0.040	0.540	0.596
Q8R1N0	Znf830	0.981	(0.028)	0.565	0.621
Q8R1N4	Nudcd3	0.927	(0.109)	0.054	0.084
Q8R1Q8	Dync1li1	0.945	(0.082)	0.022	0.042
Q8R1Q9	Rbks	0.835	(0.260)	0.001	0.007
Q8R1V4	Tmed4	0.894	(0.162)	0.013	0.028
Q8R205	Zc3h10	1.039	0.055	0.420	0.479
Q8R238	Sdsl	0.886	(0.174)	0.006	0.015
Q8R242	Ctbs	0.966	(0.050)	0.059	0.090
Q8R2K4	Taf6l	1.114	0.156	0.014	0.030

Q8R2N2	Utp4	1.084	0.116	0.009	0.022
Q8R2P1	Klhl25	0.930	(0.105)	0.271	0.329
Q8R2Q8	Bst2	1.139	0.187	0.380	0.441
Q8R2U0	Seh1l	1.040	0.057	0.309	0.369
Q8R2U6	Nudt4	1.192	0.254	0.041	0.067
Q8R2Y8	Ptrh2	1.169	0.225	0.004	0.011
Q8R307	Vps18	0.881	(0.183)	0.003	0.010
Q8R313	Exoc6	0.946	(0.081)	0.042	0.069
Q8R317	Ubqln1	0.985	(0.022)	0.450	0.510
Q8R322	Gle1	1.042	0.059	0.016	0.033
Q8R332	Nup58	1.090	0.125	0.005	0.015
Q8R344	Ccdc12	1.074	0.103	0.267	0.326
Q8R349	Cdc16	0.978	(0.032)	0.360	0.421
Q8R361	Rab11fip5	1.059	0.083	0.097	0.137
Q8R379	Gimap7	1.230	0.298	0.115	0.158
Q8R3C0	Mcmbp	0.939	(0.091)	0.164	0.215
Q8R3C7	Ddx19b	0.936	(0.096)	0.069	0.104
Q8R3F5	Mcat	0.984	(0.023)	0.661	0.710
Q8R3K3	Ptcd2	1.086	0.119	0.031	0.054
Q8R3N1	Nop14	1.168	0.224	0.012	0.026
Q8R3N6	Thoc1	1.182	0.241	0.009	0.022
Q8R3P6	Ints14	1.094	0.130	0.015	0.031
Q8R3Q0	Saraf	0.929	(0.107)	0.370	0.430
Q8R3V2	Aimp2	1.017	0.025	0.459	0.518
Q8R3Y8	Irf2bp1	0.959	(0.060)	0.217	0.273
Q8R409	Hexim1	1.076	0.106	0.012	0.026
Q8R418	Dicer1	1.038	0.053	0.380	0.441
Q8R480	Nup85	1.045	0.063	0.018	0.036
Q8R4R6	Nup35	1.062	0.086	0.038	0.063
Q8R4V1	Nfam1	0.897	(0.157)	0.007	0.018
Q8R4X3	Rbm12	1.152	0.204	0.000	0.003
Q8R550	Sh3kbp1	0.968	(0.047)	0.061	0.094
Q8R5A3	Apbb1ip	1.083	0.115	0.010	0.023
Q8R5A6	Tbc1d22a	0.864	(0.211)	0.000	0.004
Q8R5F3	Oard1	1.067	0.093	0.012	0.026
Q8R5F7	Ifih1	0.844	(0.244)	0.309	0.369
Q8R5G7	Arap3	0.908	(0.139)	0.013	0.029
Q8R5H1	Usp15	1.084	0.117	0.009	0.021
Q8R5I6	Gstm4	0.881	(0.183)	0.039	0.065
Q8R5J9	Arl6ip5	1.016	0.023	0.642	0.692
Q8R5K4	Nol6	1.131	0.177	0.014	0.029
Q8R5L3	Vps39	1.049	0.068	0.110	0.153
Q8VBT1	Txlnb	0.847	(0.240)	0.356	0.417
Q8VBV3	Exosc2	1.083	0.115	0.008	0.019
Q8VBV7	Cops8	0.940	(0.090)	0.025	0.045
Q8VC16	Lrrc14	0.960	(0.059)	0.575	0.630
Q8VC28	Akr1c13	1.106	0.146	0.116	0.159
Q8VC31	Ccdc9	0.916	(0.127)	0.019	0.037
Q8VC42	Rmc1	0.852	(0.231)	0.001	0.007
Q8VC60	Glb1l	0.914	(0.130)	0.087	0.125
Q8VC65	Nrm	0.927	(0.109)	0.015	0.031
Q8VCA8	Scrn2	0.974	(0.037)	0.483	0.542
Q8VCB1	Ndc1	0.952	(0.071)	0.013	0.029
Q8VCD5	Med17	1.036	0.051	0.062	0.094
Q8VCF1	Cant1	0.908	(0.139)	0.030	0.053
Q8VCI5	Pex19	0.930	(0.104)	0.033	0.057
Q8VCM7	Fgg	0.874	(0.194)	0.212	0.267
Q8VCM8	Ncln	0.958	(0.061)	0.341	0.401
Q8VCN6	Cd99	0.943	(0.084)	0.206	0.261
Q8VCQ8	Cald1	0.968	(0.047)	0.722	0.765
Q8VCR0	Rxrb	1.069	0.096	0.015	0.032

Q8VCR4	Trmt112	1.055	0.078	0.032	0.056
Q8VCS3	Fam20b	0.966	(0.050)	0.567	0.623
Q8VCT3	Rnpep	0.875	(0.192)	0.018	0.035
Q8VCW4	Unc93b1	0.990	(0.015)	0.810	0.842
Q8VCX5	Micu1	0.900	(0.152)	0.006	0.016
Q8VCY6	Utp6	0.934	(0.099)	0.134	0.181
Q8VD04	Gripap1	0.923	(0.116)	0.001	0.005
Q8VD31	Tapbpl	1.161	0.215	0.142	0.189
Q8VD58	Evi2b	1.045	0.063	0.286	0.345
Q8VD65	Pik3r4	0.959	(0.061)	0.481	0.540
Q8VDD5	Myh9	0.885	(0.176)	0.001	0.006
Q8VDF3	Dapk2	1.117	0.160	0.119	0.163
Q8VDG3	Parn	1.057	0.080	0.022	0.042
Q8VDG5	Ppcs	1.158	0.211	0.010	0.022
Q8VDJ3	Hdlbp	0.889	(0.169)	0.053	0.084
Q8VDK1	Nit1	0.952	(0.071)	0.158	0.207
Q8VDM1	Zgpat	0.957	(0.064)	0.042	0.069
Q8VDM4	Psmd2	0.978	(0.032)	0.600	0.654
Q8VDM6	Hnrnpul1	1.072	0.100	0.031	0.054
Q8VDN2	Atp1a1	1.045	0.063	0.014	0.029
Q8VDP3	Mical1	0.926	(0.111)	0.001	0.005
Q8VDP4	Ccar2	1.039	0.055	0.078	0.115
Q8VDQ1	Ptgr2	1.118	0.160	0.068	0.102
Q8VDS4	Rprd1a	0.857	(0.222)	0.002	0.009
Q8VDT9	Mrpl50	1.000	(0.000)	1.000	1.000
Q8VDZ4	Zdhhc5	0.926	(0.111)	0.057	0.088
Q8VE10	Naa40	1.122	0.167	0.008	0.019
Q8VE11	Mtmr6	0.867	(0.205)	0.001	0.006
Q8VE38	Oxnad1	1.081	0.113	0.037	0.062
Q8VE47	Uba5	0.843	(0.247)	0.000	0.004
Q8VE52	Ogfrl1	0.877	(0.190)	0.003	0.010
Q8VE62	Paip1	0.921	(0.119)	0.022	0.042
Q8VE65	Taf12	1.015	0.022	0.502	0.561
Q8VE70	Pdcd10	0.842	(0.248)	0.004	0.013
Q8VE80	Thoc3	1.035	0.049	0.127	0.172
Q8VE88	Fam114a2	1.019	0.026	0.244	0.301
Q8VE99	Ccdc115	1.148	0.199	0.036	0.061
Q8VEB4	Pla2g15	0.984	(0.023)	0.480	0.540
Q8VED2	Bloc1s4	0.941	(0.088)	0.005	0.014
Q8VEG6	Cnot6l	0.851	(0.233)	0.018	0.035
Q8VEH3	Arl8a	0.998	(0.002)	0.959	0.965
Q8VEH5	Epm2aip1	1.125	0.171	0.015	0.031
Q8VEH8	Erlec1	0.866	(0.208)	0.001	0.007
Q8VEJ4	Nle1	0.961	(0.058)	0.251	0.308
Q8VEJ9	Vps4a	0.987	(0.019)	0.559	0.615
Q8VEK0	Tmem30a	0.932	(0.101)	0.015	0.031
Q8VHE0	Sec63	0.968	(0.047)	0.195	0.249
Q8VHK9	Dhx36	1.038	0.054	0.486	0.544
Q8VHL1	Setd7	0.913	(0.131)	0.051	0.080
Q8VHN8	Nudt16l1	0.947	(0.079)	0.022	0.042
Q8VHR5	Gatad2b	1.004	0.006	0.632	0.682
Q8VHX6	Flnc	0.881	(0.183)	0.025	0.045
Q8VI36	Pxn	0.858	(0.220)	0.049	0.078
Q8VI63	Mob2	1.101	0.139	0.033	0.057
Q8VI75	Ipo4	0.975	(0.036)	0.381	0.441
Q8VI84	Noc3l	0.904	(0.146)	0.039	0.065
Q8VI93	Oas3	0.754	(0.407)	0.138	0.184
Q8VIG1	Rest	0.943	(0.084)	0.253	0.310
Q8VIJ6	Sfpq	1.102	0.140	0.000	0.004
Q8VIM9	Irgq	0.984	(0.023)	0.376	0.437
Q91UZ5	Impa2	0.889	(0.169)	0.006	0.017

Q91V01	Lpcat3	1.015	0.022	0.400	0.460
Q91V04	Tram1	0.886	(0.175)	0.035	0.060
Q91V12	Acot7	0.886	(0.174)	0.003	0.011
Q91V16	Etfrf1	0.958	(0.062)	0.151	0.200
Q91V24	Abca7	0.859	(0.219)	0.000	0.005
Q91V41	Rab14	0.973	(0.039)	0.046	0.074
Q91V55	Rps5	0.930	(0.105)	0.128	0.174
Q91V76		1.071	0.099	0.009	0.022
Q91V81	Rbm42	1.011	0.016	0.518	0.575
Q91VC9	Ghitm	1.104	0.143	0.005	0.014
Q91VD9	Ndufs1	1.180	0.238	0.004	0.013
Q91VE0	Slc27a4	1.043	0.061	0.084	0.121
Q91VF2	Hnmt	1.036	0.051	0.448	0.508
Q91VH2	Snx9	1.040	0.056	0.026	0.047
Q91VJ1	Aim2	0.971	(0.042)	0.071	0.105
Q91VJ4	Stk38	0.975	(0.037)	0.149	0.197
Q91VJ5	Pqbp1	1.034	0.048	0.130	0.176
Q91VK1	Bzw2	0.946	(0.081)	0.300	0.360
Q91VL0	Bnip2	0.936	(0.095)	0.157	0.207
Q91VM9	Ppa2	1.101	0.139	0.035	0.059
Q91VN6	Ddx41	0.853	(0.230)	0.003	0.010
Q91VR5	Ddx1	1.099	0.136	0.032	0.055
Q91VR8	Brk1	0.928	(0.108)	0.036	0.061
Q91VS7	Mgst1	0.981	(0.028)	0.621	0.673
Q91VT1	Nsmce2	0.955	(0.066)	0.232	0.288
Q91VU7	Pus7	1.057	0.080	0.115	0.159
Q91VW3	Sh3bgrl3	0.909	(0.138)	0.021	0.040
Q91VY6	Cytip	0.912	(0.132)	0.005	0.014
Q91VY9	Znf622	1.017	0.025	0.653	0.703
Q91VZ6	Smap1	0.982	(0.027)	0.334	0.395
Q91W34		1.123	0.167	0.003	0.010
Q91W39	Ncoa5	1.142	0.192	0.004	0.012
Q91W50	Csde1	0.985	(0.022)	0.603	0.656
Q91W67	Ubl7	0.834	(0.262)	0.015	0.031
Q91W86	Vps11	0.914	(0.129)	0.019	0.038
Q91W96	Anapc4	0.933	(0.100)	0.184	0.237
Q91W98	Slc15a4	1.001	0.002	0.961	0.966
Q91WA1	Tipin	1.035	0.050	0.045	0.073
Q91WA6	Sharpin	0.997	(0.004)	0.939	0.948
Q91WB4	Plekhf2	0.954	(0.068)	0.183	0.236
Q91WC0	Setd3	0.923	(0.115)	0.000	0.005
Q91WD5	Ndufs2	1.140	0.188	0.015	0.032
Q91WE4		1.084	0.116	0.050	0.079
Q91WG2	Rabep2	1.059	0.083	0.003	0.010
Q91WG3	Trub2	1.107	0.147	0.042	0.069
Q91WG4	Elp2	1.083	0.115	0.009	0.022
Q91WK1	Spryd4	1.164	0.220	0.003	0.010
Q91WK2	Eif3h	0.995	(0.007)	0.897	0.916
Q91WL8	Wwox	0.873	(0.197)	0.003	0.011
Q91WM2	Hdhd5	1.113	0.154	0.138	0.184
Q91WM3	Rrp9	0.998	(0.003)	0.956	0.962
Q91WQ5	Taf5l	1.131	0.177	0.007	0.017
Q91WS0	Cisd1	1.217	0.284	0.067	0.100
Q91WT4	Dnajc17	1.015	0.022	0.221	0.276
Q91WU5	As3mt	1.030	0.043	0.286	0.345
Q91WV0	Dr1	0.896	(0.159)	0.049	0.079
Q91X21	Kiaa2013	0.833	(0.263)	0.001	0.006
Q91X76	Nt5dc2	1.079	0.109	0.063	0.095
Q91X78	Erlin1	0.999	(0.001)	0.981	0.984
Q91X84	Crtc3	0.896	(0.158)	0.036	0.061
Q91X96	Rabif	0.932	(0.101)	0.136	0.182

Q91XA2	Golm1	0.944	(0.083)	0.027	0.049
Q91XB0	Trex1	0.917	(0.125)	0.528	0.585
Q91XD6	Vps36	0.942	(0.086)	0.008	0.020
Q91XH6	Vti1b	0.861	(0.216)	0.026	0.047
Q91XU0	Wrnip1	1.019	0.027	0.483	0.541
Q91XU3	Pip4k2c	1.022	0.032	0.239	0.295
Q91XV3	Basp1	0.930	(0.105)	0.430	0.490
Q91YD3	Dcp1a	1.000	0.001	0.978	0.981
Q91YE3	Egln1	0.855	(0.227)	0.000	0.004
Q91YE7	Rbm5	0.903	(0.148)	0.032	0.056
Q91YI0	Asl	1.028	0.040	0.169	0.220
Q91YJ2	Snx4	1.125	0.170	0.015	0.031
Q91YK2	Rrp1b	0.948	(0.077)	0.045	0.073
Q91YL2	Rnf126	0.868	(0.204)	0.003	0.010
Q91YM4	Tbrg4	1.070	0.098	0.035	0.060
Q91YP0	L2hgdh	1.126	0.171	0.076	0.112
Q91YP2	Nln	1.002	0.002	0.953	0.960
Q91YP3	Dera	1.149	0.201	0.003	0.010
Q91YQ5	Rpn1	1.064	0.089	0.019	0.036
Q91YR1	Twf1	1.167	0.223	0.005	0.014
Q91YR5	EEF1AKNMT	0.958	(0.062)	0.064	0.096
Q91YR7	Prpf6	1.065	0.091	0.001	0.007
Q91YS7	Map2k2	0.847	(0.239)	0.000	0.004
Q91YT7	Ythdf2	0.945	(0.081)	0.133	0.179
Q91YT8	Tmem63a	1.020	0.029	0.847	0.874
Q91YU8	Ppan	1.084	0.116	0.014	0.030
Q91YW3	Dnajc3	0.839	(0.253)	0.001	0.006
Q91YX0	Themis2	0.962	(0.056)	0.382	0.443
Q91YX5	Lpgat1	1.177	0.235	0.037	0.062
Q91YZ2	Ctbp2	0.846	(0.241)	0.000	0.003
Q91Z38	Ttc1	0.941	(0.088)	0.127	0.173
Q91Z40	Gbp7	0.933	(0.101)	0.351	0.412
Q91Z50	Fen1	1.189	0.250	0.001	0.005
Q91Z67	Srgap2	0.931	(0.103)	0.002	0.009
Q91Z83	Myh7	1.117	0.159	0.846	0.873
Q91Z96	Bmp2k	0.959	(0.060)	0.259	0.317
Q91ZJ5	Ugp2	0.968	(0.047)	0.279	0.338
Q91ZM2	Sh2b1	0.996	(0.006)	0.903	0.921
Q91ZR1	Rab4b	0.914	(0.129)	0.023	0.042
Q91ZW3	Smarca5	1.089	0.123	0.000	0.004
Q920A5	Scpep1	1.088	0.122	0.239	0.295
Q920A7	Afg3l1	1.062	0.087	0.292	0.352
Q920D3	Med28	0.946	(0.079)	0.079	0.115
Q920I9	Wdr7	0.885	(0.176)	0.005	0.013
Q921C5	Bicd2	0.941	(0.088)	0.042	0.069
Q921E2	Rab31	0.896	(0.159)	0.002	0.008
Q921E6	Eed	1.181	0.240	0.003	0.010
Q921F2	Tardbp	1.088	0.122	0.013	0.029
Q921F4	Hnrnpll	0.924	(0.115)	0.016	0.033
Q921G7	Etfdh	1.111	0.152	0.013	0.029
Q921I9	Exosc4	0.951	(0.073)	0.335	0.395
Q921L3	Tmco1	0.851	(0.232)	0.017	0.034
Q921L5	Cog2	0.891	(0.167)	0.010	0.023
Q921M3	Sf3b3	1.080	0.111	0.005	0.015
Q921M7	Fam49b	0.897	(0.156)	0.016	0.033
Q921N8	Gps2	0.931	(0.103)	0.003	0.010
Q921S7	Mrpl37	1.157	0.211	0.001	0.006
Q921T2	Tor1aip1	1.000	0.000	0.998	0.999
Q921Z5	Tnfaip8	0.938	(0.092)	0.196	0.250
Q922B1	Macrod1	1.138	0.186	0.043	0.070
Q922B2	Dars	0.973	(0.039)	0.227	0.283

Q922D4	Ppp6r3	0.888	(0.171)	0.000	0.004
Q922E6	Fastkd2	0.986	(0.020)	0.606	0.659
Q922G2	Fam76a	0.974	(0.039)	0.226	0.282
Q922H1	Prmt3	1.086	0.119	0.111	0.153
Q922H4	Gmppa	0.908	(0.139)	0.195	0.248
Q922J3	Clip1	0.851	(0.233)	0.000	0.003
Q922J9	Far1	0.846	(0.241)	0.059	0.091
Q922L6	Nelfcd	0.994	(0.008)	0.900	0.918
Q922M3	Kctd10	0.879	(0.186)	0.001	0.006
Q922M5	Cdca7l	1.004	0.005	0.894	0.914
Q922P9	Glyr1	1.150	0.202	0.001	0.006
Q922Q4	Pycr2	1.007	0.010	0.806	0.839
Q922Q8	Lrrc59	0.856	(0.224)	0.050	0.079
Q922S8	Kif2c	0.987	(0.019)	0.628	0.678
Q922V4	Plrg1	1.074	0.103	0.113	0.156
Q922X9	Prmt7	1.155	0.207	0.050	0.079
Q923B1	Dbr1	0.989	(0.017)	0.487	0.545
Q923D4	Sf3b5	1.017	0.025	0.567	0.623
Q923D5	Wbp11	1.094	0.129	0.001	0.007
Q923E4	Sirt1	0.983	(0.025)	0.731	0.773
Q923F1	Clns1a	1.178	0.236	0.120	0.165
Q923T9	Camk2g	0.991	(0.013)	0.784	0.819
Q923W1	Tgs1	0.917	(0.125)	0.182	0.234
Q923X4	Glrx2	0.924	(0.114)	0.287	0.346
Q924B0	Impa1	0.930	(0.105)	0.029	0.051
Q924C1	Xpo5	1.080	0.111	0.000	0.004
Q924L1	Letmd1	1.095	0.131	0.320	0.380
Q924M7	Mpi	0.860	(0.218)	0.012	0.027
Q924T2	Mrps2	1.006	0.009	0.904	0.921
Q924T7	Rnf31	1.074	0.104	0.092	0.131
Q924W5	Smc6	1.116	0.158	0.002	0.009
Q924Z6	Xpo6	0.935	(0.097)	0.015	0.031
Q925E7	Ppp2r2d	0.990	(0.015)	0.487	0.545
Q925I1	Atad3	1.095	0.131	0.002	0.008
Q925N2	Sfxn2	0.976	(0.036)	0.628	0.678
Q93092	Taldo1	0.933	(0.099)	0.098	0.138
Q99388	Csprs	0.920	(0.120)	0.319	0.380
Q99J09	Wdr77	0.956	(0.065)	0.061	0.093
Q99J25	Mrm1	0.918	(0.124)	0.123	0.167
Q99J45	Nrbp1	0.843	(0.247)	0.003	0.011
Q99J64	Rbm43	0.909	(0.138)	0.002	0.008
Q99J77	Nans	0.976	(0.035)	0.194	0.248
Q99J87	Dhx58	0.904	(0.146)	0.621	0.672
Q99JB2	Stoml2	1.002	0.003	0.945	0.953
Q99JH1	Rpp25l	1.062	0.087	0.224	0.280
Q99JI4	Psmd6	0.975	(0.036)	0.380	0.441
Q99JI6	Rap1b	0.940	(0.090)	0.037	0.062
Q99JP4	Cdc26	1.011	0.016	0.749	0.787
Q99JR5	Tinagl1	0.920	(0.121)	0.253	0.310
Q99JR8	Smarcd2	1.029	0.041	0.136	0.183
Q99JT1	Gatb	1.045	0.064	0.096	0.136
Q99JT2	Stk26	1.110	0.151	0.050	0.079
Q99JW4	Lims1	0.928	(0.107)	0.177	0.229
Q99JX1	Taf11	1.031	0.043	0.089	0.128
Q99JX4	Eif3m	0.975	(0.036)	0.154	0.202
Q99JX7	Nxf1	1.128	0.174	0.000	0.003
Q99JY3	Gimap4	1.227	0.295	0.066	0.100
Q99JY9	Actr3	0.895	(0.160)	0.000	0.005
Q99JZ4	Sar1a	0.890	(0.168)	0.049	0.077
Q99K23	Ufsp2	0.998	(0.003)	0.872	0.895
Q99K28	Arfgap2	0.920	(0.121)	0.016	0.032

Q99K48	Nono	1.111	0.152	0.001	0.006
Q99K70	Rragc	1.151	0.203	0.002	0.008
Q99K74	Med24	0.885	(0.176)	0.008	0.020
Q99K85	Psat1	1.127	0.173	0.004	0.013
Q99K95	Rtf2	0.944	(0.082)	0.075	0.110
Q99KC8	Vwa5a	0.849	(0.236)	0.001	0.005
Q99KD5	Unc45a	0.919	(0.122)	0.001	0.007
Q99KE1	Me2	0.990	(0.014)	0.806	0.839
Q99KF1	Tmed9	1.026	0.036	0.088	0.126
Q99KG3	Rbm10	1.016	0.023	0.270	0.328
Q99KH8	Stk24	0.965	(0.051)	0.576	0.631
Q99KI0	Aco2	1.136	0.184	0.004	0.013
Q99KI3	Emc3	0.987	(0.019)	0.690	0.735
Q99KJ8	Dctn2	1.070	0.098	0.054	0.085
Q99KK7	Dpp3	1.086	0.118	0.004	0.012
Q99KN1	Arrdc1	0.860	(0.217)	0.004	0.012
Q99KP3	Cryl1	1.101	0.138	0.018	0.036
Q99KP6	Prpf19	1.053	0.075	0.037	0.062
Q99KQ4	Nampt	1.074	0.103	0.093	0.132
Q99KR7	Ppif	1.018	0.026	0.747	0.786
Q99KW9	Itfg1	1.132	0.179	0.070	0.104
Q99L02	Pagr1a	0.965	(0.052)	0.055	0.086
Q99L04	Dhrs1	0.849	(0.235)	0.004	0.012
Q99L13	Hibadh	1.056	0.078	0.027	0.049
Q99L27	Gmpr2	0.966	(0.049)	0.082	0.119
Q99L43	Cds2	0.865	(0.209)	0.000	0.003
Q99L45	Eif2s2	0.929	(0.106)	0.066	0.100
Q99LB0	Dnttip1	0.896	(0.159)	0.017	0.035
Q99LB2	Dhrs4	1.162	0.216	0.003	0.010
Q99LB4	Capg	1.101	0.139	0.007	0.017
Q99LB6	Mat2b	0.927	(0.110)	0.084	0.122
Q99LC2	Cstf1	1.131	0.178	0.027	0.049
Q99LC8	Eif2b1	1.080	0.111	0.032	0.055
Q99LD4	Gps1	0.925	(0.112)	0.002	0.008
Q99LD7	Mkrn1	0.956	(0.064)	0.151	0.200
Q99LD9	Eif2b2	0.943	(0.085)	0.323	0.384
Q99LE6	Abcf2	0.907	(0.141)	0.072	0.106
Q99LF4	Rtcb	1.011	0.015	0.687	0.733
Q99LG2	Tnpo2	1.003	0.004	0.907	0.924
Q99LG4	Ttc5	0.976	(0.035)	0.173	0.225
Q99LH1	Gnl2	1.133	0.180	0.003	0.011
Q99LI5	Znf281	1.040	0.057	0.082	0.119
Q99LI9	Clp1	1.088	0.122	0.028	0.050
Q99LJ1	Fuca1	0.971	(0.043)	0.124	0.169
Q99LL3	Chst12	0.841	(0.249)	0.001	0.006
Q99LL5	Pwp1	0.907	(0.141)	0.002	0.009
Q99LM2	Cdk5rap3	0.846	(0.241)	0.004	0.012
Q99LN9	Dohh	0.849	(0.237)	0.059	0.091
Q99LP6	Grpel1	1.026	0.037	0.526	0.583
Q99LT0	Dpy30	0.994	(0.008)	0.916	0.931
Q99LU0	Chmp1b1	1.154	0.206	0.007	0.017
Q99LX0	Park7	0.977	(0.033)	0.230	0.286
Q99LX5	Mmtag2	0.987	(0.019)	0.569	0.625
Q99LZ3	Gins4	1.032	0.045	0.348	0.409
Q99M31	Hspa14	0.989	(0.017)	0.836	0.864
Q99M51	Nck1	0.982	(0.027)	0.482	0.541
Q99M54	Cdca3	0.990	(0.015)	0.425	0.485
Q99M87	Dnaja3	1.057	0.080	0.016	0.033
Q99MD9	Nasp	1.126	0.171	0.004	0.011
Q99ME2	Wdr6	1.043	0.061	0.036	0.061
Q99ME9	Gtpbp4	1.090	0.124	0.006	0.016

Q99MI6	Gimap3	1.015	0.022	0.789	0.824
Q99MJ9	Ddx50	1.066	0.092	0.053	0.083
Q99MK8	Grk2	0.981	(0.028)	0.296	0.356
Q99MN1	Kars	1.064	0.090	0.062	0.094
Q99MP8	Brap	0.908	(0.140)	0.020	0.039
Q99MR1	Gigyf1	0.977	(0.034)	0.328	0.388
Q99MR3	Slc12a9	0.893	(0.164)	0.022	0.042
Q99MR6	Srrt	1.106	0.145	0.001	0.005
Q99MR8	Mccc1	1.023	0.033	0.541	0.597
Q99MU3	Adar	1.117	0.160	0.286	0.345
Q99MZ7	Pecr	0.860	(0.217)	0.045	0.073
Q99N20	Brms1	0.845	(0.243)	0.005	0.014
Q99N87	Mrps5	1.042	0.060	0.315	0.375
Q99N94	Mrpl9	1.084	0.116	0.006	0.016
Q99NB8	Ubqln4	0.927	(0.109)	0.001	0.006
Q99NB9	Sf3b1	1.076	0.106	0.001	0.006
Q99NG0	Rad54l2	1.153	0.206	0.032	0.055
Q99P31	Hspbp1	1.042	0.060	0.226	0.282
Q99P58	Rab27b	0.911	(0.134)	0.386	0.446
Q99P69	Nuf2	0.983	(0.025)	0.579	0.635
Q99P72	Rtn4	0.973	(0.039)	0.105	0.147
Q99PG2	Ogfr	0.914	(0.129)	0.135	0.182
Q99PG4	Rgs18	0.850	(0.235)	0.002	0.008
Q99PM3	Gtf2a1	1.080	0.111	0.081	0.117
Q99PT1	Arhgdia	0.927	(0.109)	0.001	0.007
Q99PU7	Bap1	1.043	0.061	0.274	0.332
Q99PV0	Prpf8	1.115	0.157	0.000	0.003
Q9CPP0	Npm3	1.103	0.141	0.136	0.183
Q9CPQ1	Cox6c	1.095	0.131	0.266	0.325
Q9CPR4	Rpl17	1.065	0.091	0.203	0.257
Q9CPR5	Mrpl15	1.032	0.046	0.367	0.428
Q9CPR8	Nsmce3	1.118	0.161	0.000	0.003
Q9CPS6	Hint3	0.994	(0.008)	0.654	0.703
Q9CPS7	Pno1	1.009	0.012	0.787	0.822
Q9CPT4	Mydgf	0.928	(0.107)	0.013	0.029
Q9CPT5	Nop16	1.115	0.157	0.001	0.005
Q9CPU0	Glo1	1.025	0.036	0.331	0.391
Q9CPV4	Glod4	1.039	0.056	0.291	0.350
Q9CPV5	Pmf1	1.016	0.022	0.042	0.070
Q9CPW4	Arpc5	0.948	(0.078)	0.304	0.364
Q9CPW7	Zmat2	1.031	0.044	0.028	0.050
Q9CPX6	Atg3	0.837	(0.257)	0.000	0.004
Q9CPX7	Mrps16	1.186	0.246	0.008	0.019
Q9CPY7	Lap3	1.104	0.143	0.033	0.057
Q9CQ06	Mrpl24	0.941	(0.088)	0.032	0.055
Q9CQ10	Chmp3	1.054	0.075	0.239	0.295
Q9CQ19	Myl9	1.066	0.092	0.244	0.301
Q9CQ22	Lamtor1	1.114	0.156	0.005	0.015
Q9CQ25	Mzt2	1.127	0.173	0.021	0.040
Q9CQ40	Mrpl49	1.174	0.232	0.000	0.004
Q9CQ48	Nudcd2	1.080	0.111	0.022	0.042
Q9CQ49	Ncbp2	1.110	0.151	0.003	0.011
Q9CQ54	Ndufc2	1.099	0.136	0.016	0.032
Q9CQ60	Pgls	1.199	0.262	0.004	0.011
Q9CQ62	Decr1	1.040	0.056	0.086	0.124
Q9CQ65	Mtap	1.118	0.161	0.001	0.006
Q9CQ69	Uqcrq	1.177	0.235	0.017	0.034
Q9CQ75	Ndufa2	1.141	0.190	0.008	0.019
Q9CQ80	Vps25	0.935	(0.098)	0.035	0.060
Q9CQ86	Mien1	0.890	(0.169)	0.034	0.058
Q9CQ92	Fis1	0.948	(0.077)	0.039	0.065

Q9CQA1	Trappc5	0.890	(0.167)	0.110	0.152
Q9CQA3	Sdhb	1.151	0.202	0.008	0.019
Q9CQA5	Med4	0.958	(0.063)	0.029	0.052
Q9CQB5	Cisd2	1.028	0.039	0.409	0.470
Q9CQC6	Bzw1	0.971	(0.042)	0.258	0.316
Q9CQC8	Spg21	1.083	0.115	0.034	0.059
Q9CQC9	Sar1b	0.924	(0.114)	0.001	0.005
Q9CQE1	Nipsnap3b	1.026	0.037	0.176	0.228
Q9CQE5	Rgs10	1.029	0.042	0.351	0.412
Q9CQE8	RTRAF	1.076	0.105	0.004	0.012
Q9CQF7	Pfdn1	0.906	(0.142)	0.058	0.089
Q9CQF8	Mrpl57	1.173	0.230	0.001	0.007
Q9CQF9	Pcyox1	0.953	(0.069)	0.095	0.134
Q9CQH3	Ndufb5	1.157	0.211	0.007	0.017
Q9CQH7	Btf3l4	0.991	(0.013)	0.908	0.924
Q9CQI6	Cotl1	0.901	(0.151)	0.001	0.005
Q9CQI7	Snrpb2	1.108	0.148	0.005	0.014
Q9CQJ8	Ndufb9	0.960	(0.059)	0.272	0.330
Q9CQK7	Rwdd1	0.877	(0.190)	0.045	0.073
Q9CQL5	Mrpl18	1.124	0.169	0.009	0.022
Q9CQM5	Txndc17	0.910	(0.136)	0.015	0.031
Q9CQM8	Rpl21	0.878	(0.188)	0.163	0.213
Q9CQM9	Glrx3	0.923	(0.116)	0.085	0.123
Q9CQN1	Trap1	1.117	0.160	0.022	0.041
Q9CQQ4	Gemin2	1.128	0.174	0.149	0.198
Q9CQQ7	Atp5pb	1.077	0.107	0.105	0.147
Q9CQQ8	Lsm7	0.982	(0.025)	0.347	0.407
Q9CQR2	Rps21	0.945	(0.081)	0.186	0.239
Q9CQR6	Ppp6c	0.910	(0.136)	0.004	0.012
Q9CQS4	Slc25a46	1.018	0.026	0.459	0.518
Q9CQT1	Mri1	1.077	0.107	0.017	0.034
Q9CQU0	Txndc12	0.994	(0.009)	0.744	0.784
Q9CQU3	Rer1	1.068	0.096	0.017	0.034
Q9CQU5	Zwint	1.123	0.167	0.040	0.066
Q9CQV4	Retreg3	1.072	0.100	0.060	0.092
Q9CQV5	Mrps24	1.025	0.036	0.297	0.357
Q9CQW1	Ykt6	0.956	(0.065)	0.051	0.080
Q9CQW2	Arl8b	0.962	(0.056)	0.026	0.047
Q9CQW9	Ifitm3	0.788	(0.343)	0.175	0.227
Q9CQX6	Gm16286	0.867	(0.206)	0.037	0.063
Q9CQZ0	Ormdl2	1.075	0.105	0.008	0.020
Q9CQZ1	Hsbp1	0.937	(0.094)	0.434	0.494
Q9CQZ6	Ndufb3	1.051	0.072	0.148	0.197
Q9CR00	Psmd9	0.876	(0.190)	0.003	0.010
Q9CR02	Tma16	0.979	(0.030)	0.633	0.683
Q9CR08	Pop4	0.973	(0.039)	0.683	0.730
Q9CR11	Yeats4	1.043	0.060	0.047	0.076
Q9CR16	Ppid	0.851	(0.233)	0.005	0.015
Q9CR41	Hypk	0.852	(0.231)	0.042	0.070
Q9CR48	Dram2	0.885	(0.176)	0.013	0.028
Q9CR50	Rchy1	0.949	(0.075)	0.073	0.108
Q9CR51	Atp6v1g1	1.039	0.055	0.444	0.504
Q9CR57	Rpl14	0.879	(0.186)	0.029	0.051
Q9CR59	Gadd45gip1	1.045	0.063	0.158	0.208
Q9CR61	Ndufb7	1.053	0.075	0.330	0.391
Q9CR62	Slc25a11	0.946	(0.081)	0.085	0.122
Q9CR68	Uqcrfs1	1.065	0.091	0.016	0.033
Q9CR80	Fam32a	1.075	0.104	0.189	0.242
Q9CR86	Carhsp1	1.234	0.303	0.076	0.112
Q9CR95	Necap1	0.918	(0.123)	0.156	0.206
Q9CR98	Fam136a	0.991	(0.013)	0.513	0.571

Q9CRA5	Golph3	0.971	(0.043)	0.512	0.570
Q9CRA8	Exosc5	1.122	0.166	0.002	0.009
Q9CRA9	Fgfr1op2	1.042	0.060	0.307	0.367
Q9CRB2	Nhp2	1.138	0.187	0.023	0.044
Q9CRC6	Borcs7	0.892	(0.165)	0.009	0.021
Q9CRD2	Emc2	1.195	0.257	0.023	0.043
Q9CRG1	Tm7sf3	1.209	0.273	0.070	0.105
Q9CRT8	Xpot	0.901	(0.151)	0.018	0.036
Q9CS00	Cactin	1.159	0.213	0.000	0.004
Q9CSH3	Dis3	1.118	0.161	0.000	0.003
Q9CU62	Smc1a	1.103	0.142	0.005	0.015
Q9CVD2	Atxn3	0.953	(0.070)	0.137	0.184
Q9CW03	Smc3	1.173	0.230	0.000	0.003
Q9CW46	Raver1	1.149	0.201	0.005	0.014
Q9CWD7	Wdyhv1	0.948	(0.077)	0.001	0.006
Q9CWE0	Mtfr1l	0.992	(0.011)	0.696	0.741
Q9CWG1	Glipr1	0.841	(0.249)	0.001	0.006
Q9CWG8	Ndufaf7	1.104	0.143	0.007	0.017
Q9CWI3	Bccip	0.918	(0.123)	0.039	0.065
Q9CWJ9	Atic	1.028	0.040	0.087	0.125
Q9CWK3	Cd2bp2	1.072	0.101	0.033	0.057
Q9CWL8	Ctnnbl1	1.139	0.188	0.000	0.004
Q9CWN7	Cnot11	1.020	0.029	0.568	0.624
Q9CWQ0	Dph5	0.974	(0.037)	0.437	0.497
Q9CWU9	Nup37	1.015	0.021	0.365	0.426
Q9CWV6	Prkrip1	0.986	(0.021)	0.756	0.794
Q9CWW6	Pin4	0.900	(0.153)	0.006	0.016
Q9CWW7	Cxxc1	1.022	0.031	0.250	0.307
Q9CWY8	Rnaseh2a	1.069	0.096	0.234	0.290
Q9CWZ3	Rbm8a	1.023	0.033	0.332	0.392
Q9CWZ7	Napg	0.977	(0.034)	0.014	0.029
Q9CX00	Ist1	0.963	(0.054)	0.170	0.222
Q9CX33	Tsfm	1.028	0.040	0.302	0.362
Q9CX34	Sugt1	0.889	(0.170)	0.020	0.038
Q9CX53	Gemin6	0.957	(0.063)	0.108	0.150
Q9CX97	Wdr55	0.984	(0.023)	0.533	0.590
Q9CXE7	Tmed5	1.049	0.069	0.263	0.321
Q9CXF4	Tbc1d15	0.860	(0.218)	0.008	0.020
Q9CXG3	Ppil4	0.939	(0.091)	0.000	0.004
Q9CXJ4	Abcb8	1.145	0.196	0.003	0.010
Q9CXL3		1.161	0.215	0.002	0.009
Q9CXT8	Pmpcb	0.924	(0.114)	0.078	0.114
Q9CXV9	Dcun1d5	1.069	0.096	0.052	0.081
Q9CXW2	Mrps22	1.179	0.238	0.001	0.007
Q9CXW3	Cacybp	0.914	(0.129)	0.157	0.207
Q9CXW4	Rpl11	1.041	0.058	0.415	0.475
Q9CXY6	Ilf2	1.135	0.182	0.000	0.003
Q9CY16	Mrps28	1.045	0.063	0.301	0.361
Q9CY25	Mis12	0.895	(0.160)	0.002	0.009
Q9CY27	Tecr	0.882	(0.181)	0.001	0.006
Q9CY50	Ssr1	0.909	(0.138)	0.019	0.037
Q9CY73	Mrpl44	1.012	0.017	0.517	0.574
Q9CY94	Gins3	1.000	0.000	0.987	0.989
Q9CYA6	Zcchc8	1.046	0.065	0.117	0.160
Q9CYC5	Dsn1	0.981	(0.028)	0.554	0.610
Q9CYG7	Tomm34	1.011	0.015	0.340	0.401
Q9CYH6	Rrs1	0.921	(0.118)	0.107	0.148
Q9CYI0		1.192	0.253	0.010	0.023
Q9CYK1	Wars2	1.084	0.116	0.009	0.020
Q9CYN9	Atp6ap2	1.018	0.026	0.598	0.651
Q9CYR0	Ssbp1	1.100	0.137	0.003	0.010

Q9CYU6	Dph7	0.868	(0.205)	0.034	0.059
Q9CYX7	Rrp15	1.019	0.027	0.614	0.665
Q9CYZ6	Rex1bd	1.039	0.056	0.373	0.434
Q9CYZ7	Tfdp1	0.937	(0.094)	0.136	0.183
Q9CZ13	Uqcrc1	1.089	0.123	0.012	0.026
Q9CZ15	Gins1	1.026	0.037	0.146	0.194
Q9CZ23	Polr3f	1.172	0.229	0.003	0.010
Q9CZ28	Snf8	0.976	(0.036)	0.296	0.356
Q9CZ44	Nsfl1c	1.001	0.001	0.965	0.969
Q9CZ57	Nsun4	0.919	(0.121)	0.183	0.236
Q9CZ83	Mrpl55	1.167	0.223	0.003	0.010
Q9CZ92	Cenpp	0.949	(0.076)	0.092	0.131
Q9CZA6	Nde1	0.948	(0.077)	0.154	0.202
Q9CZD3	Gars	0.903	(0.147)	0.022	0.041
Q9CZJ1	Utp11	1.165	0.220	0.005	0.014
Q9CZM2	Rpl15	1.107	0.146	0.028	0.050
Q9CZN7	Shmt2	1.013	0.019	0.745	0.785
Q9CZS1	Aldh1b1	1.010	0.014	0.856	0.881
Q9CZT4	Polr3e	1.040	0.056	0.379	0.440
Q9CZU3	Mtrex	1.102	0.141	0.001	0.007
Q9CZU6	Cs	1.099	0.137	0.012	0.027
Q9CZV8	Fbxl20	1.125	0.170	0.053	0.083
Q9CZW5	Tomm70	1.050	0.070	0.075	0.111
Q9CZX0	Elp3	0.975	(0.036)	0.362	0.422
Q9CZX5	Pinx1	1.074	0.103	0.079	0.115
Q9CZX8	Rps19	1.082	0.114	0.226	0.282
Q9CZX9	Emc4	1.039	0.056	0.151	0.200
Q9CZY3	Ube2v1	1.010	0.014	0.863	0.887
Q9D020	Nt5c3a	1.016	0.023	0.819	0.850
Q9D024	Ccdc47	0.999	(0.002)	0.924	0.938
Q9D031	Rsu1	0.929	(0.107)	0.018	0.036
Q9D0B6	Pbdc1	0.988	(0.018)	0.413	0.473
Q9D0C4	Trmt5	1.009	0.012	0.746	0.785
Q9D0D3	Mtpap	1.131	0.178	0.040	0.067
Q9D0D4	Dimt1	1.104	0.143	0.008	0.019
Q9D0D5	Gtf2e1	0.996	(0.006)	0.601	0.655
Q9D0E1	Hnrnpm	1.188	0.248	0.000	0.004
Q9D0F6	Rfc5	1.069	0.097	0.002	0.009
Q9D0F9	Pgm1	0.892	(0.165)	0.000	0.004
Q9D0G0	Mrps30	1.156	0.209	0.001	0.005
Q9D0I8	Mrto4	1.074	0.102	0.005	0.014
Q9D0I9	Rars	1.052	0.073	0.084	0.122
Q9D0J4	Arl2	0.944	(0.083)	0.011	0.024
Q9D0J8	Ptms	1.006	0.009	0.850	0.876
Q9D0K2	Oxct1	1.143	0.192	0.011	0.026
Q9D0L8	Rnmt	1.074	0.102	0.025	0.046
Q9D0M0	Exosc7	1.015	0.022	0.205	0.260
Q9D0M3	Cyc1	1.093	0.128	0.234	0.290
Q9D0N7	Chaf1b	1.133	0.181	0.002	0.007
Q9D0P0	Ebpl	1.046	0.066	0.219	0.274
Q9D0Q7	Mrpl45	1.172	0.229	0.007	0.017
Q9D0R2	Tars	1.111	0.151	0.003	0.011
Q9D0R8	Lsm12	1.144	0.194	0.003	0.010
Q9D0R9	Wdr89	1.014	0.020	0.581	0.637
Q9D0T1	Snu13	1.084	0.116	0.009	0.021
Q9D0W5	Ppil1	1.064	0.090	0.088	0.127
Q9D154	Serpinb1a	1.112	0.153	0.336	0.396
Q9D168	Ints12	0.904	(0.145)	0.036	0.062
Q9D172	Gatd3a	1.098	0.135	0.001	0.007
Q9D187	Ciao2b	0.858	(0.222)	0.003	0.010
Q9D1A2	Cndp2	1.072	0.100	0.028	0.050

Q9D1C1	Ube2c	1.168	0.224	0.036	0.062
Q9D1D4	Tmed10	0.947	(0.078)	0.081	0.117
Q9D1G1	Rab1b	0.988	(0.017)	0.613	0.665
Q9D1H6	Ndufaf4	1.047	0.067	0.080	0.116
Q9D1H7	Get4	0.891	(0.166)	0.019	0.037
Q9D1I6	Mrpl14	1.150	0.202	0.027	0.048
Q9D1J1	Necap2	1.090	0.125	0.016	0.032
Q9D1J3	Sarnp	1.127	0.173	0.003	0.010
Q9D1K2	Atp6v1f	1.013	0.018	0.734	0.776
Q9D1L9	Lamtor5	0.913	(0.132)	0.041	0.068
Q9D1M0	Sec13	0.926	(0.111)	0.050	0.079
Q9D1M1	Polr1d	0.983	(0.025)	0.727	0.769
Q9D1M4	Eef1e1	1.059	0.083	0.147	0.195
Q9D1P4	Chordc1	1.037	0.053	0.111	0.154
Q9D1Q1	Mphosph6	0.955	(0.066)	0.097	0.137
Q9D1Q4	Dpm3	0.939	(0.091)	0.183	0.236
Q9D1Q6	Erp44	0.917	(0.125)	0.009	0.022
Q9D1R2	Kti12	1.121	0.165	0.046	0.074
Q9D1R9	Rpl34	1.099	0.136	0.186	0.239
Q9D211	Cdkn2aipnl	1.175	0.232	0.000	0.005
Q9D289	Trappc6b	0.944	(0.083)	0.005	0.014
Q9D2C2	Saal1	0.893	(0.163)	0.007	0.018
Q9D2E2	Toe1	0.999	(0.001)	0.963	0.968
Q9D2P4	Urm1	0.966	(0.050)	0.445	0.505
Q9D2Q3		0.912	(0.133)	0.018	0.036
Q9D2V7	Coro7	0.932	(0.101)	0.014	0.030
Q9D2Y4	Mlkl	0.880	(0.184)	0.021	0.040
Q9D358	Acp1	1.136	0.184	0.002	0.008
Q9D385	Arl2bp	0.951	(0.073)	0.275	0.334
Q9D394	Rufy3	0.961	(0.057)	0.739	0.780
Q9D3B1	Hacd2	0.918	(0.124)	0.000	0.004
Q9D3D9	Atp5f1d	1.040	0.056	0.104	0.146
Q9D3P8	Plgrkt	1.111	0.152	0.020	0.039
Q9D3U0	Pus10	1.095	0.131	0.003	0.010
Q9D404	Oxsm	1.099	0.136	0.099	0.139
Q9D4G5	Pop1	1.038	0.053	0.309	0.369
Q9D4G9	Rmi1	0.924	(0.114)	0.047	0.076
Q9D4H1	Exoc2	0.861	(0.217)	0.001	0.006
Q9D4H8	Cul2	0.977	(0.034)	0.227	0.283
Q9D4H9	Phf14	1.147	0.198	0.001	0.006
Q9D516	Ccdc130	1.005	0.007	0.875	0.898
Q9D554	Sf3a3	1.122	0.167	0.000	0.003
Q9D5V5	Cul5	1.003	0.005	0.822	0.852
Q9D5V6	Syap1	0.859	(0.220)	0.001	0.007
Q9D620	Rab11fip1	0.900	(0.151)	0.003	0.011
Q9D662	Sec23b	0.979	(0.031)	0.045	0.073
Q9D666	Sun1	0.969	(0.045)	0.185	0.238
Q9D6J5	Ndufb8	0.987	(0.019)	0.396	0.456
Q9D6J6	Ndufv2	1.095	0.132	0.014	0.030
Q9D6K5	Synj2bp	1.043	0.060	0.039	0.065
Q9D6R2	Idh3a	1.149	0.200	0.001	0.007
Q9D6S7	Mrrf	1.117	0.160	0.029	0.052
Q9D6U8	Fam162a	1.184	0.244	0.001	0.007
Q9D6Y4	Borcs8	1.140	0.189	0.009	0.021
Q9D6Y7	Msra	0.935	(0.097)	0.129	0.175
Q9D6Y9	Gbe1	0.924	(0.115)	0.005	0.014
Q9D6Z1	Nop56	1.195	0.257	0.000	0.004
Q9D706	Rpap3	1.080	0.111	0.039	0.066
Q9D735	Trir	1.065	0.091	0.032	0.055
Q9D753	Exosc8	1.043	0.061	0.073	0.108
Q9D786	Haus5	0.922	(0.117)	0.087	0.126

Q9D7A8	Armc1	0.891	(0.167)	0.003	0.010
Q9D7E3	Ovca2	1.011	0.015	0.861	0.885
Q9D7H3	RtcA	0.942	(0.086)	0.133	0.180
Q9D7J4	Cox20	1.021	0.031	0.492	0.550
Q9D7M1	Gid8	0.926	(0.111)	0.001	0.006
Q9D7M8	Polr2d	0.994	(0.008)	0.830	0.860
Q9D7N3	Mrps9	1.050	0.070	0.177	0.229
Q9D7N9	Apmap	0.968	(0.047)	0.212	0.267
Q9D7S9	Chmp5	0.926	(0.112)	0.175	0.227
Q9D7V9	Naaa	1.006	0.008	0.785	0.820
Q9D7X8	Ggct	1.096	0.132	0.025	0.046
Q9D819	Ppa1	1.102	0.140	0.001	0.006
Q9D820	Prorsd1	1.106	0.145	0.015	0.031
Q9D823	Rpl37	1.034	0.048	0.708	0.753
Q9D824	Fip1l1	1.173	0.230	0.004	0.011
Q9D832	Dnajb4	1.099	0.137	0.090	0.128
Q9D868	Ppih	1.036	0.052	0.135	0.182
Q9D880	Timm50	1.180	0.239	0.004	0.012
Q9D892	Itpa	1.169	0.226	0.014	0.029
Q9D8B3	Chmp4b	1.060	0.085	0.012	0.027
Q9D8B6	Fam210b	1.165	0.220	0.002	0.008
Q9D8C4	Ifi35	0.669	(0.580)	0.102	0.143
Q9D8E6	Rpl4	1.020	0.028	0.823	0.853
Q9D8M4	Rpl7l1	1.015	0.021	0.704	0.749
Q9D8M7	Phf10	1.139	0.188	0.001	0.007
Q9D8N0	Eef1g	1.067	0.093	0.197	0.251
Q9D8S3	Arfgap3	0.860	(0.217)	0.000	0.004
Q9D8S4	Rexo2	0.911	(0.134)	0.032	0.055
Q9D8S9	Bola1	0.894	(0.161)	0.000	0.004
Q9D8T2	Gsdmdc1	0.836	(0.259)	0.009	0.021
Q9D8T7	Slirp	1.072	0.101	0.216	0.272
Q9D8V0	Hm13	0.869	(0.202)	0.010	0.022
Q9D8V7	Sec11c	1.221	0.288	0.065	0.098
Q9D8W5	Psmd12	0.994	(0.008)	0.541	0.597
Q9D8X2	Ccdc124	0.871	(0.199)	0.008	0.020
Q9D8X5	Cnot8	1.037	0.053	0.182	0.234
Q9D8Y8	Ing5	1.024	0.034	0.473	0.531
Q9D8Z2	Triap1	1.050	0.071	0.021	0.040
Q9D8Z6	Atg101	0.959	(0.060)	0.409	0.469
Q9D902	Gtf2e2	1.058	0.082	0.090	0.129
Q9D906	Atg7	0.836	(0.258)	0.001	0.006
Q9D937		1.007	0.010	0.888	0.909
Q9D945	Llph	0.889	(0.169)	0.418	0.477
Q9D997	Nagk	1.033	0.047	0.160	0.210
Q9D9V3	Echdc1	0.879	(0.187)	0.001	0.007
Q9D9Z5	Dda1	0.920	(0.121)	0.013	0.028
Q9DA08	Sgf29	0.946	(0.080)	0.203	0.258
Q9DAK9	Phpt1	0.995	(0.007)	0.879	0.901
Q9DAP7	Asf1b	1.010	0.015	0.724	0.767
Q9DAR7	Dcps	1.113	0.154	0.001	0.005
Q9DAT2	Mrgbp	1.155	0.208	0.003	0.010
Q9DAU9	Znf654	0.902	(0.149)	0.001	0.005
Q9DAV9	Tmem38b	1.126	0.171	0.136	0.183
Q9DAW6	Prpf4	1.177	0.235	0.001	0.006
Q9DB05	Napa	1.072	0.101	0.176	0.228
Q9DB15	Mrpl12	1.136	0.184	0.003	0.011
Q9DB25	Alg5	0.902	(0.149)	0.004	0.012
Q9DB27	Mcts1	0.948	(0.077)	0.010	0.023
Q9DB28	Pop5	1.026	0.037	0.615	0.667
Q9DB29	Iah1	1.043	0.061	0.384	0.444
Q9DB42	Znf593	0.881	(0.182)	0.032	0.055

Q9DB85	Rrp8	1.045	0.064	0.004	0.013
Q9DBB4	Naa16	0.921	(0.119)	0.007	0.018
Q9DBB5	Eif4e3	0.892	(0.165)	0.059	0.090
Q9DBB8	Dhdh	0.932	(0.102)	0.029	0.051
Q9DBC0	Selenoo	0.941	(0.088)	0.236	0.292
Q9DBC3	Cmtr1	1.024	0.034	0.192	0.246
Q9DBC7	Prkar1a	0.999	(0.001)	0.959	0.965
Q9DBD5	Pelp1	0.959	(0.061)	0.014	0.030
Q9DBE9	Ftsj3	1.140	0.188	0.008	0.020
Q9DBF1	Aldh7a1	1.011	0.016	0.873	0.896
Q9DBF7	Cwc25	0.921	(0.119)	0.103	0.144
Q9DBG3	Ap2b1	0.938	(0.093)	0.020	0.038
Q9DBG7	Srpra	0.909	(0.138)	0.008	0.019
Q9DBJ1	Pgam1	1.017	0.024	0.455	0.515
Q9DBK7	Uba7	1.189	0.250	0.014	0.030
Q9DBL7	Coasy	0.894	(0.162)	0.012	0.027
Q9DBN5	Lonp2	0.862	(0.214)	0.094	0.134
Q9DBP5	Cmpk1	1.067	0.093	0.075	0.110
Q9DBQ6	Nfatc1	0.902	(0.148)	0.013	0.028
Q9DBQ7	Scyl3	0.853	(0.230)	0.008	0.019
Q9DBR0	Akap8	1.193	0.255	0.001	0.005
Q9DBR1	Xrn2	1.164	0.219	0.008	0.019
Q9DBS1	Tmem43	0.883	(0.180)	0.000	0.004
Q9DBS5	Klc4	0.930	(0.105)	0.016	0.033
Q9DBS8	Poc5	1.027	0.039	0.079	0.116
Q9DBT5	Ampd2	0.851	(0.232)	0.007	0.018
Q9DBZ5	Eif3k	1.140	0.189	0.006	0.016
Q9DC23	Dnajc10	0.871	(0.199)	0.009	0.021
Q9DC33	Hmg20a	1.122	0.166	0.060	0.093
Q9DC37	Mfsd1	1.122	0.166	0.024	0.045
Q9DC40	Telo2	0.946	(0.081)	0.032	0.055
Q9DC48	Cdc40	1.006	0.009	0.589	0.643
Q9DC51	Gnai3	0.880	(0.184)	0.006	0.016
Q9DC61	Pmpca	0.947	(0.079)	0.130	0.177
Q9DC70	Ndufs7	1.010	0.014	0.681	0.728
Q9DC71	Mrps15	1.177	0.235	0.019	0.037
Q9DCA5	Brix1	0.949	(0.076)	0.236	0.292
Q9DCC4	Pycr3	1.029	0.042	0.743	0.783
Q9DCC8	Tomm20	0.948	(0.077)	0.153	0.202
Q9DCD0	Pgd	0.893	(0.163)	0.052	0.081
Q9DCD2	Xab2	1.080	0.111	0.002	0.008
Q9DCE5	Pak1ip1	0.928	(0.107)	0.084	0.121
Q9DCE9	Igtp	1.012	0.018	0.923	0.937
Q9DCF9	Ssr3	0.908	(0.140)	0.013	0.028
Q9DCI9	Mrpl32	0.894	(0.161)	0.017	0.034
Q9DCJ9	Npl	1.103	0.142	0.006	0.016
Q9DCL9	Paics	1.165	0.221	0.050	0.079
Q9DCM2	Gstk1	1.040	0.057	0.089	0.128
Q9DCS2	Mettl26	0.930	(0.105)	0.027	0.049
Q9DCS3	Mecr	1.118	0.162	0.003	0.011
Q9DCS9	Ndufb10	1.047	0.066	0.127	0.173
Q9DCT1	Akr1e2	1.050	0.070	0.269	0.328
Q9DCT5	Sdf2	1.171	0.227	0.092	0.131
Q9DCV4	Rmdn1	0.956	(0.065)	0.169	0.220
Q9DCW4	Etfb	1.140	0.189	0.005	0.014
Q9DCX1	Mad2l1bp	1.138	0.187	0.004	0.012
Q9DCZ4	Apoo	1.079	0.110	0.045	0.072
Q9DD02	Hikeshi	0.951	(0.073)	0.049	0.078
Q9DD18	Dtd1	1.002	0.003	0.894	0.914
Q9EP53	Tsc1	0.831	(0.267)	0.061	0.093
Q9EP69	Sacm1l	1.043	0.060	0.049	0.078

Q9EP72	Emc7	0.970	(0.044)	0.772	0.808
Q9EP82	Wdr4	0.976	(0.036)	0.456	0.515
Q9EP89	Lactb	1.126	0.172	0.004	0.012
Q9EPB4	Pycard	0.923	(0.115)	0.001	0.005
Q9EPE9	Atp13a1	1.025	0.035	0.108	0.150
Q9EPJ9	Arfgap1	1.008	0.011	0.696	0.741
Q9EPK2	Rp2	1.059	0.083	0.060	0.092
Q9EPK7	Xpo7	1.010	0.014	0.708	0.753
Q9EPL8	Ipo7	0.910	(0.136)	0.093	0.133
Q9EPQ7	Stard5	0.922	(0.118)	0.127	0.172
Q9EPU0	Upf1	0.980	(0.030)	0.236	0.292
Q9EPU4	Cpsf1	1.002	0.003	0.926	0.939
Q9EQ06	Hsd17b11	0.986	(0.020)	0.248	0.305
Q9EQ08	Sgsh	0.980	(0.030)	0.685	0.731
Q9EQ32	Pik3ap1	1.110	0.150	0.022	0.041
Q9EQ61	Pes1	1.081	0.112	0.173	0.225
Q9EQ80	Nif3l1	0.982	(0.025)	0.057	0.088
Q9EQC8	Prcc	1.194	0.256	0.004	0.012
Q9EQE2	Ppm1a	0.890	(0.168)	0.001	0.007
Q9EQG9	Col4a3bp	0.846	(0.241)	0.006	0.015
Q9EQH2	Erap1	0.963	(0.055)	0.236	0.292
Q9EQH3	Vps35	0.946	(0.081)	0.009	0.021
Q9EQH4	Taf8	0.935	(0.097)	0.144	0.192
Q9EQI5	Ppbp	0.975	(0.037)	0.589	0.643
Q9EQM6	Dgcr8	0.930	(0.105)	0.275	0.334
Q9EQN3	Tsc22d4	0.973	(0.039)	0.200	0.254
Q9EQP2	Ehd4	1.104	0.143	0.062	0.094
Q9EQQ2	Yipf5	0.920	(0.120)	0.175	0.227
Q9EQQ9	Oga	1.021	0.030	0.272	0.330
Q9EQS3	Mycbp	0.997	(0.004)	0.891	0.912
Q9EQU5	Set	1.077	0.107	0.050	0.080
Q9ER00	Stx12	1.038	0.053	0.189	0.242
Q9ER38	Tor3a	1.107	0.146	0.056	0.087
Q9ER41	Tor1b	1.115	0.157	0.000	0.004
Q9ER72	Cars	0.877	(0.189)	0.026	0.047
Q9ER73	Elp4	1.113	0.155	0.048	0.077
Q9ERA6	Tfip11	1.050	0.070	0.009	0.021
Q9ERB0	Snap29	1.200	0.263	0.001	0.005
Q9ERD8	Parvg	0.878	(0.187)	0.008	0.019
Q9ERE7	Mesd	0.956	(0.065)	0.114	0.157
Q9ERG0	Lima1	0.949	(0.076)	0.006	0.017
Q9ERI2	Rab27a	0.978	(0.033)	0.345	0.406
Q9ERI6	Rdh14	0.988	(0.018)	0.551	0.607
Q9ERK4	Cse1l	1.062	0.087	0.016	0.033
Q9ERL7	Gmfg	1.029	0.041	0.361	0.422
Q9ERR1	Ndel1	0.920	(0.121)	0.024	0.045
Q9ERR8	Znf319	0.998	(0.003)	0.930	0.942
Q9ERS5	Plekha2	1.168	0.224	0.030	0.053
Q9ERU9	Ranbp2	1.121	0.165	0.001	0.007
Q9ERV1	Mkrn2	1.030	0.042	0.347	0.407
Q9ERY9	Erg28	0.974	(0.038)	0.056	0.087
Q9ES00	Ube4b	0.896	(0.158)	0.022	0.041
Q9ES34	Ube3b	0.921	(0.119)	0.005	0.014
Q9ES46	Parvb	1.168	0.224	0.104	0.146
Q9ES52	Inpp5d	1.017	0.024	0.350	0.410
Q9ES57	Cd200r1	1.152	0.204	0.001	0.006
Q9ES74	Nek7	1.035	0.050	0.050	0.079
Q9ES97	Rtn3	0.902	(0.150)	0.006	0.016
Q9ESJ0	Xpo4	0.975	(0.037)	0.245	0.302
Q9ESJ4	Nckipsd	0.928	(0.108)	0.031	0.054
Q9ESL4	Map3k20	0.899	(0.154)	0.002	0.007

Q9ESP1	Sdf2l1	0.855	(0.226)	0.003	0.011
Q9EST3	Eif4enif1	0.866	(0.207)	0.002	0.008
Q9EST4	Psmg2	0.907	(0.141)	0.007	0.018
Q9EST5	Anp32b	1.155	0.208	0.002	0.008
Q9ESU6	Brd4	0.976	(0.036)	0.406	0.467
Q9ESX4	Zcchc17	1.017	0.025	0.583	0.638
Q9ESY9	Ifi30	0.897	(0.157)	0.068	0.102
Q9ET01	Pygl	0.932	(0.101)	0.083	0.120
Q9ET22	Dpp7	1.092	0.127	0.017	0.035
Q9ET26	Rnf114	0.972	(0.041)	0.639	0.689
Q9JHF5	Tcirg1	0.933	(0.101)	0.001	0.007
Q9JHI5	Ivd	1.064	0.090	0.003	0.010
Q9JHI7	Exosc9	1.090	0.124	0.001	0.005
Q9JHJ0	Tmod3	0.950	(0.074)	0.074	0.109
Q9JHJ8	Icoslg	1.125	0.170	0.089	0.127
Q9JHK4	Rabggta	0.900	(0.152)	0.001	0.005
Q9JHN8	Stk19	0.924	(0.114)	0.005	0.014
Q9JHS3	Lamtor2	1.006	0.008	0.937	0.947
Q9JHS4	Clpx	1.141	0.190	0.001	0.006
Q9JHS9	Cwc15	1.080	0.111	0.080	0.117
Q9JHU4	Dync1h1	0.980	(0.029)	0.315	0.375
Q9JHU9	Isyna1	0.848	(0.238)	0.005	0.013
Q9JHW2	Nit2	1.014	0.021	0.611	0.663
Q9JI11	Stk4	1.037	0.053	0.381	0.441
Q9JI19	Fibp	0.985	(0.021)	0.590	0.644
Q9JI44	Dmap1	1.082	0.114	0.013	0.028
Q9JI48	Plac8	0.855	(0.226)	0.011	0.025
Q9JI75	Nqo2	0.871	(0.200)	0.000	0.004
Q9JI90	Rnf14	0.902	(0.149)	0.007	0.017
Q9JIA7	Sphk2	0.865	(0.209)	0.003	0.010
Q9JIF3	Slc2a8	1.105	0.144	0.072	0.106
Q9JIF7	Copb1	0.878	(0.188)	0.002	0.008
Q9JIG7	Ccdc22	0.954	(0.068)	0.008	0.019
Q9JIH2	Nup50	1.001	0.002	0.935	0.946
Q9JII5	Dazap1	1.117	0.160	0.210	0.265
Q9JII6	Akr1a1	1.184	0.243	0.002	0.009
Q9JIK9	Mrps34	1.048	0.068	0.217	0.273
Q9JIW9	Ralb	1.016	0.024	0.827	0.856
Q9JIY5	Htra2	0.886	(0.174)	0.000	0.004
Q9JJ06	C1galt1	1.159	0.213	0.002	0.009
Q9JJ28	Flii	0.891	(0.167)	0.000	0.003
Q9JJ78	Pbk	1.070	0.097	0.037	0.062
Q9JJ89	Ccdc86	1.052	0.073	0.202	0.257
Q9JJ94	Ssna1	0.968	(0.047)	0.336	0.397
Q9JJA2	Cog8	0.944	(0.083)	0.067	0.101
Q9JJA4	Wdr12	0.986	(0.020)	0.765	0.802
Q9JJA7	Ccnl2	0.834	(0.262)	0.001	0.006
Q9JJD0	Thap11	1.164	0.219	0.000	0.004
Q9JJF9	Sppl2a	1.035	0.050	0.191	0.244
Q9JJG9	Noa1	1.092	0.127	0.012	0.026
Q9JJI8	Rpl38	1.007	0.010	0.873	0.896
Q9JJL8	Sars2	1.129	0.175	0.011	0.025
Q9JJQ0	Pigb	0.948	(0.077)	0.297	0.357
Q9JJU8	Sh3bgrl	1.000	0.001	0.987	0.989
Q9JJY4	Ddx20	0.993	(0.010)	0.782	0.817
Q9JJZ2	Tuba8	0.999	(0.001)	0.987	0.988
Q9JJZ6	Klf13	1.085	0.118	0.070	0.104
Q9JK23	Psmg1	0.901	(0.151)	0.043	0.070
Q9JK81	Myg1	0.937	(0.095)	0.017	0.034
Q9JK91	Mlh1	0.955	(0.067)	0.035	0.060
Q9JKB1	Uchl3	0.995	(0.007)	0.839	0.867

Q9JKB3	Ybx3	1.077	0.107	0.357	0.418
Q9JKE1	Trem3	1.043	0.061	0.245	0.302
Q9JKF1	Iqgap1	0.965	(0.051)	0.033	0.057
Q9JKF7	Mrpl39	1.164	0.219	0.011	0.025
Q9JKP7	Pole3	0.972	(0.040)	0.394	0.454
Q9JKR6	Hyou1	0.837	(0.257)	0.003	0.011
Q9JKV1	Adrm1	0.940	(0.090)	0.004	0.013
Q9JKW0	Arl6ip1	1.034	0.048	0.435	0.494
Q9JKX4	Aatf	1.089	0.123	0.001	0.006
Q9JKX6	Nudt5	1.171	0.228	0.000	0.004
Q9JKY0	Cnot9	0.954	(0.068)	0.391	0.452
Q9JKY5	Hip1r	0.912	(0.133)	0.003	0.011
Q9JL26	Fmnl1	0.928	(0.108)	0.001	0.006
Q9JL35	Hmgn5	1.067	0.093	0.004	0.012
Q9JL62	Gltp	1.196	0.258	0.001	0.007
Q9JLB0	Mpp6	1.119	0.162	0.000	0.004
Q9JLC3	Msrb1	0.958	(0.061)	0.444	0.504
Q9JLI8	Sart3	1.100	0.138	0.005	0.014
Q9JLJ2	Aldh9a1	0.896	(0.158)	0.001	0.007
Q9JLJ5	Elovl1	0.949	(0.075)	0.763	0.800
Q9JLN9	Mtor	0.939	(0.091)	0.012	0.027
Q9JLQ2	Git2	0.948	(0.078)	0.170	0.222
Q9JLV5	Cul3	1.010	0.014	0.515	0.573
Q9JM05	Pias4	0.958	(0.062)	0.232	0.288
Q9JM13	Rabgef1	0.983	(0.025)	0.095	0.135
Q9JM76	Arpc3	0.932	(0.102)	0.000	0.003
Q9JM90	Stap1	0.891	(0.166)	0.019	0.037
Q9JM93	Arl6ip4	0.904	(0.146)	0.053	0.083
Q9JMA2	Qtrt1	0.869	(0.202)	0.003	0.010
Q9JMD0	Znf207	1.026	0.038	0.361	0.422
Q9JME7	Trappc2l	1.182	0.241	0.054	0.084
Q9JMF7	Dolpp1	0.836	(0.258)	0.021	0.040
Q9JMG1	Edf1	1.025	0.035	0.391	0.452
Q9JMG2	C1galt1c1	1.071	0.099	0.070	0.105
Q9JMK2	Csnk1e	0.971	(0.043)	0.250	0.307
Q9QUG9	Rasgrp2	0.952	(0.070)	0.031	0.055
Q9QUH0	Glrx	0.911	(0.135)	0.046	0.074
Q9QUI0	Rhoa	1.180	0.239	0.005	0.015
Q9QUJ7	Acsl4	0.889	(0.170)	0.007	0.018
Q9QUM0	Itga2b	0.948	(0.078)	0.490	0.548
Q9QUM9	Psma6	0.878	(0.188)	0.000	0.004
Q9QUR6	Prep	1.130	0.176	0.023	0.044
Q9QWF0	Chaf1a	1.029	0.041	0.073	0.108
Q9QWH1	Phc2	1.195	0.257	0.132	0.178
Q9QWR8	Naga	1.012	0.017	0.790	0.824
Q9QWV9	Ccnt1	0.900	(0.152)	0.043	0.070
Q9QX11	Cyth1	0.998	(0.003)	0.925	0.939
Q9QXA5	Lsm4	0.920	(0.120)	0.003	0.010
Q9QXB9	Drg2	1.039	0.056	0.212	0.268
Q9QXD6	Fbp1	1.010	0.015	0.940	0.949
Q9QXD8	Limd1	0.903	(0.147)	0.033	0.057
Q9QXG4	Acss2	0.992	(0.011)	0.802	0.835
Q9QXJ2	Stat2	0.904	(0.146)	0.465	0.524
Q9QXK3	Copg2	0.878	(0.188)	0.000	0.004
Q9QXK7	Cpsf3	1.037	0.052	0.130	0.176
Q9QXN3	Trip4	0.858	(0.221)	0.001	0.005
Q9QXT0	Cnpy2	0.874	(0.194)	0.000	0.004
Q9QXT1	Dapp1	0.967	(0.048)	0.135	0.182
Q9QXX4	Slc25a13	0.840	(0.251)	0.003	0.010
Q9QY66	Znhit2	0.943	(0.084)	0.488	0.546
Q9QY93	Dctpp1	0.939	(0.090)	0.028	0.050

Q9QYA2	Tomm40	1.144	0.194	0.024	0.044
Q9QYB1	Clic4	1.170	0.227	0.006	0.015
Q9QYB2	Dach1	0.864	(0.211)	0.008	0.019
Q9QYC0	Add1	0.977	(0.034)	0.168	0.219
Q9QYE6	Golga5	0.910	(0.137)	0.004	0.012
Q9QYI3	Dnajc7	1.175	0.233	0.001	0.006
Q9QYJ0	Dnaja2	1.081	0.112	0.001	0.005
Q9QYJ3	Dnajb1	0.985	(0.022)	0.182	0.235
Q9QYL7	Abt1	0.959	(0.061)	0.115	0.158
Q9QYM8	Cenph	1.056	0.078	0.168	0.220
Q9QYP6	Azi2	0.854	(0.228)	0.001	0.005
Q9QYS9	Qki	1.016	0.023	0.697	0.742
Q9QZ06	Tollip	0.891	(0.166)	0.000	0.003
Q9QZ67	Ppm1d	1.184	0.244	0.033	0.057
Q9QZ85	Iigp1	1.310	0.389	0.293	0.352
Q9QZB7	Actr10	1.071	0.099	0.182	0.235
Q9QZC2	Plxnc1	0.930	(0.105)	0.023	0.044
Q9QZD4	Ercc4	0.934	(0.099)	0.034	0.059
Q9QZD8	Slc25a10	1.181	0.241	0.000	0.004
Q9QZD9	Eif3i	0.961	(0.058)	0.129	0.175
Q9QZE5	Copg1	0.879	(0.185)	0.022	0.042
Q9QZE7	Tsnax	0.945	(0.082)	0.067	0.101
Q9QZH3	Ppie	1.071	0.099	0.026	0.048
Q9QZK7	Dok3	1.064	0.090	0.032	0.055
Q9QZL0	Ripk3	0.872	(0.198)	0.057	0.088
Q9QZM0	Ubqln2	0.957	(0.064)	0.035	0.059
Q9QZQ8	H2afy	0.990	(0.014)	0.728	0.770
Q9QZS3	Numb	0.849	(0.236)	0.003	0.010
Q9QZS8	Sh2d3c	0.896	(0.159)	0.001	0.005
Q9QZV9	Nxt1	1.100	0.137	0.009	0.022
Q9R049	Amfr	0.929	(0.107)	0.000	0.004
Q9R059	Fhl3	0.880	(0.185)	0.051	0.081
Q9R060	Nubp1	0.929	(0.106)	0.120	0.164
Q9R062	Gyg1	0.857	(0.222)	0.044	0.071
Q9R099	Tbl2	0.990	(0.014)	0.611	0.663
Q9R0A0	Pex14	0.930	(0.105)	0.154	0.203
Q9R0C8	Vav3	0.945	(0.081)	0.017	0.034
Q9R0E2	Plod1	1.120	0.164	0.092	0.131
Q9R0H0	Acox1	0.887	(0.173)	0.004	0.012
Q9R0L6	Pcm1	1.002	0.003	0.940	0.949
Q9R0M6	Rab9a	0.997	(0.005)	0.904	0.921
Q9R0N0	Galk1	1.028	0.040	0.369	0.429
Q9R0P3	Esd	1.053	0.075	0.039	0.065
Q9R0P5	Dstn	0.965	(0.052)	0.302	0.362
Q9R0Q3	Tmed2	0.848	(0.237)	0.003	0.010
Q9R0Q4	Morf4l2	0.960	(0.059)	0.216	0.272
Q9R0Q6	Arpc1a	1.170	0.227	0.010	0.022
Q9R0Q7	Ptges3	0.956	(0.065)	0.193	0.247
Q9R0X4	Acot9	1.127	0.173	0.002	0.008
Q9R112	Sqor	1.044	0.062	0.150	0.198
Q9R190	Mta2	1.109	0.149	0.003	0.011
Q9R1C0	Taf7	0.951	(0.072)	0.270	0.328
Q9R1E0	Foxo1	1.091	0.126	0.063	0.096
Q9R1J0	Nsdhl	0.874	(0.194)	0.001	0.007
Q9R1P0	Psma4	0.908	(0.139)	0.003	0.010
Q9R1P1	Psmb3	0.917	(0.124)	0.004	0.012
Q9R1P3	Psmb2	0.855	(0.226)	0.001	0.006
Q9R1P4	Psma1	0.887	(0.172)	0.000	0.004
Q9R1Q9	Atp6ap1	0.873	(0.195)	0.003	0.010
Q9R1R8	Rdh11	1.029	0.041	0.099	0.140
Q9R1T2	Sae1	1.115	0.158	0.003	0.011

Q9R1X4	Timeless	1.106	0.146	0.001	0.006
Q9R1X5	Abcc5	0.990	(0.014)	0.717	0.761
Q9R207	Nbn	0.916	(0.127)	0.010	0.024
Q9R210	Tfeb	1.011	0.016	0.759	0.797
Q9R233	Tapbp	1.040	0.056	0.323	0.383
Q9WTK2	Cdyl	1.158	0.212	0.003	0.011
Q9WTL7	Lypla2	1.019	0.028	0.287	0.347
Q9WTN0	Ggps1	0.914	(0.130)	0.056	0.087
Q9WTP6	Ak2	0.896	(0.158)	0.007	0.017
Q9WTR1	Trpv2	1.150	0.202	0.016	0.034
Q9WTU0	Phf2	1.110	0.150	0.017	0.035
Q9WTU6	Mapk9	1.002	0.002	0.933	0.944
Q9WTX2	Prkra	0.885	(0.176)	0.104	0.145
Q9WTX5	Skp1	0.951	(0.073)	0.335	0.395
Q9WTX6	Cul1	1.034	0.048	0.024	0.045
Q9WTX8	Mad1l1	1.038	0.054	0.006	0.016
Q9WU28	Pfdn5	0.951	(0.072)	0.085	0.123
Q9WU56	Pus1	1.049	0.069	0.062	0.094
Q9WU62	Incenp	1.072	0.100	0.056	0.087
Q9WU78	Pdcd6ip	0.907	(0.142)	0.019	0.037
Q9WUA2	Farsb	1.012	0.017	0.320	0.380
Q9WUH1	Tmem115	0.765	(0.386)	0.188	0.241
Q9WUH5	Trim10	1.090	0.124	0.149	0.198
Q9WUK4	Rfc2	1.190	0.250	0.001	0.006
Q9WUM3	Coro1b	1.015	0.022	0.177	0.229
Q9WUM4	Coro1c	0.942	(0.086)	0.024	0.044
Q9WUM5	Suclg1	1.123	0.167	0.062	0.094
Q9WUN2	Tbk1	0.913	(0.131)	0.003	0.010
Q9WUP7	Uchl5	1.079	0.110	0.024	0.044
Q9WUQ2	Preb	1.025	0.036	0.202	0.257
Q9WUT3	Rps6ka2	0.912	(0.133)	0.019	0.037
Q9WUU7	Ctsz	1.063	0.088	0.099	0.139
Q9WUU9	Mcm3ap	1.012	0.017	0.385	0.446
Q9WV02	Rbmx	1.072	0.100	0.004	0.012
Q9WV32	Arpc1b	0.915	(0.128)	0.014	0.030
Q9WV54	Asah1	1.135	0.182	0.008	0.019
Q9WV55	Vapa	0.981	(0.028)	0.631	0.680
Q9WV80	Snx1	0.971	(0.043)	0.507	0.565
Q9WV85	Nme3	1.134	0.181	0.033	0.057
Q9WVE8	Pacsin2	1.004	0.006	0.879	0.901
Q9WVF7	Pole	1.123	0.167	0.002	0.009
Q9WVJ2	Psmd13	0.979	(0.030)	0.326	0.386
Q9WVK4	Ehd1	1.160	0.214	0.007	0.017
Q9WVL3	Slc12a7	0.998	(0.003)	0.940	0.949
Q9WVM1	Racgap1	0.995	(0.007)	0.777	0.813
Q9WVM3	Anapc7	1.133	0.180	0.012	0.027
Q9WVQ5	Apip	0.920	(0.120)	0.085	0.123
Q9WVS8	Mapk7	0.893	(0.163)	0.014	0.030
Q9Z0E6	Gbp2	0.951	(0.073)	0.585	0.640
Q9Z0F6	Rad9a	1.085	0.118	0.004	0.012
Q9Z0F8	Adam17	0.870	(0.201)	0.003	0.011
Q9Z0G0	Gipc1	0.915	(0.128)	0.004	0.012
Q9Z0H1	Wdr46	1.193	0.255	0.001	0.007
Q9Z0H3	Smarcb1	1.161	0.216	0.003	0.011
Q9Z0H8	Clip2	0.949	(0.075)	0.089	0.128
Q9Z0I6	Slfn2	0.774	(0.369)	0.068	0.102
Q9Z0I7	Slfn1	0.960	(0.059)	0.592	0.646
Q9Z0J0	Npc2	0.848	(0.238)	0.014	0.029
Q9Z0L8	Ggh	0.912	(0.133)	0.004	0.012
Q9Z0M5	Lipa	1.123	0.167	0.009	0.021
Q9Z0M9	Il18bp	0.850	(0.234)	0.006	0.017

Q9Z0N1	Eif2s3x	0.986	(0.021)	0.576	0.631
Q9Z0N2	Eif2s3y	0.948	(0.077)	0.322	0.383
Q9Z0P5	Twf2	0.886	(0.175)	0.003	0.011
Q9Z0R9	Fads2	1.087	0.120	0.233	0.289
Q9Z0S1	Bpnt1	1.068	0.095	0.026	0.047
Q9Z0W3	Nup160	1.038	0.054	0.078	0.114
Q9Z110	Aldh18a1	1.040	0.057	0.454	0.514
Q9Z179	Shcbp1	1.134	0.182	0.000	0.003
Q9Z183	Padi4	0.921	(0.119)	0.017	0.034
Q9Z1A1	Tfg	1.025	0.036	0.523	0.581
Q9Z1B5	Mad2l1	1.016	0.023	0.428	0.488
Q9Z1D1	Eif3g	0.975	(0.037)	0.352	0.413
Q9Z1E4	Gys1	0.884	(0.178)	0.015	0.031
Q9Z1F9	Uba2	1.100	0.138	0.005	0.015
Q9Z1G3	Atp6v1c1	0.968	(0.047)	0.167	0.218
Q9Z1G4	Atp6v0a1	1.036	0.051	0.055	0.085
Q9Z1J3	Nfs1	1.178	0.237	0.004	0.013
Q9Z1K5	Arih1	0.960	(0.060)	0.050	0.079
Q9Z1K6	Arih2	0.995	(0.008)	0.765	0.802
Q9Z1M2	Irgm2	0.861	(0.217)	0.211	0.267
Q9Z1M8	Ik	1.193	0.255	0.000	0.004
Q9Z1N2	Orc1	1.044	0.063	0.014	0.030
Q9Z1P6	Ndufa7	1.192	0.254	0.003	0.010
Q9Z1Q2	Abhd16a	1.102	0.140	0.217	0.273
Q9Z1Q5	Clic1	1.164	0.219	0.004	0.013
Q9Z1Q9	Vars	1.084	0.117	0.013	0.028
Q9Z1S0	Bub1b	1.175	0.233	0.006	0.016
Q9Z1T1	Ap3b1	0.900	(0.152)	0.002	0.009
Q9Z1X9	Cdc45	0.942	(0.086)	0.039	0.065
Q9Z1Z0	Uso1	0.915	(0.129)	0.001	0.006
Q9Z1Z2	Strap	1.058	0.081	0.029	0.052
Q9Z247	Fkbp9	1.059	0.082	0.681	0.728
Q9Z277	Baz1b	1.104	0.142	0.000	0.004
Q9Z2B9	Rps6ka4	0.923	(0.116)	0.046	0.074
Q9Z2C4	Mtmr1	1.096	0.132	0.046	0.074
Q9Z2D0	Mtmr9	0.966	(0.050)	0.006	0.016
Q9Z2D3	Gsdme	0.864	(0.211)	0.008	0.020
Q9Z2D6	Mecp2	1.170	0.227	0.029	0.052
Q9Z2G9	Htatip2	1.006	0.008	0.841	0.869
Q9Z2H7	Gipc2	0.929	(0.106)	0.023	0.043
Q9Z2I0	Letm1	1.071	0.098	0.031	0.054
Q9Z2I8	Suclg2	1.168	0.224	0.001	0.006
Q9Z2I9	Sucla2	1.072	0.100	0.046	0.074
Q9Z2L6	Minpp1	1.094	0.130	0.021	0.040
Q9Z2L7	Crlf3	1.040	0.057	0.025	0.047
Q9Z2M6	Ubl3	0.862	(0.214)	0.005	0.014
Q9Z2M7	Pmm2	1.188	0.249	0.001	0.005

Q9Z2N8	Actl6a	1.068	0.095	0.055	0.085
Q9Z2Q6	Septin5	0.875	(0.193)	0.055	0.085
Q9Z2R6	Unc119	0.819	(0.288)	0.057	0.088
Q9Z2U0	Psma7	0.895	(0.160)	0.000	0.004
Q9Z2U1	Psma5	0.903	(0.148)	0.004	0.012
Q9Z2V5	Hdac6	0.896	(0.159)	0.025	0.045
Q9Z2W0	Dnpep	0.836	(0.259)	0.006	0.016
Q9Z2X1	Hnrnpf	1.051	0.072	0.175	0.227
Q9Z2X8	Keap1	0.875	(0.192)	0.000	0.003
Q9Z2Y8	Plpbp	1.153	0.205	0.006	0.015
Q9Z2Z6	Slc25a20	0.949	(0.076)	0.142	0.190
Q9Z315	Sart1	1.128	0.173	0.000	0.004
Q9Z321	Top3b	0.931	(0.103)	0.008	0.020
Q9Z329	Itpr2	0.961	(0.057)	0.103	0.145
R4GML3	Cntrl	0.880	(0.185)	0.001	0.006
R4H4V1	Scyl1	1.055	0.078	0.167	0.219
S4R1B9	Taf1	1.077	0.107	0.023	0.043
S4R1E5	Gpx4	0.945	(0.082)	0.043	0.071
S4R1L5	Birc6	0.856	(0.224)	0.025	0.046
S4R1M2	Safb	1.098	0.135	0.001	0.005
S4R1P7	Washc3	0.888	(0.172)	0.002	0.009
S4R1T3	Dclre1a	0.824	(0.279)	0.061	0.093
S4R1W5	Rbm6	0.998	(0.003)	0.785	0.820
S4R225	Wdr13	0.968	(0.047)	0.176	0.228
S4R270	Bin2	0.970	(0.044)	0.154	0.202
S4R293	Ncf1	0.893	(0.163)	0.009	0.021
S4R2D2	Gga3	0.879	(0.187)	0.003	0.011
S4R2L4	Rsrc2	0.909	(0.138)	0.012	0.026
S4R2R2	Rpf2	0.972	(0.040)	0.517	0.574
V9GWS9	Mx1	1.138	0.186	0.028	0.051
V9GWW1	Zfp280d	0.864	(0.211)	0.048	0.076
V9GX22	Tmem254a	1.043	0.060	0.475	0.534
V9GX42	Meaf6	1.026	0.038	0.061	0.094
V9GXD9	Synrg	1.031	0.044	0.025	0.047
V9GXJ9	Kri1	0.953	(0.069)	0.024	0.045
V9GXK6	Crtc2	0.946	(0.080)	0.468	0.527
V9GXY4	Ripk2	0.850	(0.234)	0.017	0.034
Z4YJC9	Npc1l1	1.061	0.085	0.046	0.075
Z4YJL4	Tnks1bp1	0.843	(0.247)	0.037	0.062
Z4YJW0	Emc1	1.062	0.087	0.009	0.021
Z4YJZ7	Ehmt1	1.025	0.036	0.171	0.222
Z4YK42	Nadk2	1.013	0.019	0.502	0.561
Z4YKB8	Hp1bp3	1.125	0.170	0.003	0.011
Z4YKC4	Eif4g3	0.926	(0.112)	0.005	0.014
Z4YKQ9	Atad5	1.086	0.118	0.006	0.017
Z4YKT6	Dhrs7b	1.118	0.161	0.007	0.018
Z4YL78	Ckap5	0.914	(0.129)	0.023	0.043
Z4YLT1	Inip	1.009	0.012	0.856	0.881
Z4YLT8	Rnf11	0.972	(0.041)	0.134	0.181
Z4YNA3	Mau2	1.018	0.026	0.520	0.578
Z4YNA9	AB124611	0.906	(0.142)	0.036	0.061

**Analytical column :**	C18, 50 µm*15 cm, C18, 2 µm, 100 Å
**Mobile phase A:**	0.1% formic acid
**Mobile phase B:**	0.1% formic acid/80% CAN
**MS scan model:**	MS was set in DDA top15 mode with a full scan range of 350–1800 m/z
**MaxQuant parameters:**	Isobaric labels: TMT tags at the K and peptide N-term<br>Variable modifications: Oxidation (M), Acetyl (Protein N-term)<br>Fixed modification: Carbamidomethyl (C)<br>Enzyme digestion: Trypsin/P with a maximum of 2 missed cleavages<br>Mass tolerance: First search and main search were set to 20 ppm and 4.5 ppm, the fragment match tolerance was set to 20 ppm

Table S4 Summary of KEGG pathway enrichment.

Entry	Entry name	Entry class	Qe ratio	Bg ratio	Rich factor	Fold enrichme	P value	P adjust	Count
map05322	Systemic lupus erythematosus	Human Diseases	13|515	33|3192	0.3939	2.4417	0.0011	0.1516	13
map00860	Porphyrin metabolism	Metabolism	10|515	23|3192	0.4348	2.6948	0.0017	0.1516	10
map04613	Neutrophil extracellular trap formation	Organismal Systems	27|515	96|3192	0.2813	1.7432	0.0018	0.1516	27
map00565	Ether lipid metabolism	Metabolism	7|515	13|3192	0.5385	3.3374	0.0019	0.1516	7
map04060	Cytokine-cytokine receptor interaction	Environmental Information Processing	11|515	28|3192	0.3929	2.4350	0.0027	0.1516	11
map04610	Complement and coagulation cascades	Organismal Systems	11|515	28|3192	0.3929	2.4350	0.0027	0.1516	11
map03430	Mismatch repair	Genetic Information Processing	8|515	19|3192	0.4211	2.6097	0.0063	0.2798	8
map04974	Protein digestion and absorption	Organismal Systems	6|515	12|3192	0.5000	3.0990	0.0066	0.2798	6
map00564	Glycerophospholipid metabolism	Metabolism	11|515	33|3192	0.3333	2.0660	0.0113	0.4212	11
map00640	Propanoate metabolism	Metabolism	9|515	25|3192	0.3600	2.2313	0.0125	0.4212	9
map04213	Longevity regulating pathway - multiple species	Organismal Systems	11|515	34|3192	0.3235	2.0053	0.0143	0.4400	11
map04064	NF-kappa B signaling pathway	Environmental Information Processing	14|515	48|3192	0.2917	1.8078	0.0160	0.4454	14
map04978	Mineral absorption	Organismal Systems	7|515	18|3192	0.3889	2.4104	0.0171	0.4454	7
map04936	Alcoholic liver disease	Human Diseases	16|515	59|3192	0.2712	1.6808	0.0211	0.4770	16
map00510	N-Glycan biosynthesis	Metabolism	9|515	27|3192	0.3333	2.0660	0.0212	0.4770	9
map05150	Staphylococcus aureus infection	Human Diseases	8|515	23|3192	0.3478	2.1558	0.0226	0.4770	8
map04913	Ovarian steroidogenesis	Organismal Systems	4|515	8|3192	0.5000	3.0990	0.0273	0.5422	4
map00513	Various types of N-glycan biosynthesis	Metabolism	7|515	21|3192	0.3333	2.0660	0.0405	0.6813	7
map05152	Tuberculosis	Human Diseases	21|515	89|3192	0.2360	1.4625	0.0410	0.6813	21
map03410	Base excision repair	Genetic Information Processing	9|515	30|3192	0.3000	1.8594	0.0415	0.6813	9
map05206	MicroRNAs in cancer	Human Diseases	19|515	79|3192	0.2405	1.4907	0.0423	0.6813	19
map00650	Butanoate metabolism	Metabolism	5|515	13|3192	0.3846	2.3839	0.0449	0.6897	5
map05034	Alcoholism	Human Diseases	13|515	50|3192	0.2600	1.6115	0.0490	0.7198	13
